# Supplementary material for: BCL-2 and BOK regulate apoptosis by interaction of their C-terminal transmembrane domains
Source: EMBO Rep. 2024 Jul 24;25(9):12. doi: 10.1038/s44319-024-00206-6 (PMC11387410; doi:10.1038/s44319-024-00206-6)
Supplement: Supplementary file 1 — Appendix [file 44319_2024_206_MOESM1_ESM.pdf]

Appendix for

BCL-2 and BOK regulate apoptosis by interaction of their C-terminal transmembrane domains

Tobias B. Beigl[1], Alexander Paul[1], Thomas P. Fellmeth[2], Dang Nguyen[3,4], Lynn Barber[1], Sandra Weller[1], Benjamin Schäfer[1], Bernhard F. Gillissen[5], Walter E. Aulitzky[6], Hans-Georg Kopp[1,6], Markus Rehm[7,8], David W. Andrews[3,4,9], Kristyna Pluhackova[2] and Frank Essmann[1,10,*]

1 Robert Bosch Center for Tumor Diseases, Stuttgart, Germany

2 Cluster of Excellence SimTech, University of Stuttgart, Germany

3 Department of Medical Biophysics, Faculty of Medicine, University of Toronto, Canada

4 Biological Sciences Platform, Sunnybrook Research Institute, Canada

5 Department of Hematology, Oncology, and Tumorimmunology, Charité University Medicine, Berlin, Germany

6 Robert-Bosch-Hospital, Stuttgart, Germany

7 Institute of Cell Biology and Immunology, University of Stuttgart, Germany

8 Stuttgart Research Center Systems Biology, University of Stuttgart, Germany

9 Department of Biochemistry, Faculty of Medicine, University of Toronto, Canada

10 Department of Molecular Medicine, Interfaculty Institute for Biochemistry, Eberhard Karls University Tübingen, Germany

*To whom the correspondence should be addressed:

PD Dr. Frank Essmann

Robert Bosch Center for Tumor Diseases

Auerbachstr. 112

70376 Stuttgart

Germany

Phone: +49-711-81015755

Email: frank.essmann@bosch-health-campus.com

Running title

BCL-2/BOK transmembrane domains interact

This PDF file includes:

Supporting information text 3
Appendix Figures S1 to S11 6
Appendix Tables S1 to S12 18
Appendix References 29

Supporting information text

High-throughput multiscaling molecular dynamics simulation

TMDs of BOK and BCL-2 were modelled as α-helices in PyMOL (Schrödinger, 2023) based on their amino acid sequence and coarse-grained (CG) by martinize2 (https://github.com/marrink-lab/vermouth-martinize). The CG peptides were aligned along the z-axis, randomly rotated along the z-axis and placed in 6 nm relative distance. The peptides were then surrounded by an endoplasmic reticulum (ER) membrane and water with 0.1 M NaCl by insane (Wassenaar *et al,* 2015). The simulation systems were consecutively energy minimized by 5000 steps of the steepest descent algorithm. Then velocities, reflecting the Boltzmann distribution at 310 K, were generated and the systems were equilibrated to 310 K and to 1 bar in 5 ns MD simulation with the time step of 20 fs, v-rescale thermostat with time constant of 1 ps, Berendsen barostat (Berendsen *et al,* 1984) with time constant of 12 ps, semiisotropic pressure coupling and compressibility of 3e-4 1/bar. The van der Waals interactions were shifted to 0 at 1.1 nm by the Potential-shift-Verlet algorithm and the electrostatics among charged particles in a distance larger than 1.1 nm was described by reaction field and the ε_r of 15. The Verlet cut-off scheme (Páll & Hess, 2013) was used for particle-based cut-offs and the neighbor list was updated every 20 steps. The bond constraints were achieved by LINCS (Hess, 2008) and the center of mass of the simulation system was linearly removed every 1000 steps. Dimerization was simulated for 10 µs, tetramerization was simulated 50 µs and Parrinello-Rahman barostat (Parrinello & Rahman, 1980; Parrinello & Rahman, 1981) was used instead of the Berendsen barostat. For the complete list of simulations, see Table S7.

After resolution conversion to atomistic resolutions by backward (Wassenaar *et al,* 2014), all atom (AA) simulations were initiated by a short simulation (1 ps with 0.2 fs timestep) with position restraints on the peptides in which the system was heated up to 310 K by the Berendsen thermostat. Afterwards, a 10 ns simulation with the time step of 2 fs was performed with position restraints on the peptides´ backbone atoms only to equilibrate the membrane and solvent around the oligomers. The temperature was coupled to a bath of 310 K by the Nosé-Hoover thermostat (Evans & Holian, 1985) and coupling constant of 0.5 ps and the pressure was controlled by the Berendsen barostat each 1 ps to be 1 bar. The following production run simulations lasted 1 µs using the time step of 2 fs. The temperature of 310 K was conserved by the Nosé-Hoover thermostat (Evans & Holian, 1985) and coupling constant of 0.5 ps. The pressure of 1 bar was controlled in a semiisotropic manner by the Parrinello-Rahman barostat using the compressibility of 4.5e-5 and coupling constant 1 ps. While the van der Waals interactions were switched to zero between 0.8 and 1.2 nm, the electrostatics was treated by PME (Darden *et al,* 1993) beyond 1.2 nm.

The neighbor list was updated each 10 steps, the Verlet cut-off scheme was used and the center of mass of the system was removed every 500 steps. The bonds to hydrogen atoms were constrained by LINCS (Hess, 2008). All simulations were performed in GROMACS 2020 (Abraham *et al,* 2015).

ER membrane model

The lipid composition of the ER membrane model was based on recent mass spectrometric data (Scrima *et al,* 2022). Because the ER membrane is suggested to be thinner than other membranes (Prasad *et al,* 2020), short-chain lipid tails were assumed. For the ER membrane mimic, 75% 1-myristoyl-2-oleoyl-sn-glycero-3-phosphocholine (MOPC), 7% 1-myristoyl-2-oleoyl-sn-glycero-3-phosphoethanolamine (MOPE), 7% 1-palmitoyl-2-oleoyl-sn-glycero-3-phosphoinositol (POPI), 4% a-palmitoyl-2-oleoyl-sn-glycerol (PODG) and 7% cholesterol were distributed symmetrically in the two membrane leaflets.

Analysis details

Conformation stability of oligomers consisting of BOK-TMD and BCL-2-TMD was evaluated by estimation of root mean square deviations (RMSDs) on per dimer basis as described in the following in order to reduce the dependency of RMSD on the protein/peptide assembly size. At first, the peptides in each AA simulation were clustered to remove splitting of the oligomers over periodic boundary conditions. Next, trajectories of all pairs of dimers were extracted from each simulation and fitted onto the frame at 0 ns (corresponding to the CG conformation, (Irving *et al,* 2001)). Then, average RMSD over 100-1000 ns AA simulation time relative to 0 ns was estimated for each pair of peptides and averaged over a simulation (i.e. in trimers RMSD of three pairs of peptides were averaged per simulation and in tetramers the RMSD of six pairs of peptides were averaged). The average per simulation RMSD values were then used to calculate the average and SEM per simulation set (i.e. BOK-TMD homodimer, BOK-TMD/BCL-2-TMD heterodimer, etc.). Peptide secondary structure was estimated by the dictionary of protein secondary structure (DSSP, (Kabsch & Sander, 1983)) over 100-1000 ns AA simulation. Average interaction energies per residue of one peptide with all other peptides were extracted for each residue of each peptide over the last 100 ns of AA simulations and averaged over all BOK-TMDs or BCL-2-TMDs. These average interaction energies per residue were averaged over all simulations from the simulation set which enabled also the estimation of SEM. Contact maps were calculated using the GROMACS tool mdmat and the settings of 10 levels and truncate distance of 1.2 nm for 1 µs frames. Lipid binding per residue was calculated over 100-1000 ns of AA simulations. In the first step the number of lipids and their type (e.g. cholesterol, POPI etc.) that were in contact (i.e. within 0.3 nm) to each residue of each peptide were estimated in each trajectory frame.

In the next step the relative lipid composition in the surroundings of each residue in each trajectory frame was evaluated and compared to the lipid composition in the bilayer, i.e. cholesterol 0.07, MOPC 0.75 MOPE 0.07, PODG 0.04 and POPI 0.07. Lipid depletion and enrichment was defined as relative abundance that is smaller or larger, respectively, around the residue than in the total bilayer. The relative hidden protein surface was calculated for each trajectory frame of AA simulations as the ratio between the inaccessible solvent surface of the oligomer (i.e. the difference between the sum of solvent accessible surface areas (SASAs) of all peptides and of the oligomer) and the total SASA of all peptides. Standard settings of GROMACS tool sasa were used. Average relative hidden protein surface for each simulation from 100-1000 ns was then calculated and averages per simulation used to calculate the average and SEM over each simulation set.

Gnuplot (Williams & Kelley, 2013) was used to generate contact maps and secondary structure plots, R (R Core Team, 2022) was used to make the interaction energy plot, all images of molecules were rendered in PyMOL (Schrödinger, 2023).

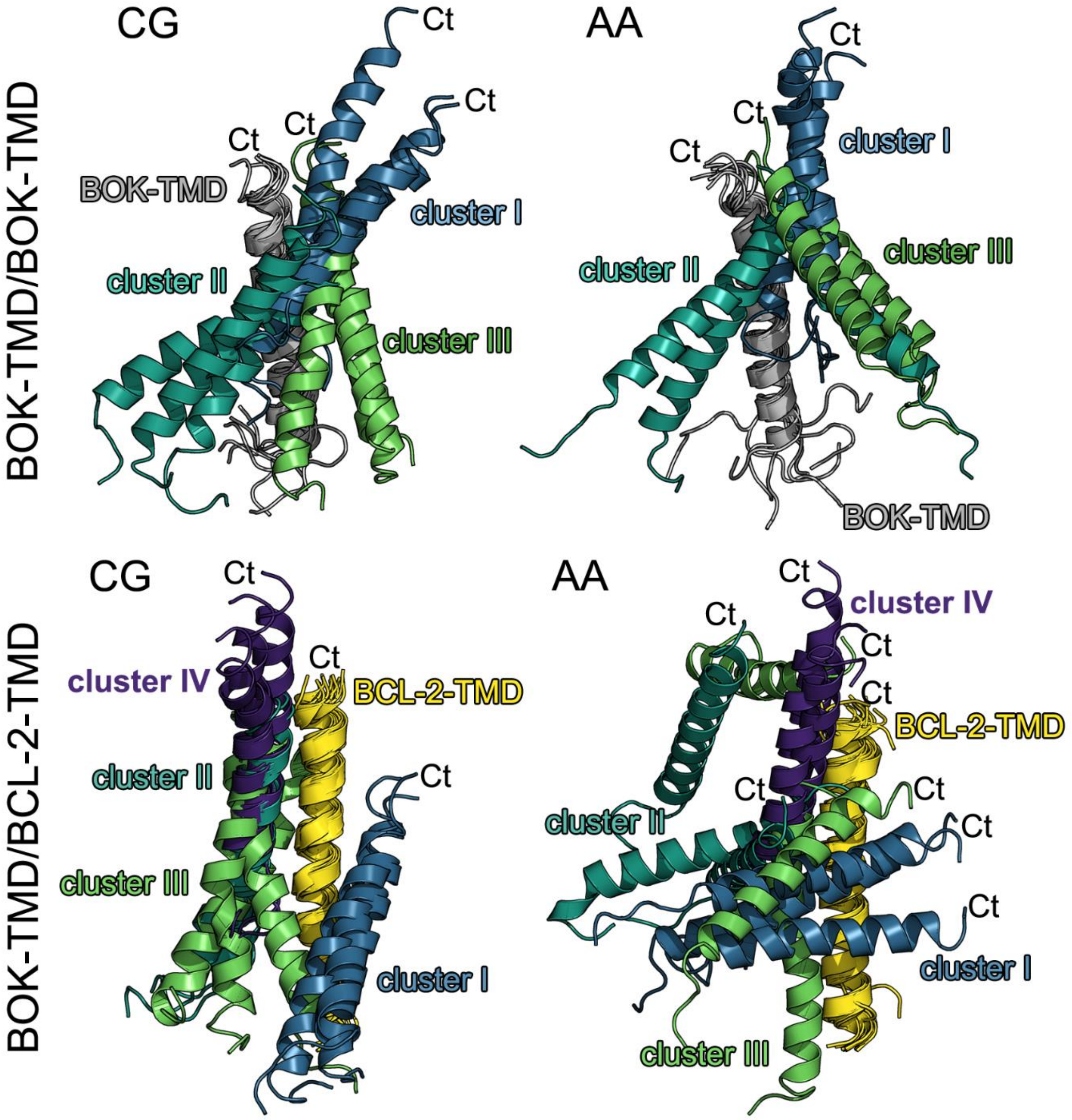

Appendix Figures

Appendix Figure S1: Comparison of CG and AA clusters in BOK-TMD/BOK-TMD and BOK-TMD/BCL-2-TMD dimers

Comparison of CG and AA clusters of BOK-TMD/BOK-TMD homodimers (top) and BOK-TMD/BCL-2-TMD heterodimers (bottom), each after an overlay to a reference helix: BOK-TMD reference in grey and BCL-2-TMD in yellow. CG labels backward representative structures of the given cluster, AA denotes structures after 1 µs AA simulation. The non-overlapping BOK TMDs in cluster I are colored blue, in cluster II teal, in cluster III green and in cluster IV purple.

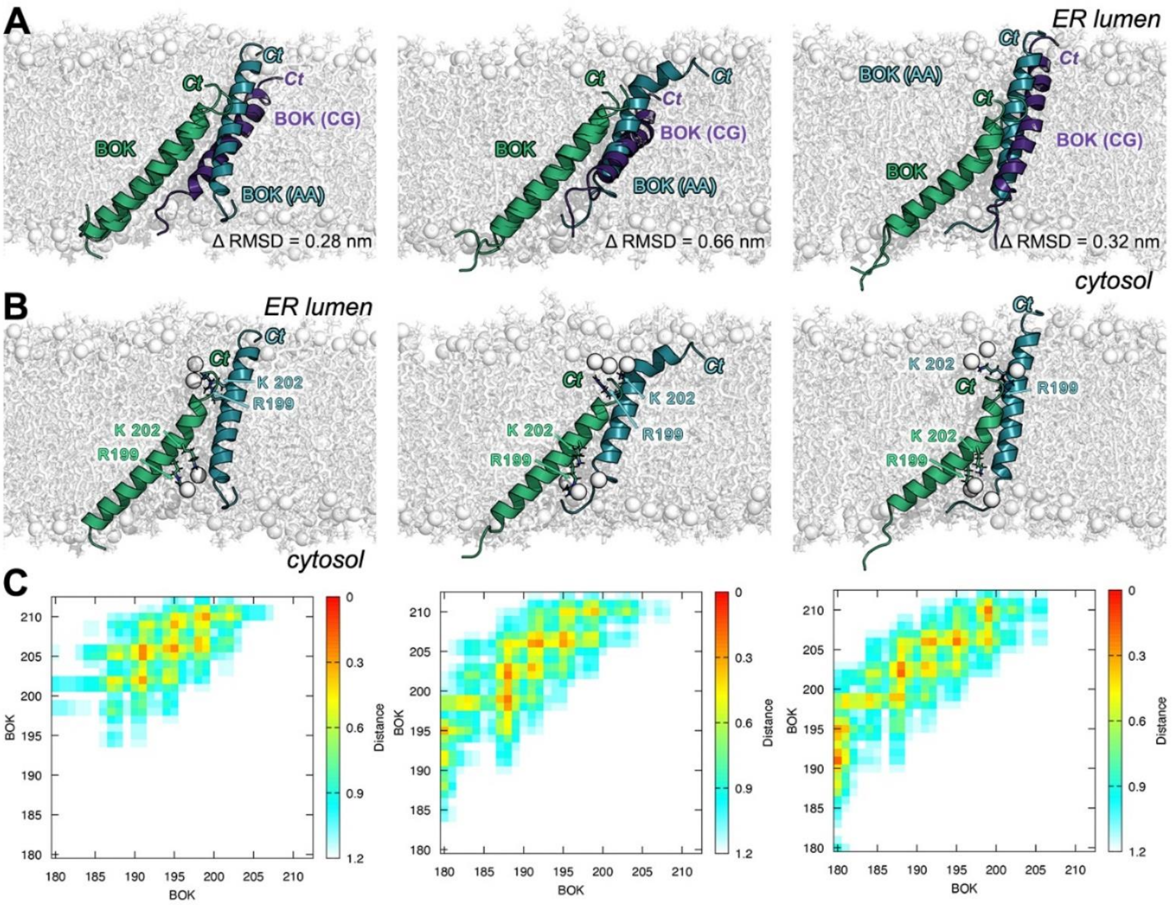

Appendix Figure S2: Structures of three representative BOK-TMD/BOK-TMD-I homodimers.

A - Three representative BOK-TMD/BOK-TMD-I homodimers and their positioning in the membrane after 1 µs AA simulation (one BOK-TMD is colored green and the other teal and labelled as BOK (AA)). For comparison, the dimer structures which spontaneously formed in high-throughput CG simulations were transformed back to atomistic resolution and aligned to the first BOK-TMD (green), the other BOK-TMD is colored purple and labelled as BOK (CG). The deviation between the positions of the backbone atoms in the two dimer structures is given as Δ RMSD.

B - Membrane indentation by $R199^{BOK}$ and $K202^{BOK}$ (shown as sticks). The phosphates of the deformed lipids are shown as nontransparent spheres. Unlike the other BOK-TMD/BOK-TMD homodimers, BOK-TMD/BOK-TMD-I deforms both membrane leaflets, because $R199^{BOK}$ and $K202^{BOK}$ of one BOK-TMD are anchored in the cytosolic membrane leaflet, while the $R199^{BOK}$ and $K202^{BOK}$ of the other BOK-TMD are anchored in the ER lumen leaflet

C - Contact maps of BOK-TMD in the corresponding dimers after 1 µs AA simulations.

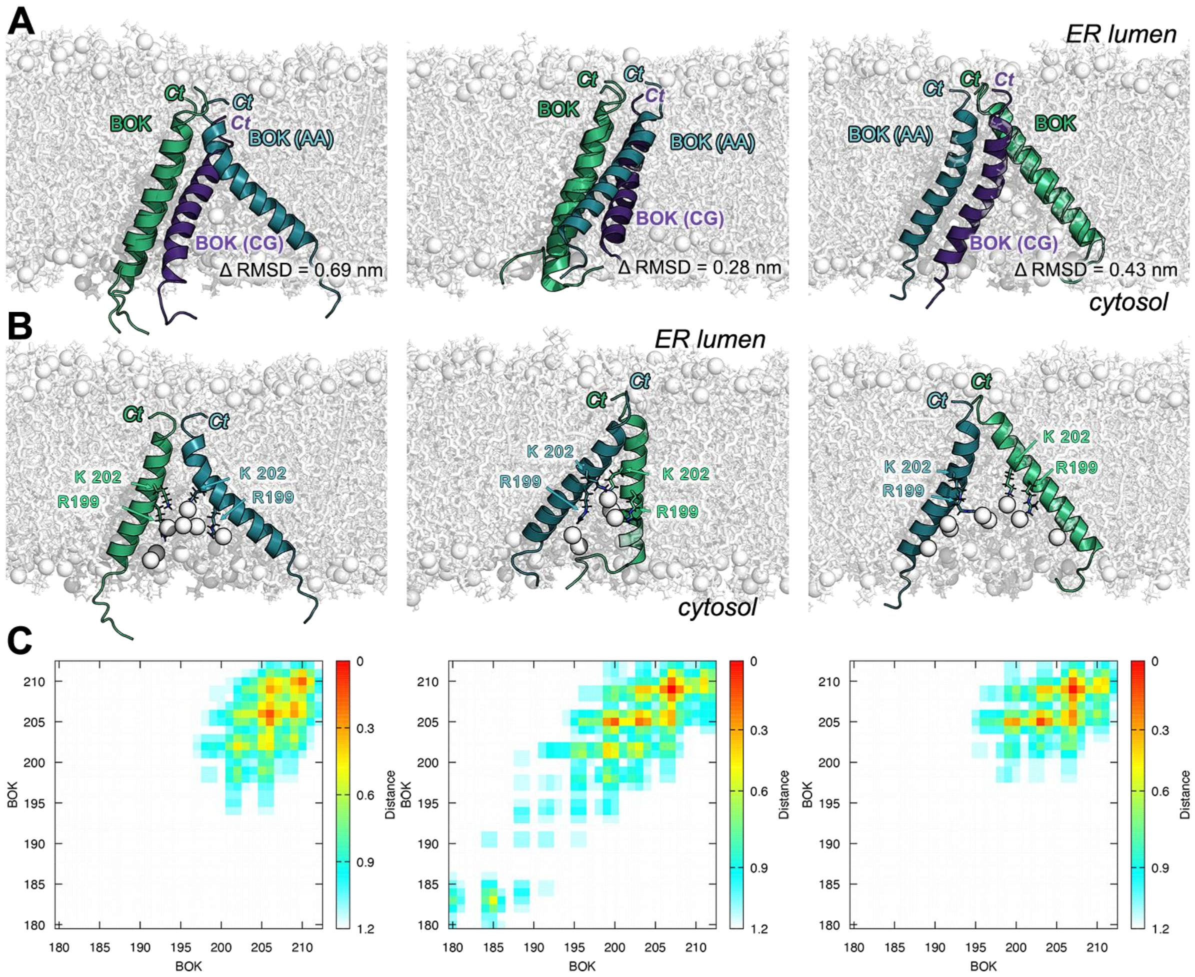

Appendix Figure S3: Structures of three representative BOK-TMD/BOK-TMD-II homodimers.

A - Three representative BOK-TMD/BOK-TMD-II homodimers and their positioning in the membrane after 1 µs AA simulation (one BOK-TMD is colored green and the other teal and labelled as BOK (AA)). For comparison, the dimer structures which spontaneously formed in high-throughput CG simulations were transformed back to atomistic resolution and aligned to the first BOK-TMD (green), the other BOK-TMD is colored purple and labelled as BOK (CG). The deviation between the positions of the backbone atoms in the two dimer structures is given as Δ RMSD.

B - Membrane indentation by $R199^{BOK}$ and $K202^{BOK}$ (shown as sticks). The phosphates of the deformed lipids are shown as nontransparent spheres.

C - Contact maps of BOK-TMD in the corresponding dimers after 1 µs AA simulations.

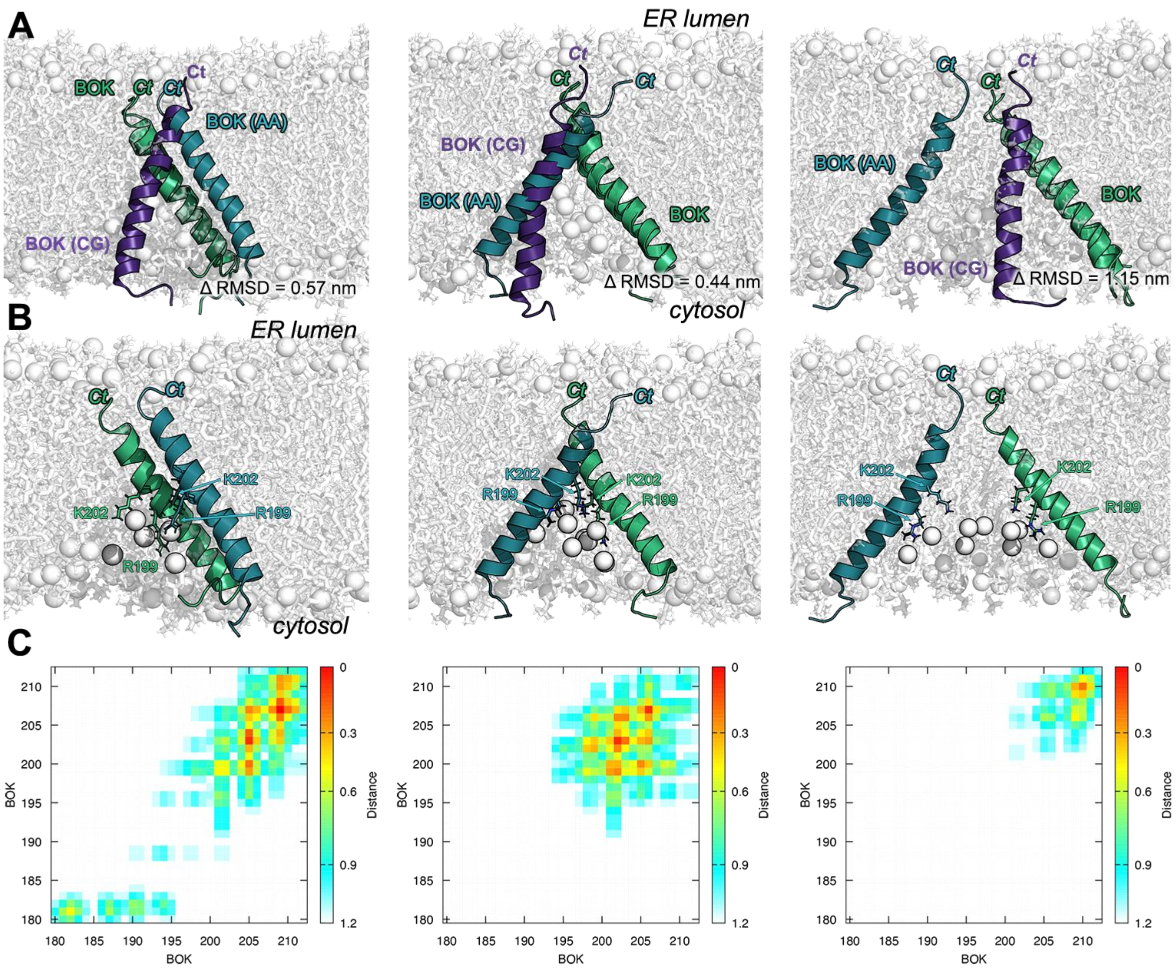

Appendix Figure S4: Structures of three representative BOK-TMD/BOK-TMD-III homodimers.

A - Three representative BOK-TMD/BOK-TMD-III homodimers and their positioning in the membrane after 1 µs AA simulation (one BOK-TMD is colored green and the other teal and labelled as BOK (AA)). For comparison, the dimer structures which spontaneously formed in high-throughput CG simulations were transformed back to atomistic resolution and aligned to the first BOK-TMD (green), the other BOK-TMD is colored purple and labelled as BOK (CG). The deviation between the positions of the backbone atoms in the two dimer structures is given as Δ RMSD.

B - Membrane indentation by $R199^{BOK}$ and $K202^{BOK}$ (shown as sticks). The phosphates of the deformed lipids are shown as nontransparent spheres.

C - Contact maps of BOK-TMD in the corresponding dimers after 1 µs AA simulations.

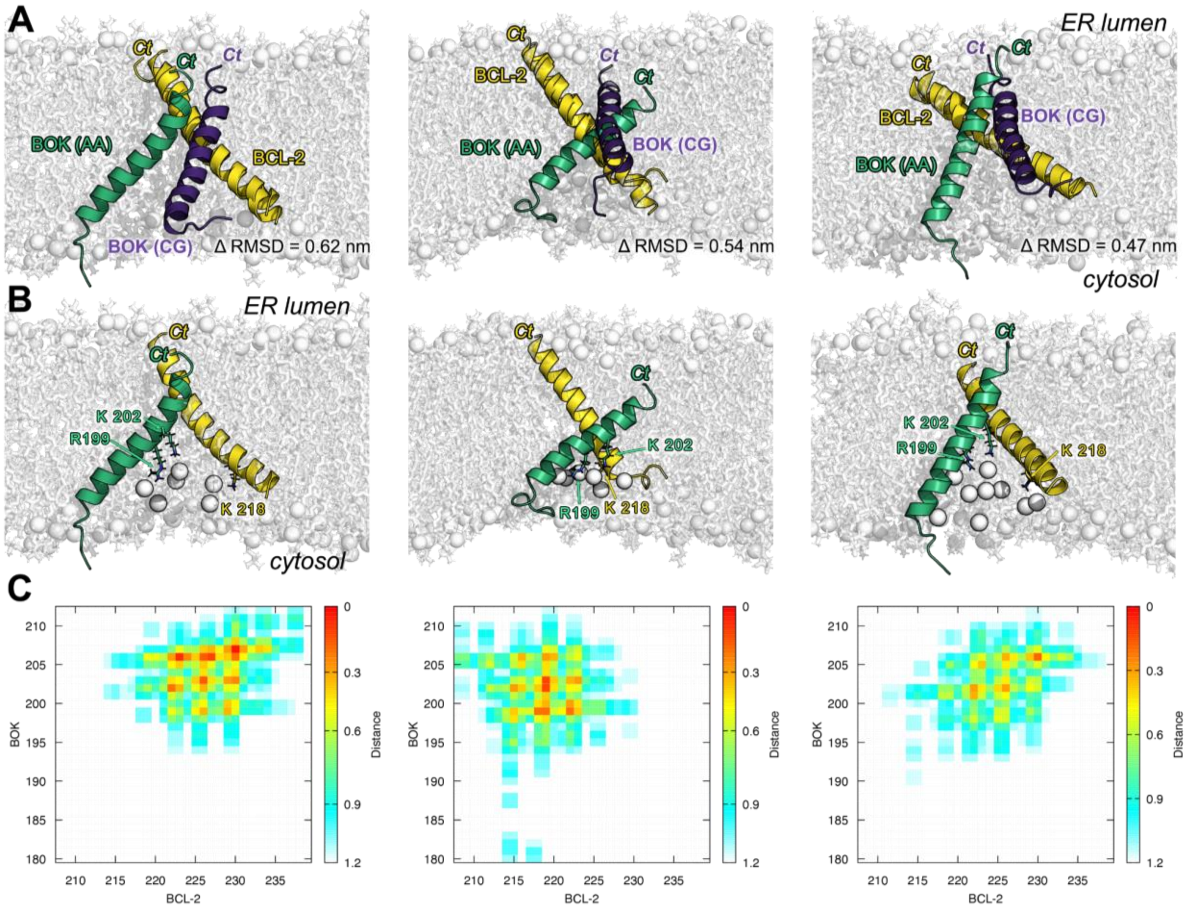

Appendix Figure S5: Structures of three representative BOK-TMD/BCL-2-TMD-I heterodimers.

A - Three representative BOK-TMD/BCL-2-TMD-I heterodimers and their positioning in the membrane after 1 µs AA simulation (BCL-2-TMD is colored yellow, BOK-TMD green and labelled as BOK (AA)). For comparison, the dimer structures which spontaneously formed in high-throughput CG simulations were transformed back to atomistic resolution and aligned to the BCL-2-TMD (yellow), the BOK-TMD is colored purple and labelled as BOK (CG). The deviation between the positions of the backbone atoms in the two dimer structures is given as Δ RMSD.

B - Membrane indentation by $R199^{BOK}$ and $K202^{BOK}$ as well as $K218^{BCL\text{-}2}$ (shown as sticks). The phosphates of the deformed lipids are shown as nontransparent spheres.

C - Contact maps of the BOK-TMD/BCL-2-TMD heterodimers after 1 µs AA simulations.

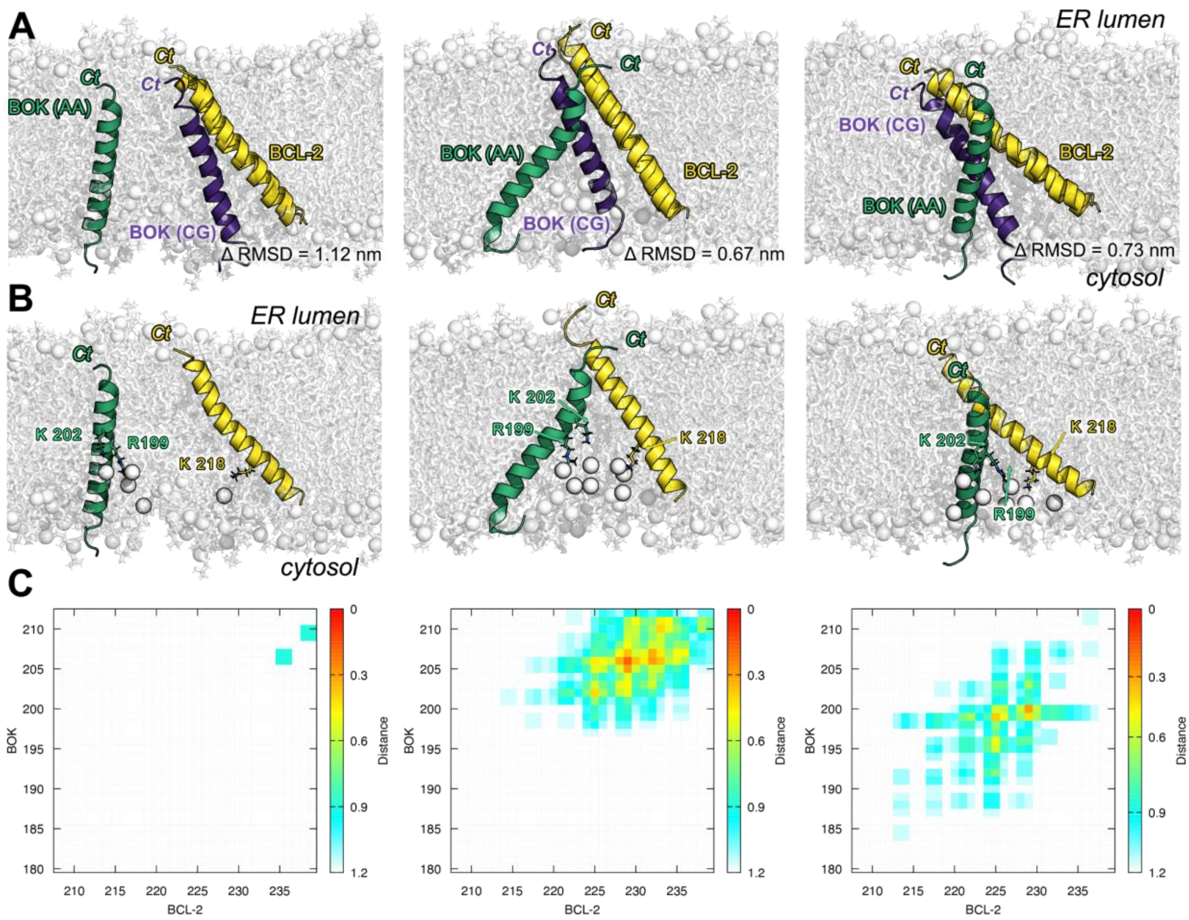

Appendix Figure S6: Structures of three representative BOK-TMD/BCL-2-TMD-II heterodimers.

A - Three representative BOK-TMD/BCL-2-TMD-II heterodimers and their positioning in the membrane after 1 µs AA simulation (BCL-2-TMD is colored yellow, BOK-TMD green and labelled as BOK (AA)). For comparison, the dimer structures which spontaneously formed in high-throughput CG simulations were transformed back to atomistic resolution and aligned to the BCL-2-TMD (yellow), the BOK-TMD is colored purple and labelled as BOK (CG). The deviation between the positions of the backbone atoms in the two dimer structures is given as Δ RMSD.

B - Membrane indentation by $R199^{BOK}$ and $K202^{BOK}$ as well as $K218^{BCL\text{-}2}$ (shown as sticks). The phosphates of the deformed lipids are shown as nontransparent spheres.

C - Contact maps of the BOK-TMD/BCL-2-TMD heterodimers after 1 µs AA simulations.

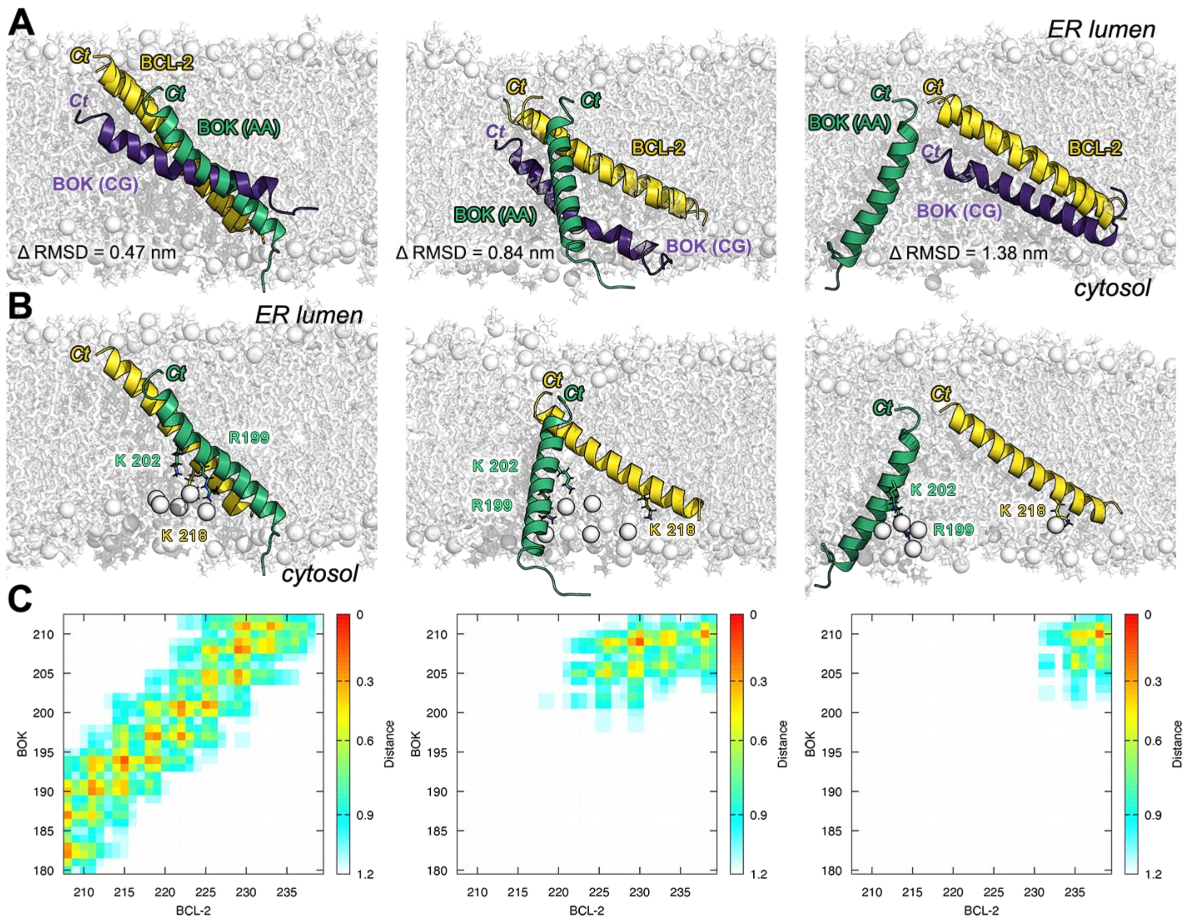

Appendix Figure S7: Structures of three representative BOK-TMD/BCL-2-TMD-III heterodimers.

A - Three representative BOK-TMD/BCL-2-TMD-III heterodimers and their positioning in the membrane after 1 µs AA simulation (BCL-2-TMD is colored yellow, BOK-TMD green and labelled as BOK (AA)). For comparison, the dimer structures which spontaneously formed in high-throughput CG simulations were transformed back to atomistic resolution and aligned to the BCL-2-TMD (yellow), the BOK-TMD is colored purple and labelled as BOK (CG). The deviation between the positions of the backbone atoms in the two dimer structures is given as Δ RMSD.

B - Membrane indentation by R199^{BOK} and K202^{BOK} as well as K218^{BCL-2} (shown as sticks). The phosphates of the deformed lipids are shown as nontransparent spheres.

C - Contact maps of the BOK-TMD/BCL-2-TMD heterodimers after 1 µs AA simulations.

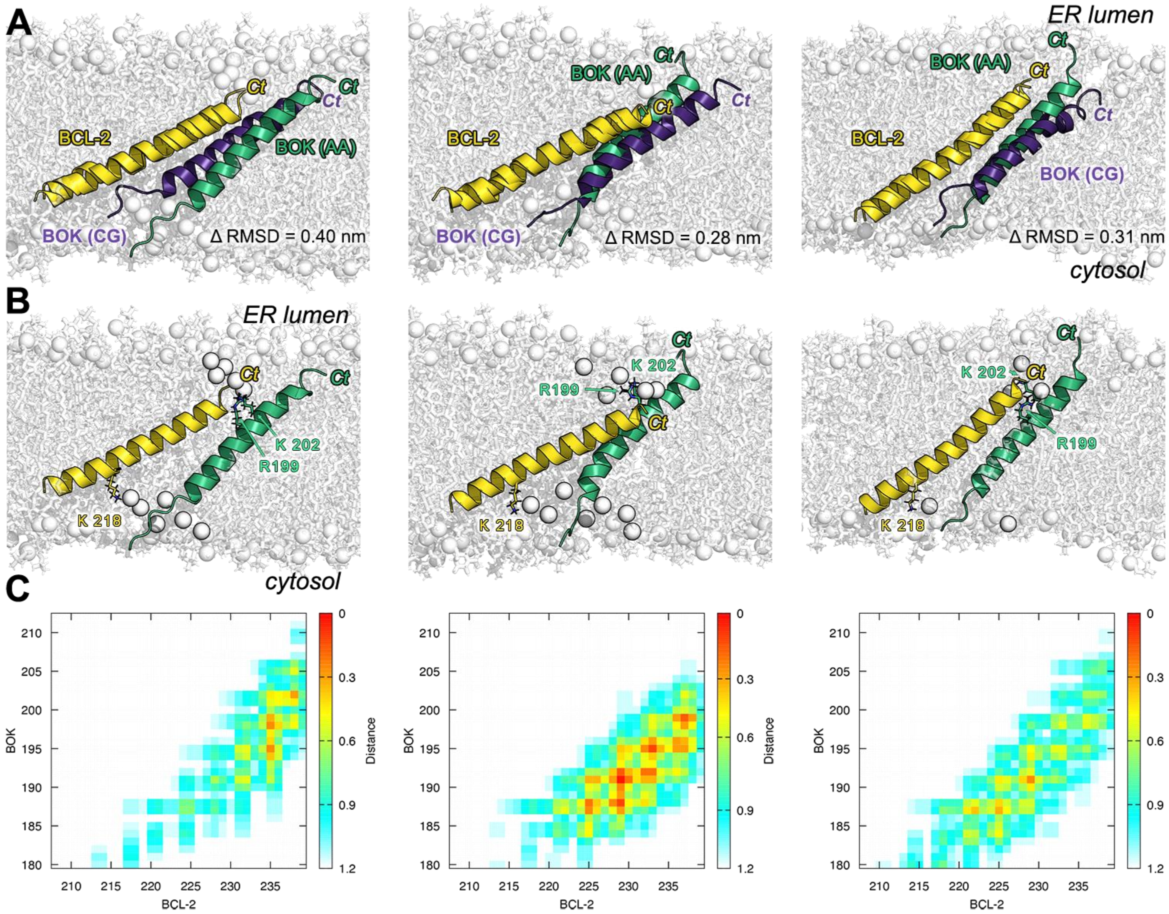

Appendix Figure S8: Structures of three representative BOK-TMD/BCL-2-TMD-IV heterodimers.

A - Three representative BOK-TMD/BCL-2-TMD-IV heterodimers and their positioning in the membrane after 1 µs AA simulation (BCL-2-TMD is colored yellow, BOK-TMD green and labelled as BOK (AA)). For comparison, the dimer structures which spontaneously formed in high-throughput CG simulations were transformed back to atomistic resolution and aligned to the BCL-2-TMD (yellow), the BOK-TMD is colored purple and labelled as BOK (CG). The deviation between the positions of the backbone atoms in the two dimer structures is given as Δ RMSD.

B - Membrane indentation by $R199^{BOK}$ and $K202^{BOK}$ as well as $K218^{BCL\text{-}2}$ (shown as sticks). The phosphates of the deformed lipids are shown as nontransparent spheres.

C - Contact maps of the BOK-TMD/BCL-2-TMD heterodimers after 1 µs AA simulations.

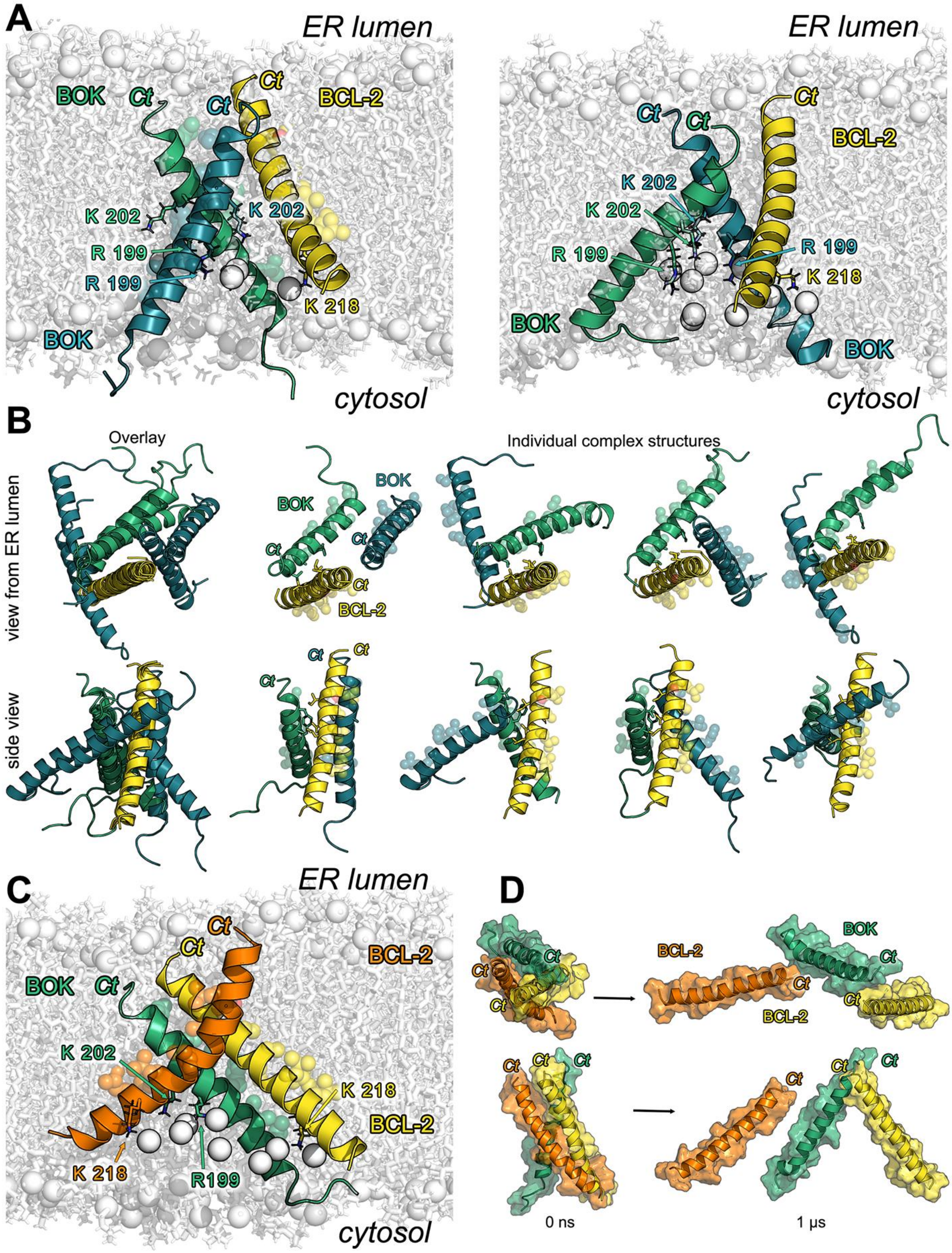

Appendix Figure S9: Structures of BOK-TMD/2xBCL-2-TMD and 2xBOK-TMD/BCL-2-TMD heterotrimers.

A - Oligomer structure and membrane indentation caused by R199^{BOK}, K202^{BOK} and K218$^{BCL\text{-}2}$, shown as sticks, of the two most common 2xBOK-TMD/BCL-2-TMD heterotrimers. The most often observed heterotrimer, comprising 58% of all 2xBOK-TMD/BCL-2-TMD heterotrimers formed is shown on the left. Here, BCL-2-TMD stabilizes the symmetric BOK-

TMD/BOK-TMD-II homodimer by L223^{BCL-2}, V226^{BCL-2}, C229^{BCL-2} and I230^{BCL-2}. The two K202^{BOK} residues are localized deeply in the membrane hydrophobic core, the amino groups are interacting with lipid carbonyls. The second most often (15%) observed 2xBOK-TMD/BCL-2-TMD heterotrimer, shown on the right, consists of BOK-TMD/BCL-2-TMD-I heterodimer (A203^{BOK}, V207^{BOK} F200^{BOK} interact with L223^{BCL-2}, V226^{BCL-2}, C229^{BCL-2} and I230^{BCL-2}) and the second BOK-TMD attached in different positions. The phosphates of the deformed lipids are shown as non-transparent spheres. One BOK-TMD is colored green, another BOK-TMD is colored blue, BCL-2-TMD is colored yellow.

B - Structures of 2xBOK-TMD/BCL-2-TMD trimers with conserved BOK-TMD/BCL-2-TMD-I heterodimer (BCL-2-TMD colored yellow, BOK-TMD colored green). The putative other BOK-TMD peptide is shown in blue. On the left most, the overlay of all four trimers is shown. The top row shows the view from the ER lumen, the bottom row shows a side view.

C - Oligomer structure and membrane indentation caused by R199^{BOK}, K202^{BOK} and K218^{BCL-2}, shown as sticks, of the most common BOK-TMD/2xBCL-2-TMD heterotrimer, comprising 56% of all BOK-TMD/2xBCL-2-TMD heterotrimers formed. Here, one BCL-2-TMD binds to BOK-TMD in the orientation of BOK-TMD/BCL-2-TMD-I heterodimer and the other BCL-2-TMD binds to BOK-TMD with the same interaction interface like in the BOK-TMD/BCL-2-TMD-II heterodimer. Additionally, the two BCL-2-TMDs form a symmetric interaction interface with I230^{BCL-2} and T231^{BCL-2} from both BCL-2-TMDs. The small alanine and glycine residues play again a crucial role for close contacts between the TMDs. A204^{BOK} thus allows a close contact with one BCL-2-TMD and A203^{BOK} with the other BCL-2-TMD. BOK-TMD is colored green, one BCL-2-TMD is yellow and the other BCL-2-TMD orange. The lipid phosphates in the indent are shown as non-transparent spheres.

D - Reorientation of a left-handed BOK-TMD/2xBCL-2-TMD heterotrimer over the course of an atomistic simulation. Top row shows the view from the ER lumen, bottom row a side view, with the C-termini on the top. The orange-colored BCL-2-TMD dissociates completely from the BOK-TMD/BCL-2-TMD heterotrimer, which converts from a compact left-handed to a V-shaped loosed dimer. BOK-TMD is colored green and the BCL-2-TMD which stays in the heterodimer is colored yellow. A surface representation of the peptides is included to highlight the compactness of the initial trimer and the full dissociation of one BCL-2-TMD from the two other peptides after 1 µs.

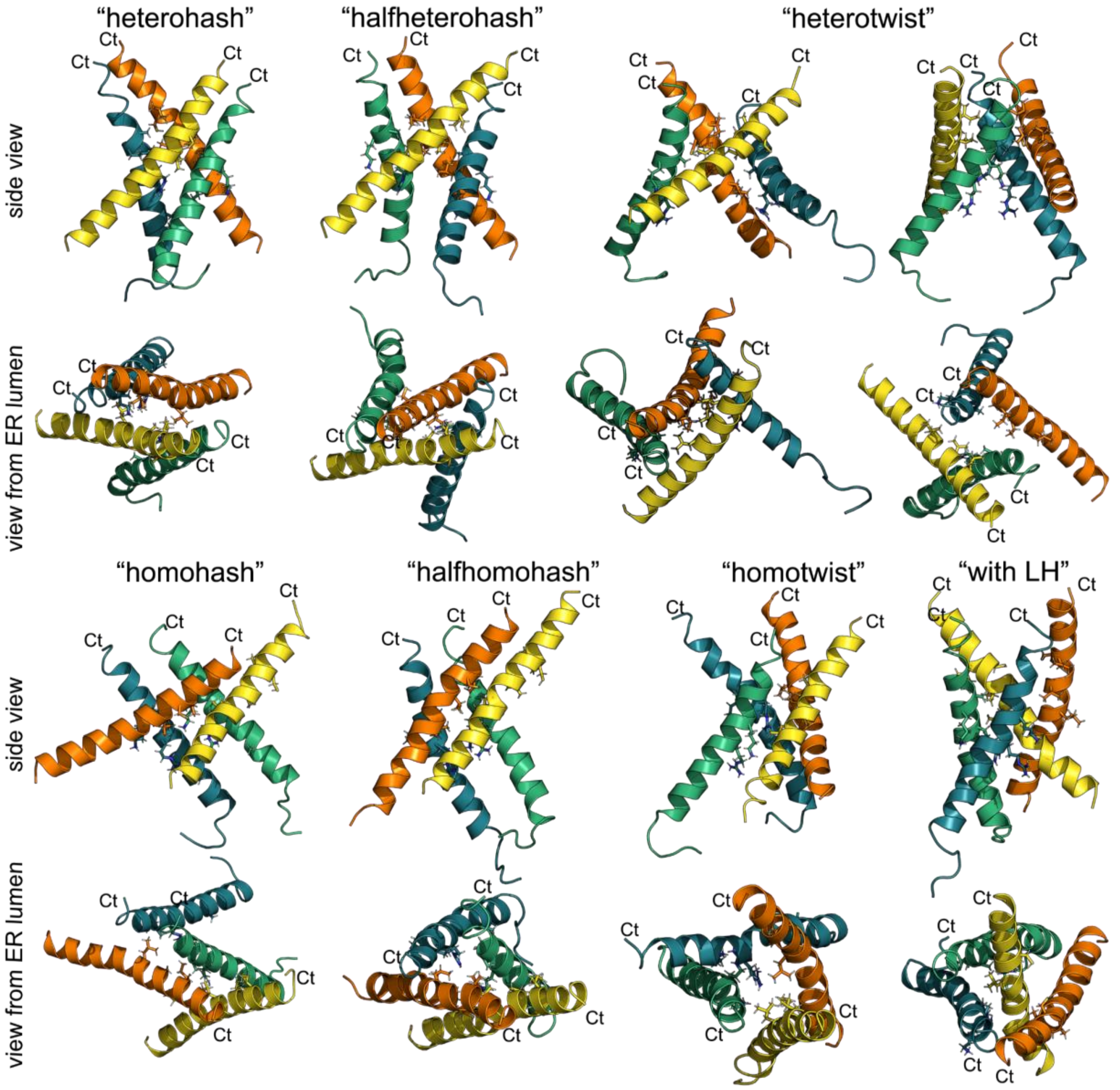

Appendix Figure S10: Structures of representative 2xBOK-TMD/2xBCL-2-TMD heterotetramers.

Representative heterotetramer structures which formed by spontaneous association of two BOK-TMD (colored green and teal) with two BCL-2-TMDs (colored yellow and orange) over 50 µs of CG simulations and converted back to atomistic resolution. The C-termini are located on the top in the side view and in the front in the view from ER lumen. R199^{BOK}, K202^{BOK}, I230$^{BCL\text{-}2}$, C229$^{BCL\text{-}2}$, V226$^{BCL\text{-}2}$, and L223$^{BCL\text{-}2}$ are shown as sticks. "Hash" heterodimers consist of two parallel dimers, "halfhash" tetramers are made of one parallel and one crossed dimer, in the "twist" tetramers each peptide crosses its neighbor forming right-handed compact tetramers. In the structure labelled as "with LH" a compact trimer with lefthandedly attached monomer (here the orange BCL-2-TMD) is shown. After 18 AA simulations, 67 % of all heterodimers had compact "twist" shapes, 17% were homohash, 6% halfhomohash and in two cases (11%) linear dimer of dimers was observed.

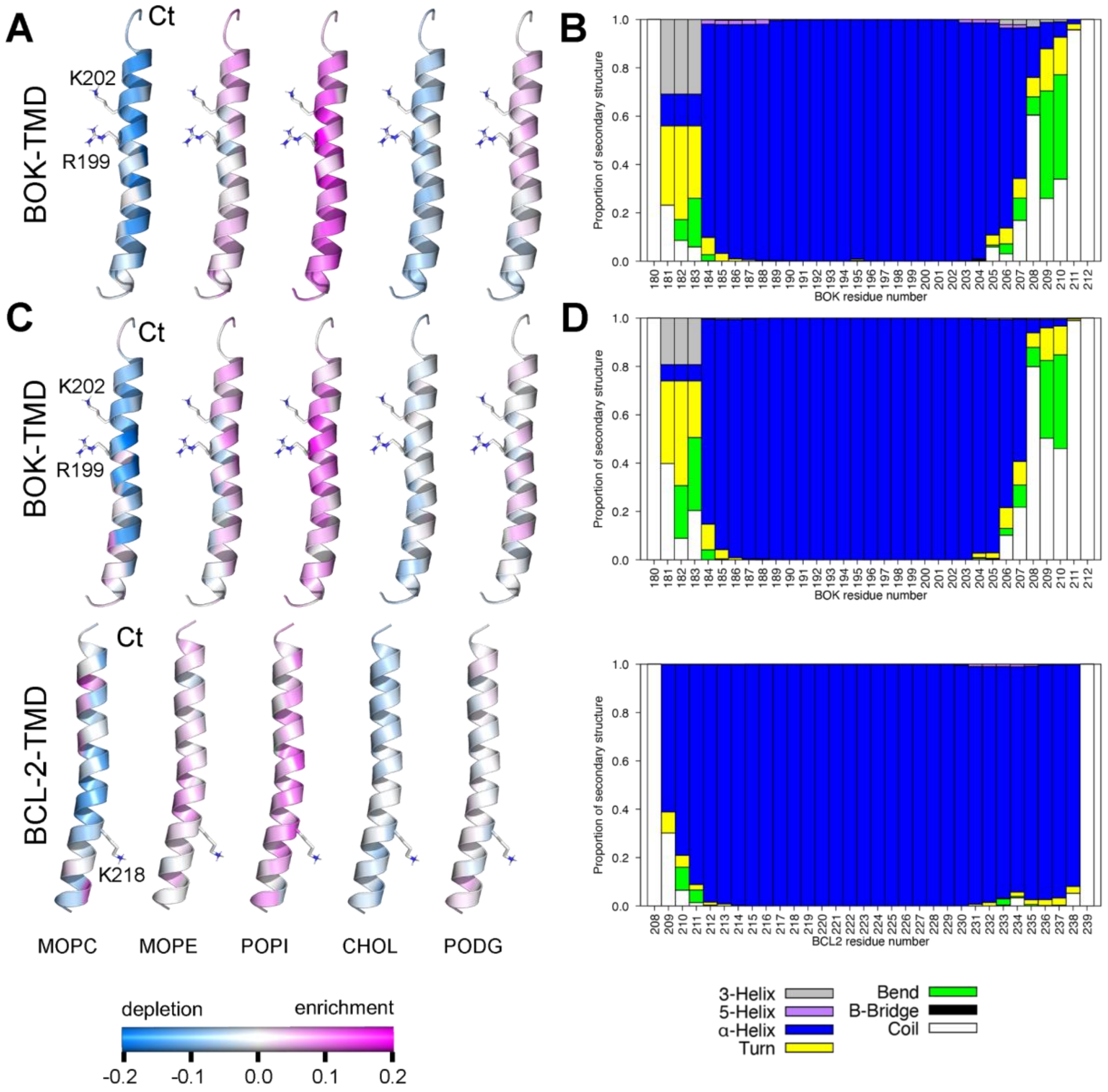

Appendix Figure S11: Lipid binding to BOK-TMD and BCL-2-TMD and secondary structure of TMD peptides.

A - Lipid enrichment/depletion in BOK-TMD/BOK-TMD homodimers.

B - Average BOK-TMD secondary structure per residue in BOK-TMD/BOK-TMD homodimers.

C - Lipid enrichment/depletion in BOK-TMD/BCL-2-TMD heterodimers

D - Average secondary structure per residue of BOK-TMD (top) and BCL-2-TMD (bottom) in BOK-TMD/BCL-2-TMD heterodimers. The membrane indenting positively charged $R199^{BOK}$, $K202^{BOK}$, and $K218^{BCL\text{-}2}$ are in A and C visualized as sticks.

Appendix Tables

Appendix Table S1: Overview of protein sequences used for cloning.

Name	function	Uniprot entry
BAX	Pro-apoptotic	Q07812
BAK	Pro-apoptotic	Q16611
BOK	Pro-apoptotic	Q9UMX3
BCL-2	Anti-apoptotic	P10415
BCL-XL	Anti-apoptotic	Q07817
MCL-1	Anti-apoptotic	Q07820
TOM5	Mito control	Q8N4H5
cb5	ER control	P00167

Appendix Table S2: C-terminal TMD sequences used for plasmid cloning.

Protein	function	amino acid sequence
BAX	Pro-apoptotic	$_{166}$GTPTWQTVTI FVAGVLTASL TIWKKMG$_{192}$
BAK	Pro-apoptotic	$_{184}$GNGPILNVLV VLGVVLLGQF VVRRFFKS$_{211}$
BOK	Pro-apoptotic	$_{185}$LRSHWLVAAL CSFGRFLKAA FFVLLPER$_{212}$
BCL-2	Anti-apoptotic	$_{208}$PLFDFSWLSL KTLLSLALVG ACITLGAYLG HK$_{239}$
BCL-XL	Anti-apoptotic	$_{209}$RFNRWFLTGM TVAGVVLLGS LFSRK$_{233}$
MCL-1	Anti-apoptotic	$_{328}$LEGGIRNVLL AFAGVAGVGA GLAYLIR$_{350}$
TOM5	Mito control	$_{23}$DVISSIRNFL IYVALLRVTP FILKKLDSI$_{51}$
cb5	ER control	$_{100}$DTTIDSSSSW WTNWVIPAIS AVAVALMYRL YMAED$_{134}$

Appendix Table S3: Primer sequences used for Gibson assembly.

Gibson assembly mediated generation of plasmids for the expression of NanoBiT-TMD fusion proteins. Plasmid backbone pBiT_1.1-C[TK/LgBiT] was digested using restriction enzymes HindIII and XbaI. fw = forward; rv = reverse

Fragment	orientation (adjacent fragment)	primer sequence
mCitrine/ mTurquoise2	fw (backbone)	5′-CCTGCAGCGACCCGCTTAAAGATATCATGGTGAGCAAGGGCGAG-3′
	rv (T2A)	5′-AGCCGAATTCCTTGTACAGCTCGTCCATGC-3′
T2A	fw (mCitr/mTurq2)	5′-GCTGTACAAGGAATTCGGCTCCGGCGAGGGCAGAGGAAGTC-3′
	rv (LgBiT)	5′-TGAAGACCATTGCGCAAGGGCCGGGATTCTCCTCC-3′
LgBiT	fw (T2A)	5′-CCCTTGCGCAATGGTCTTCACACTCGAAG-3′
	rv (TMD)	5′-AAGCTTGCTGCCGCCGCCGCCGCTGCCGCCGCCGCCACTGTTGATGGTTACTCG-3′
BAX-TMD	fw (LgBiT/SmBiT)	5′-GGAGGTGGAGGCTCAGGAGGTGGAGGCTCAAAGCTTGGGACGCCCACGTGGCAG-3′
	rv (backbone)	5′-GCGGCCGGCCGCCCCGACTCTCGAGTCAGCCCATCTTCTTCCAGATGGTG-3′
BAK-TMD	fw (LgBiT/SmBiT)	5′-CTCAGGAGGTGGAGGCTCAAAGCTTGGCAACGGCCCCATCCTG-3′
	rv (backbone)	5′-GCGGCCGGCCGCCCCGACTCTCGAGTCATGATTTGAAGAATCTTCGTACC-3′
BOK-TMD	fw (LgBiT/SmBiT)	5′-CTCAGGAGGTGGAGGCTCAAAGCTTCTGAGGAGCCACTGGCTG-3′
	rv (backbone)	5′-GCGGCCGGCCGCCCCGACTCTCGAGTCACCTCTCGGGCAGCAG-3′
BCL-2-TMD	fw (LgBiT/SmBiT)	5′-CAGCGGCGGCGGCGGCAGCAAGCTTCCTCTGTTTGATTTCTCC-3′
	rv (backbone)	5′-GCGGCCGGCCGCCCCGACTCTCGAGTCACTTGTGGCCCAGATAG-3′
BCL-XL-TMD	fw (LgBiT/SmBiT)	5′-CAGCGGCGGCGGCGGCAGCAAGCTTCGCTTCAACCGCTGGTTC-3′
	rv (backbone)	5′-GCGGCCGGCCGCCCCGACTCTCGAGTCATTTCCGACTGAAGAGTGAG-3′
MCL-1-TMD	fw (LgBiT/SmBiT)	5′-CAGCGGCGGCGGCGGCAGCAAGCTTCTAGAAGGTGGCATCAGG-3′
	rv (backbone)	5′-GCGGCCGGCCGCCCCGACTCTCGAGTCATCTTATTAGATATGCCAAACC-3′
TOM5-TMD	fw (LgBiT/SmBiT)	5′-CAGCGGCGGCGGCGGCAGCAAGCTTGACGTGATCAGCAGCATC-3′
	rv (backbone)	5′-GCGGCCGGCCGCCCCGACTCTCGAGTCAGATGCTGTCCAGCTTC-3′

Appendix Table S4: Primer sequences used for Gibson assembly.

Gibson assembly–mediated generation of plasmids for the expression of mCitrine or mTurquoise2-tagged Bcl-2 TMD fusion proteins and chimeric fluorophore-fused full length Bcl-2 proteins with exchanged TMDs. For fluorophore-fused TMDs, plasmid backbone pEGFP-C1 (Clontech) was digested previously to NEBuilder cloning using restriction enzymes XhoI and AgeI. For full-length proteins, plasmid backbones used were described previously (Einsele-Scholz *et al,* 2016).

Fragment	orientation (adjacent fragment)	primer sequence
mTurquoise2	fw (backbone)	5′-CGTCAGATCCGCTAGCGCTAGCCACCATGGTGAGCAAGGGCGAG-3′
	rv (Linker)	5′-GCAGAATTCGAAGCTTGAGCCTCGAGTCAGCCCATCTTC-3′
Linker + TMD	fw (fluo)	5′-CATGGACGAGCTGTACAAGGAATTCGGAGGTGGAGGCTCAGGAG-3′
BAX-TMD	rv (backbone)	5′-GGAGAGGGGCACCGGTTCAGCCCATCTTCTTCCAG-3′
BAK-TMD	rv (backbone)	5′-GGAGAGGGGCACCGGTTCATGATTTGAAGAATCTTCGTACC-3′
BOK-TMD	rv (backbone)	5′-GGAGAGGGGCACCGGTTCACCTCTCGGGCAGCAG-3′
BCL-2-TMD	rv (backbone)	5′-GGAGAGGGGCACCGGTTCACTTGTGGCCCAGATAG-3′
BCL-XL-TMD	rv (backbone)	5′-GGAGAGGGGCACCGGTTCACTTCCTGCTGAACAG-3′
MCL-1-TMD	rv (backbone)	5′-GGAGAGGGGCACCGGTTCATCTTATTAGATATGCCAAACC-3′
TOM5-TMD	rv (backbone)	5′-GGAGAGGGGCACCGGTTCAGATGCTGTCCAGCTTC-3′
BOK Core	rv (BAX-TMD)	5′-TGGGCGTCCCGCCAGGGTCTGTGCTGACC-3′
BCL-2 Core	rv (cb5-TMD)	5′-TAGTAGTGTCCCGCATGCTGGGGCCGTA-3′
	rv (TOM5-TMD)	5′-TGATCACGTCCCGCATGCTGGGGCCGTA-3′
BAX-TMD	fw (BOK Core)	5′-AGACCCTGGCGGGACGCCCACGTGGCAG-3′
	rv (backbone)	5′-GGAGAGGGGCACCGGTTCAGCCCATCTTCTTCCAG-3′
Cb5-TMD	fw (BCL-2 Core)	5′-CAGCATGCGGGACACTACTATTGATTCTAGTTC-3′
	rv (backbone)	5′-CGGCCGCCACTGTGCTGGATGAATTCTCAATCTTCAGCCATGTAC-3′
TOM5-TMD	fw (BCL-2 Core)	5′-CAGCATGCGGGACGTGATCAGCAGCATC-3′
	rv (backbone)	5′-CGGCCGCCACTGTGCTGGATGAATTCTCAGATGCTGTCCAGCTTC-3′

Appendix Table S5: Primer sequences used for mutagenesis.

Oligonucleotide sequences used for site-directed mutagenesis of plasmids for expression of BCL-2 SmBiT-TMDs,mTurq2-fused TMD peptides and full-length BCL-2 protein.

Mutation	primer name	primer sequence
L223A V226A I230A	L223A V226A I230A_fw	5'-ctctgctcagtgcggccctggcgggagcttgcgccaccctgggtg-3'
	L223A V226A I230A_rv	5'-cacccagggtggcgcaagctcccgccagggccgcactgagcagag-3'
L223A V226A	L223A V226A_fw	5'-tctgaagactctgctcagtgcggccctggcgggagcttgc-3'
	L223A V226A_rv	5'-gcaagctcccgccagggccgcactgagcagagtcttcaga-3'
V226A I230A	V226A I230A_fw	5'-tttggccctggcgggagcttgcgccaccctgggtg-3'
	V226A I230A_rv	5'-cacccagggtggcgcaagctcccgccagggccaaa-3'
I230A	I230A_fw	5'-ggtgggagcttgcgccaccctgggtgcc-3'
	I230A_rv	5'-ggcacccagggtggcgcaagctcccacc-3'

Appendix Table S6: Overview of Pearson's correlation coefficients (TMD peptides).

Pearson's correlation coefficients (r-values) for colocalization summarized from quantitative analyses performed on cLSM and confocal spinning disk images in Figure 2, 3 and EV2.

Protein/marker 1	protein/marker 2	figure	cell line	number of cells	mean r-value
mTurq2-BAX-TMD	EYFP-Mito	2C	MCF-7	18	0.51
mTurq2-BAK-TMD	EYFP-Mito	2C	MCF-7	18	0.65
mTurq2-BOK-TMD	EYFP-Mito	2C	MCF-7	22	0.04
mTurq2-BCL-2-TMD	EYFP-Mito	2C	MCF-7	23	0.04
mTurq2-TOM5-TMD	EYFP-Mito	2C	MCF-7	17	0.39
mTurq2-cb5-TMD	EYFP-Mito	2C	MCF-7	24	-0.29
mTurq2-BAX-TMD	EYFP-ER	2C	MCF-7	21	-0.07
mTurq2-BAK-TMD	EYFP-ER	2C	MCF-7	18	-0.04
mTurq2-BOK-TMD	EYFP-ER	2C	MCF-7	18	0.50
mTurq2-BCL-2-TMD	EYFP-ER	2C	MCF-7	15	0.42
mTurq2-TOM5-TMD	EYFP-ER	2C	MCF-7	22	-0.01
mTurq2-cb5-TMD	EYFP-ER	2C	MCF-7	17	0.40
mTurq2-BCL-XL-TMD	EYFP-Mito	EV2C	MCF-7	26	0.47
mTurq2-MCL-1-TMD	EYFP- Mito	EV2C	MCF-7	21	-0.17
mTurq2-BCL-XL-TMD	EYFP-ER	EV2D	MCF-7	26	0.34
mTurq2-MCL-1-TMD	EYFP-ER	EV2D	MCF-7	18	0.35
mCitrine-BOK-TMD	mTurq2-BCL-2-TMD	3B	MCF-7	17	0.52
mTurq2-BAX-TMD	Mitotracker red	2E	BMK DKO	1117	0.81
mTurq2-BAK-TMD	Mitotracker red	2E	BMK DKO	1500	0.79
mTurq2-BOK-TMD	Mitotracker red	2E	BMK DKO	1467	0.56
mCerulean3-BIK	Mitotracker red	2E	BMK DKO	1011	0.46
mTurq2-BAX-TMD	BODIPY-Thapsigargin	2E	BMK DKO	1117	0.65
mTurq2-BAK-TMD	BODIPY-Thapsigargin	2E	BMK DKO	1500	0.76
mTurq2-BOK-TMD	BODIPY-Thapsigargin	2E	BMK DKO	1467	0.92
mCerulean3-BIK	BODIPY-Thapsigargin	2E	BMK DKO	1011	0.88
Mitotracker red	BODIPY-Thapsigargin	EV2E	BMK DKO	1059	0.57
Mitotracker red	Mitotracker green	EV2E	BMK DKO	1033	0.95

Appendix Table S7: Overview of Pearson's correlation coefficients (full-length proteins).

Pearson's correlation coefficients (r-values) for colocalization summarized from quantitative analyses performed on cLSM images in Figure EV4.

Protein/marker 1	protein/marker 2	figure	cell line	number of cells	mean r-value
EGFP-BAX	Mitotracker red	EV3A	MCF-7	29	0.38
EGFP-BAK	Mitotracker red	EV3A	MCF-7	31	0.43
EGFP-BOK	Mitotracker red	EV3A	MCF-7	20	0.29
EGFP-BAX	mCherry-BCL-2	EV3B	MCF-7	17	0.34
EGFP-BAK	mCherry-BCL-2	EV3B	MCF-7	33	0.52
EGFP-BOK	mCherry-BCL-2	EV3B	MCF-7	28	0.47
EGFP-BAX (high)	mCherry-BCL-2$^{cb5\text{-}TMD}$	EV3D	MCF-7	16	0.44
EGFP-BAK (high)	mCherry-BCL-2$^{cb5\text{-}TMD}$	EV3D	MCF-7	18	0.53
EGFP-BAX (low)	mCherry-BCL-2$^{cb5\text{-}TMD}$	EV3D	MCF-7	25	-0.21
EGFP-BAK (low)	mCherry-BCL-2$^{cb5\text{-}TMD}$	EV3D	MCF-7	18	0.10
EGFP-BOK	mCherry-BCL-2$^{cb5\text{-}TMD}$	EV3D	MCF-7	12	0.44
EGFP-BAX	mCherry-BCL-2$^{TOM5\text{-}TMD}$	EV3E	MCF-7	13	0.42
EGFP-BAK	mCherry-BCL-2$^{TOM5\text{-}TMD}$	EV3E	MCF-7	22	0.57
EGFP-BOK	mCherry-BCL-2$^{TOM5\text{-}TMD}$	EV3E	MCF-7	18	-0.08
EGFP-BOK L70E	Mitotracker red	EV3G	MCF-7	18	-0.12
EGFP-BOK L70E	mCherry-BCL-2	EV3G	MCF-7	12	0.75
mCherry-BOK$^{\Delta TMD}$	EYFP-Mito	EV3H	MCF-7	17	-0.38
mCherry-BOK$^{\Delta TMD}$	EGFP-BCL-2	EV3H	MCF-7	18	-0.17

Appendix Table S8: Summary of all performed simulations.

Heterotrimer structures for AA simulations were taken from 2xBOK-TMD/2xBCL-2-TMD self-assembly simulations in which heterotrimers formed instead of heterotetramers.

System	resolution	# of simulations	simulation duration (μs)
**BOK-TMD/BOK-TMD**	CG	50	10
	AA	13	1
**BOK-TMD/BCL-2-TMD**	CG	50	10
	AA	15	1
**2xBOK-TMD/2xBCL-2-TMD**	CG	50	50
	AA	18	1
**2xBOK-TMD/BCL-2-TMD**	AA	9	1
**BOK-TMD/2xBCL-2-TMD**	AA	10	1

Appendix Table S9: BOK-TMD/BOK-TMD homodimers.

Summary and characteristics of BOK-TMD/BOK-TMD homodimers spontaneously formed in MD simulations. The errors denote standard errors of the mean over 3 representative structures for each cluster. Characteristics of CG structures were obtained at atomistic resolution after conversion by backward, i.e. at 0 ns of AA simulations. AA characteristics were taken after 1 µs of AA simulation. RH stands for right-handed.

BOK-TMD/ BOK-TMD Cluster	Handed-ness	Frequency CG [%]	ΔRMSD AA/CG [nm]	COM distance CG [nm]	COM distance AA [nm]	Longitudinal shift CG [nm]	Longitudinal shift AA [nm]
I	RH	55	0.43±0.10	1.92±0.17	2.11±0.04	1.56±0.19	1.81±0.06
II	RH	13	0.46±0.15	1.37±0.13	1.95±0.38	0.35±0.11	0.68±0.26
III	RH	11	0.72±0.27	1.43±0.06	2.01±0.75	0.39±0.03	0.86±0.54

Appendix Table S10: BOK-TMD/BCL-2-TMD heterodimers.

Summary and characteristics of observed BOK-TMD/BCL-2-TMD heterodimers. The errors denote standard errors of the mean over 3 representative structures for each cluster. Characteristics of CG structures were obtained at atomistic resolution after conversion by backward, i.e. at 0 ns of AA simulations. AA characteristics were taken after 1 µs of AA simulation. RH stands for right-handed, LH for left-handed

BOK-TMD/BCL-2-TMD Cluster	Handed-ness	Frequency CG [%]	ΔRMSD (AA/CG) [nm]	COM distance CG [nm]	COM distance AA [nm]	Longitudinal shift CG [nm]	Longitudinal shift AA [nm]
I	RH	44	0.54±0.05	1.24±0.05	1.29±0.13	1.29±0.02	0.94±0.24
II	RH	34	0.84±0.18	1.04±0.05	2.06±0.69	0.43±0.03	0.91±0.41
III	LH	8	0.90±0.32	0.97±0.12	2.15±0.75	0.60±0.11	1.57±0.42
IV	RH	7	0.33±0.04	2.24±0.02	2.24±0.09	-1.57±0.03	-1.29±0.07

Appendix Table S11: Relative lipid enrichment/depletion around BOK-TMDs in heterotetramers.

Relative lipid enrichment (positive values) or depletion (negative values) per BOK-TMD residue in 2xBOK-TMD/2xBCL-2-TMD heterotetramer simulations.

BOK-TMD	cholesterol	MOPC	MOPE	PODG	POPI
Ser180	-6.9%	2.8%	0.1%	-3.1%	7.0%
Thr181	-6.7%	3.4%	-0.2%	-3.6%	7.1%
Asp182	-6.7%	-1.0%	4.0%	-3.0%	6.7%
Pro183	-6.0%	-1.3%	-2.7%	-1.9%	12.0%
Gly184	-6.9%	-3.8%	0.3%	-3.8%	14.2%
Leu185	-5.4%	-3.8%	-0.7%	-1.4%	11.3%
Arg186	-3.7%	-10.9%	2.5%	-2.5%	14.5%
Ser187	-5.9%	-2.9%	0.7%	-1.4%	9.5%
His188	-6.9%	2.3%	0.0%	-2.0%	6.6%
Trp189	-2.7%	-11.9%	1.6%	0.9%	12.0%
Leu190	-4.0%	-11.0%	2.9%	0.8%	11.3%
Val191	-5.3%	2.4%	0.1%	-0.6%	3.4%
Ala192	-4.5%	1.6%	-3.5%	-0.8%	7.1%
Ala193	-3.5%	-4.8%	-1.5%	1.4%	8.4%
Leu194	-3.4%	-9.6%	0.5%	2.7%	9.8%
Cys195	-5.9%	0.5%	-4.0%	-1.4%	10.9%
Ser196	-1.9%	-7.4%	-4.6%	-0.3%	14.1%
Phe197	-1.2%	-18.0%	-0.1%	5.0%	14.3%
Gly198	-4.4%	-4.3%	-4.9%	2.4%	11.2%
Arg199	-6.3%	-7.4%	-5.4%	-2.2%	21.4%
Phe200	-2.9%	-5.5%	-3.6%	0.2%	11.9%
Lue201	-2.8%	-9.4%	-2.9%	3.4%	11.6%
Lys202	-3.9%	-9.6%	-5.9%	1.8%	17.5%
Ala203	-6.3%	1.3%	-3.8%	-4.0%	12.7%
Ala204	-5.8%	9.6%	-5.3%	-0.7%	2.1%
Phe205	-3.6%	-11.5%	-1.4%	3.0%	13.6%
Phe206	-5.1%	2.5%	-1.1%	-0.5%	4.1%
Val207	-4.7%	7.7%	-1.6%	-2.6%	1.3%
Leu208	-5.2%	1.0%	-2.1%	0.0%	6.2%
Leu209	-4.7%	-3.8%	1.2%	1.1%	6.2%
Pro210	-3.8%	4.0%	1.7%	-0.6%	-1.2%
Glu211	-5.9%	5.0%	5.5%	-3.3%	-1.3%
Arg212	-5.4%	-4.2%	1.1%	-0.5%	9.0%

Appendix Table S12: Relative lipid enrichment/depletion around BCL-2-TMDs in heterotetramers.

Relative lipid enrichment (positive values) or depletion (negative values) per BCL-2-TMD residue in 2xBOK-TMD/2xBCL-2-TMD heterotetramer simulations.

BCL-2-TMD	cholesterol	MOPC	MOPE	PODG	POPI
Pro208	-4.6%	-2.0%	1.1%	-1.9%	7.3%
Leu209	-2.6%	-10.7%	2.9%	1.4%	8.9%
Phe210	-1.8%	-9.8%	5.7%	0.7%	5.1%
Asp211	-6.9%	-0.4%	6.7%	-2.1%	2.7%
Phe212	-3.5%	-4.0%	-1.2%	0.4%	8.4%
Ser213	-2.5%	-5.1%	1.7%	2.3%	3.7%
Trp214	-4.6%	-6.1%	0.8%	0.4%	9.5%
Leu215	-6.5%	-2.1%	-4.4%	-3.1%	16.1%
Ser216	-3.3%	-1.4%	-1.3%	1.2%	4.8%
Leu217	-3.3%	-11.3%	3.5%	3.5%	7.6%
Lys218	-4.9%	-3.9%	-4.6%	-2.6%	16.0%
Thr219	-5.3%	-7.6%	-4.5%	-2.8%	20.2%
Leu220	-1.7%	-9.8%	2.0%	2.5%	7.0%
Leu221	-2.1%	-12.6%	-0.2%	1.5%	13.5%
Ser222	-2.1%	-9.1%	-6.1%	-3.4%	20.7%
Leu223	-5.5%	4.0%	-3.0%	-1.9%	6.3%
Ala224	-3.4%	0.3%	-0.8%	-1.0%	4.9%
Leu225	-0.4%	-13.2%	-0.6%	-1.0%	15.3%
Val226	-3.7%	-4.4%	-3.5%	-3.1%	14.8%
Gly227	-5.9%	17.6%	-3.8%	-2.6%	-5.3%
Ala228	-3.5%	-1.3%	-1.0%	-1.2%	7.0%
Cys229	-3.5%	-1.1%	0.5%	-1.3%	5.3%
Ile230	-6.0%	10.2%	-0.6%	-2.4%	-1.2%
Thr231	-3.5%	8.2%	-2.4%	-2.6%	0.2%
Leu232	-2.9%	-8.0%	1.6%	0.5%	8.8%
Gly233	-5.2%	1.5%	2.2%	-1.6%	3.1%
Ala234	-6.5%	15.6%	-2.6%	-3.1%	-3.5%
Tyr235	-4.1%	-3.1%	1.3%	-0.8%	6.7%
Leu236	-3.3%	-8.1%	2.5%	0.7%	8.2%
Gly237	-5.8%	5.0%	-3.0%	-1.4%	5.1%
His238	-6.5%	10.9%	-3.4%	-3.4%	2.4%
Lys239	-5.9%	-4.8%	4.1%	-1.3%	7.9%

Appendix References

Abraham MJ, Murtola T, Schulz R, Páll S, Smith JC, Hess B, Lindahl E (2015) GROMACS: High performance molecular simulations through multi-level parallelism from laptops to supercomputers. *SoftwareX* 1-2: 19–25

Berendsen HJC, Postma JPM, van Gunsteren WF, DiNola A, Haak JR (1984) Molecular dynamics with coupling to an external bath. *The Journal of Chemical Physics* 81: 3684–3690

Darden T, York D, Pedersen L (1993) Particle mesh Ewald: An N ·log(N) method for Ewald sums in large systems. *The Journal of Chemical Physics* 98: 10089–10092

Einsele-Scholz S, Malmsheimer S, Bertram K, Stehle D, Johänning J, Manz M, Daniel PT, Gillissen BF, Schulze-Osthoff K, Essmann F (2016) Bok is a genuine multi-BH-domain protein that triggers apoptosis in the absence of Bax and Bak. *J Cell Sci* 129: 3054

Evans DJ, Holian BL (1985) The Nose–Hoover thermostat. *The Journal of Chemical Physics* 83: 4069–4074

Hess B (2008) P-LINCS: A Parallel Linear Constraint Solver for Molecular Simulation. *J Chem Theory Comput* 4: 116–122

Irving JA, Whisstock JC, Lesk AM (2001) Protein structural alignments and functional genomics. *Proteins* 42: 378–382

Kabsch W, Sander C (1983) Dictionary of protein secondary structure: pattern recognition of hydrogen-bonded and geometrical features. *Biopolymers* 22: 2577–2637

Páll S, Hess B (2013) A flexible algorithm for calculating pair interactions on SIMD architectures. *Computer Physics Communications* 184: 2641–2650

Parrinello M, Rahman A (1980) Crystal Structure and Pair Potentials: A Molecular-Dynamics Study. *Phys. Rev. Lett.* 45: 1196–1199

Parrinello M, Rahman A (1981) Polymorphic transitions in single crystals: A new molecular dynamics method. *Journal of Applied Physics* 52: 7182–7190

Prasad R, Sliwa-Gonzalez A, Barral Y (2020) Mapping bilayer thickness in the ER membrane. *Sci Adv* 6

R Core Team (2022) R: A language and environment for statistical computing. *R Foundation for Statistical Computing, Vienna, Austria.*

Schrödinger L (2023) The PyMOL Molecular Graphics System, Version 2.0. *pymol.org*

Scrima S, Tiberti M, Campo A, Corcelle-Termeau E, Judith D, Foged MM, Clemmensen KKB, Tooze SA, Jäättelä M, Maeda K et al (2022) Unraveling membrane properties at the

organelle-level with LipidDyn. *Computational and Structural Biotechnology Journal* 20: 3604–3614

Wassenaar TA, Ingólfsson HI, Böckmann RA, Tieleman DP, Marrink SJ (2015) Computational Lipidomics with insane: A Versatile Tool for Generating Custom Membranes for Molecular Simulations. *J Chem Theory Comput* 11: 2144–2155

Wassenaar TA, Pluhackova K, Böckmann RA, Marrink SJ, Tieleman DP (2014) Going Backward: A Flexible Geometric Approach to Reverse Transformation from Coarse Grained to Atomistic Models. *J Chem Theory Comput* 10: 676–690

Williams T, Kelley C (2013) Gnuplot 4.6: an interactive plotting program. *gnuplot.sourceforge.net*
