# Supplementary material for: BCL-2 and BOK regulate apoptosis by interaction of their C-terminal transmembrane domains
Source: EMBO Rep. 2024 Jul 24;25(9):12. doi: 10.1038/s44319-024-00206-6 (PMC11387410; doi:10.1038/s44319-024-00206-6)
Supplement: Supplementary file 3 — Source data Fig. 1 [file 44319_2024_206_MOESM3_ESM.zip › Figure 1/1C/1C_western_annotated.pptx]

## Slide 1
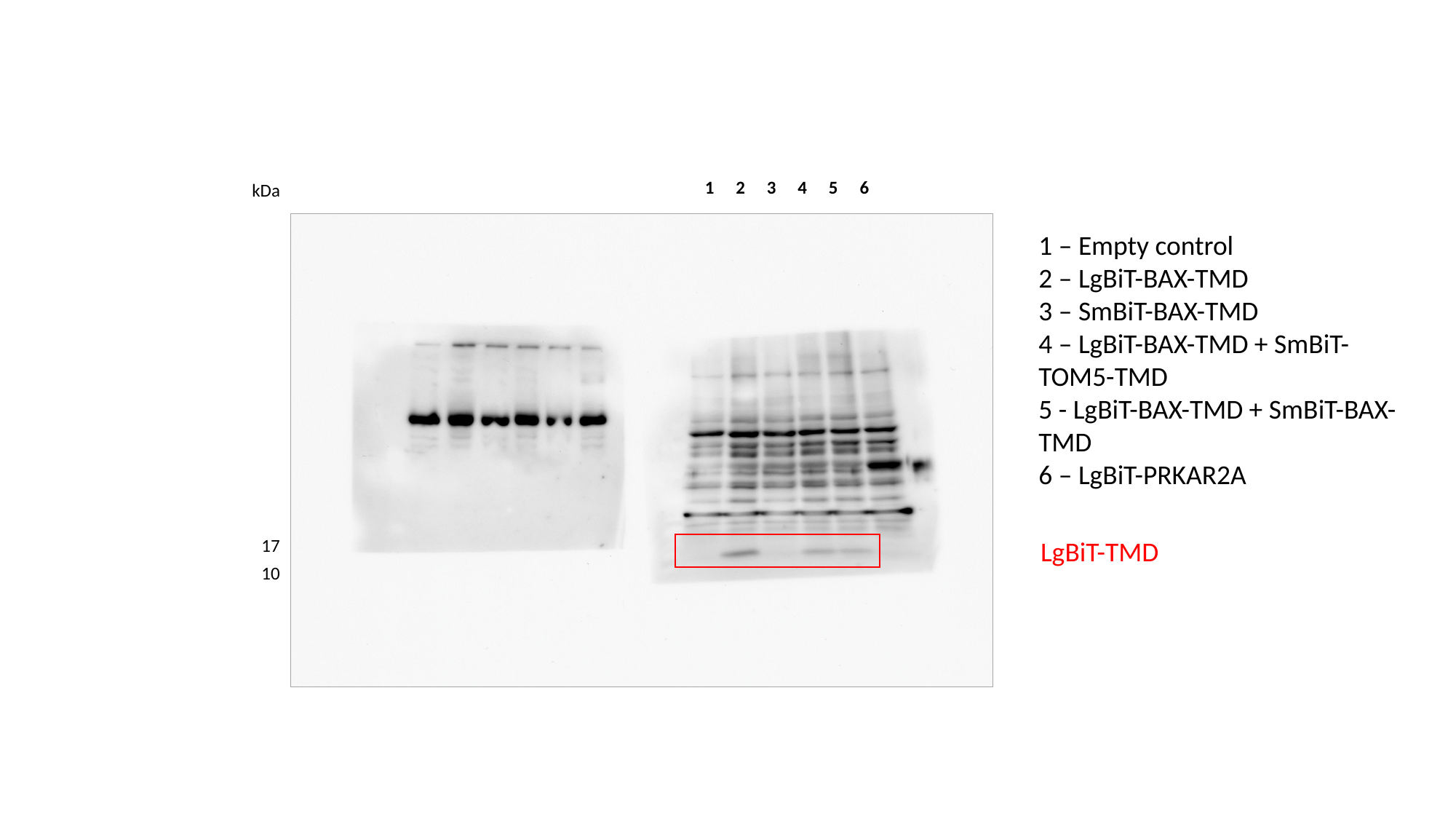

kDa
| 1 | 2 | 3 | 4 | 5 | 6 |
| --- | --- | --- | --- | --- | --- |
1 – Empty control
2 – LgBiT-BAX-TMD
3 – SmBiT-BAX-TMD
4 – LgBiT-BAX-TMD + SmBiT-TOM5-TMD
5 - LgBiT-BAX-TMD + SmBiT-BAX-TMD
6 – LgBiT-PRKAR2A
17
LgBiT-TMD
10

## Slide 2
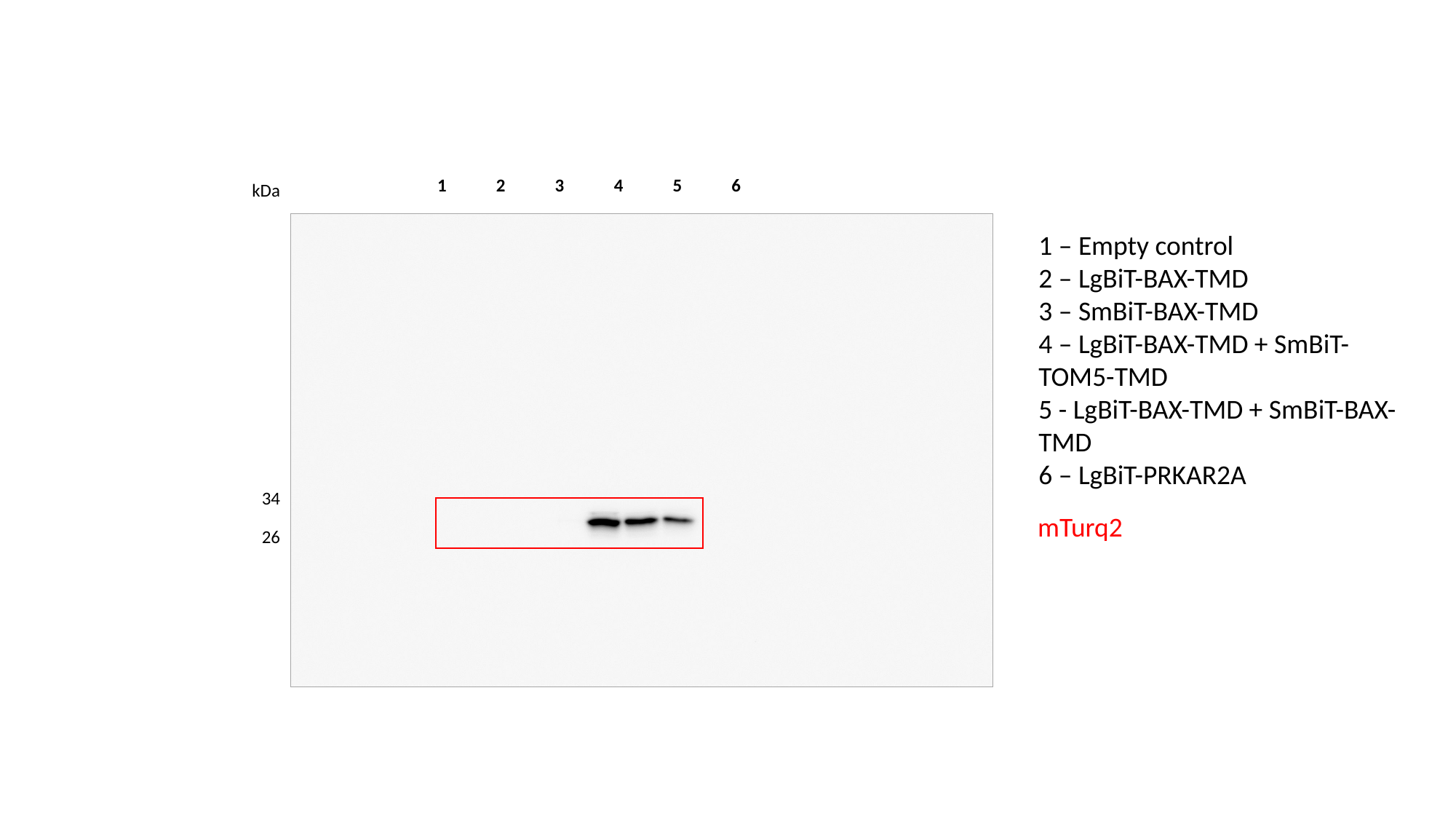

kDa
| 1 | 2 | 3 | 4 | 5 | 6 |
| --- | --- | --- | --- | --- | --- |
1 – Empty control
2 – LgBiT-BAX-TMD
3 – SmBiT-BAX-TMD
4 – LgBiT-BAX-TMD + SmBiT-TOM5-TMD
5 - LgBiT-BAX-TMD + SmBiT-BAX-TMD
6 – LgBiT-PRKAR2A
34
mTurq2
26

## Slide 3
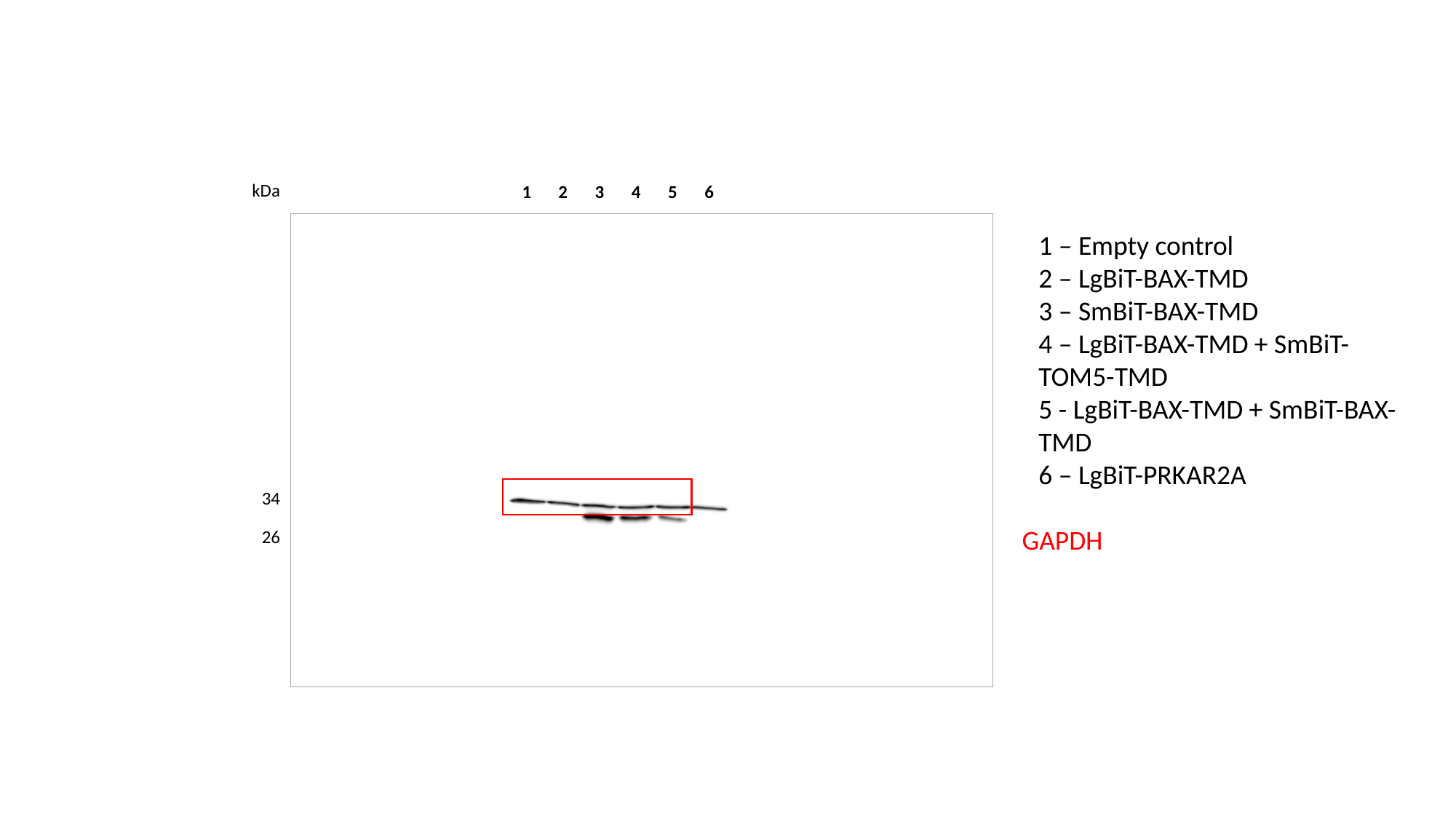

kDa
| 1 | 2 | 3 | 4 | 5 | 6 |
| --- | --- | --- | --- | --- | --- |
1 – Empty control
2 – LgBiT-BAX-TMD
3 – SmBiT-BAX-TMD
4 – LgBiT-BAX-TMD + SmBiT-TOM5-TMD
5 - LgBiT-BAX-TMD + SmBiT-BAX-TMD
6 – LgBiT-PRKAR2A
34
GAPDH
26
