# Supplementary material for: BCL-2 and BOK regulate apoptosis by interaction of their C-terminal transmembrane domains
Source: EMBO Rep. 2024 Jul 24;25(9):12. doi: 10.1038/s44319-024-00206-6 (PMC11387410; doi:10.1038/s44319-024-00206-6)
Supplement: Supplementary file 3 — Source data Fig. 1 [file 44319_2024_206_MOESM3_ESM.zip › Figure 1/1F/1F_western_annotated.pptx]

## Slide 1
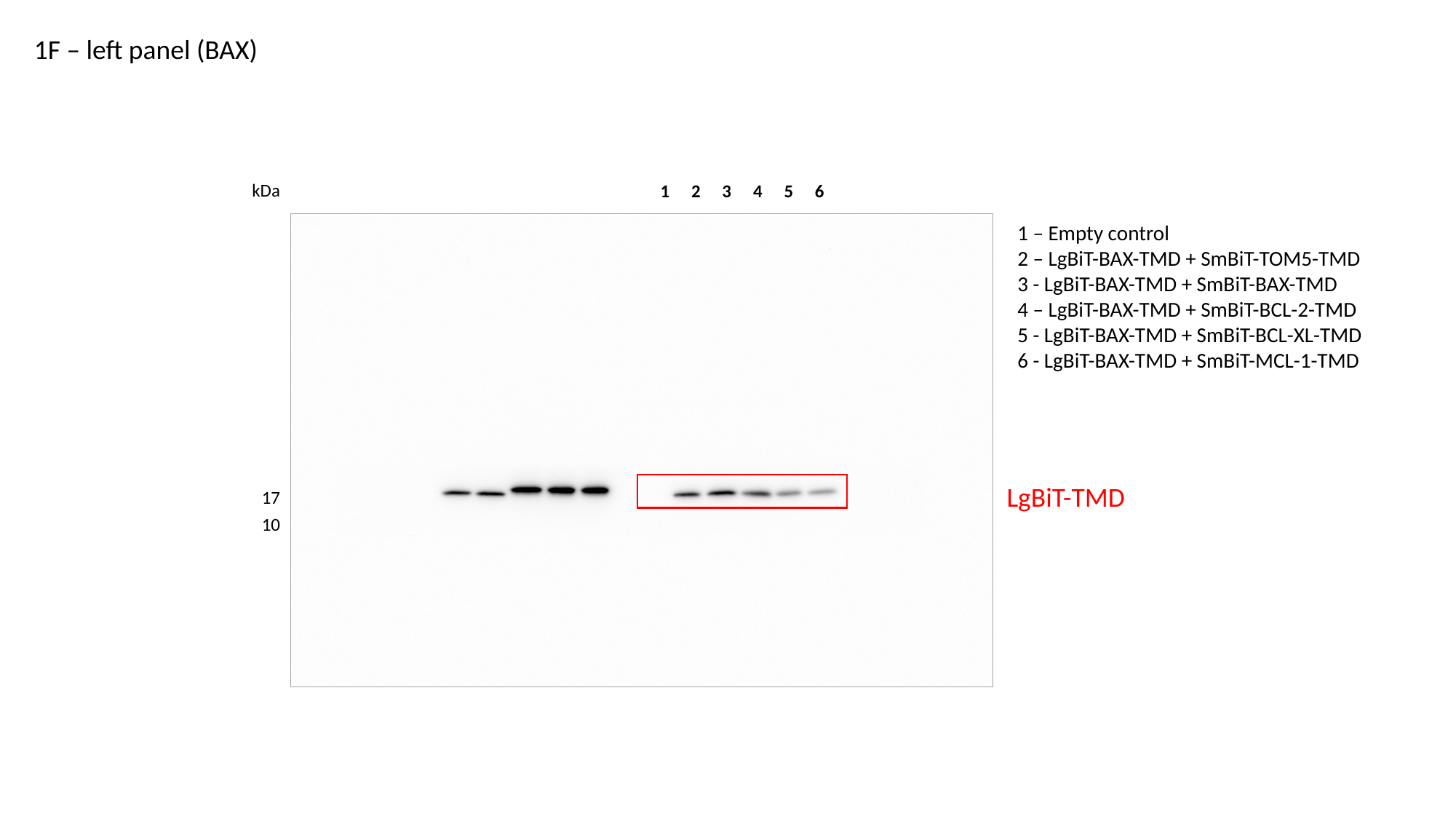

1F – left panel (BAX)
kDa
| 1 | 2 | 3 | 4 | 5 | 6 |
| --- | --- | --- | --- | --- | --- |
1 – Empty control
2 – LgBiT-BAX-TMD + SmBiT-TOM5-TMD
3 - LgBiT-BAX-TMD + SmBiT-BAX-TMD
4 – LgBiT-BAX-TMD + SmBiT-BCL-2-TMD
5 - LgBiT-BAX-TMD + SmBiT-BCL-XL-TMD
6 - LgBiT-BAX-TMD + SmBiT-MCL-1-TMD
LgBiT-TMD
17
10

## Slide 2
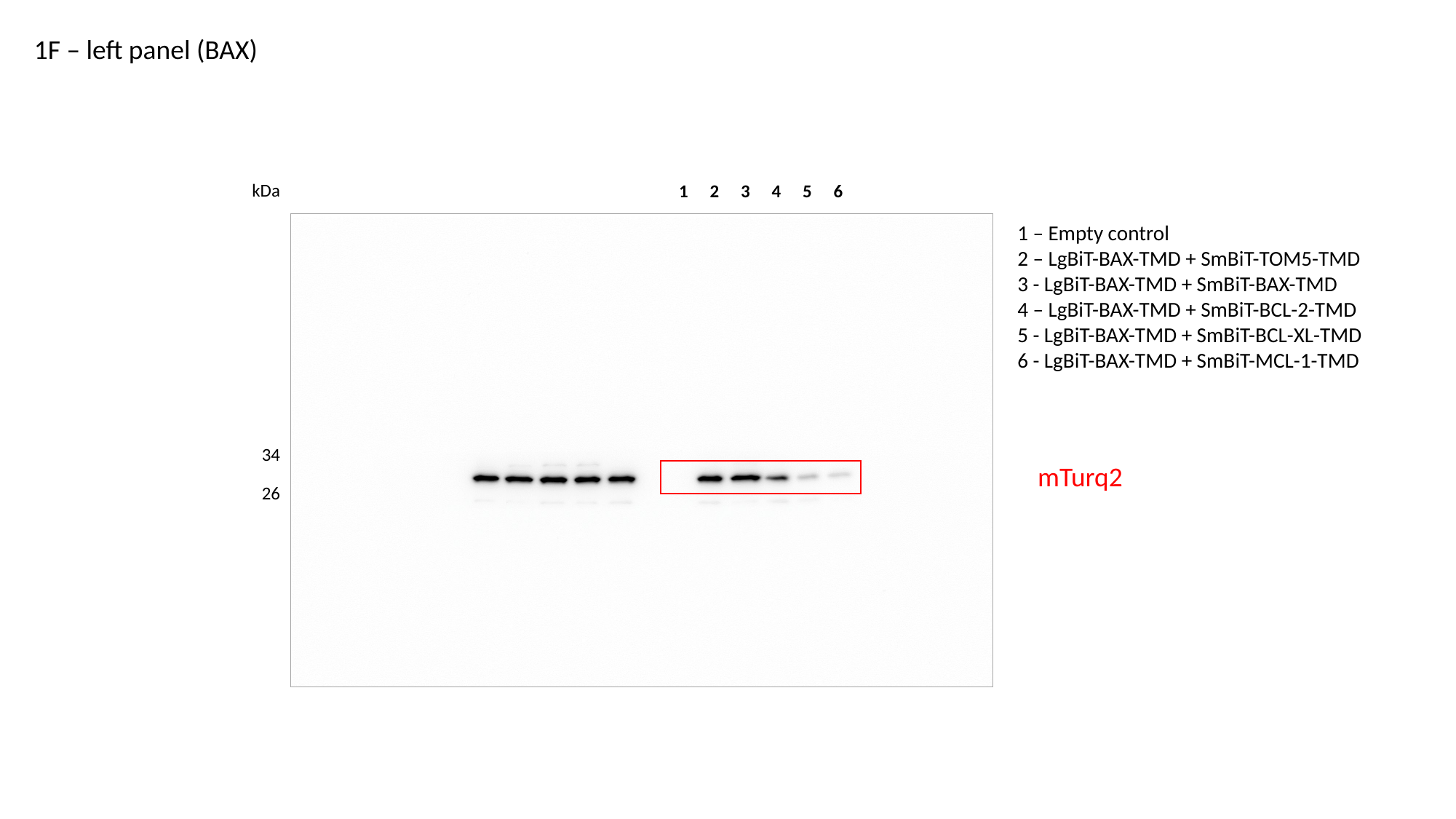

1F – left panel (BAX)
kDa
| 1 | 2 | 3 | 4 | 5 | 6 |
| --- | --- | --- | --- | --- | --- |
1 – Empty control
2 – LgBiT-BAX-TMD + SmBiT-TOM5-TMD
3 - LgBiT-BAX-TMD + SmBiT-BAX-TMD
4 – LgBiT-BAX-TMD + SmBiT-BCL-2-TMD
5 - LgBiT-BAX-TMD + SmBiT-BCL-XL-TMD
6 - LgBiT-BAX-TMD + SmBiT-MCL-1-TMD
34
mTurq2
26

## Slide 3
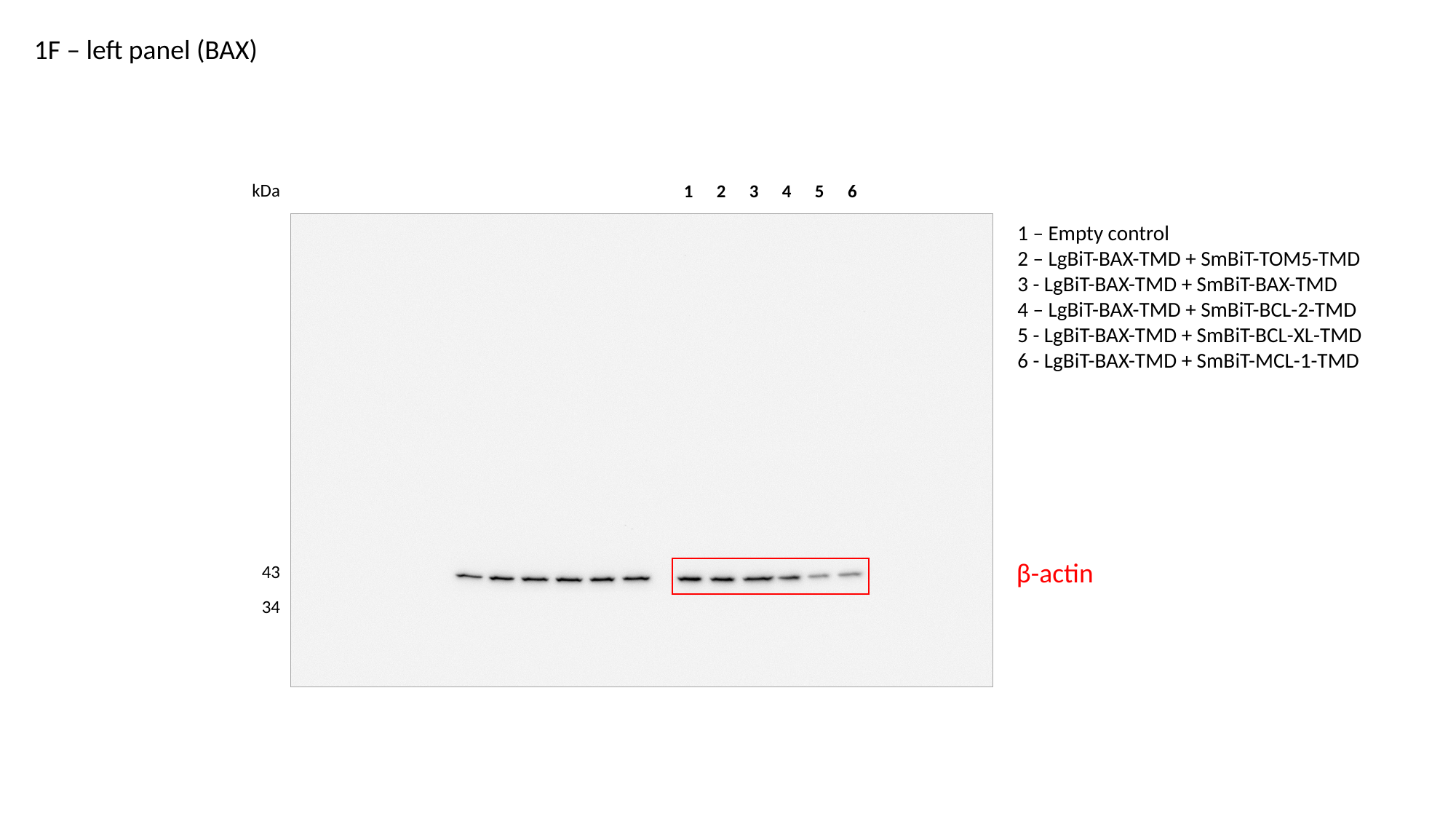

1F – left panel (BAX)
kDa
| 1 | 2 | 3 | 4 | 5 | 6 |
| --- | --- | --- | --- | --- | --- |
1 – Empty control
2 – LgBiT-BAX-TMD + SmBiT-TOM5-TMD
3 - LgBiT-BAX-TMD + SmBiT-BAX-TMD
4 – LgBiT-BAX-TMD + SmBiT-BCL-2-TMD
5 - LgBiT-BAX-TMD + SmBiT-BCL-XL-TMD
6 - LgBiT-BAX-TMD + SmBiT-MCL-1-TMD
β-actin
43
34

## Slide 4
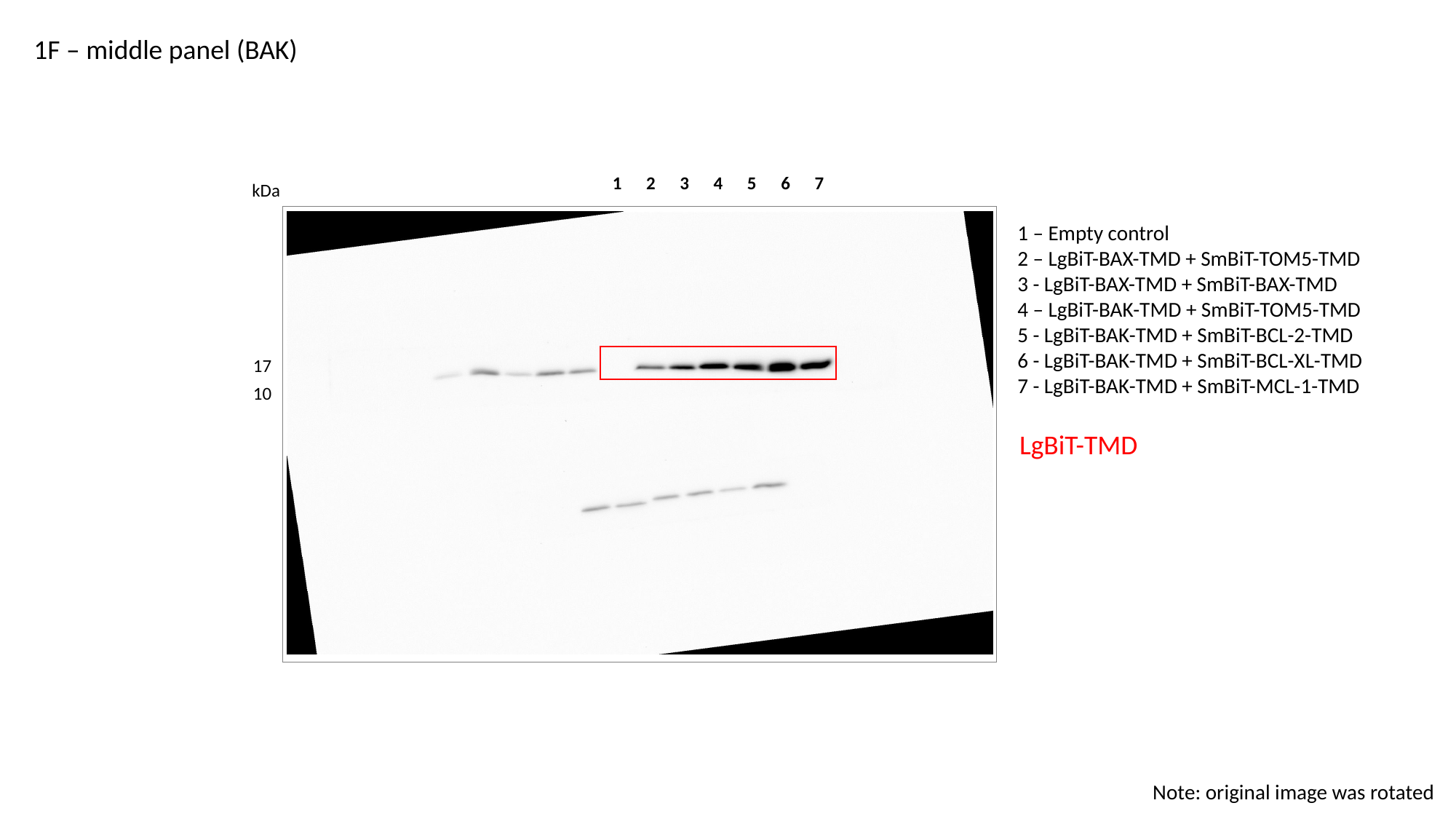

1F – middle panel (BAK)
| 1 | 2 | 3 | 4 | 5 | 6 | 7 |
| --- | --- | --- | --- | --- | --- | --- |
kDa
1 – Empty control
2 – LgBiT-BAX-TMD + SmBiT-TOM5-TMD
3 - LgBiT-BAX-TMD + SmBiT-BAX-TMD
4 – LgBiT-BAK-TMD + SmBiT-TOM5-TMD
5 - LgBiT-BAK-TMD + SmBiT-BCL-2-TMD
6 - LgBiT-BAK-TMD + SmBiT-BCL-XL-TMD
7 - LgBiT-BAK-TMD + SmBiT-MCL-1-TMD
17
10
LgBiT-TMD
Note: original image was rotated

## Slide 5
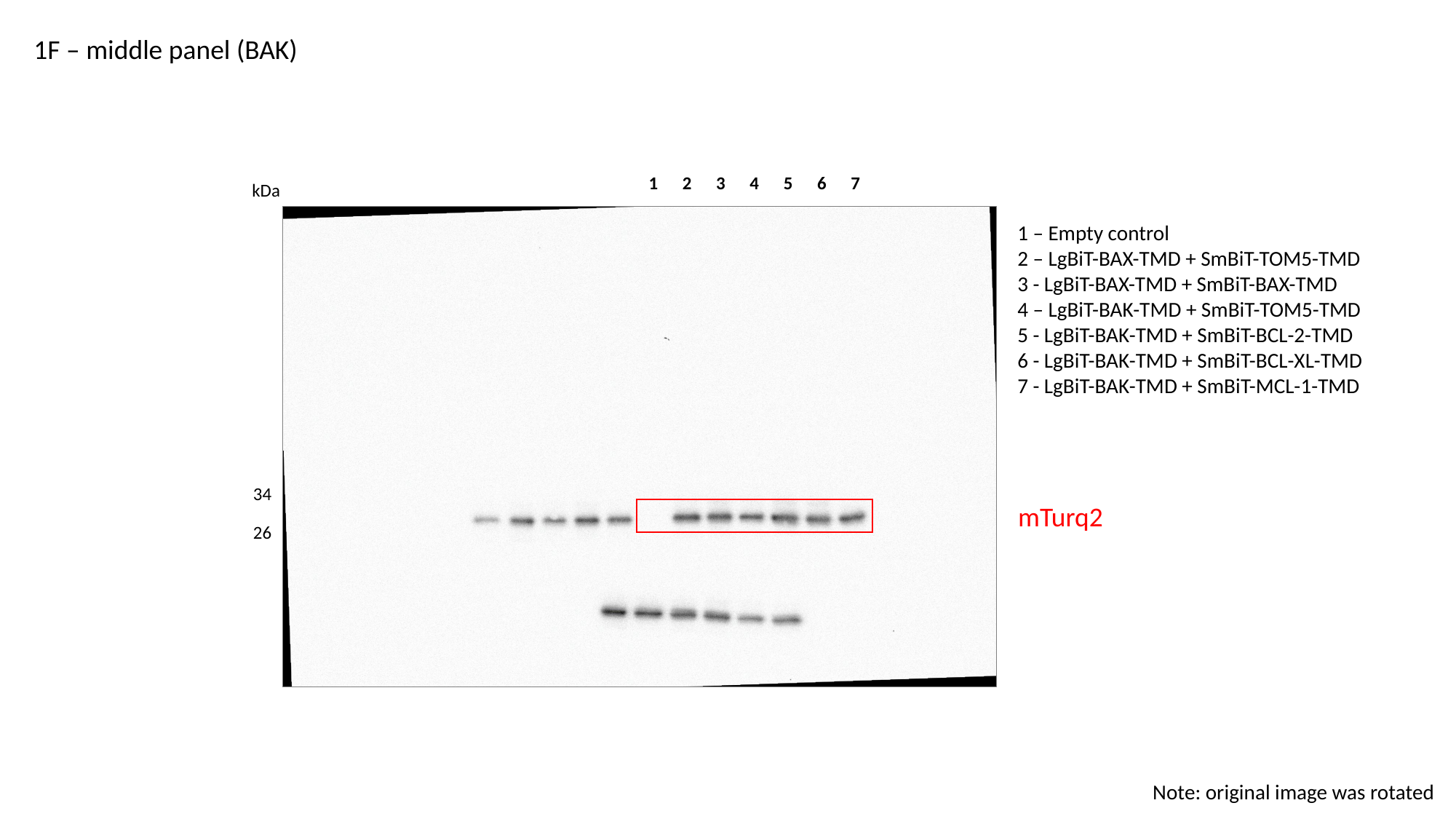

1F – middle panel (BAK)
| 1 | 2 | 3 | 4 | 5 | 6 | 7 |
| --- | --- | --- | --- | --- | --- | --- |
kDa
1 – Empty control
2 – LgBiT-BAX-TMD + SmBiT-TOM5-TMD
3 - LgBiT-BAX-TMD + SmBiT-BAX-TMD
4 – LgBiT-BAK-TMD + SmBiT-TOM5-TMD
5 - LgBiT-BAK-TMD + SmBiT-BCL-2-TMD
6 - LgBiT-BAK-TMD + SmBiT-BCL-XL-TMD
7 - LgBiT-BAK-TMD + SmBiT-MCL-1-TMD
34
mTurq2
26
Note: original image was rotated

## Slide 6
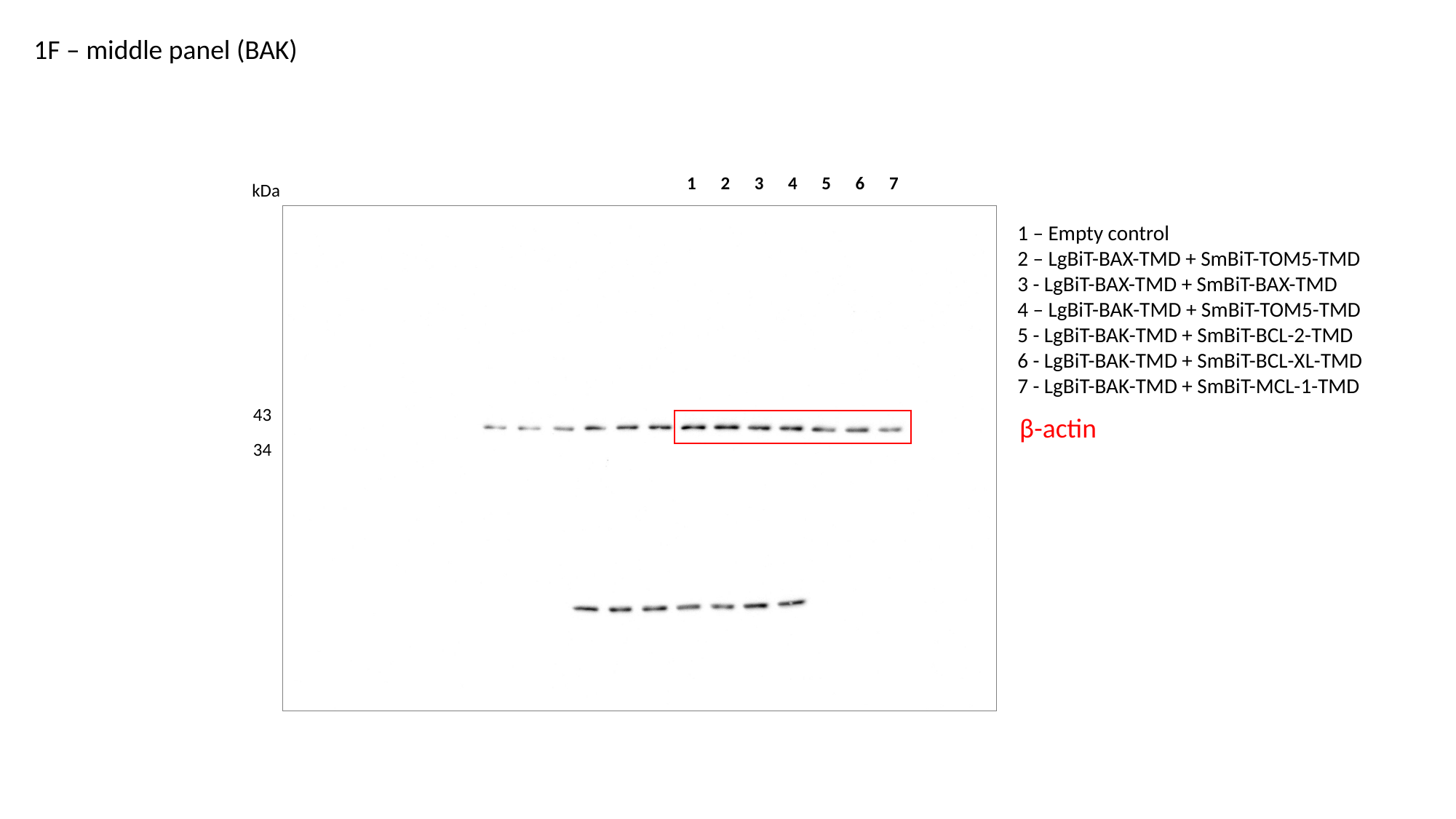

1F – middle panel (BAK)
| 1 | 2 | 3 | 4 | 5 | 6 | 7 |
| --- | --- | --- | --- | --- | --- | --- |
kDa
1 – Empty control
2 – LgBiT-BAX-TMD + SmBiT-TOM5-TMD
3 - LgBiT-BAX-TMD + SmBiT-BAX-TMD
4 – LgBiT-BAK-TMD + SmBiT-TOM5-TMD
5 - LgBiT-BAK-TMD + SmBiT-BCL-2-TMD
6 - LgBiT-BAK-TMD + SmBiT-BCL-XL-TMD
7 - LgBiT-BAK-TMD + SmBiT-MCL-1-TMD
43
β-actin
34

## Slide 7
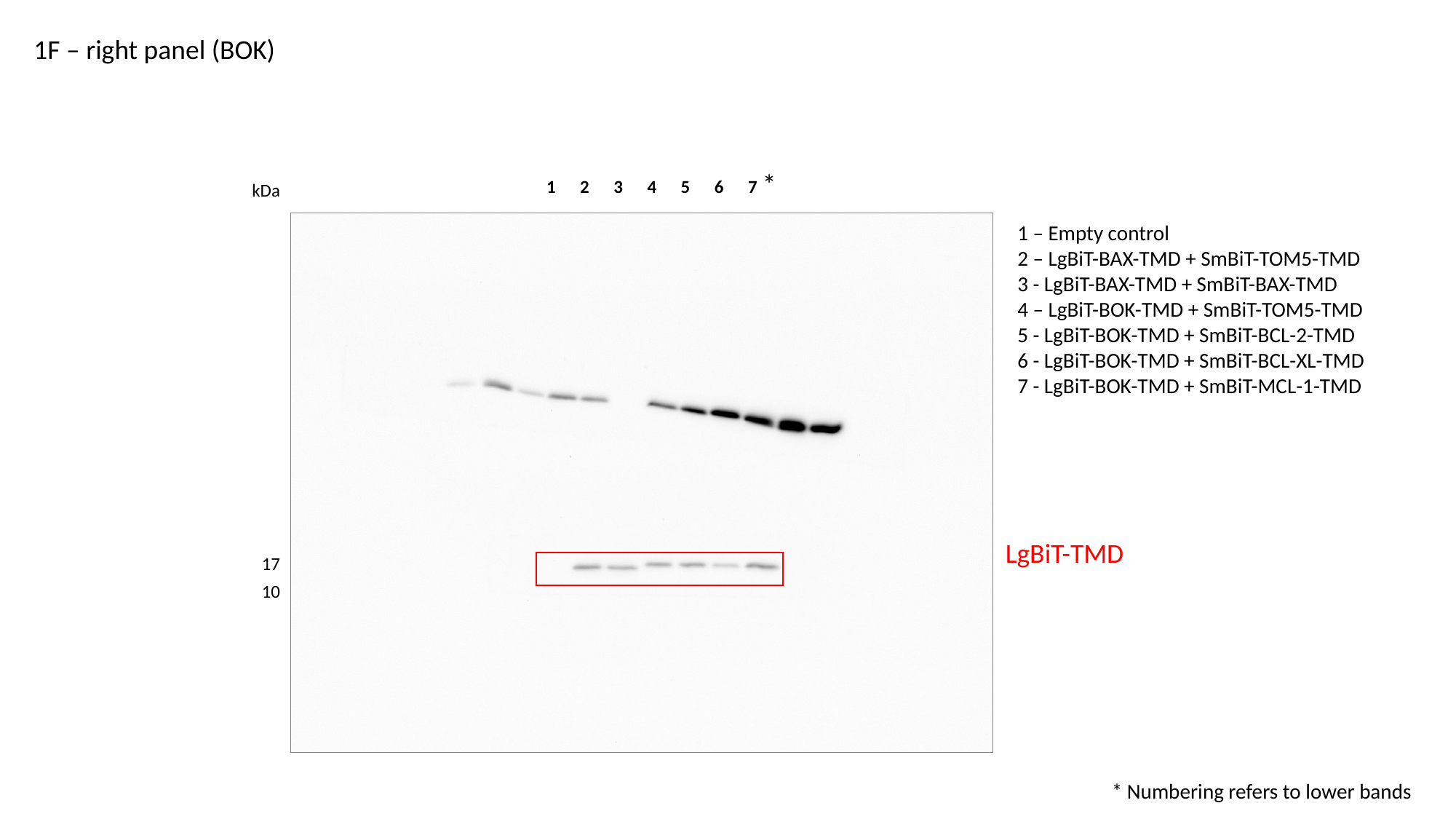

1F – right panel (BOK)
*
kDa
| 1 | 2 | 3 | 4 | 5 | 6 | 7 |
| --- | --- | --- | --- | --- | --- | --- |
1 – Empty control
2 – LgBiT-BAX-TMD + SmBiT-TOM5-TMD
3 - LgBiT-BAX-TMD + SmBiT-BAX-TMD
4 – LgBiT-BOK-TMD + SmBiT-TOM5-TMD
5 - LgBiT-BOK-TMD + SmBiT-BCL-2-TMD
6 - LgBiT-BOK-TMD + SmBiT-BCL-XL-TMD
7 - LgBiT-BOK-TMD + SmBiT-MCL-1-TMD
LgBiT-TMD
17
10
* Numbering refers to lower bands

## Slide 8
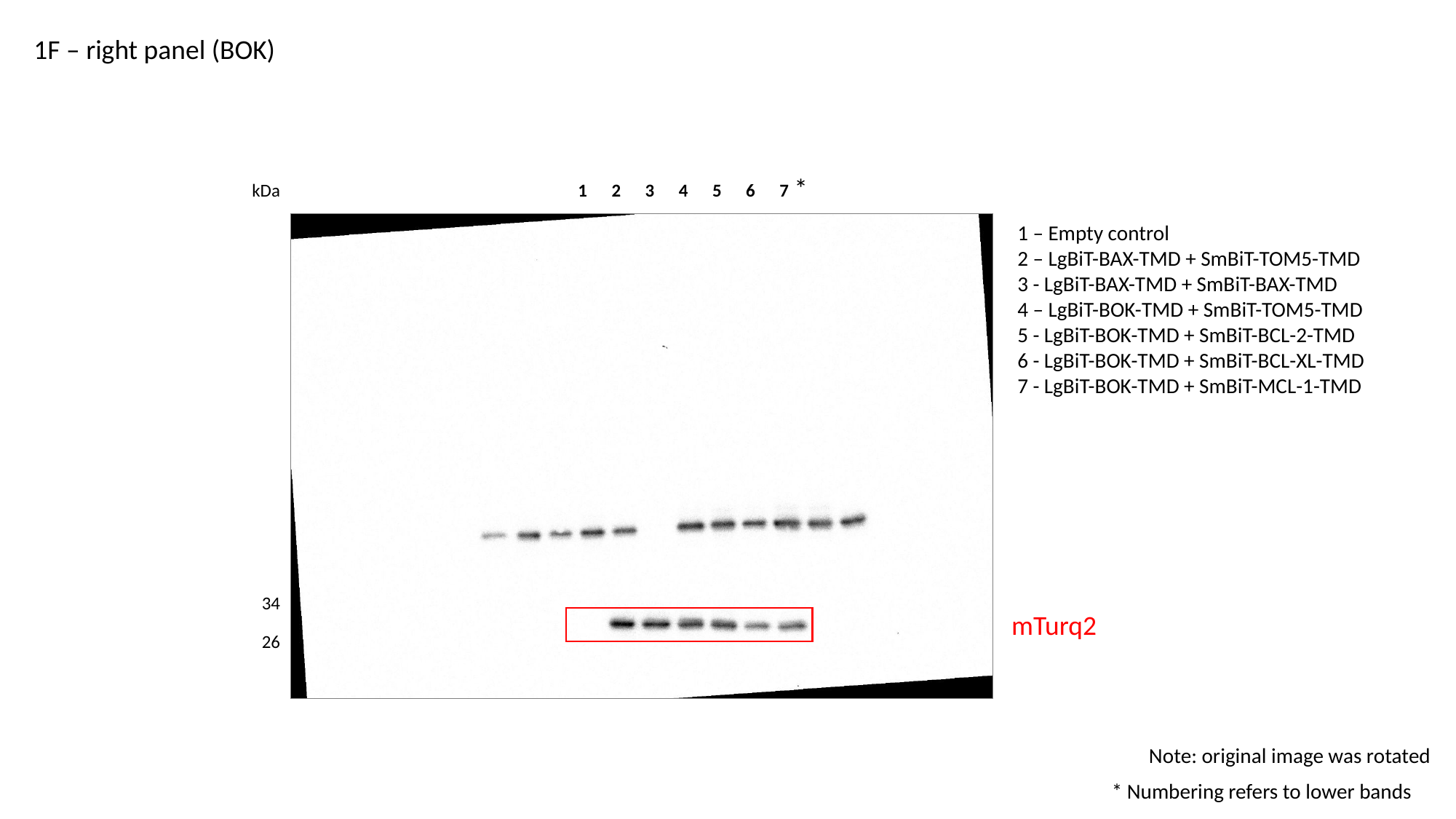

1F – right panel (BOK)
*
kDa
| 1 | 2 | 3 | 4 | 5 | 6 | 7 |
| --- | --- | --- | --- | --- | --- | --- |
1 – Empty control
2 – LgBiT-BAX-TMD + SmBiT-TOM5-TMD
3 - LgBiT-BAX-TMD + SmBiT-BAX-TMD
4 – LgBiT-BOK-TMD + SmBiT-TOM5-TMD
5 - LgBiT-BOK-TMD + SmBiT-BCL-2-TMD
6 - LgBiT-BOK-TMD + SmBiT-BCL-XL-TMD
7 - LgBiT-BOK-TMD + SmBiT-MCL-1-TMD
34
mTurq2
26
Note: original image was rotated
* Numbering refers to lower bands

## Slide 9
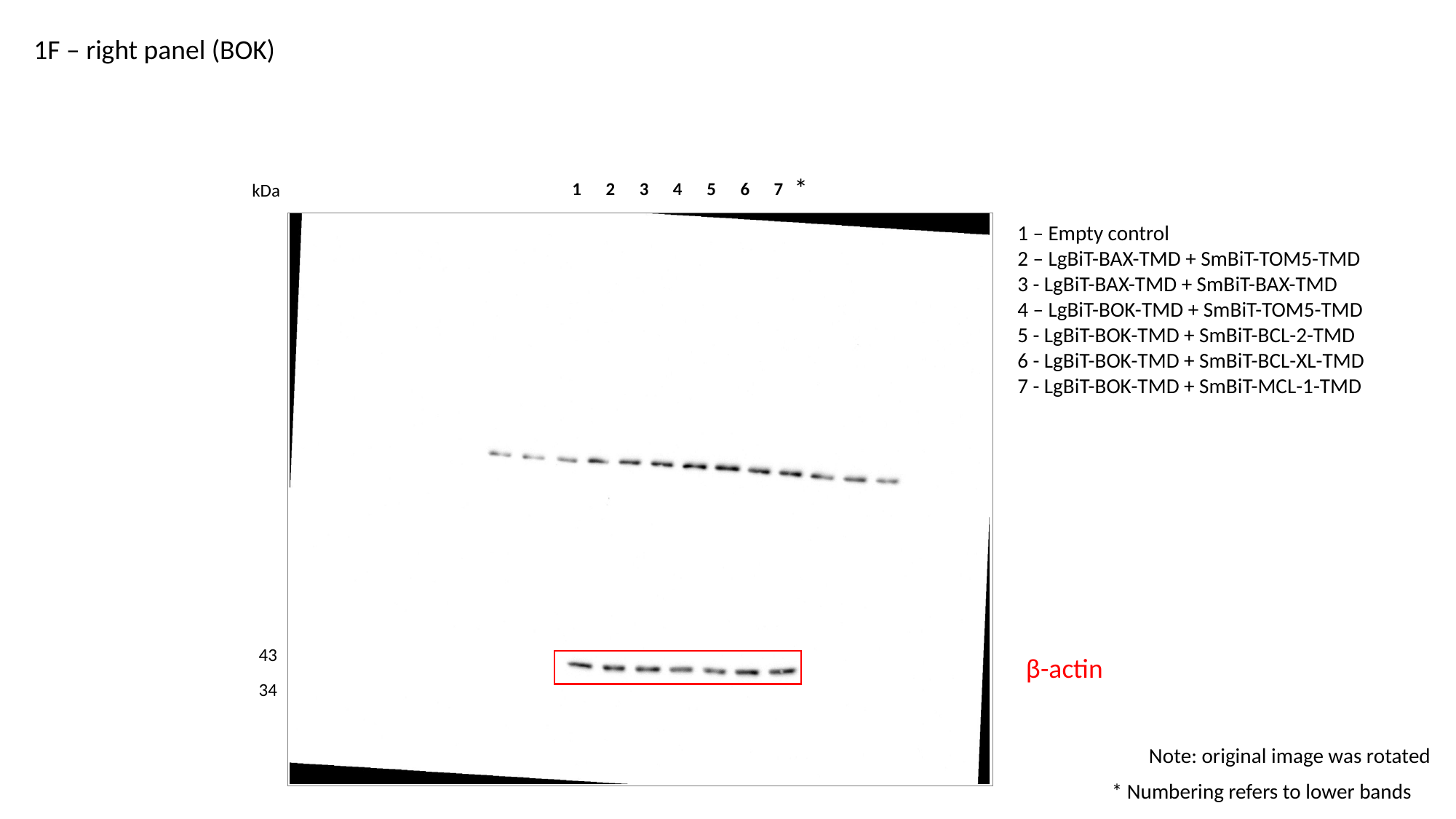

1F – right panel (BOK)
*
kDa
| 1 | 2 | 3 | 4 | 5 | 6 | 7 |
| --- | --- | --- | --- | --- | --- | --- |
1 – Empty control
2 – LgBiT-BAX-TMD + SmBiT-TOM5-TMD
3 - LgBiT-BAX-TMD + SmBiT-BAX-TMD
4 – LgBiT-BOK-TMD + SmBiT-TOM5-TMD
5 - LgBiT-BOK-TMD + SmBiT-BCL-2-TMD
6 - LgBiT-BOK-TMD + SmBiT-BCL-XL-TMD
7 - LgBiT-BOK-TMD + SmBiT-MCL-1-TMD
43
β-actin
34
Note: original image was rotated
* Numbering refers to lower bands
