# Supplementary figures and images for: BCL-2 and BOK regulate apoptosis by interaction of their C-terminal transmembrane domains (part 1 of 2)
Source: EMBO Rep. 2024 Jul 24;25(9):12. doi: 10.1038/s44319-024-00206-6 (PMC11387410; doi:10.1038/s44319-024-00206-6)

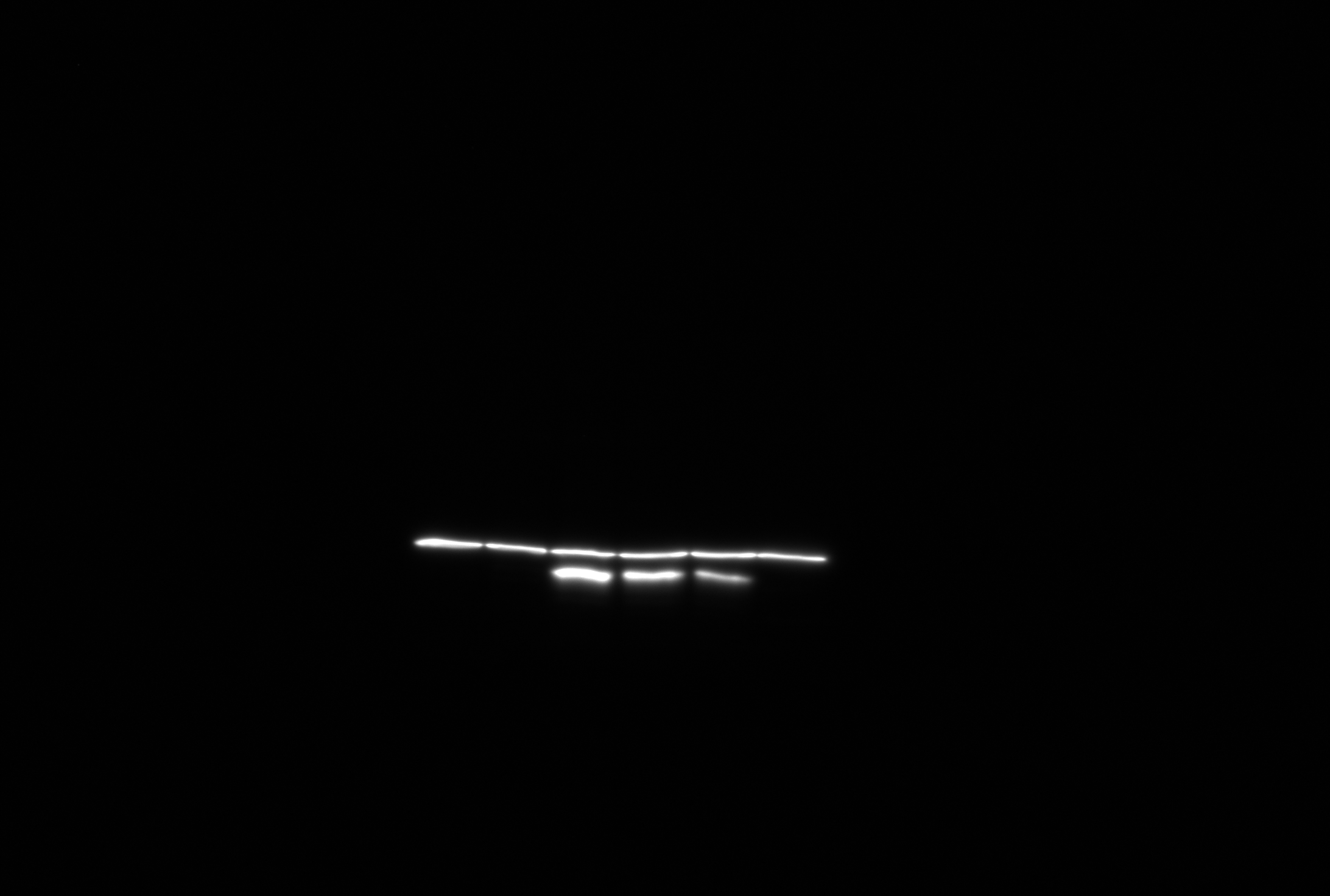

Supplement: Supplementary file 3 — Source data Fig. 1 [file 44319_2024_206_MOESM3_ESM.zip › Figure 1/1C/1C_GAPDH_western.tif]

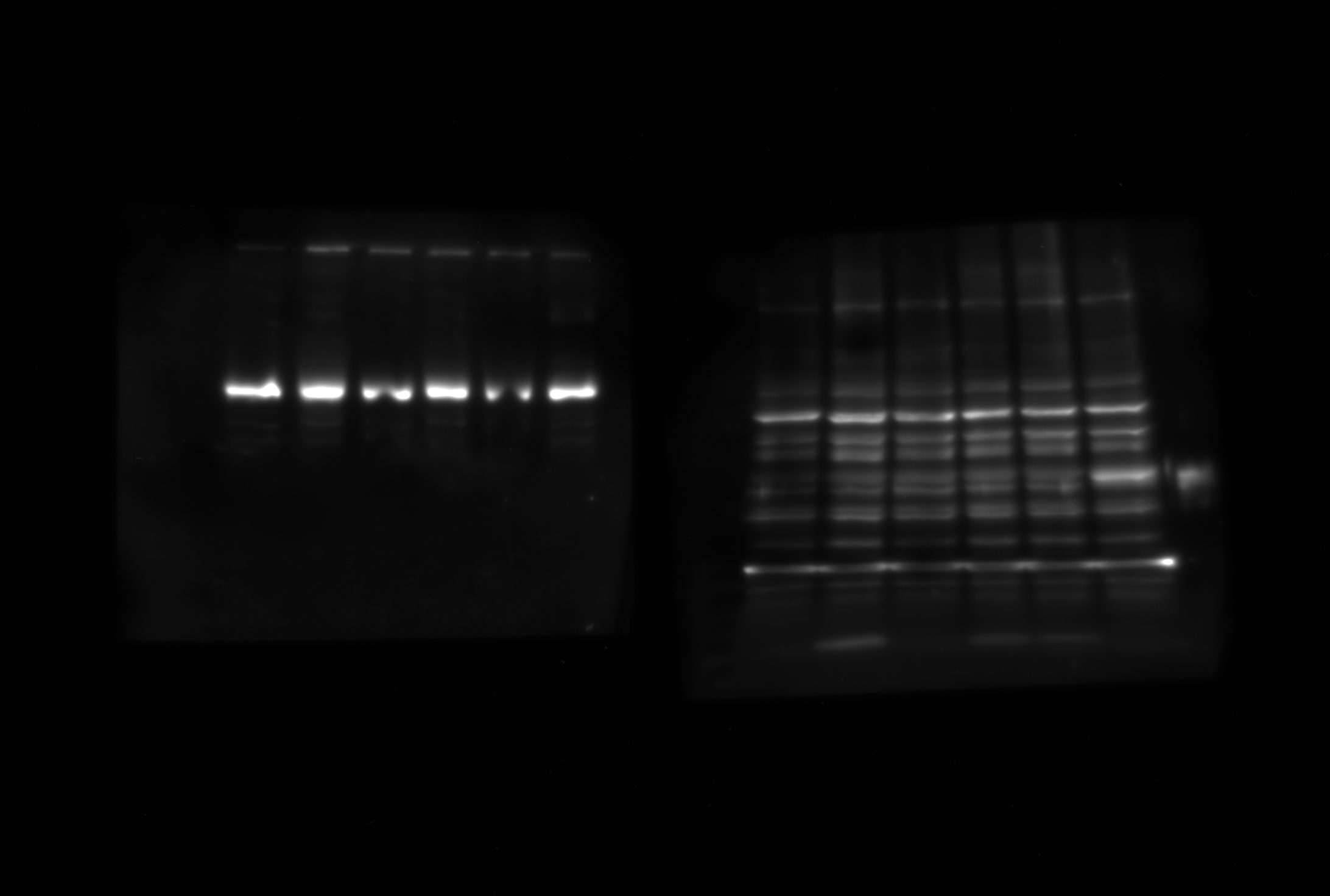

Supplement: Supplementary file 3 — Source data Fig. 1 [file 44319_2024_206_MOESM3_ESM.zip › Figure 1/1C/1C_LgBiT_western.tif]

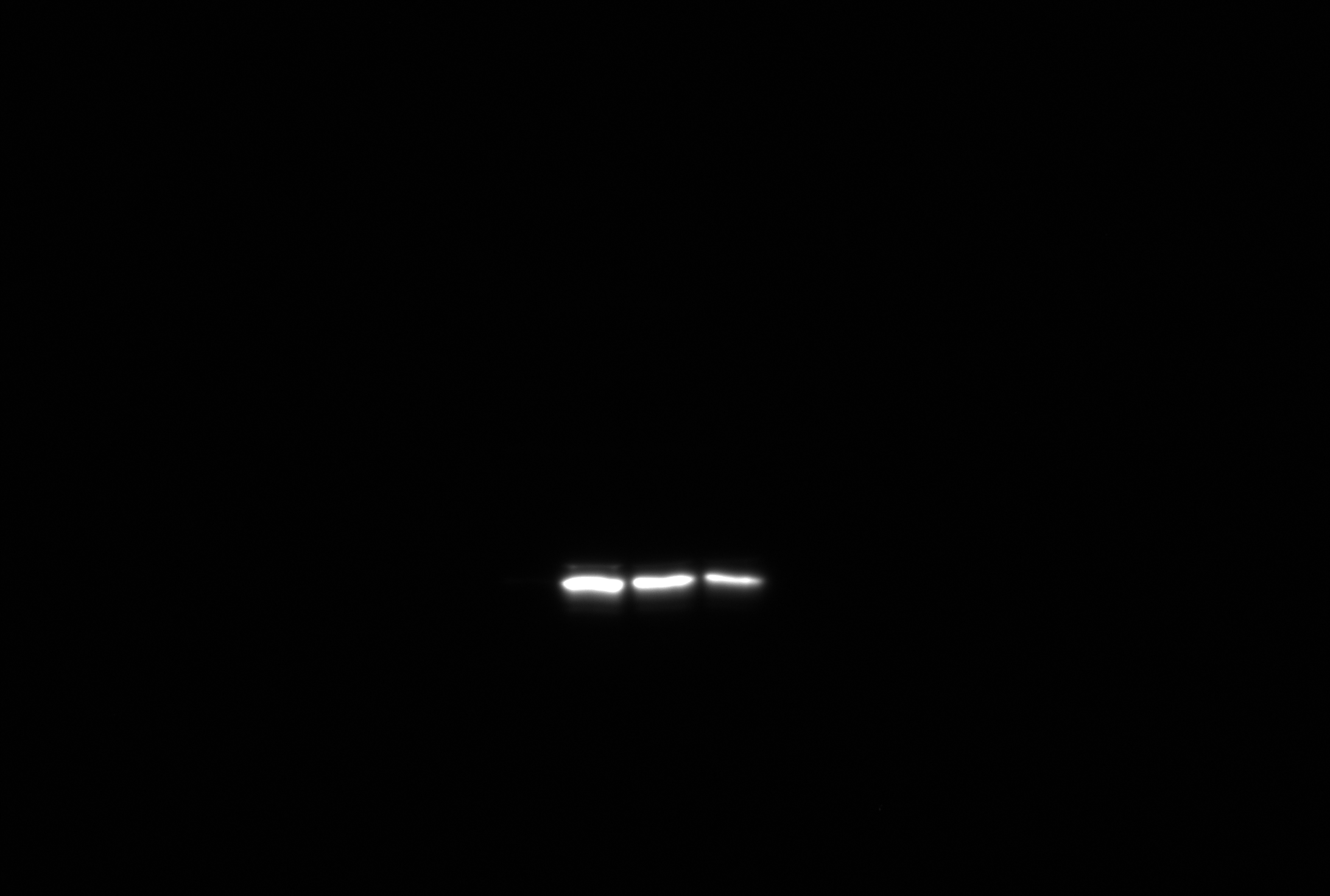

Supplement: Supplementary file 3 — Source data Fig. 1 [file 44319_2024_206_MOESM3_ESM.zip › Figure 1/1C/1C_mTurq2_western.tif]

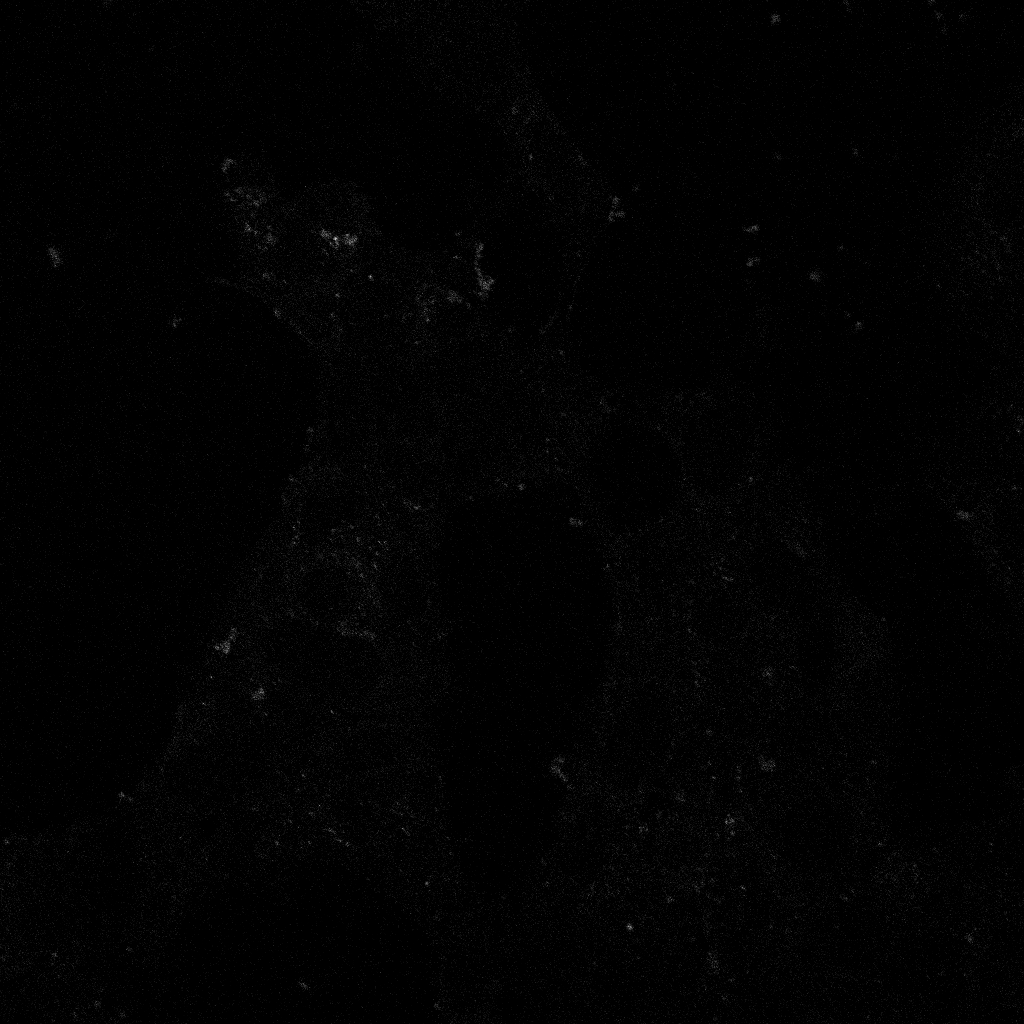

Supplement: Supplementary file 3 — Source data Fig. 1 [file 44319_2024_206_MOESM3_ESM.zip › Figure 1/1D/LgBiT-BAX-TMD/1D_LgBiTBAX_405nm.tif]

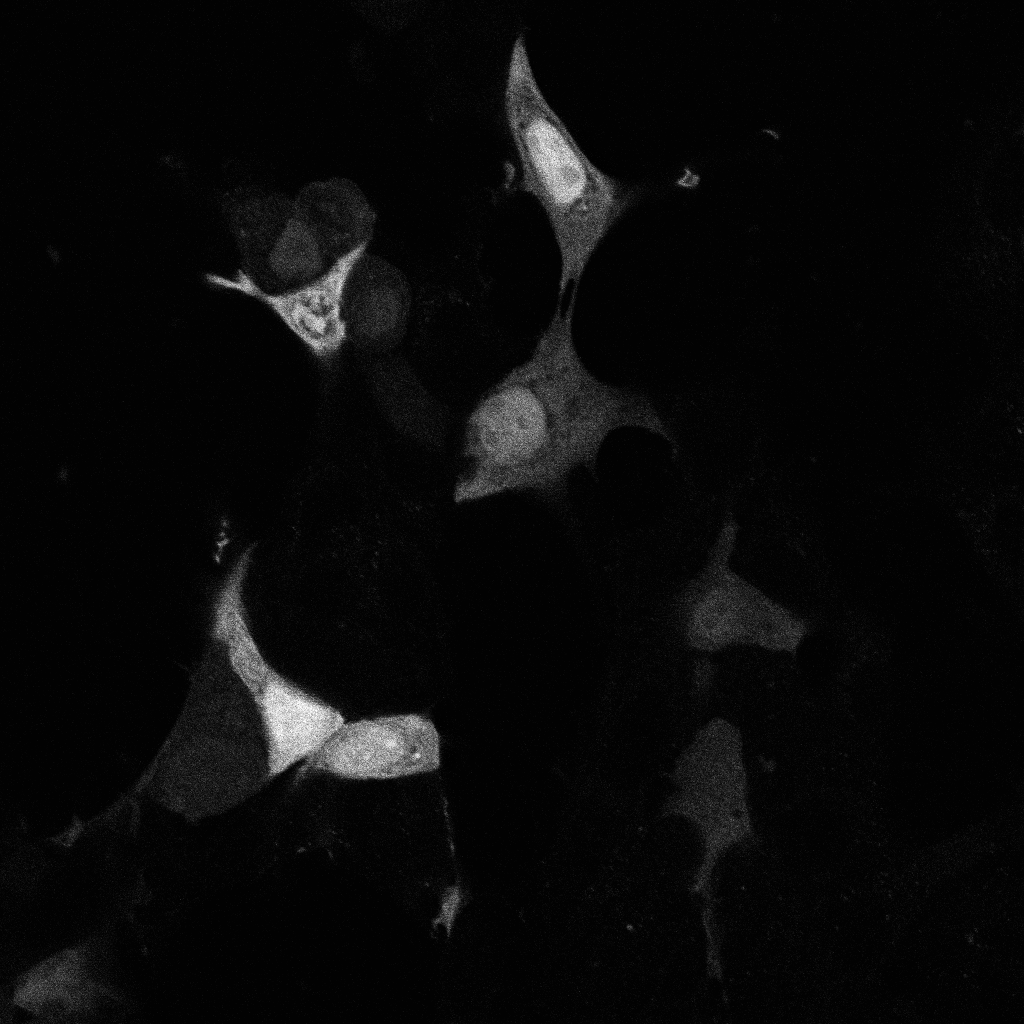

Supplement: Supplementary file 3 — Source data Fig. 1 [file 44319_2024_206_MOESM3_ESM.zip › Figure 1/1D/LgBiT-BAX-TMD/1D_LgBiTBAX_488nm.tif]

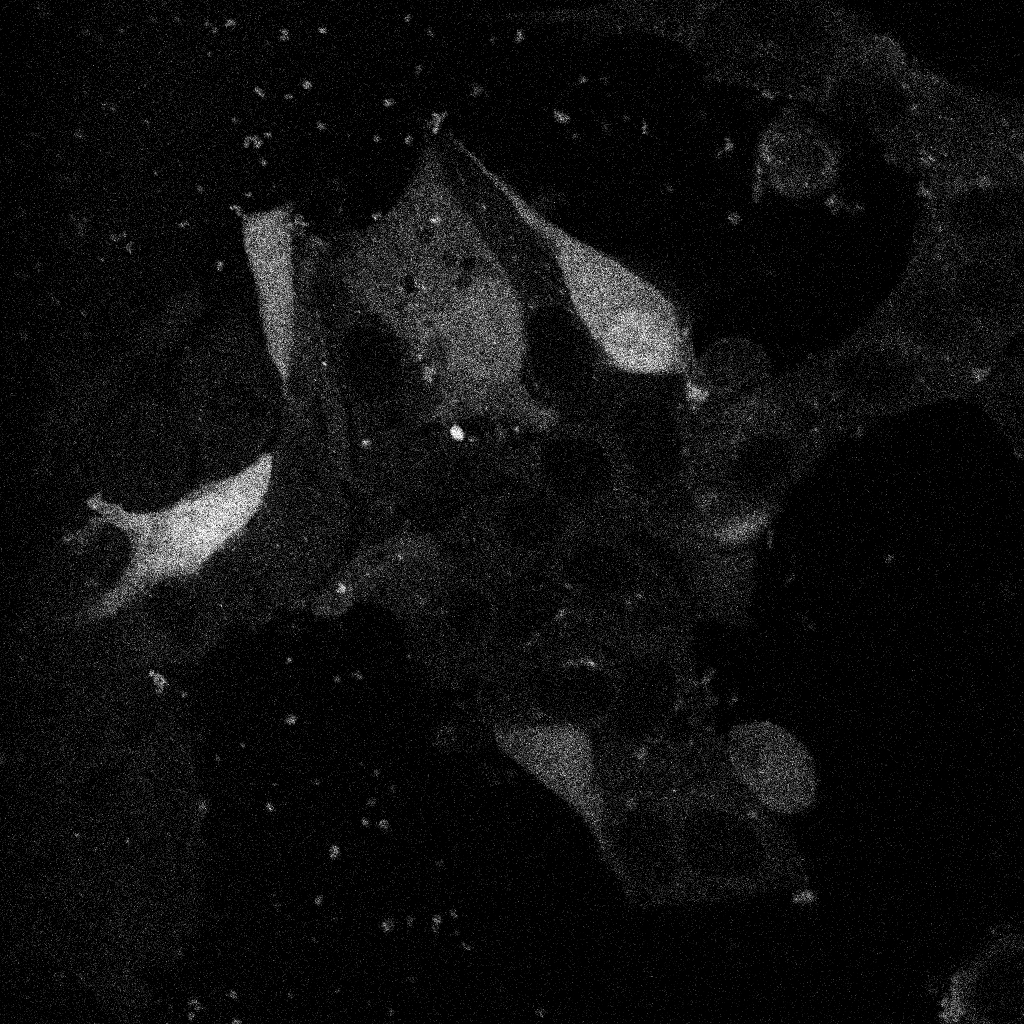

Supplement: Supplementary file 3 — Source data Fig. 1 [file 44319_2024_206_MOESM3_ESM.zip › Figure 1/1D/LgBiT-BAX-TMD+SmBiT-BAX-TMD/1D_LgBiTSmBiTBAX_405nm.tif]

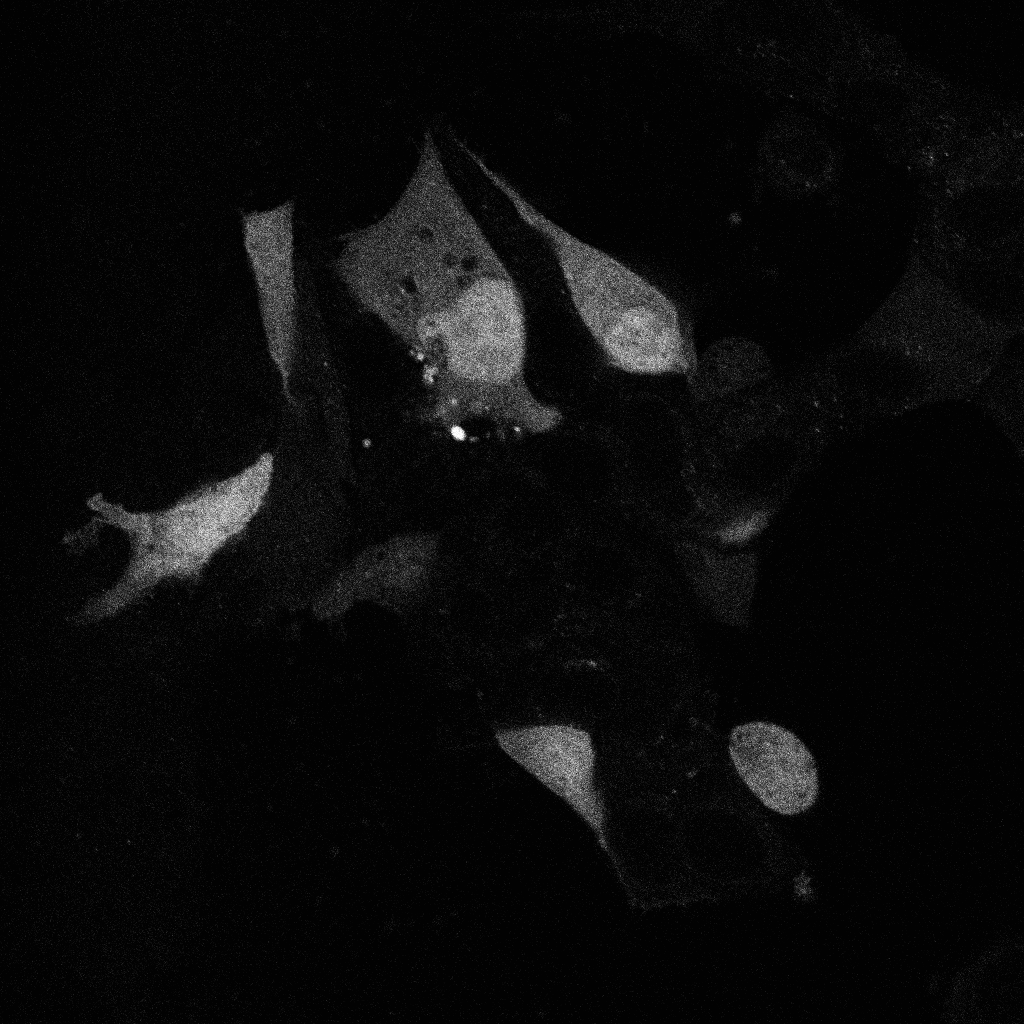

Supplement: Supplementary file 3 — Source data Fig. 1 [file 44319_2024_206_MOESM3_ESM.zip › Figure 1/1D/LgBiT-BAX-TMD+SmBiT-BAX-TMD/1D_LgBiTSmBiTBAX_488nm.tif]

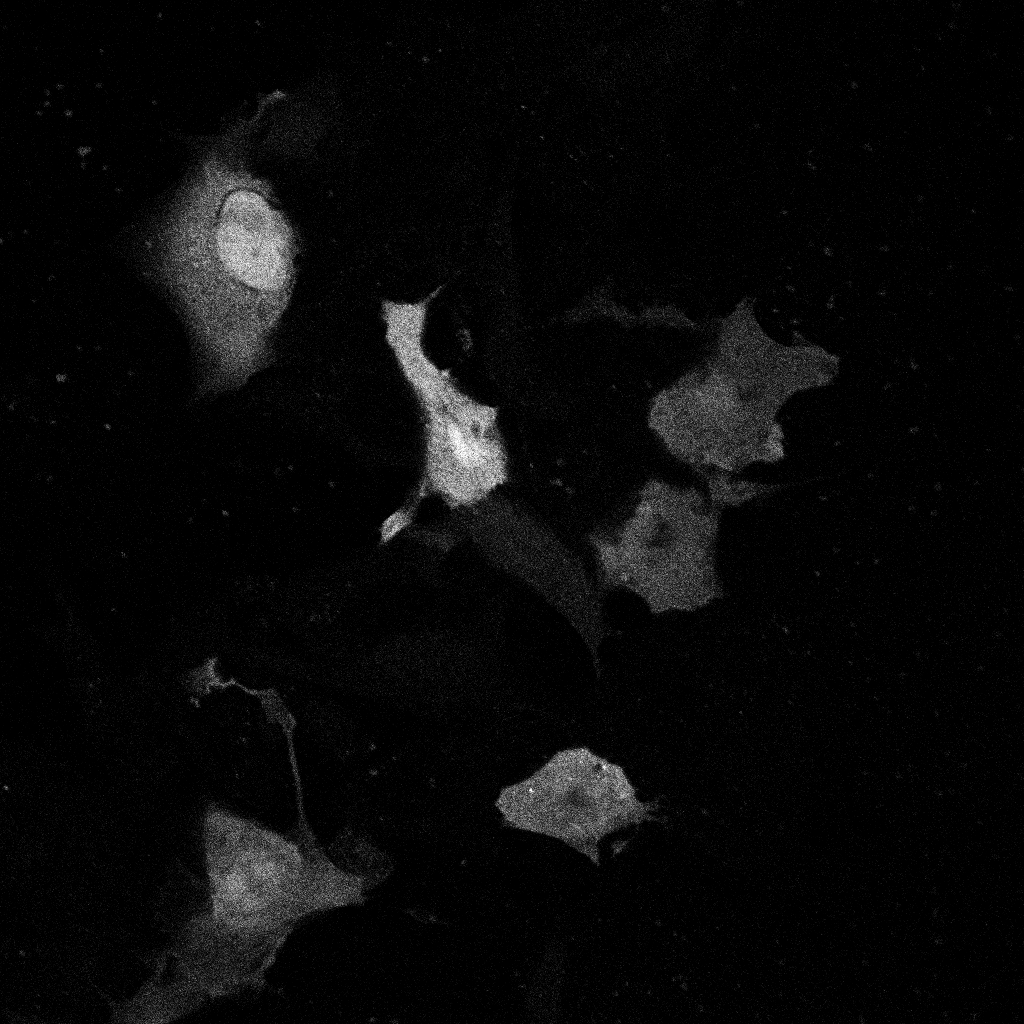

Supplement: Supplementary file 3 — Source data Fig. 1 [file 44319_2024_206_MOESM3_ESM.zip › Figure 1/1D/SmBiT-BAX-TMD/1D_SmBiTBAXTMD_405nm.tif]

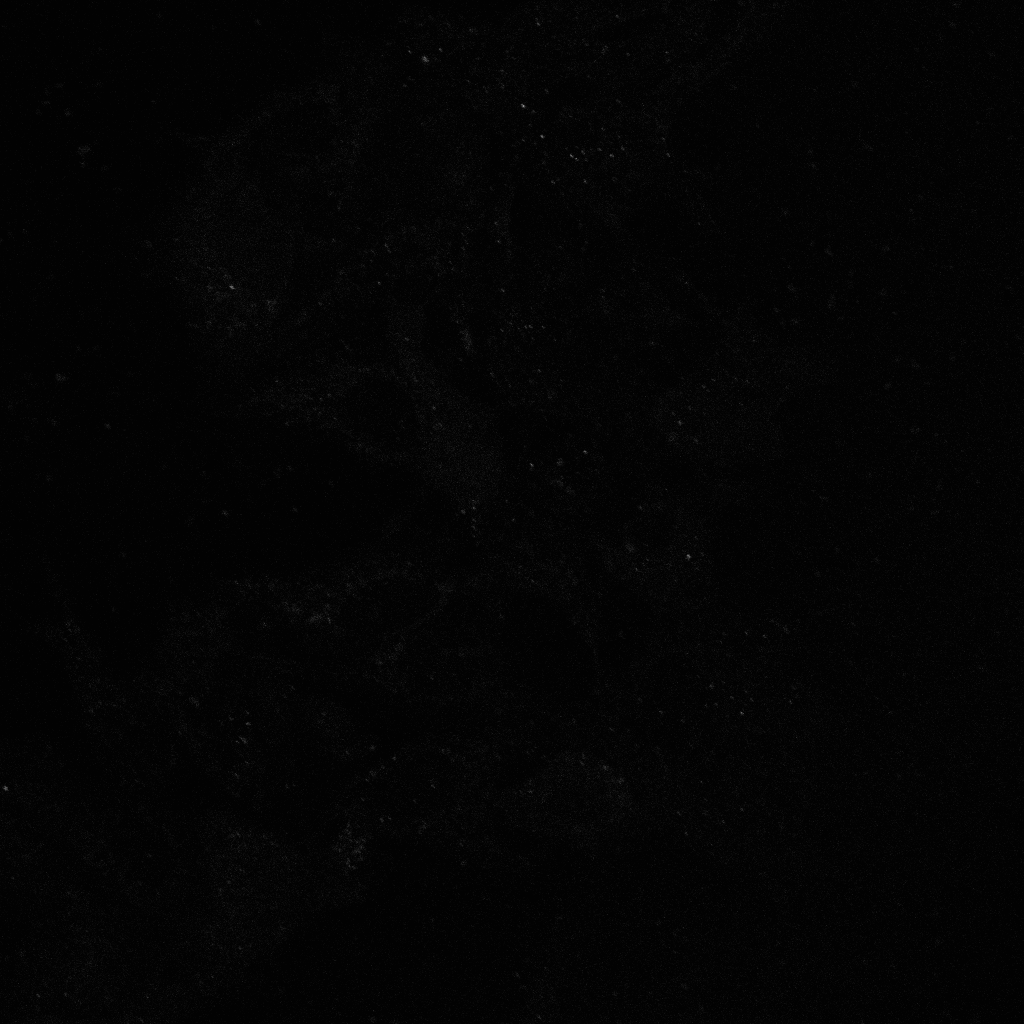

Supplement: Supplementary file 3 — Source data Fig. 1 [file 44319_2024_206_MOESM3_ESM.zip › Figure 1/1D/SmBiT-BAX-TMD/1D_SmBiTBAXTMD_488nm.tif]

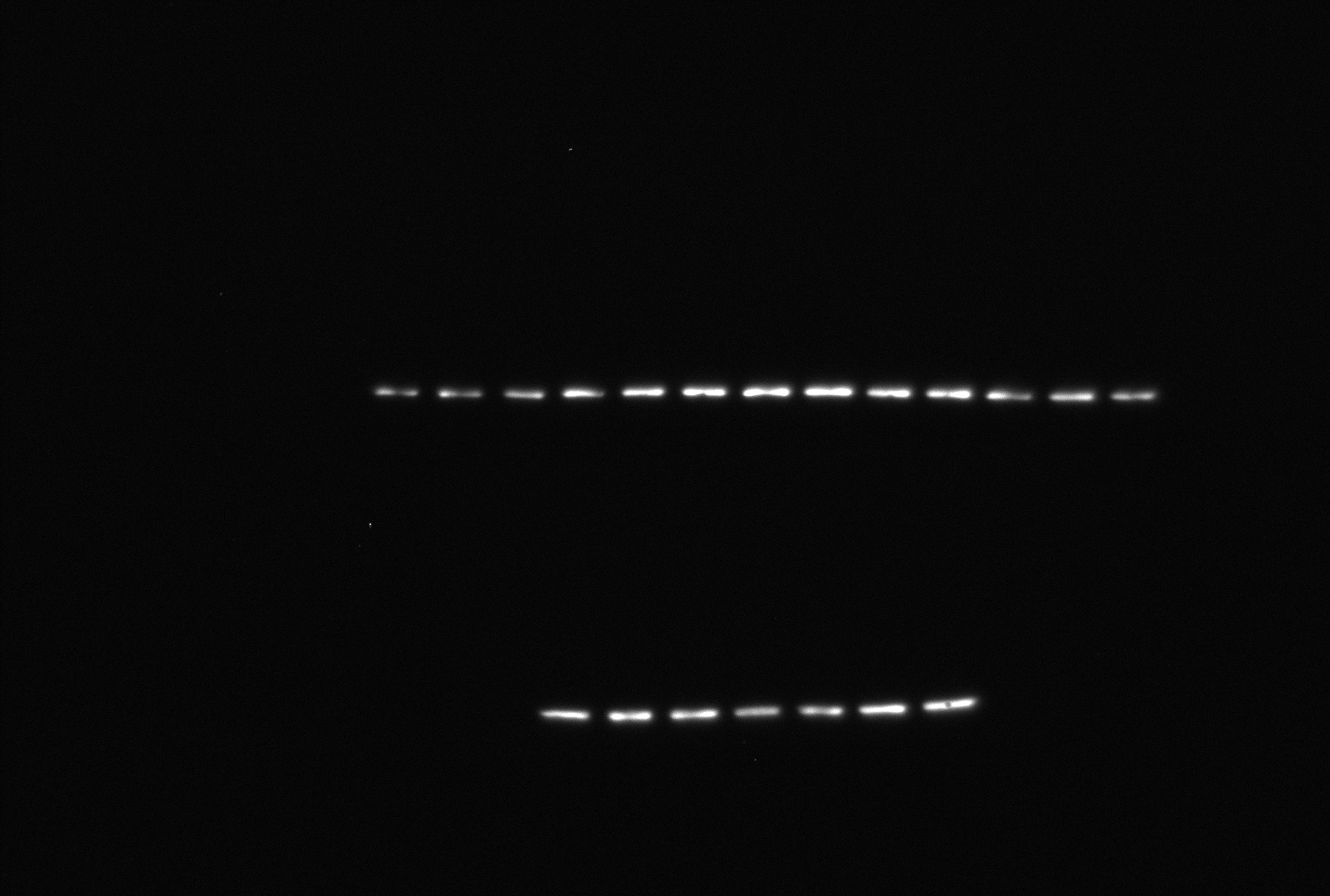

Supplement: Supplementary file 3 — Source data Fig. 1 [file 44319_2024_206_MOESM3_ESM.zip › Figure 1/1F/1F_western_BAKTMD_BOKTMD/1F_western_bActin.tif]

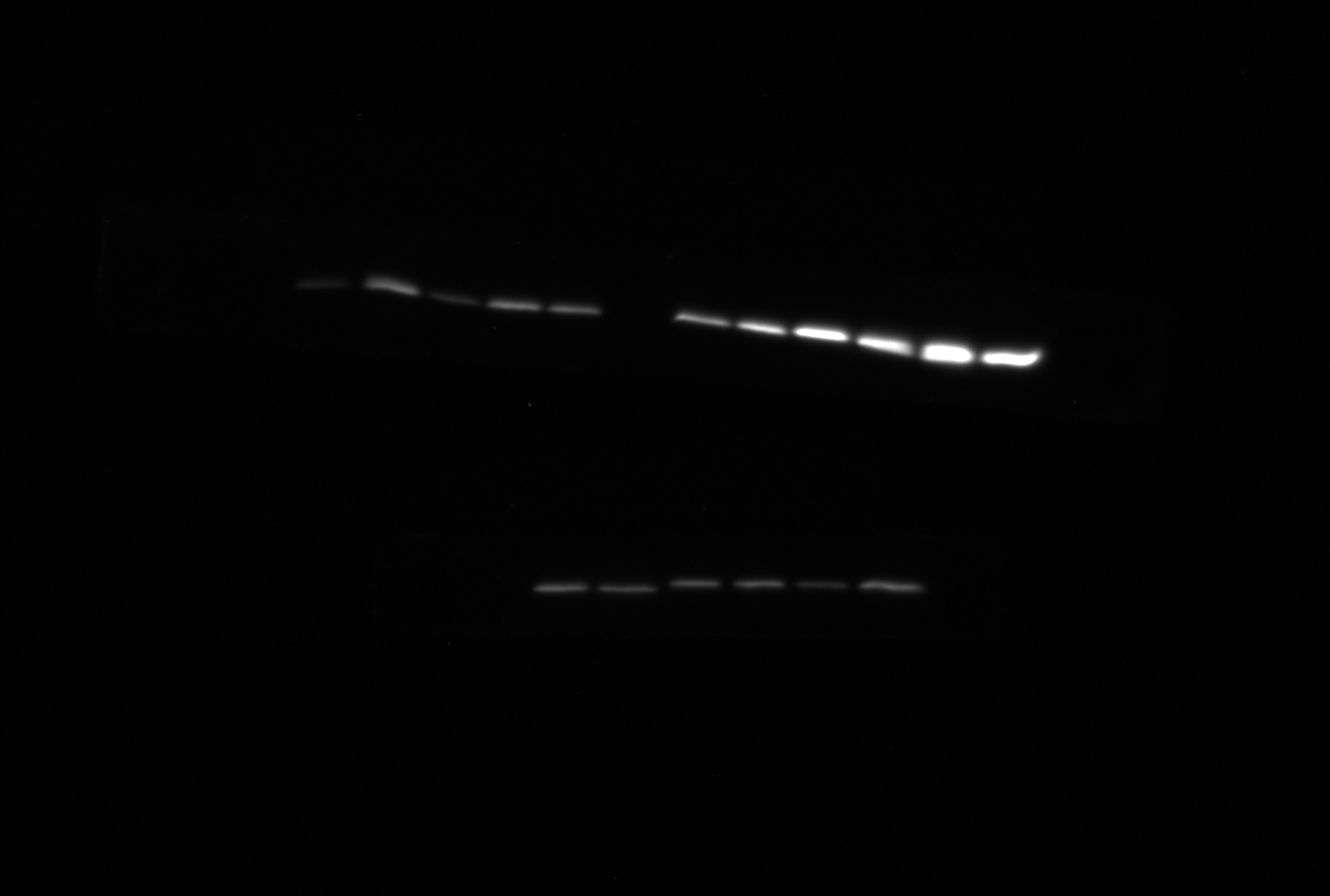

Supplement: Supplementary file 3 — Source data Fig. 1 [file 44319_2024_206_MOESM3_ESM.zip › Figure 1/1F/1F_western_BAKTMD_BOKTMD/1F_western_LgBiT.tif]

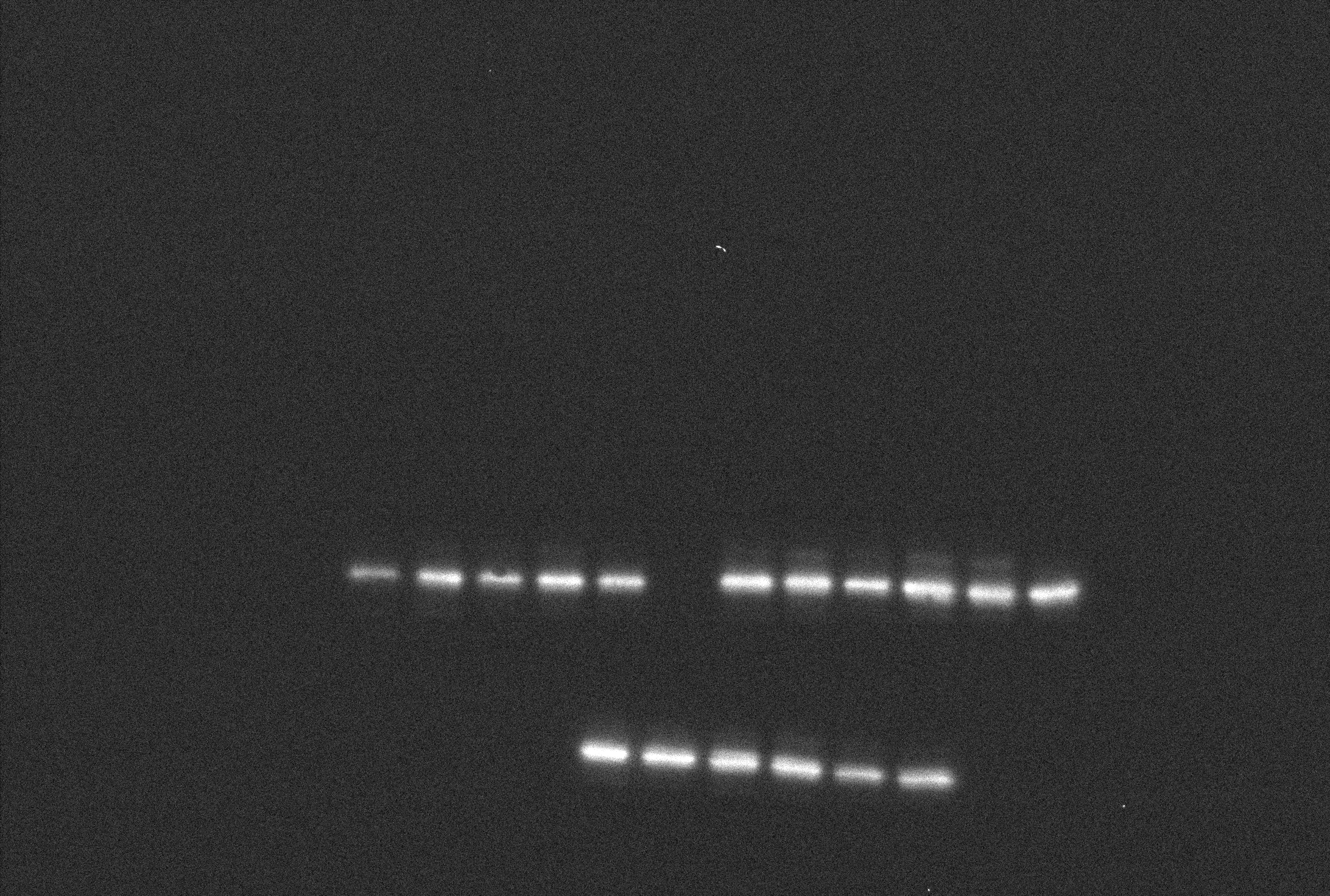

Supplement: Supplementary file 3 — Source data Fig. 1 [file 44319_2024_206_MOESM3_ESM.zip › Figure 1/1F/1F_western_BAKTMD_BOKTMD/1F_western_mTurq2.tif]

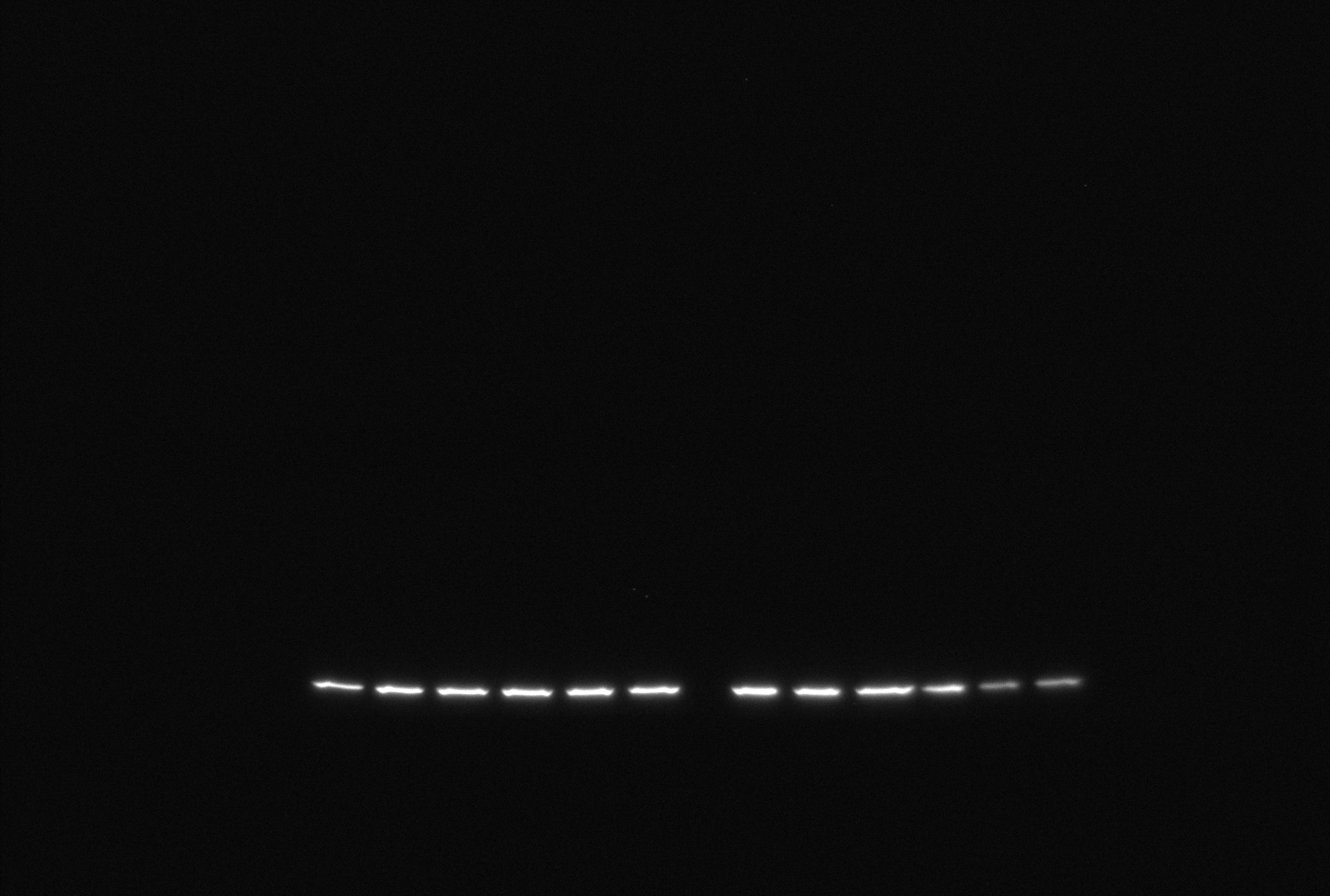

Supplement: Supplementary file 3 — Source data Fig. 1 [file 44319_2024_206_MOESM3_ESM.zip › Figure 1/1F/1F_western_BAXTMD/1F_bActin.tif]

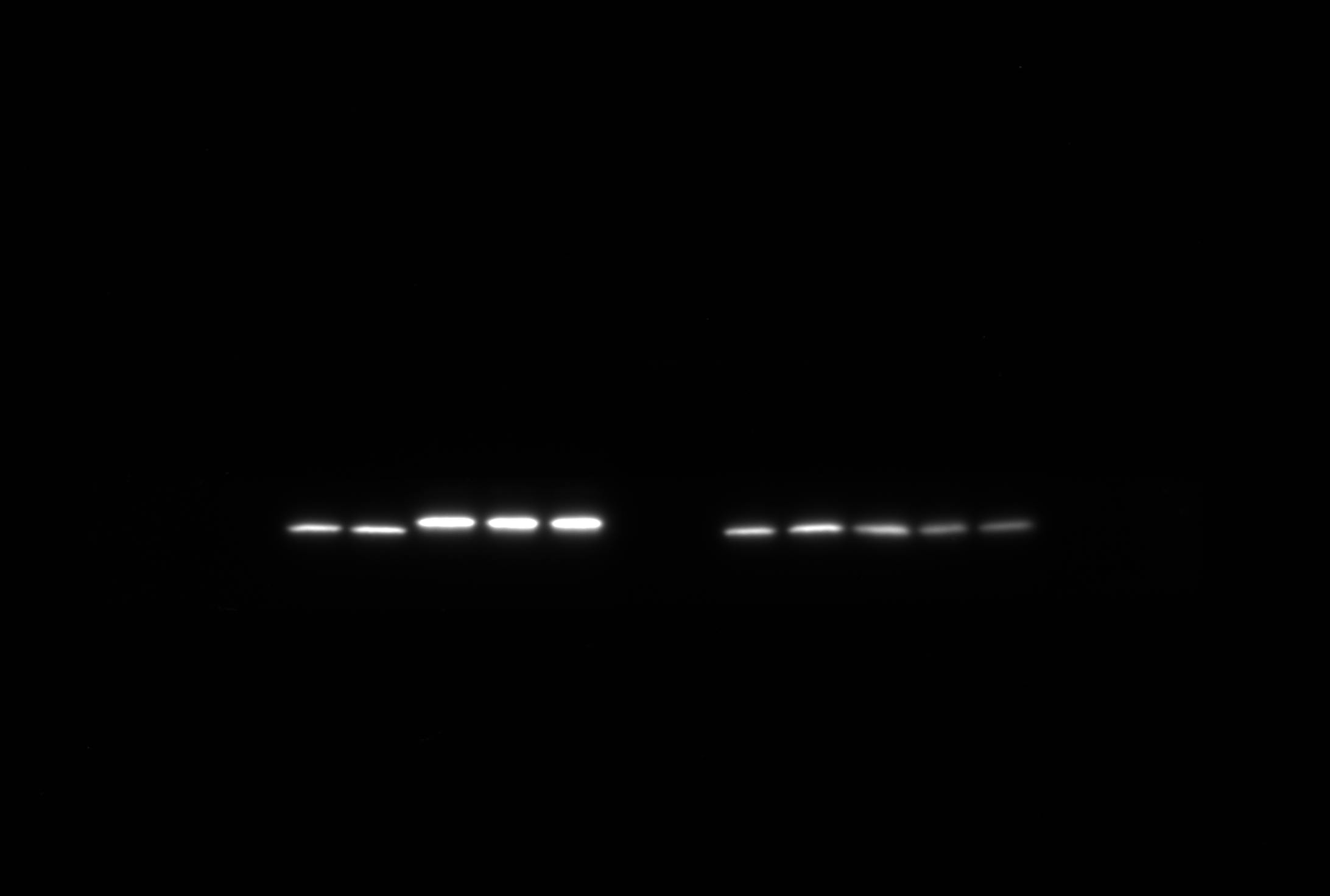

Supplement: Supplementary file 3 — Source data Fig. 1 [file 44319_2024_206_MOESM3_ESM.zip › Figure 1/1F/1F_western_BAXTMD/1F_LgBiT.tif]

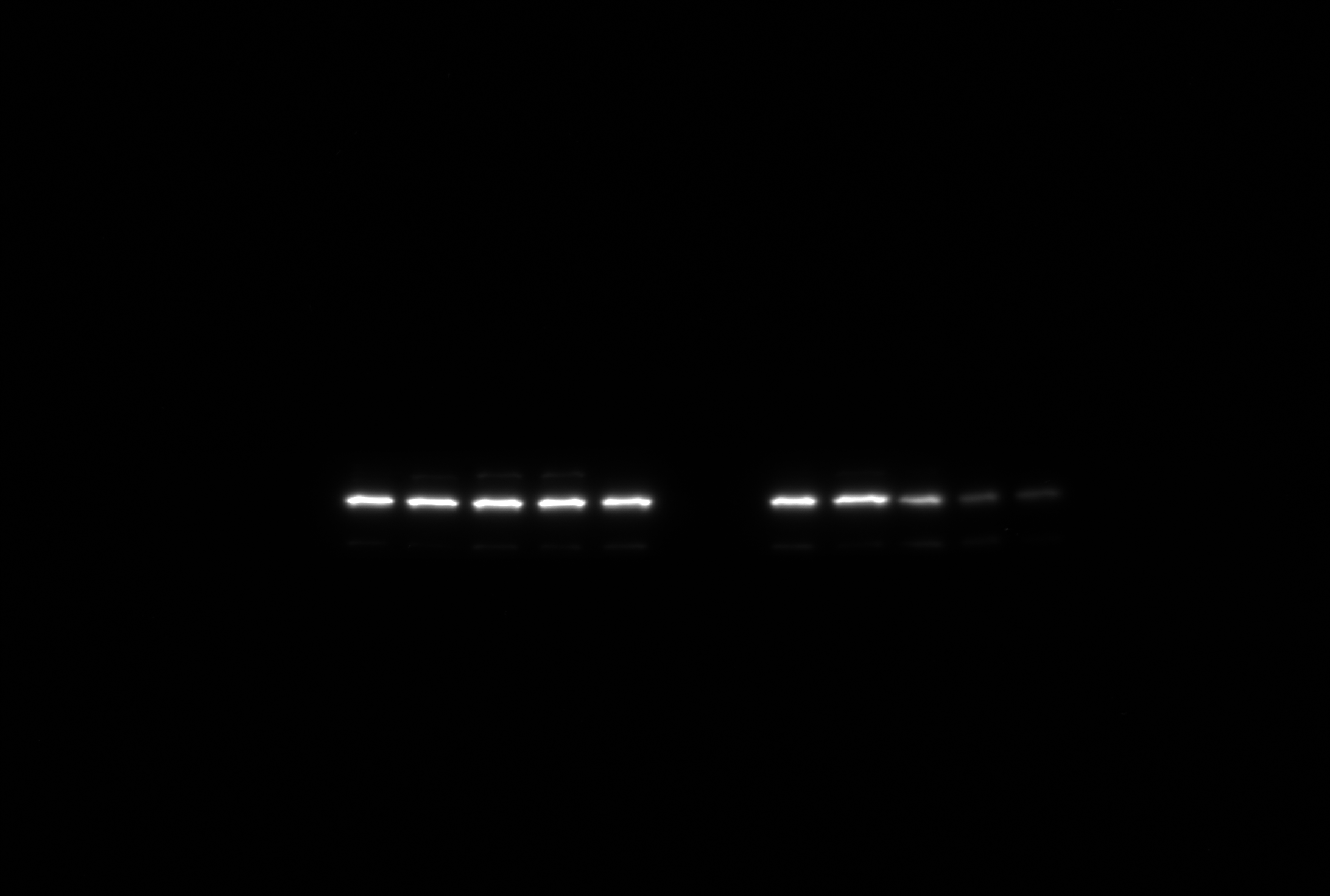

Supplement: Supplementary file 3 — Source data Fig. 1 [file 44319_2024_206_MOESM3_ESM.zip › Figure 1/1F/1F_western_BAXTMD/1F_mTurq2.tif]

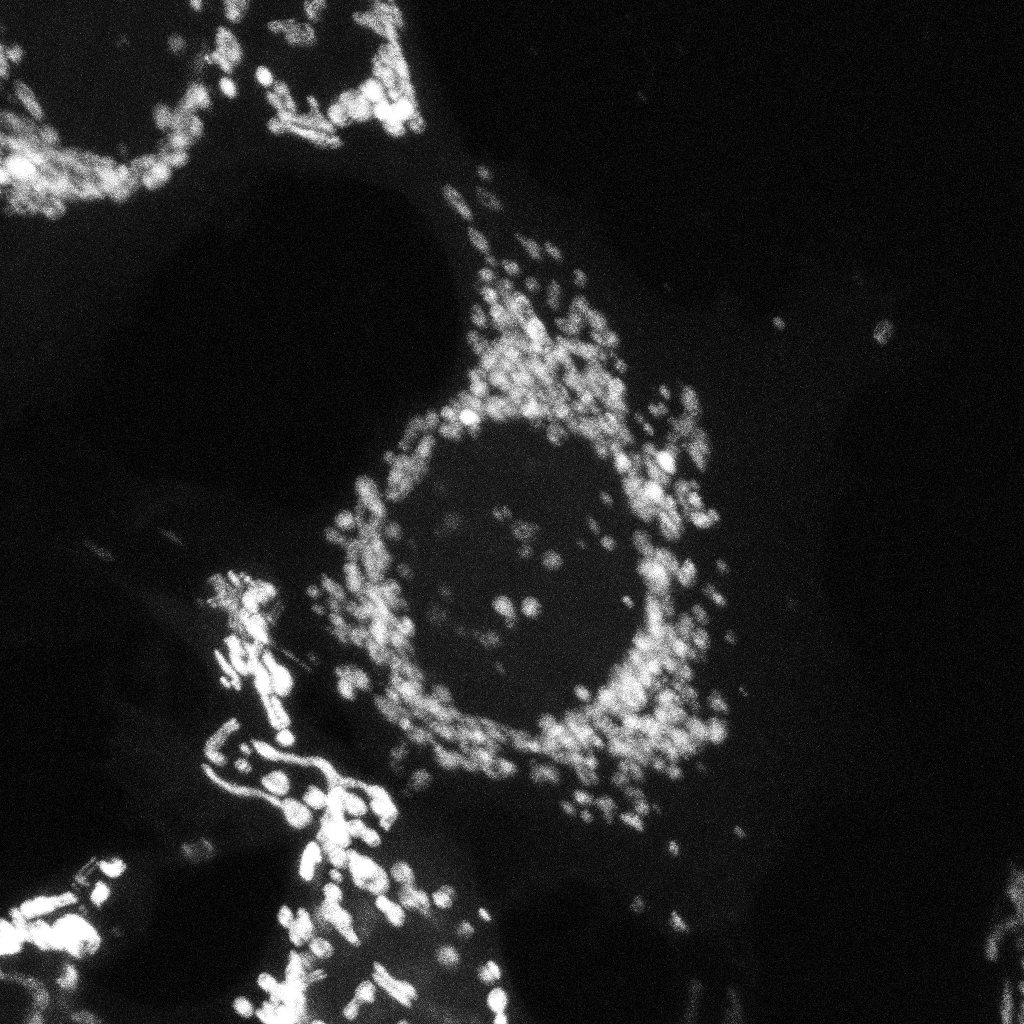

Supplement: Supplementary file 4 — Source data Fig. 2 [file 44319_2024_206_MOESM4_ESM.zip › Figure 2/2A/2A_BAKTMD_EYFPMito.tif]

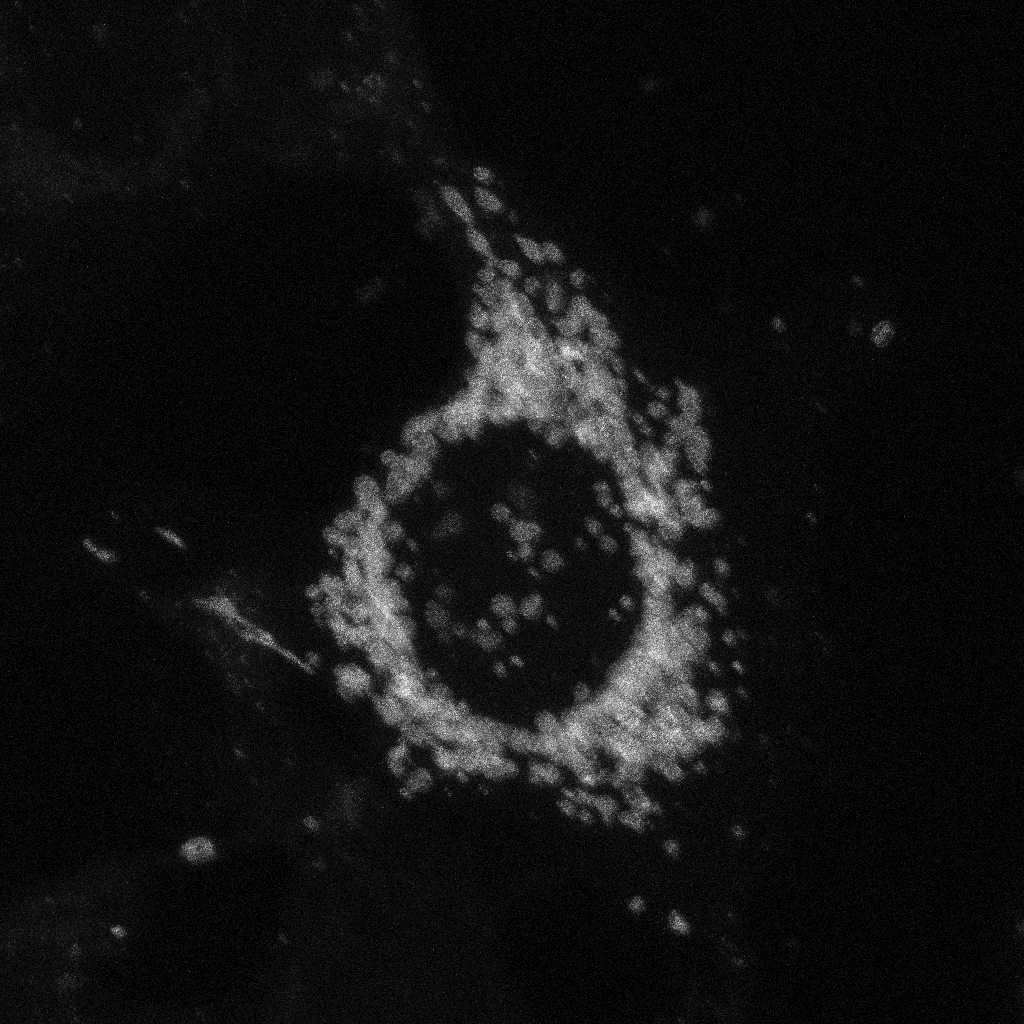

Supplement: Supplementary file 4 — Source data Fig. 2 [file 44319_2024_206_MOESM4_ESM.zip › Figure 2/2A/2A_BAKTMD_mTurq2.tif]

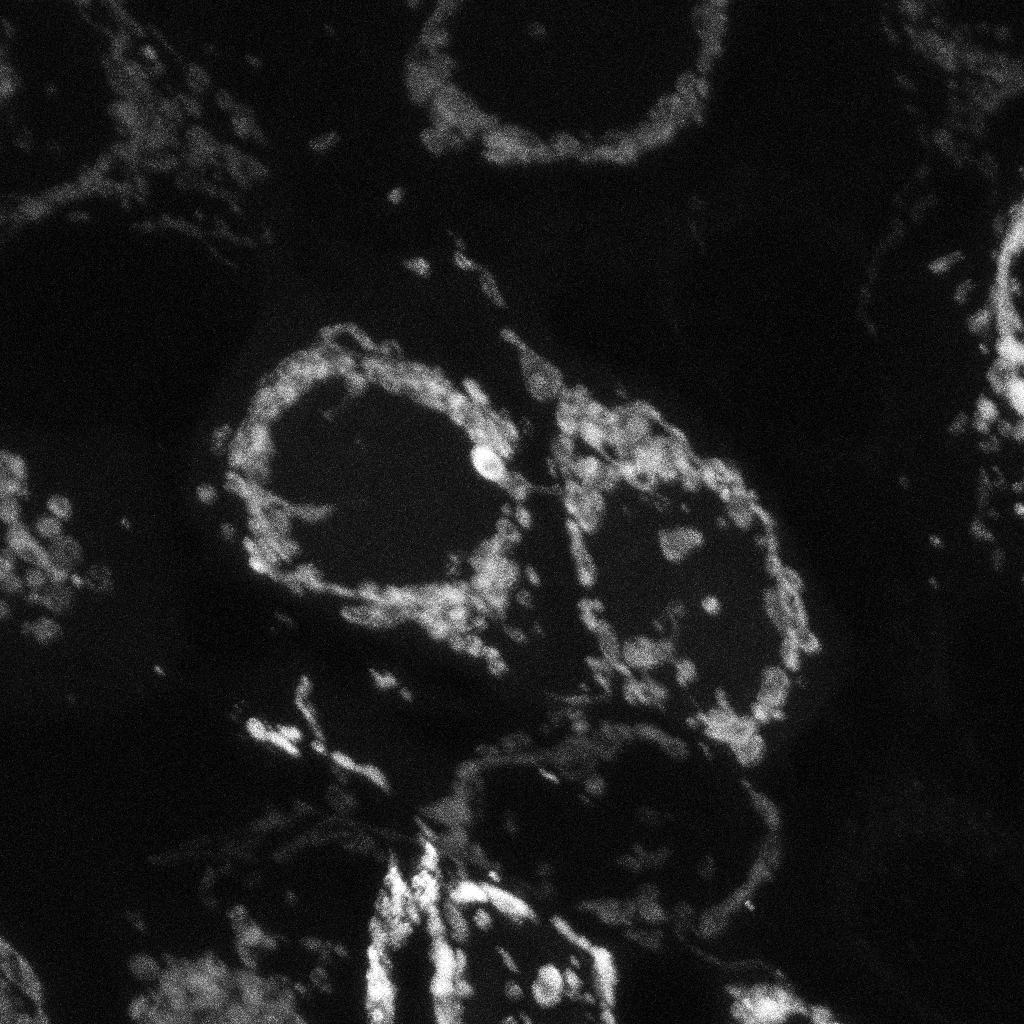

Supplement: Supplementary file 4 — Source data Fig. 2 [file 44319_2024_206_MOESM4_ESM.zip › Figure 2/2A/2A_BAXTMD_EYFPMito.tif]

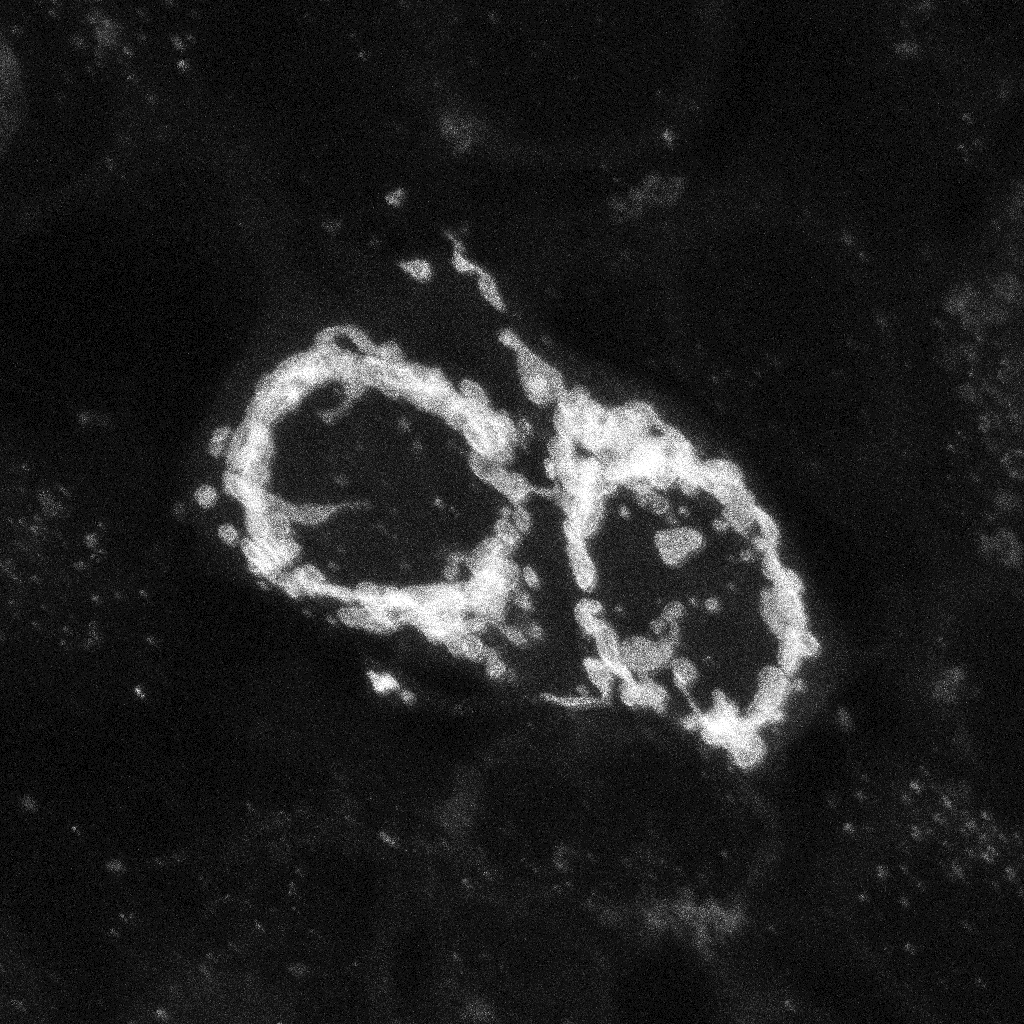

Supplement: Supplementary file 4 — Source data Fig. 2 [file 44319_2024_206_MOESM4_ESM.zip › Figure 2/2A/2A_BAXTMD_mTurq2.tif]

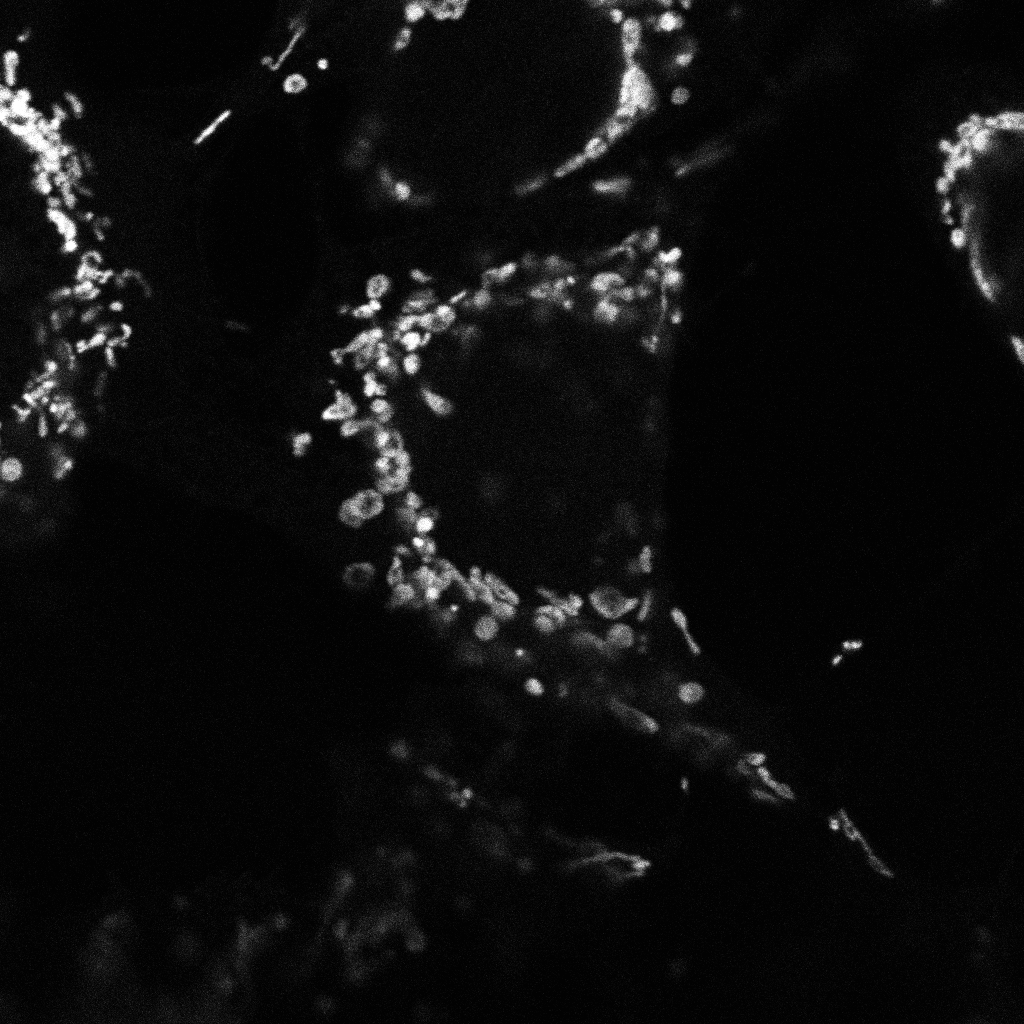

Supplement: Supplementary file 4 — Source data Fig. 2 [file 44319_2024_206_MOESM4_ESM.zip › Figure 2/2A/2A_BCL2TMD_EYFPMito.tif]

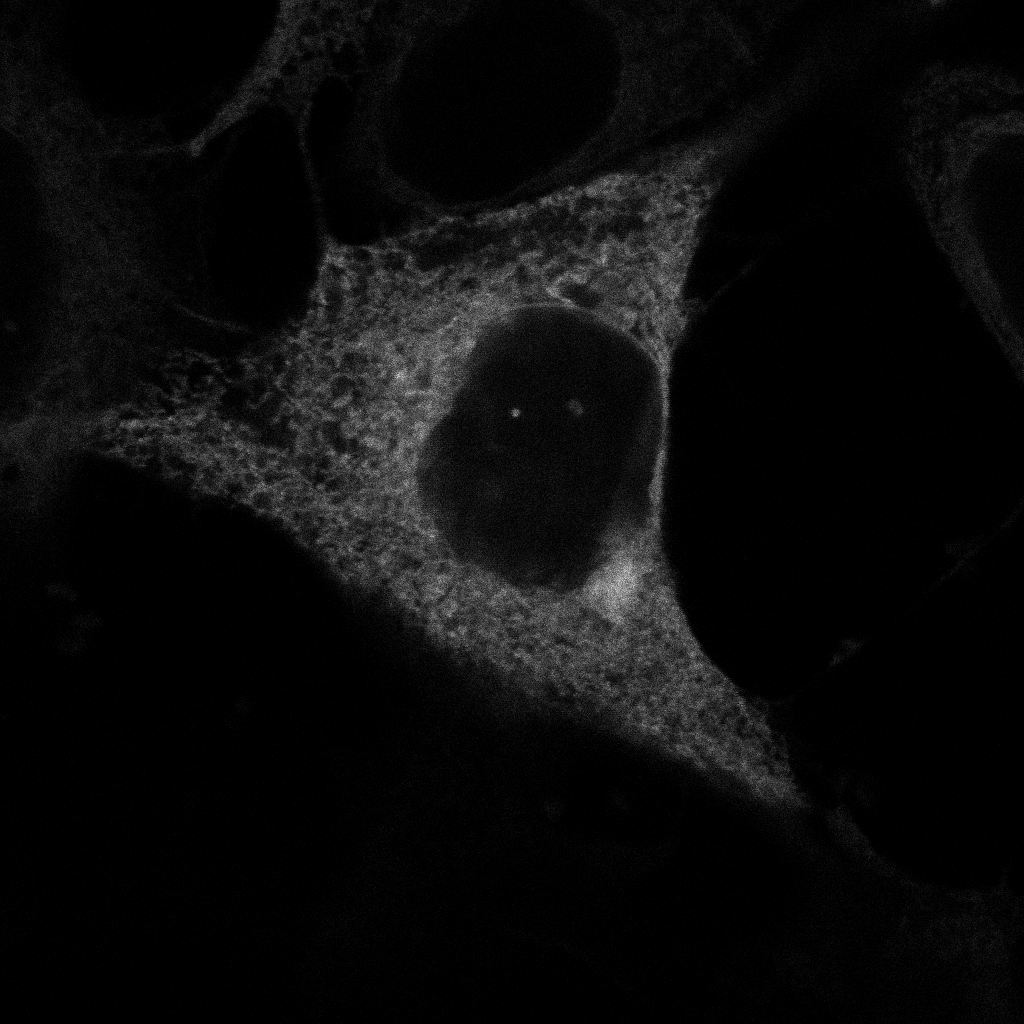

Supplement: Supplementary file 4 — Source data Fig. 2 [file 44319_2024_206_MOESM4_ESM.zip › Figure 2/2A/2A_BCL2TMD_mTurq2.tif]

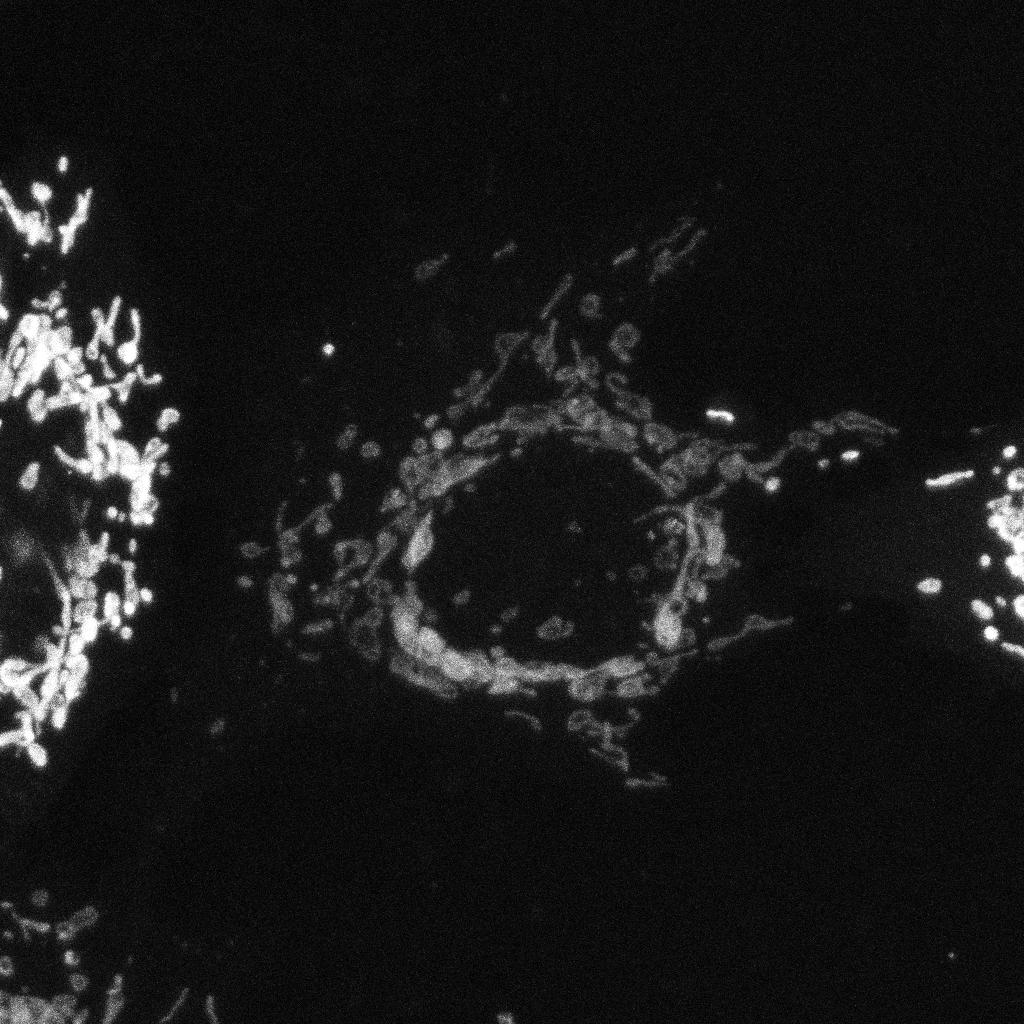

Supplement: Supplementary file 4 — Source data Fig. 2 [file 44319_2024_206_MOESM4_ESM.zip › Figure 2/2A/2A_BOKTMD_EYFPMito.tif]

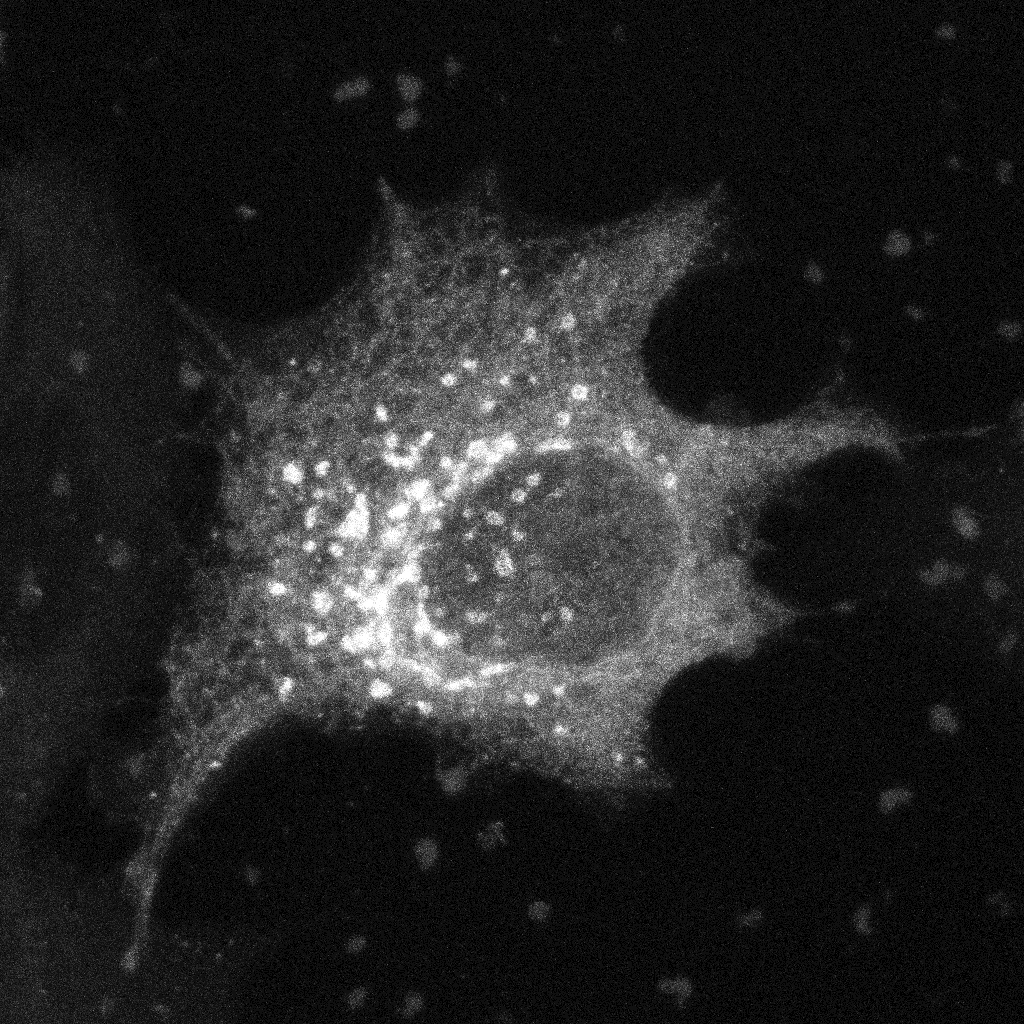

Supplement: Supplementary file 4 — Source data Fig. 2 [file 44319_2024_206_MOESM4_ESM.zip › Figure 2/2A/2A_BOKTMD_mTurq2.tif]

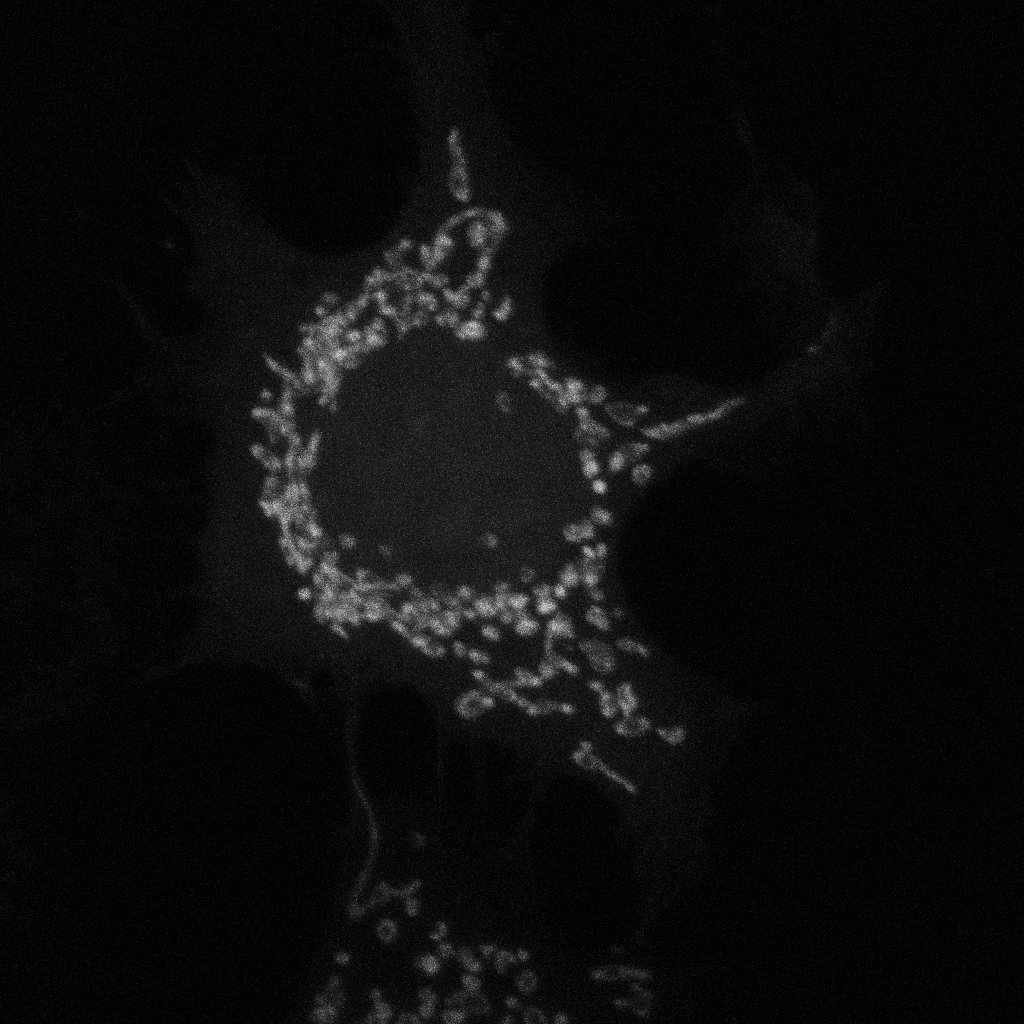

Supplement: Supplementary file 4 — Source data Fig. 2 [file 44319_2024_206_MOESM4_ESM.zip › Figure 2/2A/2A_cb5TMD_EYFPMito.tif]

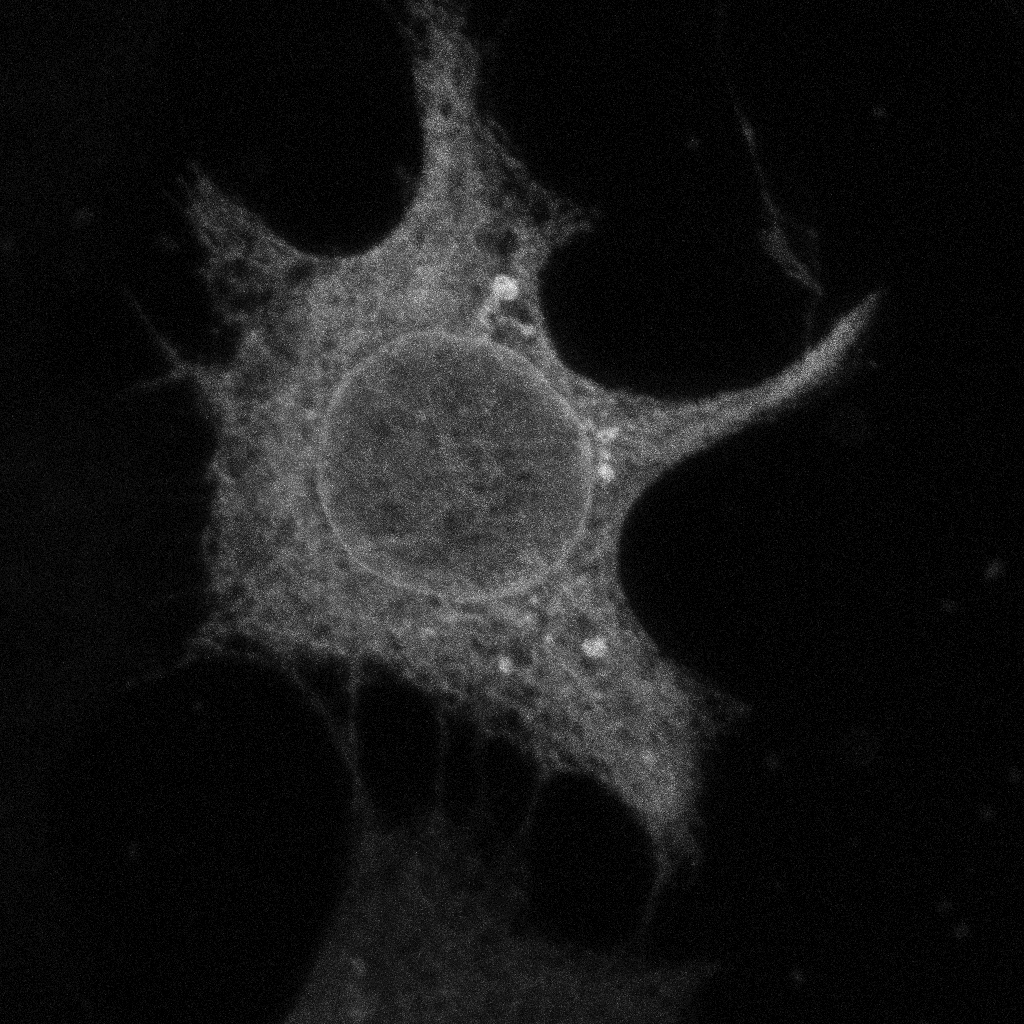

Supplement: Supplementary file 4 — Source data Fig. 2 [file 44319_2024_206_MOESM4_ESM.zip › Figure 2/2A/2A_cb5TMD_mTurq2.tif]

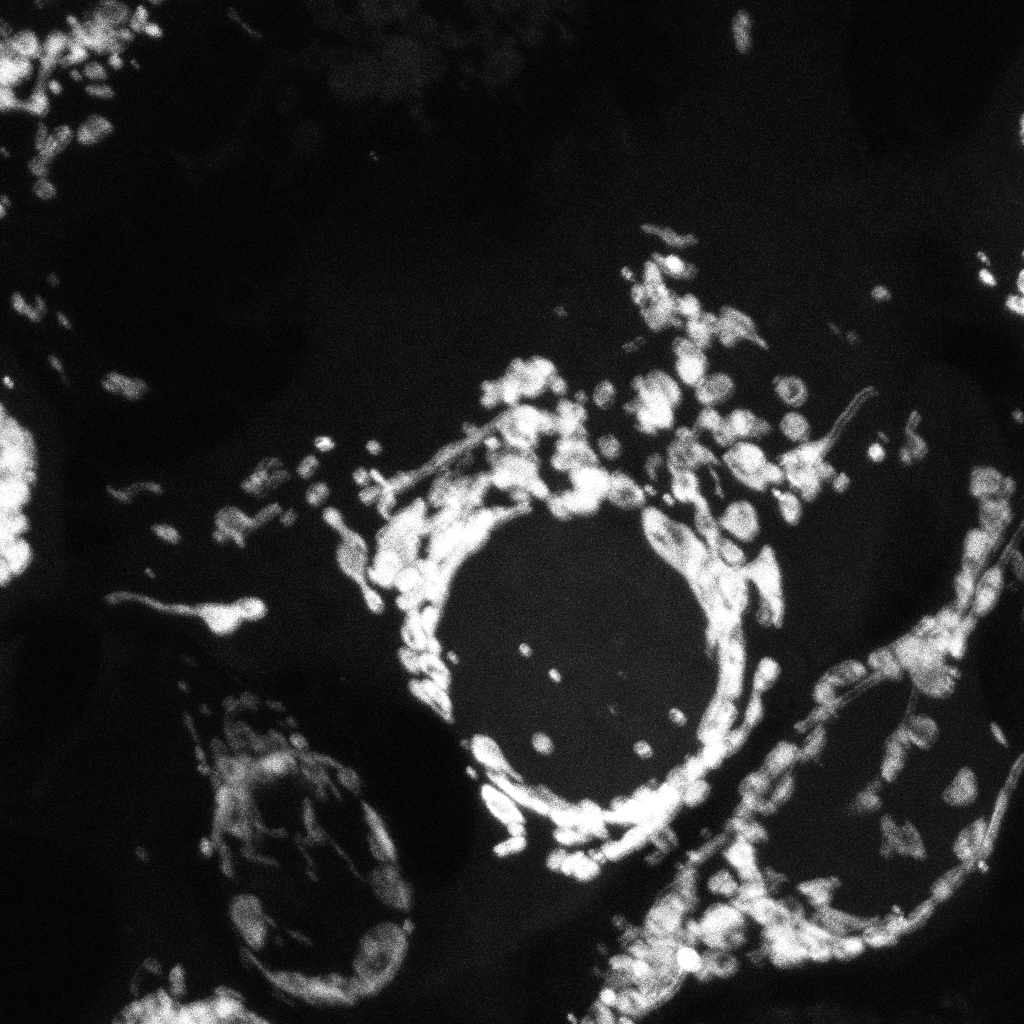

Supplement: Supplementary file 4 — Source data Fig. 2 [file 44319_2024_206_MOESM4_ESM.zip › Figure 2/2A/2A_TOM5TMD_EYFPMito.tif]

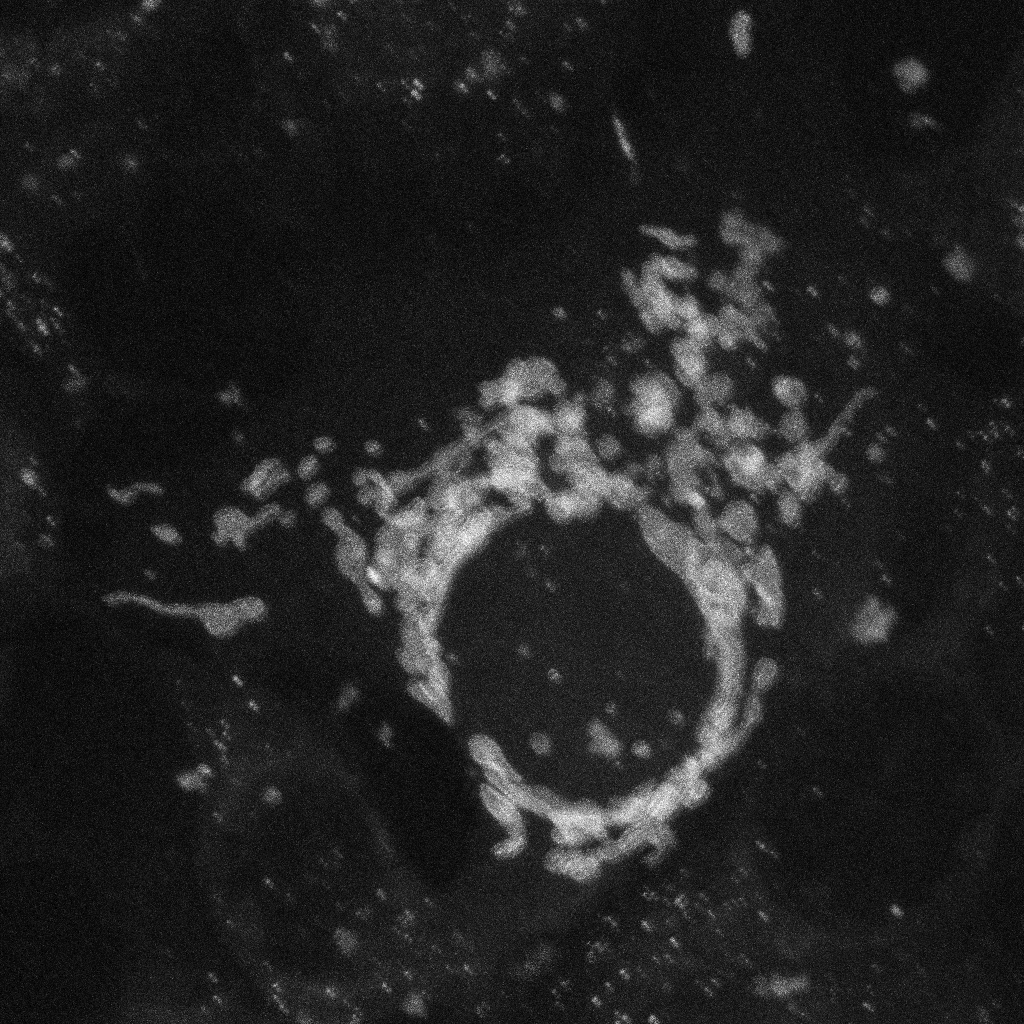

Supplement: Supplementary file 4 — Source data Fig. 2 [file 44319_2024_206_MOESM4_ESM.zip › Figure 2/2A/2A_TOM5TMD_mTurq2.tif]

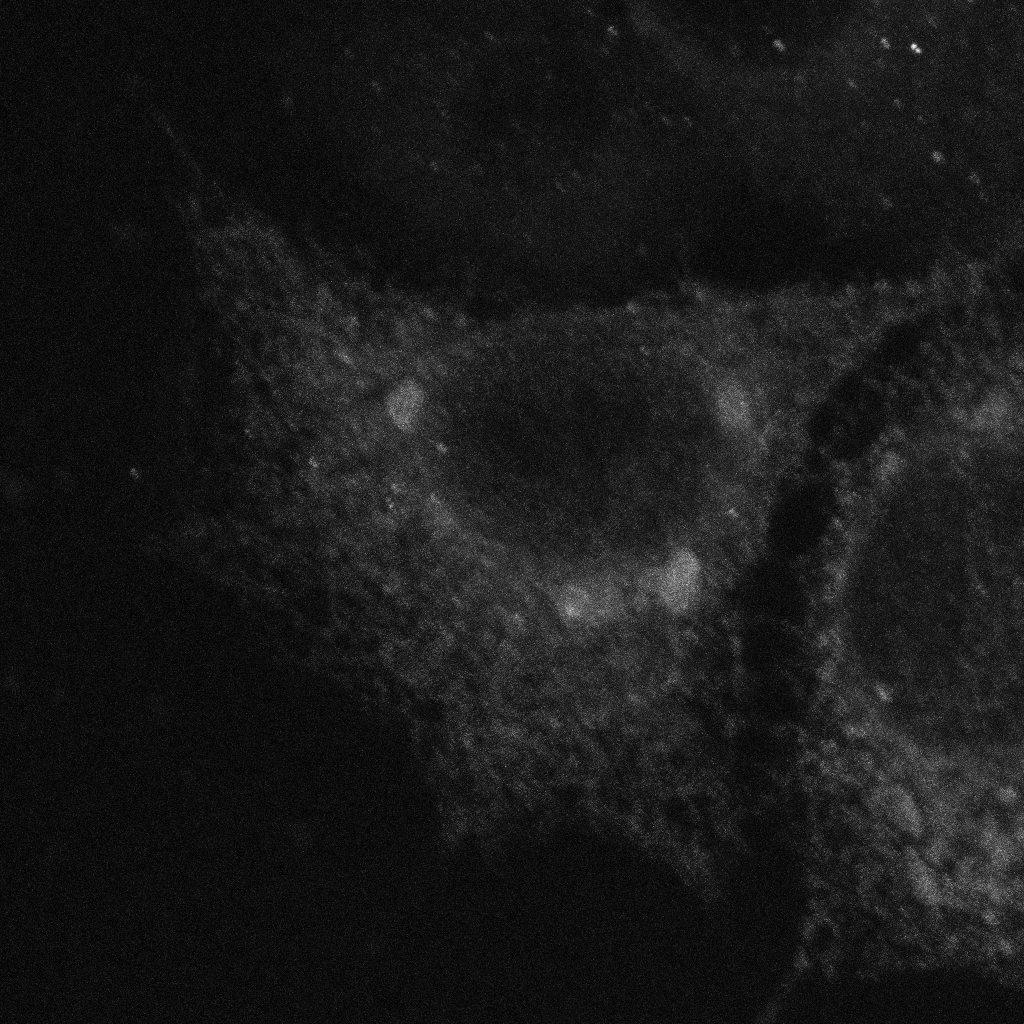

Supplement: Supplementary file 4 — Source data Fig. 2 [file 44319_2024_206_MOESM4_ESM.zip › Figure 2/2B/2B_BAKTMD_EYFPER.tif]

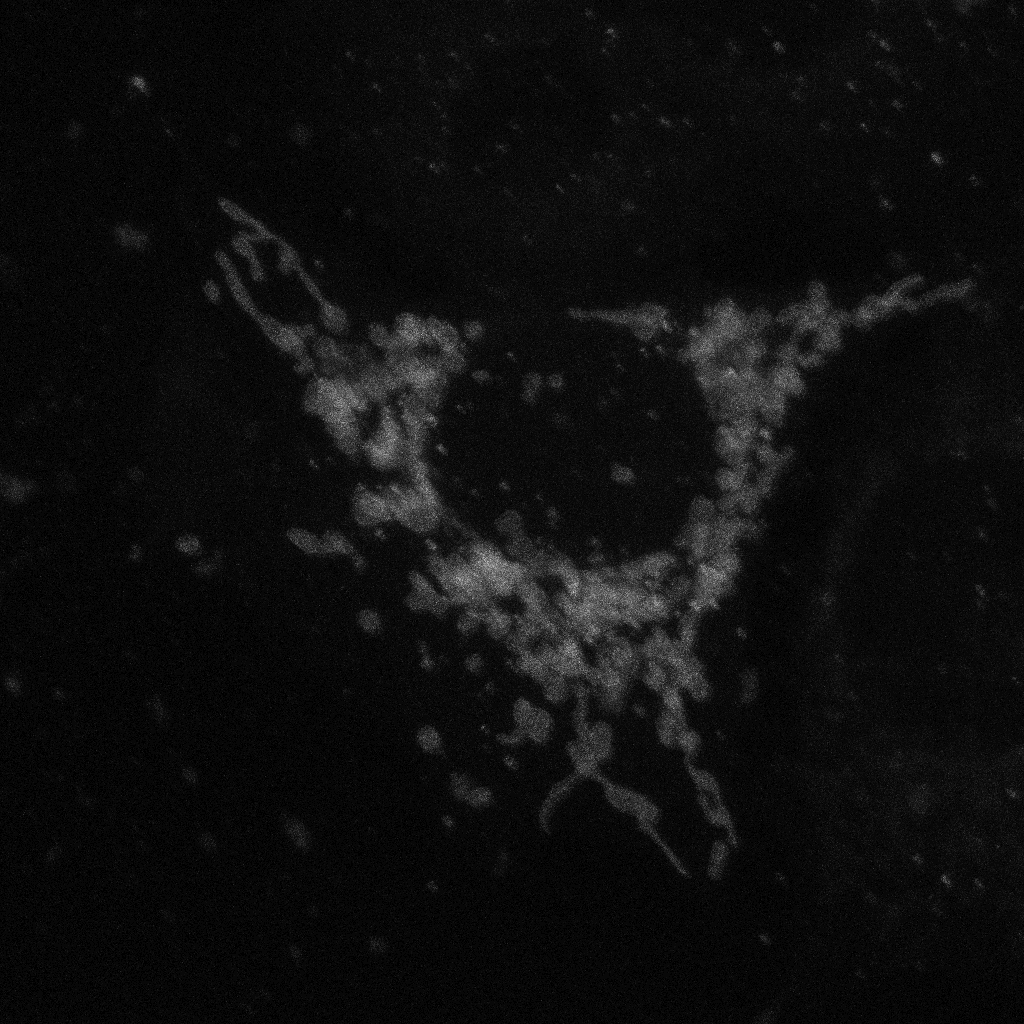

Supplement: Supplementary file 4 — Source data Fig. 2 [file 44319_2024_206_MOESM4_ESM.zip › Figure 2/2B/2B_BAKTMD_mTurq2.tif]

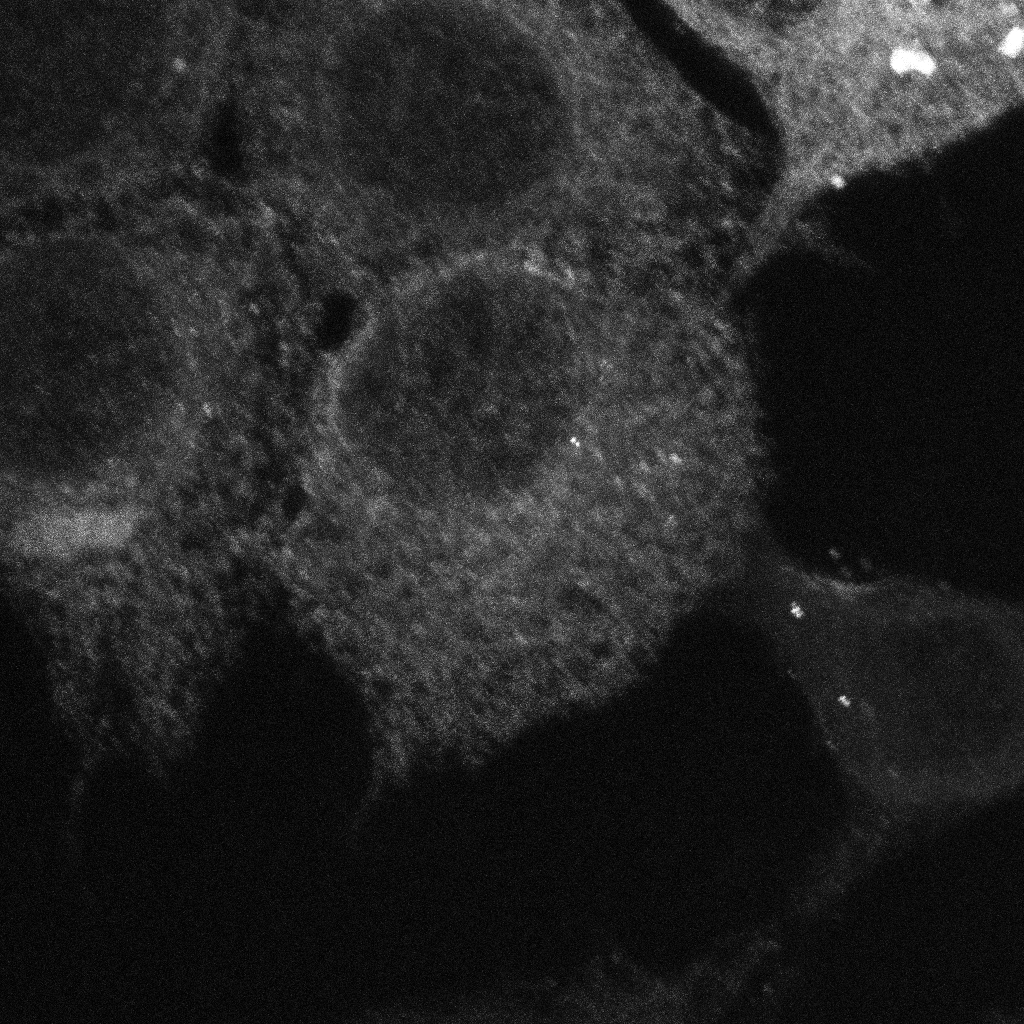

Supplement: Supplementary file 4 — Source data Fig. 2 [file 44319_2024_206_MOESM4_ESM.zip › Figure 2/2B/2B_BAXTMD_EYFPER.tif]

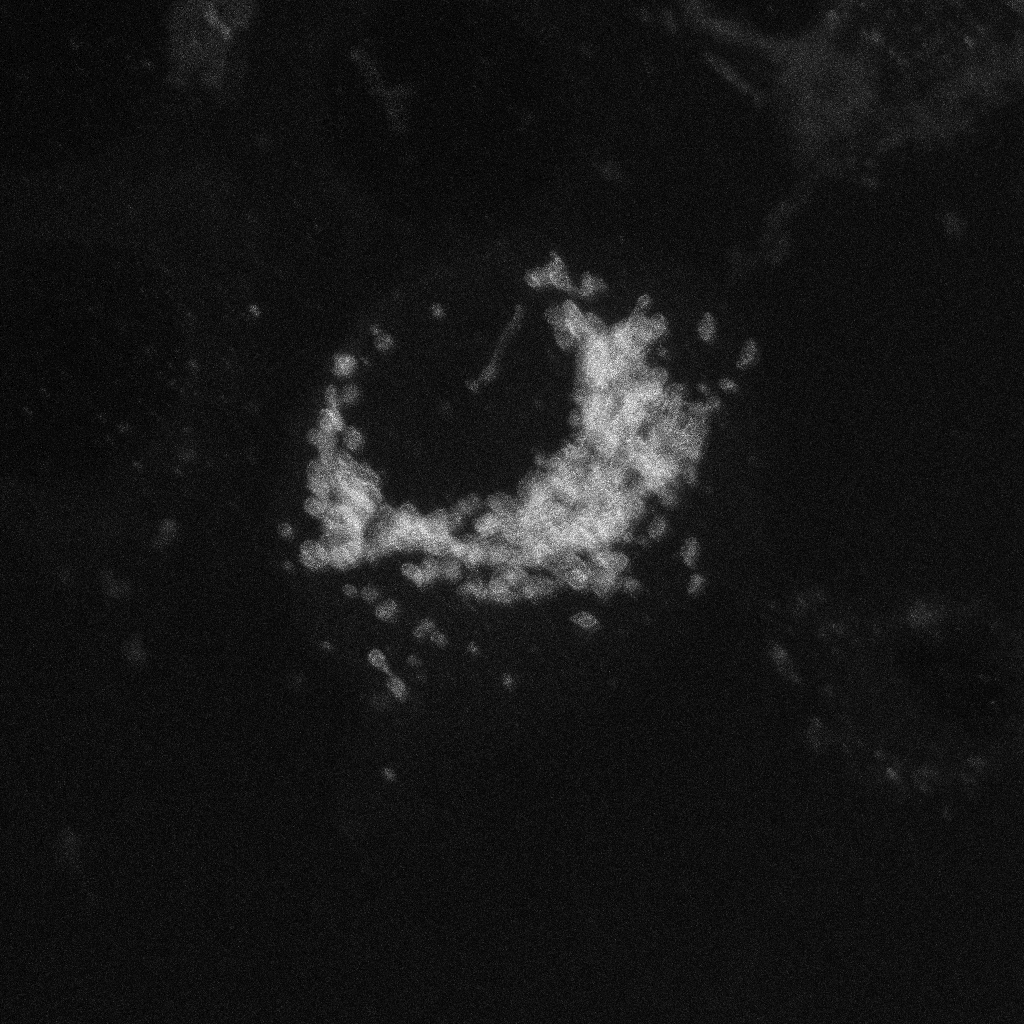

Supplement: Supplementary file 4 — Source data Fig. 2 [file 44319_2024_206_MOESM4_ESM.zip › Figure 2/2B/2B_BAXTMD_mTurq2.tif]

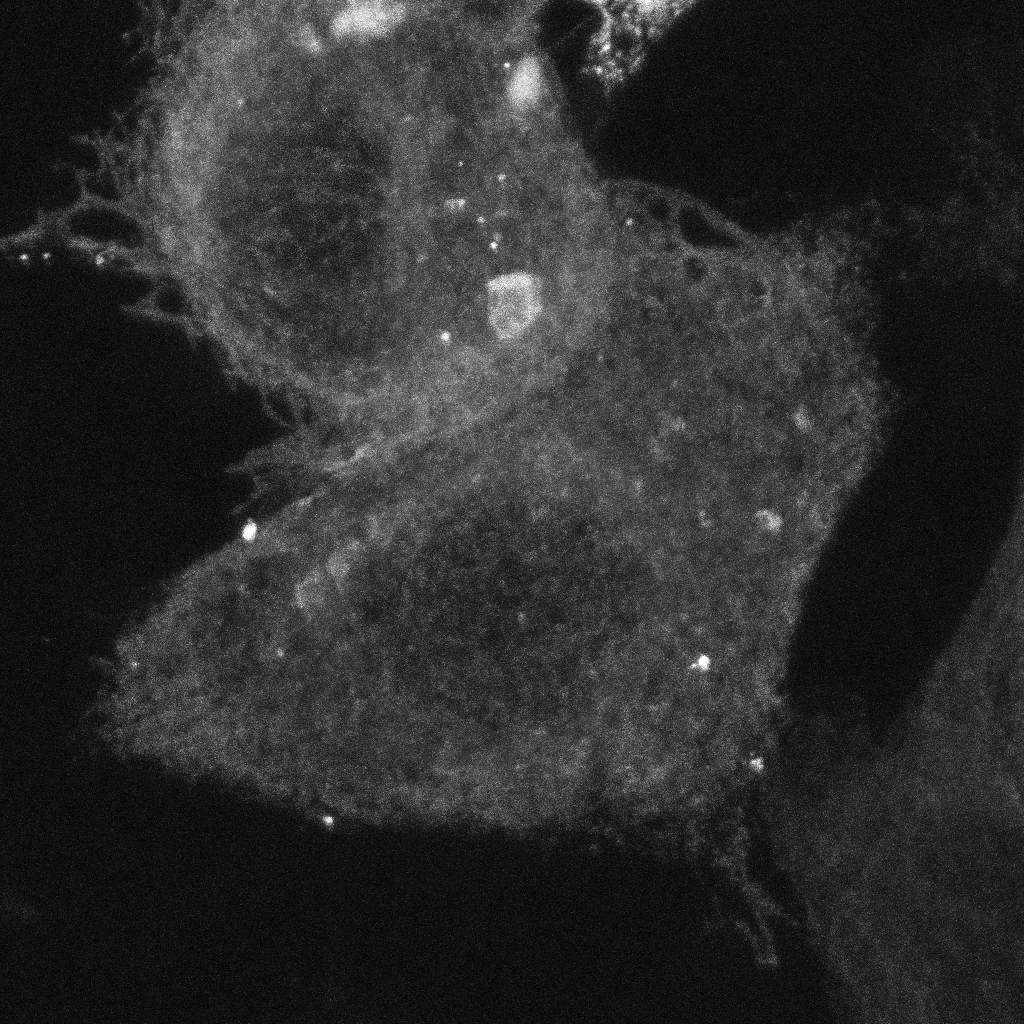

Supplement: Supplementary file 4 — Source data Fig. 2 [file 44319_2024_206_MOESM4_ESM.zip › Figure 2/2B/2B_BCL2TMD_EYFPER.tif]

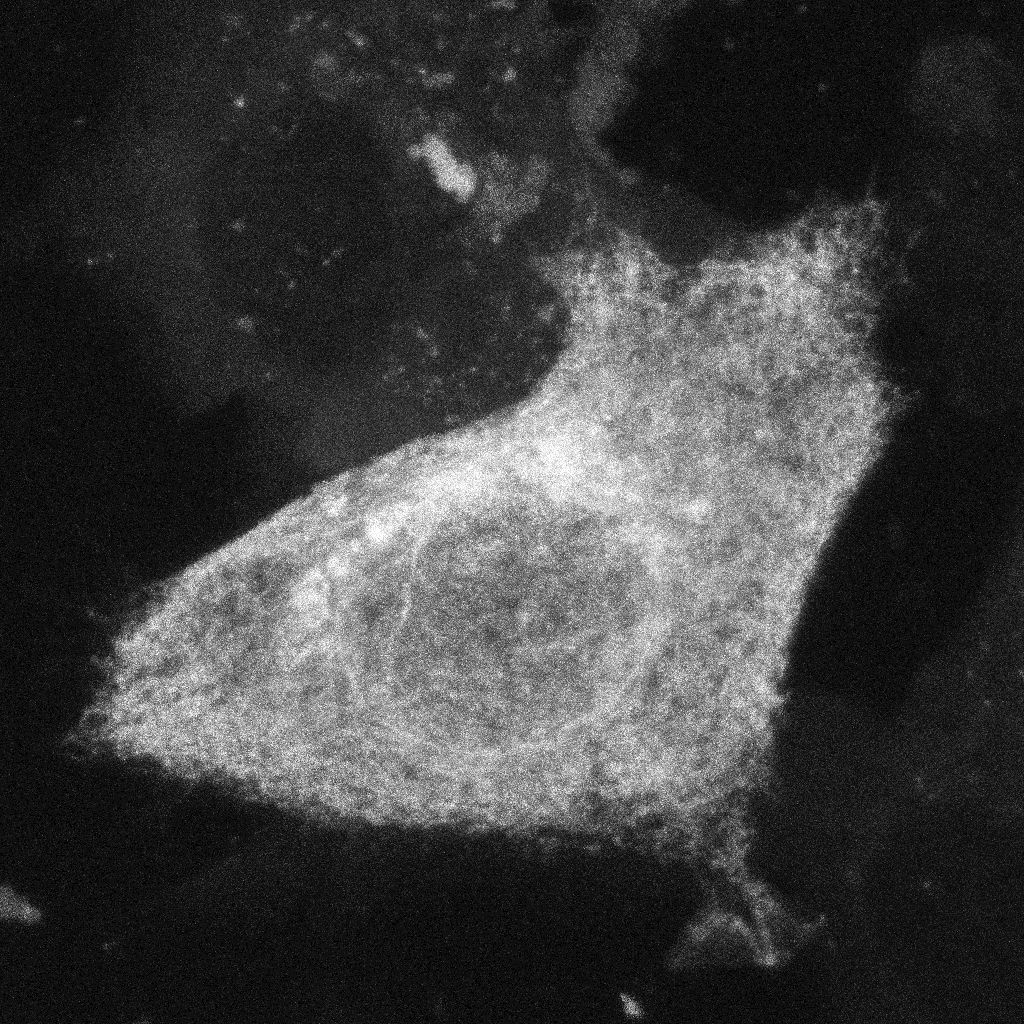

Supplement: Supplementary file 4 — Source data Fig. 2 [file 44319_2024_206_MOESM4_ESM.zip › Figure 2/2B/2B_BCL2TMD_mTurq2.tif]

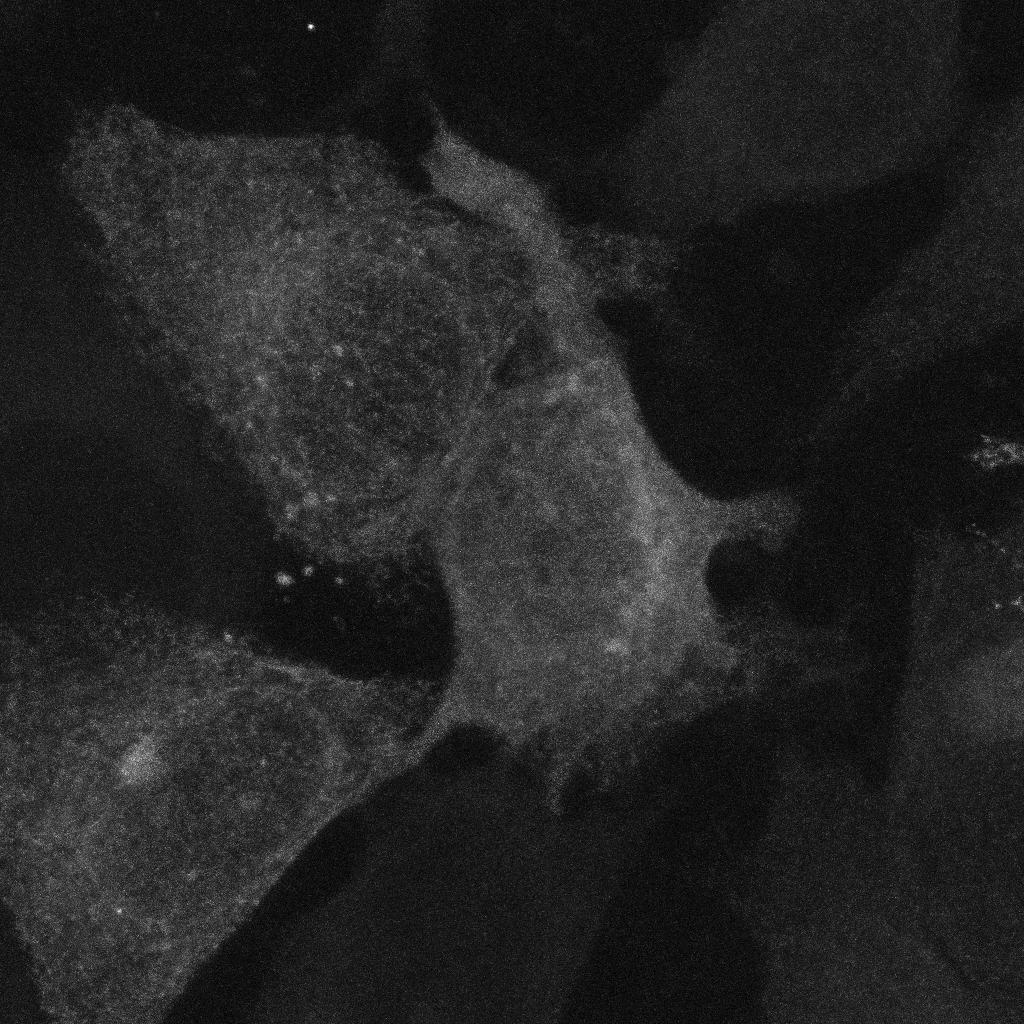

Supplement: Supplementary file 4 — Source data Fig. 2 [file 44319_2024_206_MOESM4_ESM.zip › Figure 2/2B/2B_BOKTMD_EYFPER.tif]

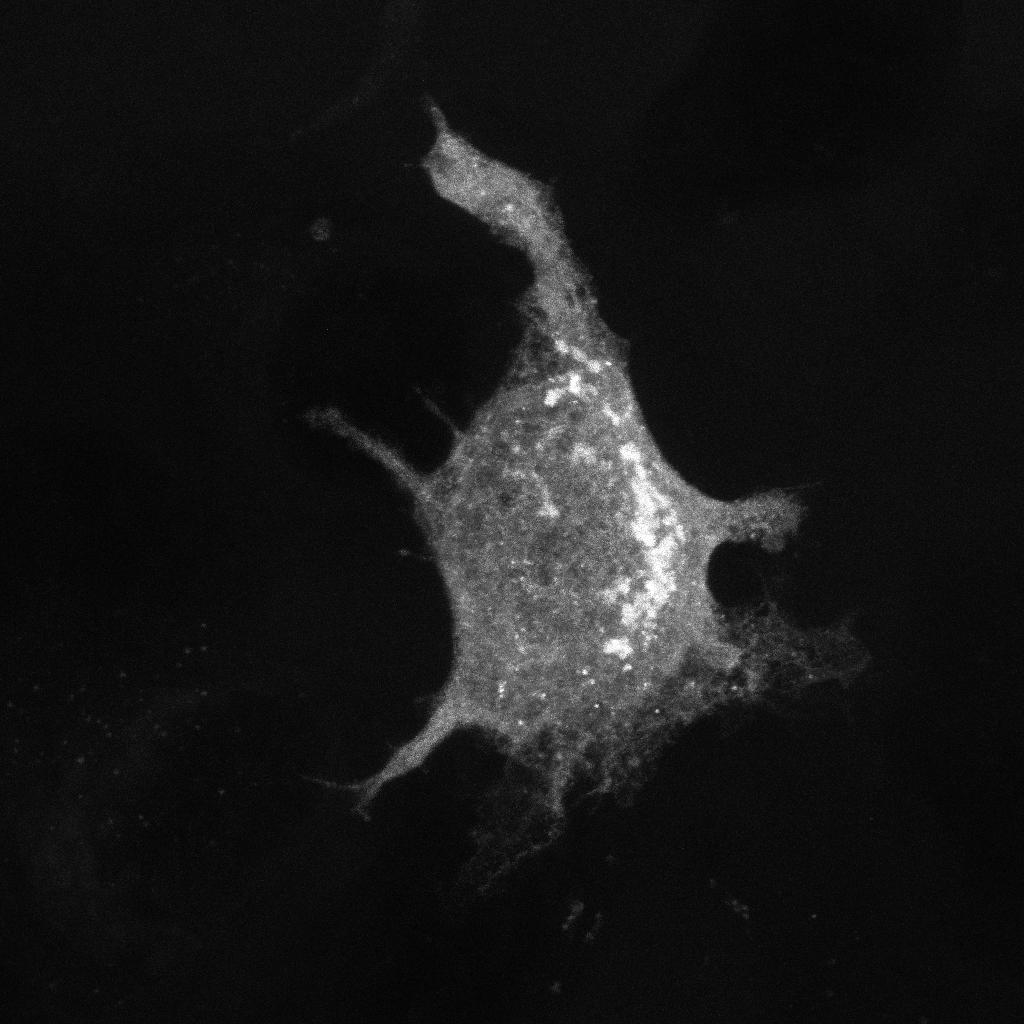

Supplement: Supplementary file 4 — Source data Fig. 2 [file 44319_2024_206_MOESM4_ESM.zip › Figure 2/2B/2B_BOKTMD_mTurq2.tif]

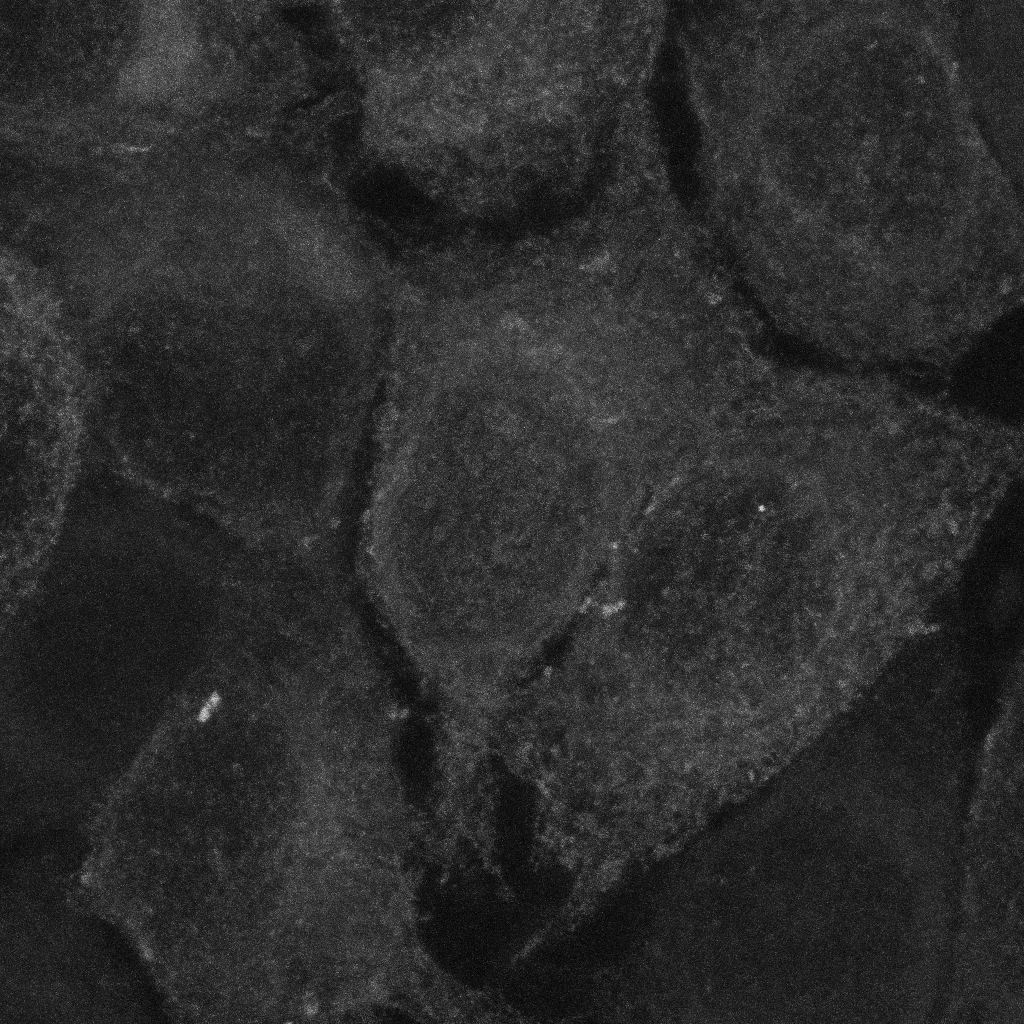

Supplement: Supplementary file 4 — Source data Fig. 2 [file 44319_2024_206_MOESM4_ESM.zip › Figure 2/2B/2B_cb5TMD_EYFPER.tif]

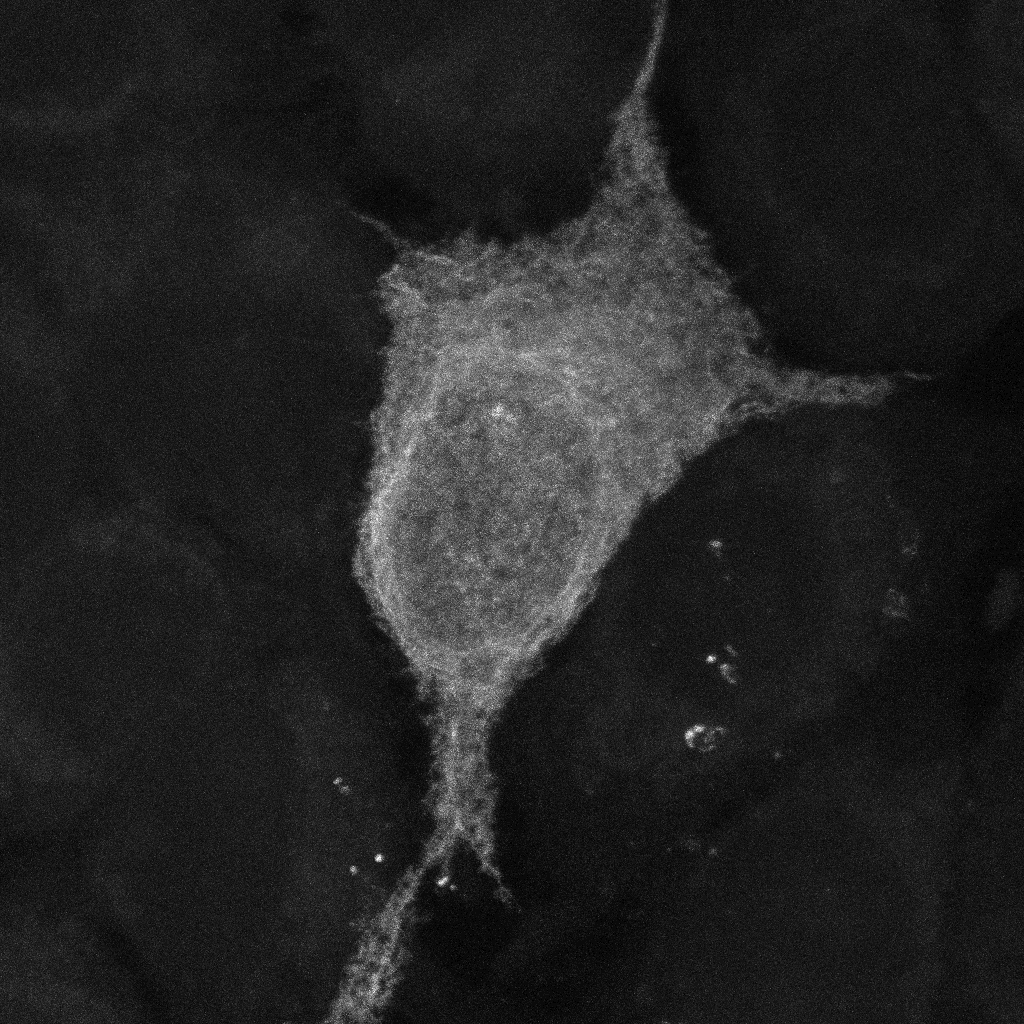

Supplement: Supplementary file 4 — Source data Fig. 2 [file 44319_2024_206_MOESM4_ESM.zip › Figure 2/2B/2B_cb5TMD_mTurq2.tif]

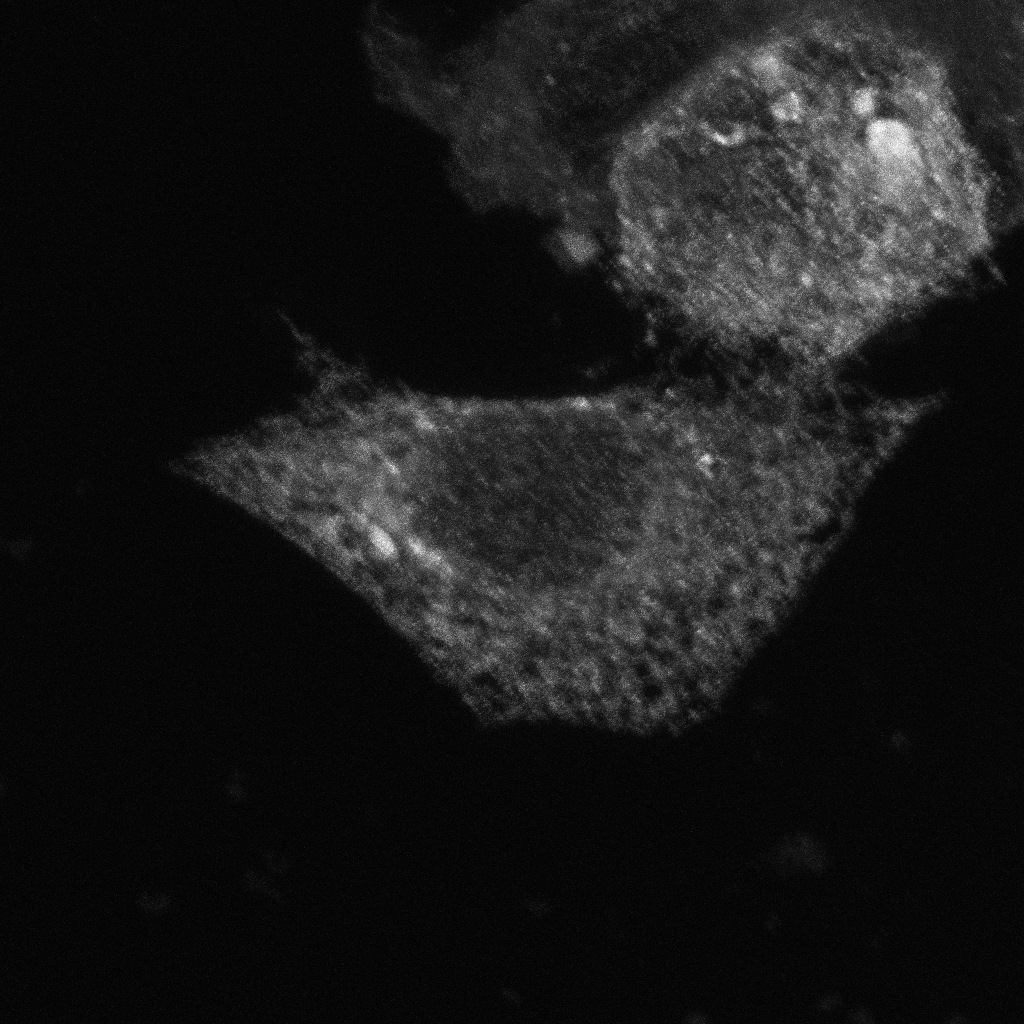

Supplement: Supplementary file 4 — Source data Fig. 2 [file 44319_2024_206_MOESM4_ESM.zip › Figure 2/2B/2B_TOM5TMD_EYFPER.tif]

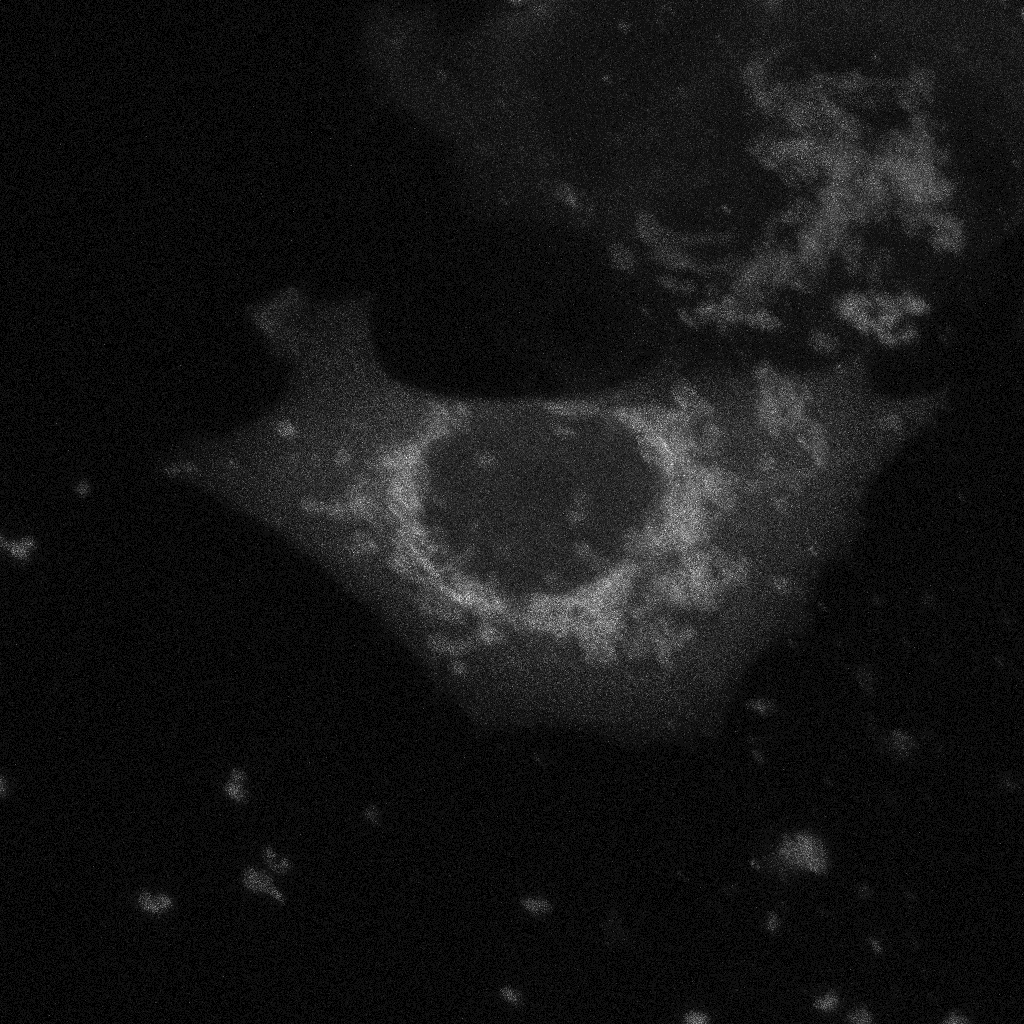

Supplement: Supplementary file 4 — Source data Fig. 2 [file 44319_2024_206_MOESM4_ESM.zip › Figure 2/2B/2B_TOM5TMD_mTurq2.tif]

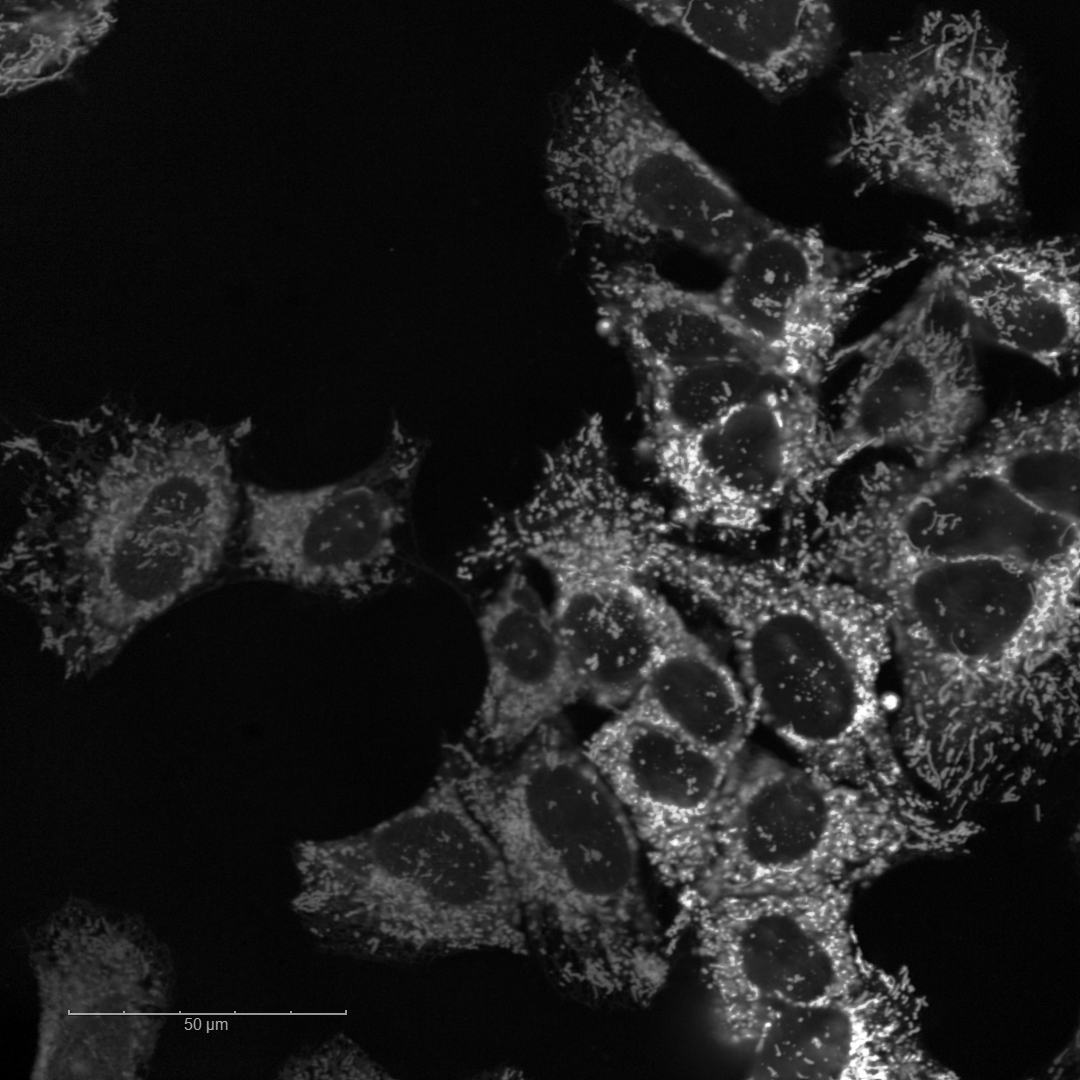

Supplement: Supplementary file 4 — Source data Fig. 2 [file 44319_2024_206_MOESM4_ESM.zip › Figure 2/2D/2D_mTurq2-BAKTMD.tif]

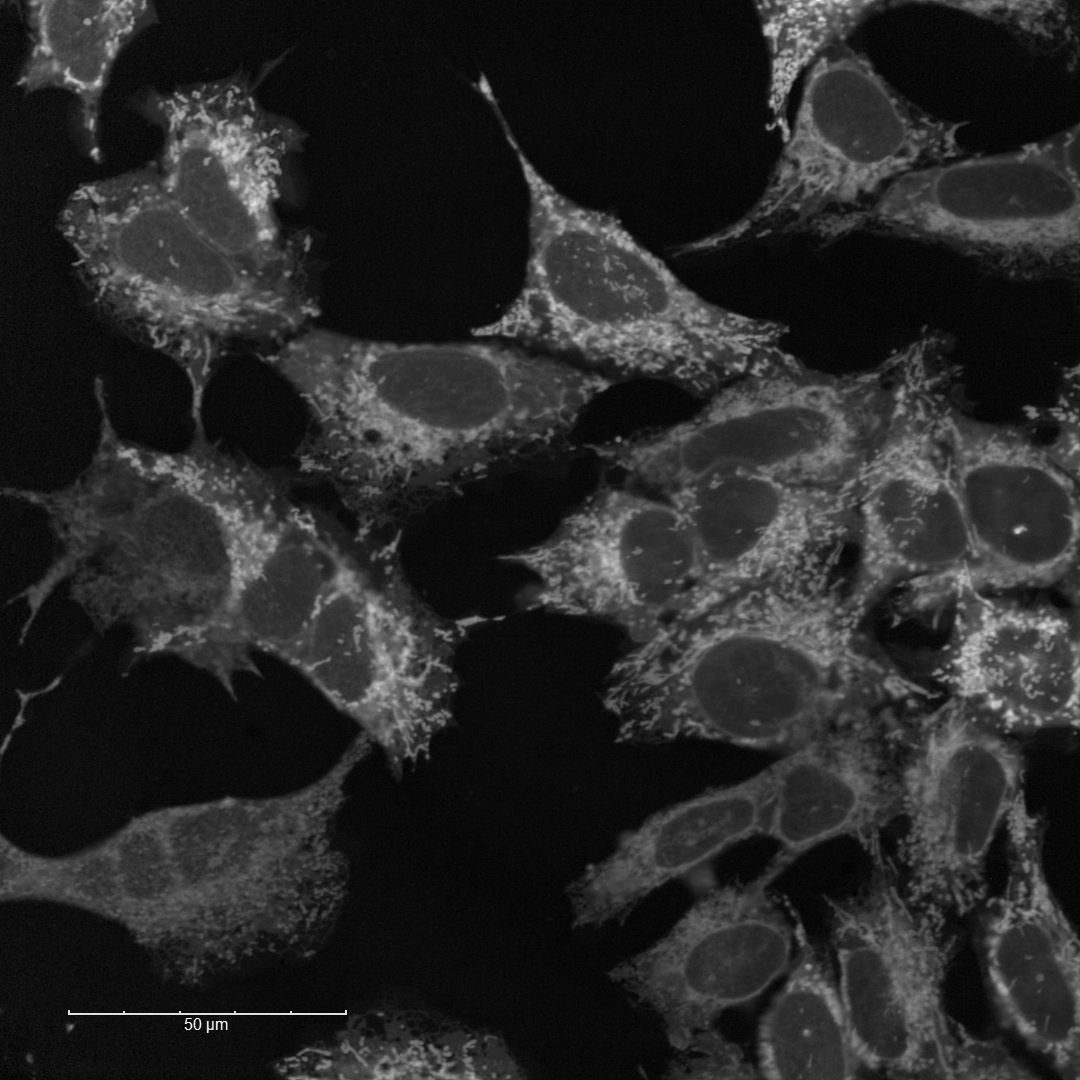

Supplement: Supplementary file 4 — Source data Fig. 2 [file 44319_2024_206_MOESM4_ESM.zip › Figure 2/2D/2D_mTurq2-BAXTMD.tif]

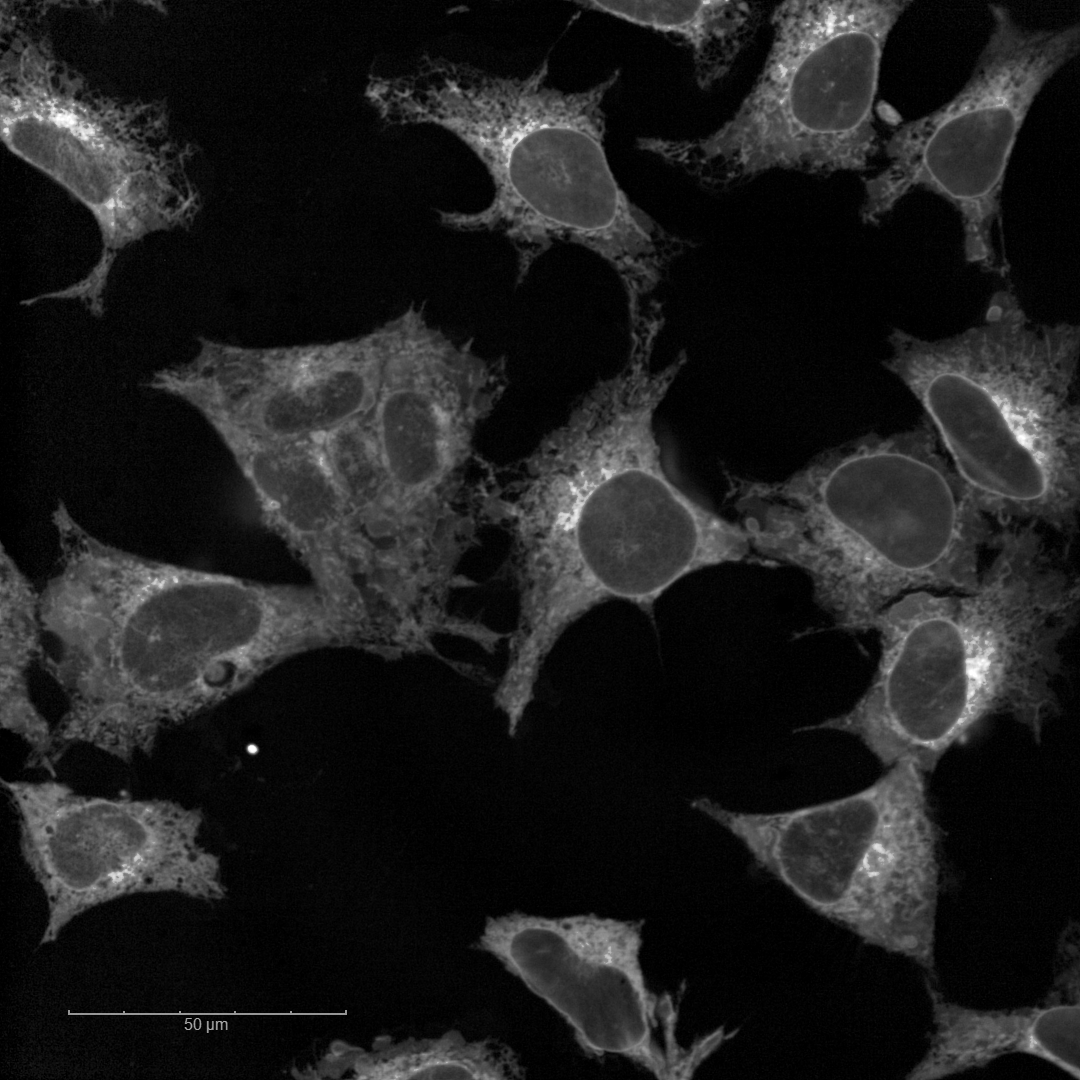

Supplement: Supplementary file 4 — Source data Fig. 2 [file 44319_2024_206_MOESM4_ESM.zip › Figure 2/2D/2D_mTurq2-BOKTMD.tif]

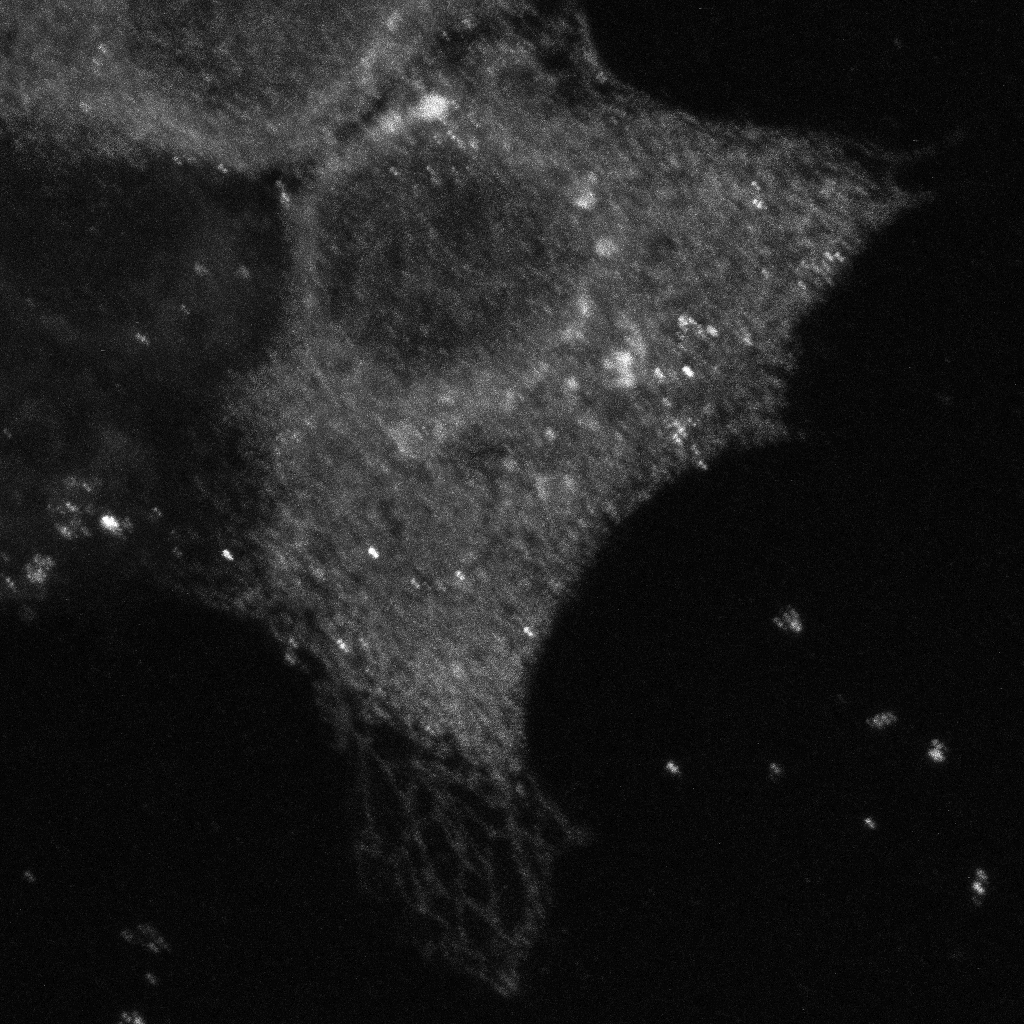

Supplement: Supplementary file 5 — Source data Fig. 3 [file 44319_2024_206_MOESM5_ESM.zip › Figure 3/3A/3A_mCarmine-ER/3A_mCarmineER.tif]

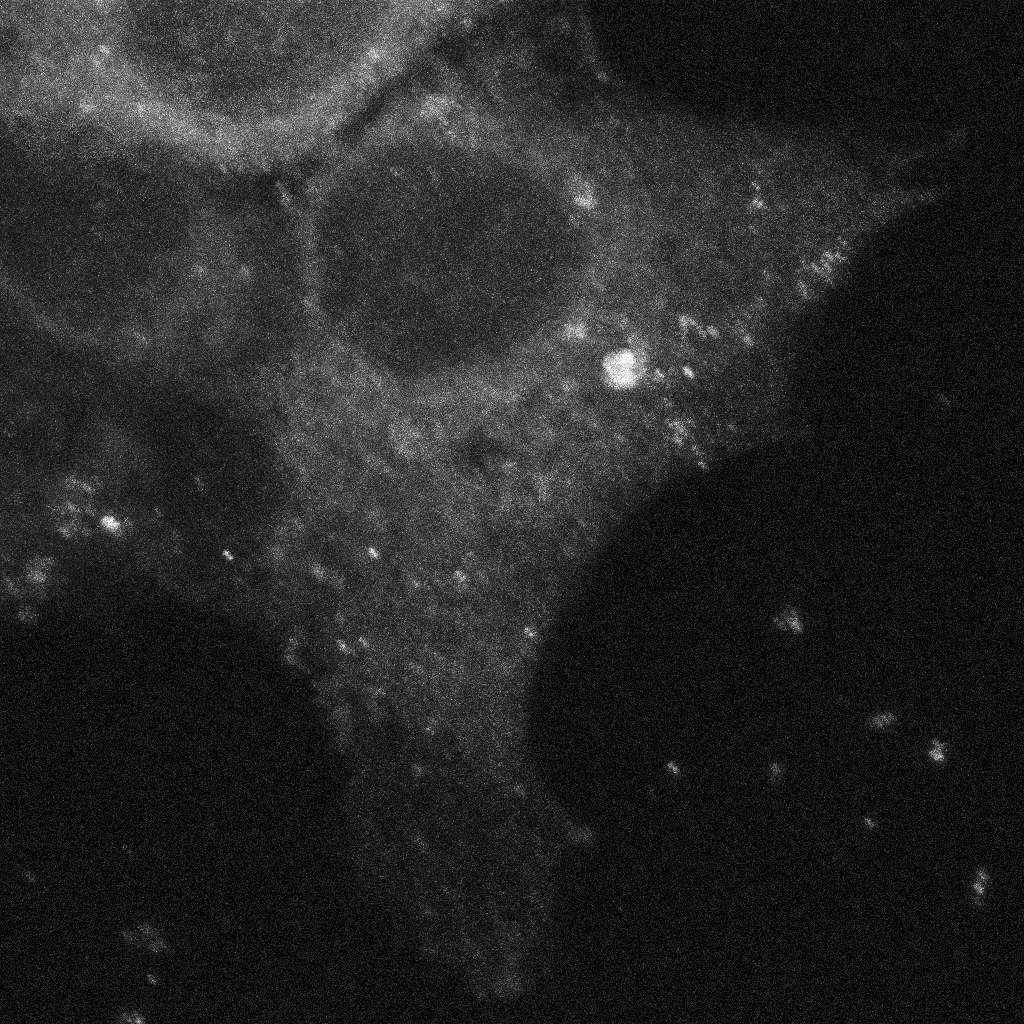

Supplement: Supplementary file 5 — Source data Fig. 3 [file 44319_2024_206_MOESM5_ESM.zip › Figure 3/3A/3A_mCarmine-ER/3A_mCitrBOKTMD.tif]

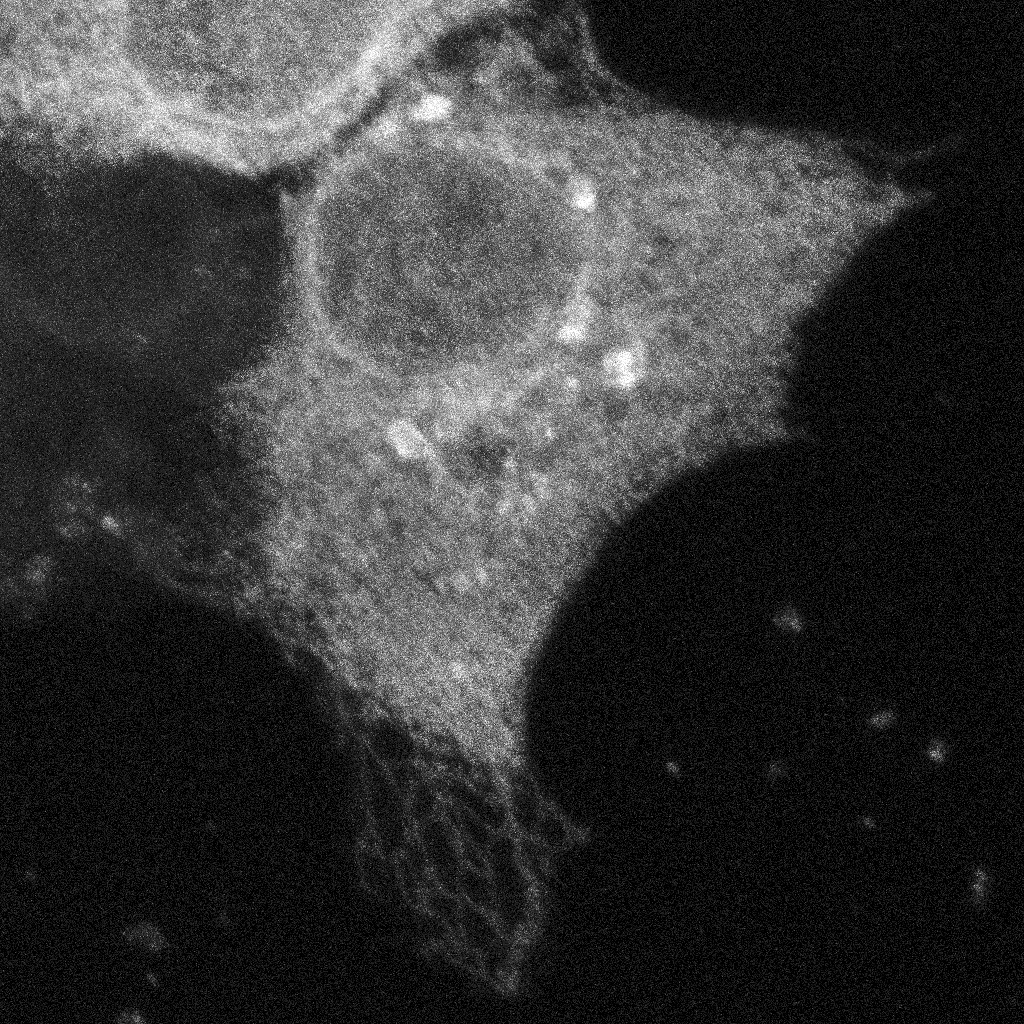

Supplement: Supplementary file 5 — Source data Fig. 3 [file 44319_2024_206_MOESM5_ESM.zip › Figure 3/3A/3A_mCarmine-ER/3A_mTurq2BCL2TMD.tif]

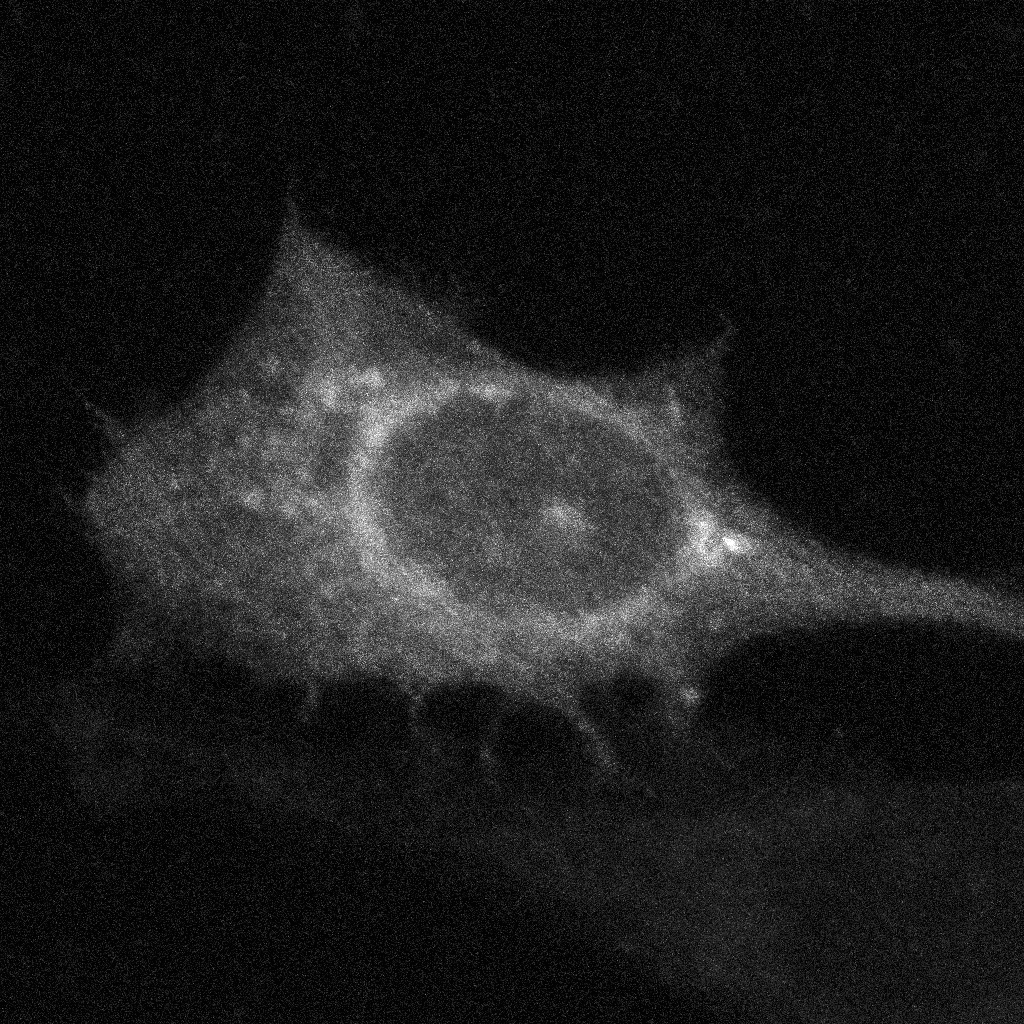

Supplement: Supplementary file 5 — Source data Fig. 3 [file 44319_2024_206_MOESM5_ESM.zip › Figure 3/3A/3A_Mitotracker/3A_mCitrBOKTMD.tif]

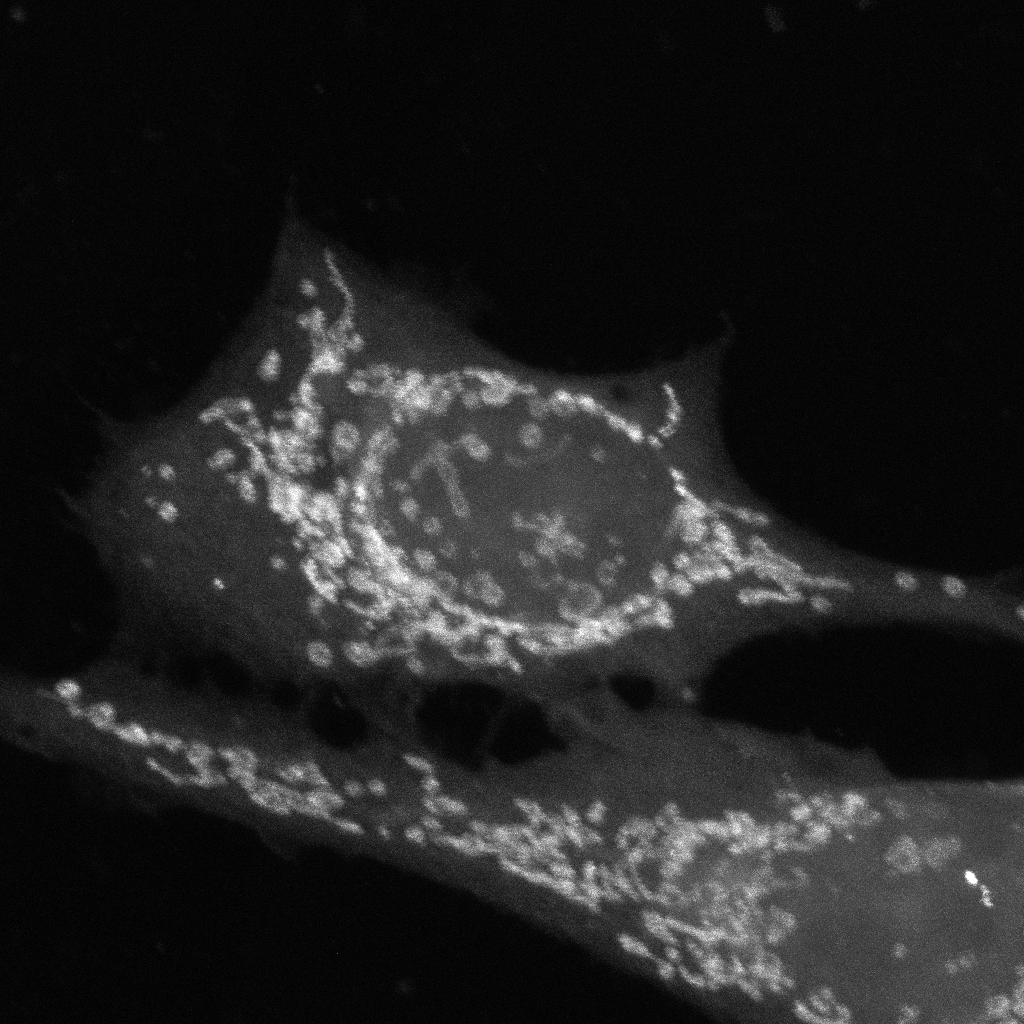

Supplement: Supplementary file 5 — Source data Fig. 3 [file 44319_2024_206_MOESM5_ESM.zip › Figure 3/3A/3A_Mitotracker/3A_Mitotracker.tif]

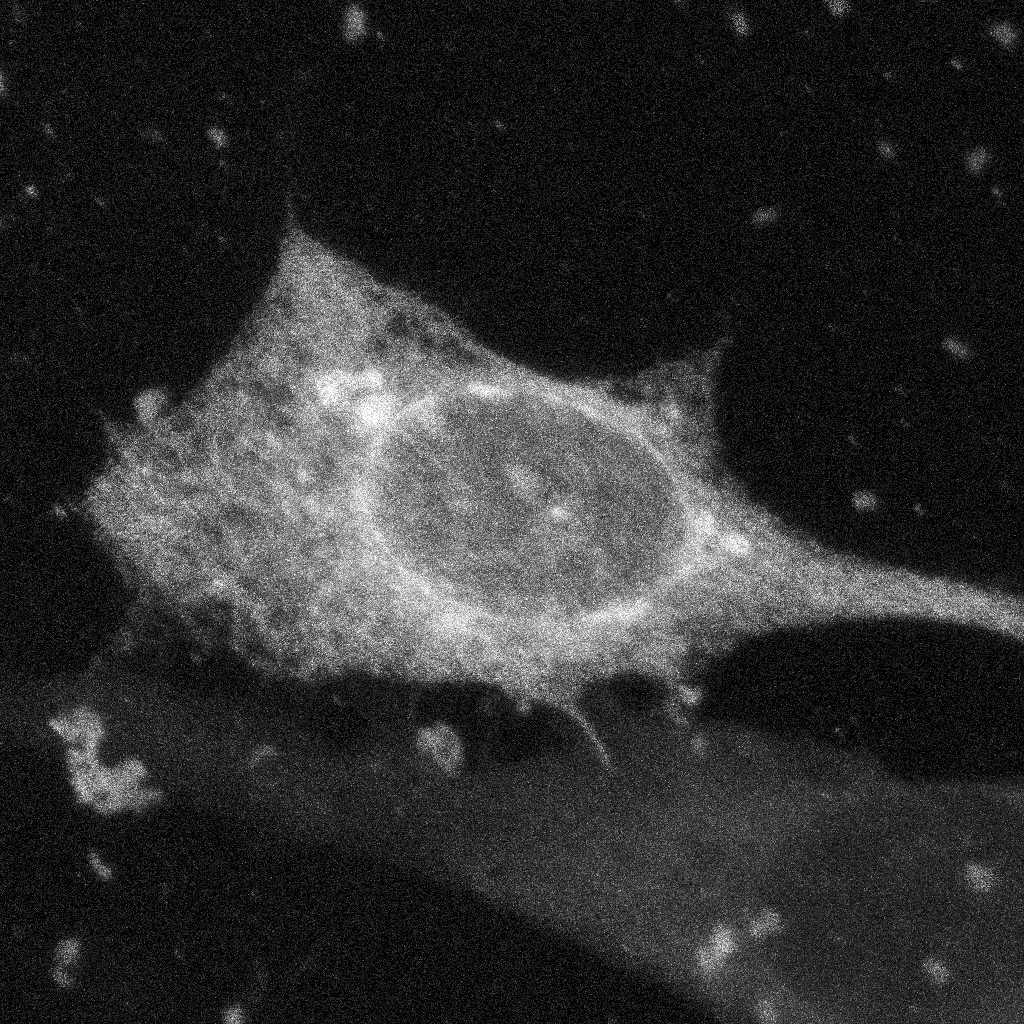

Supplement: Supplementary file 5 — Source data Fig. 3 [file 44319_2024_206_MOESM5_ESM.zip › Figure 3/3A/3A_Mitotracker/3A_mTurq2BCL2TMD.tif]

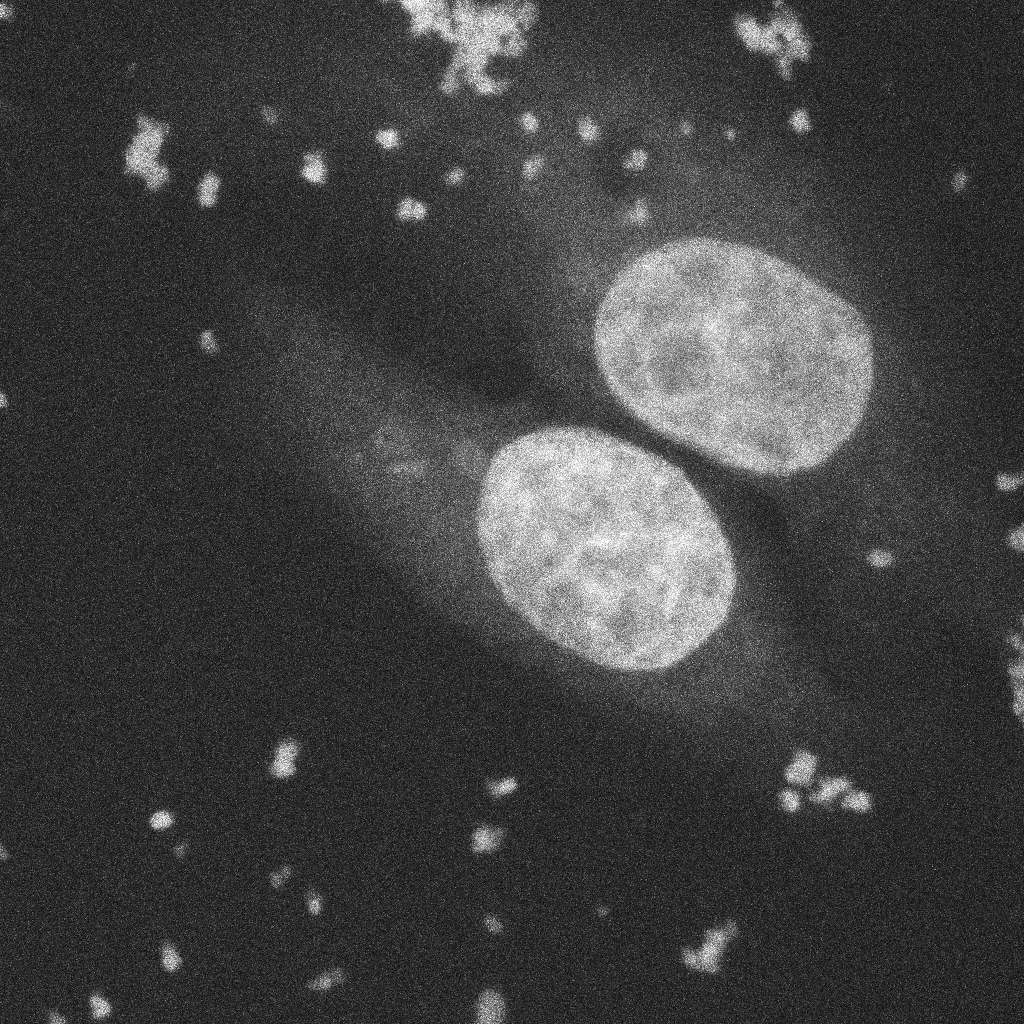

Supplement: Supplementary file 6 — Source data Fig. 4 [file 44319_2024_206_MOESM6_ESM.zip › Figure 4/4C/BAK/4C_BAK_DAPI.tif]

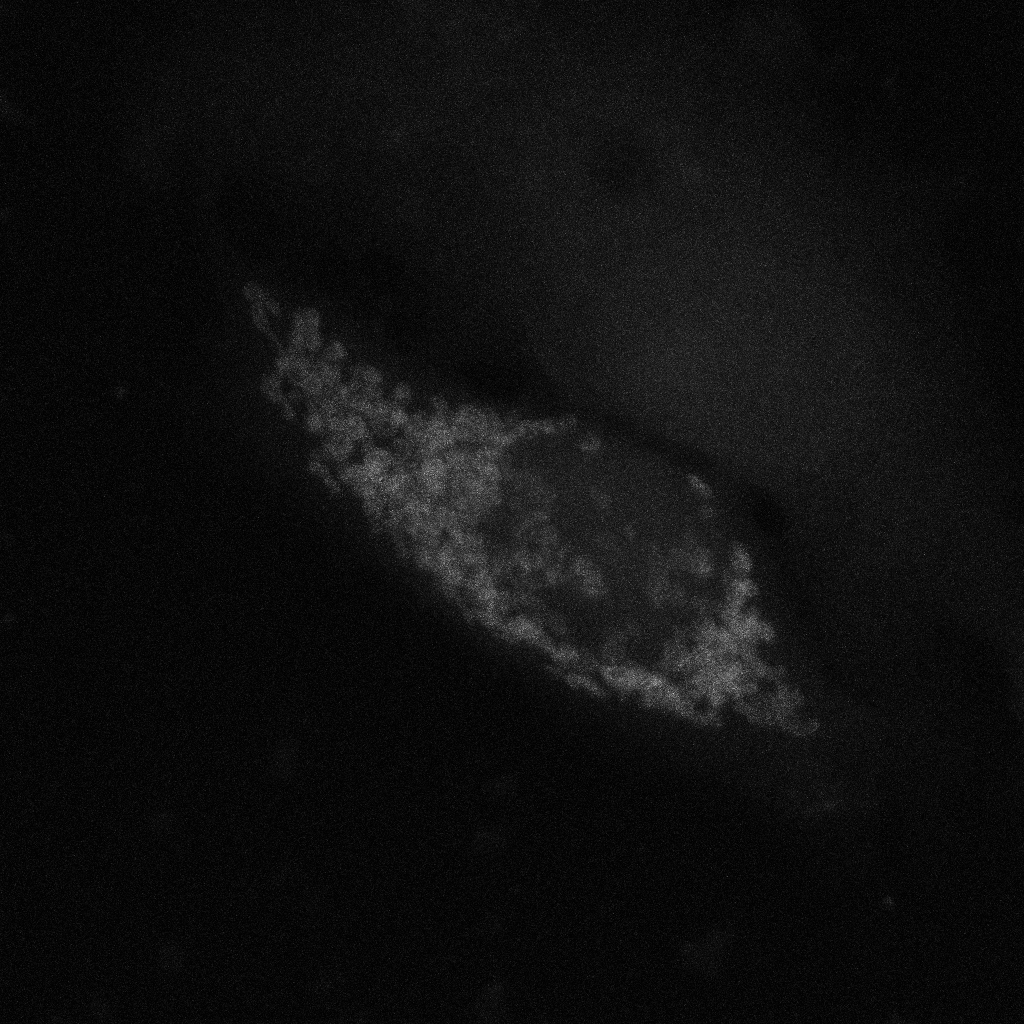

Supplement: Supplementary file 6 — Source data Fig. 4 [file 44319_2024_206_MOESM6_ESM.zip › Figure 4/4C/BAK/4C_BAK_EGFP.tif]

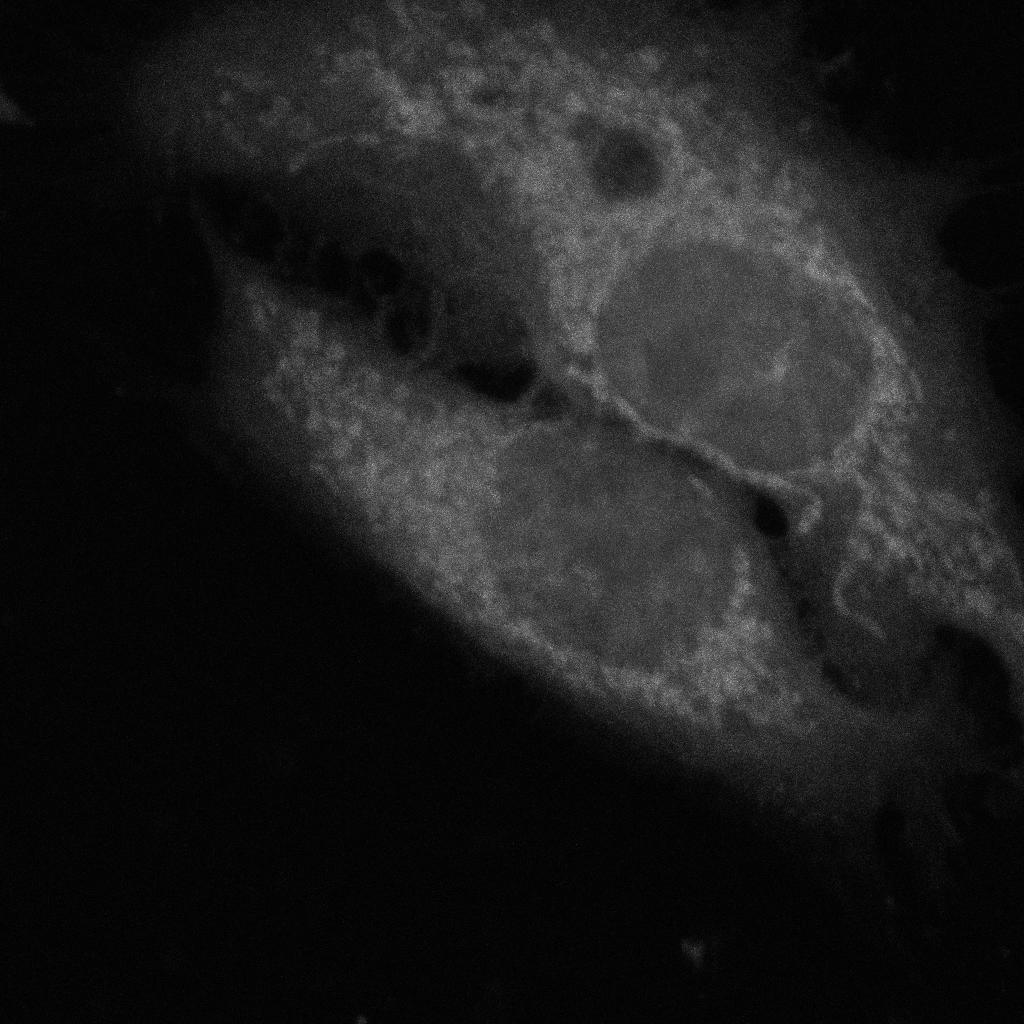

Supplement: Supplementary file 6 — Source data Fig. 4 [file 44319_2024_206_MOESM6_ESM.zip › Figure 4/4C/BAK/4C_BAK_Mito.tif]

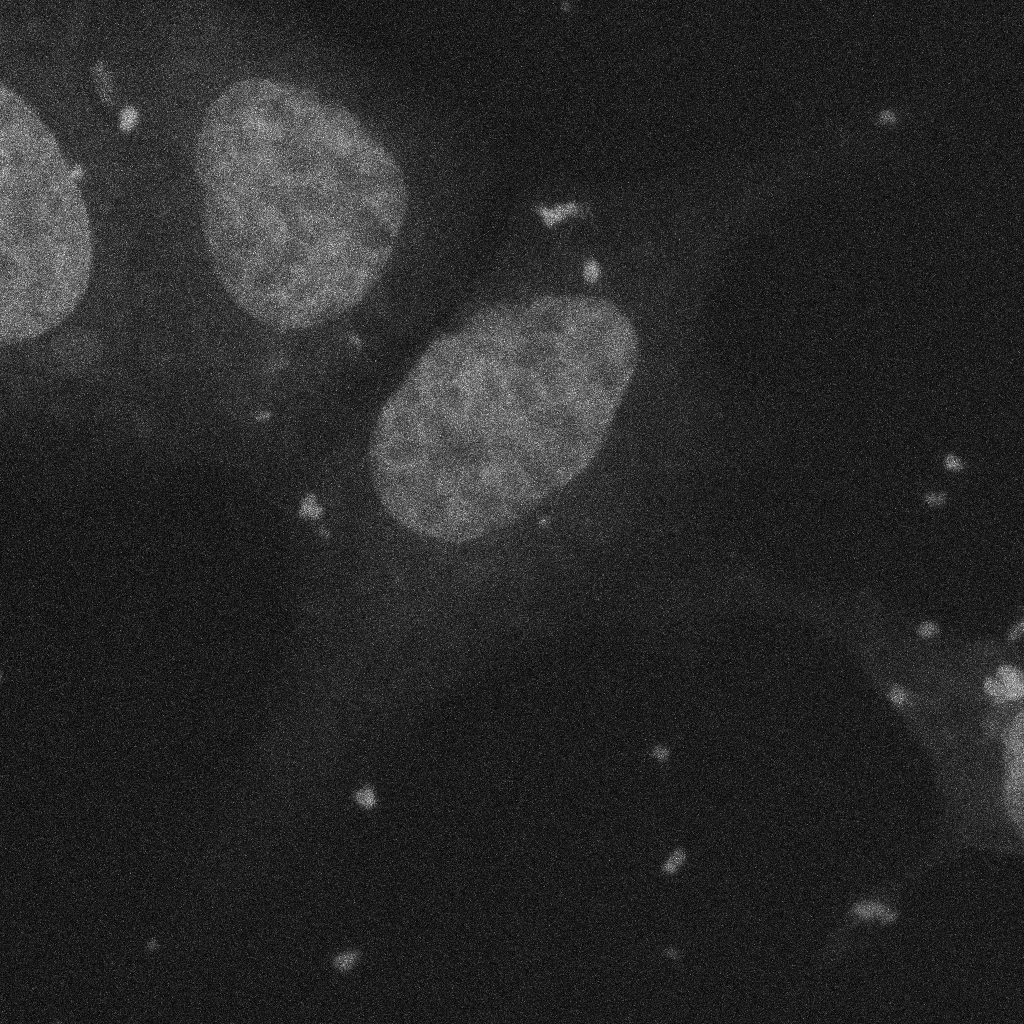

Supplement: Supplementary file 6 — Source data Fig. 4 [file 44319_2024_206_MOESM6_ESM.zip › Figure 4/4C/BAX/4C_BAX_DAPI.tif]

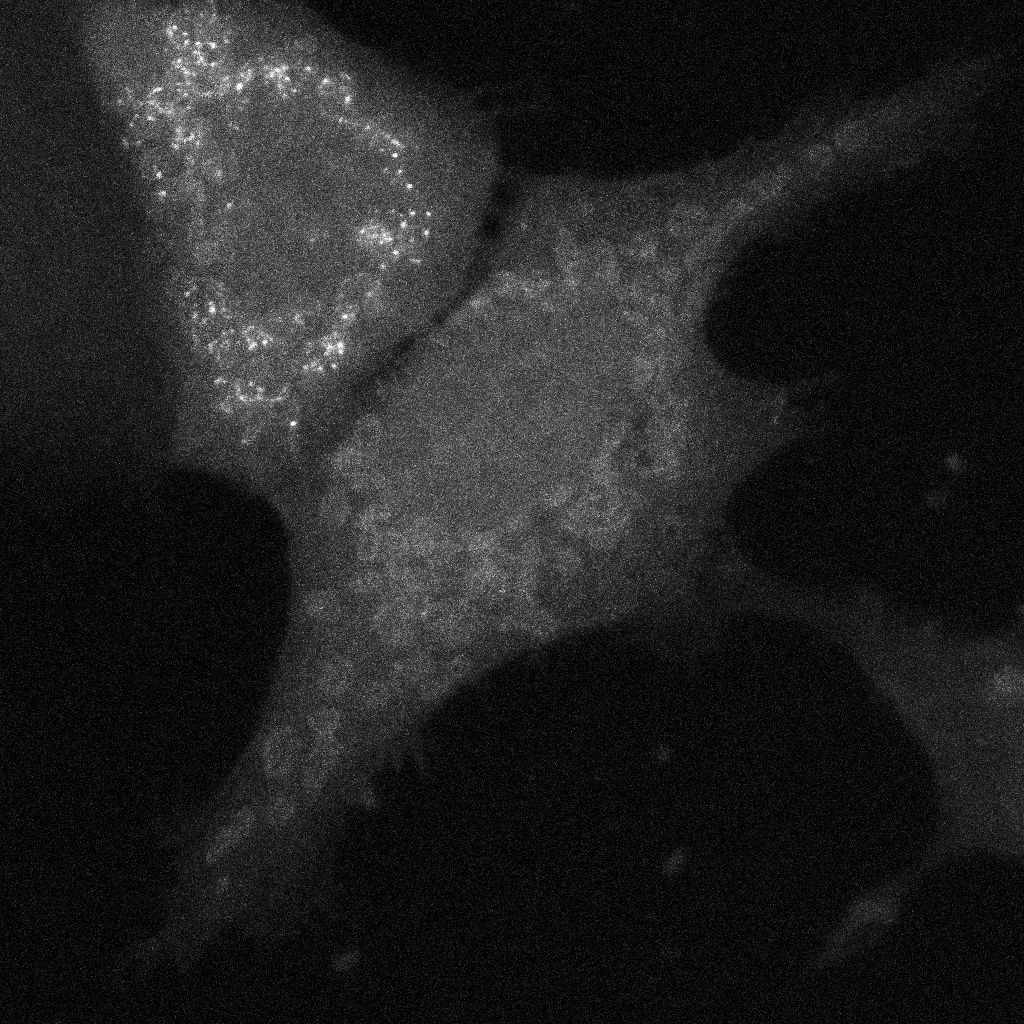

Supplement: Supplementary file 6 — Source data Fig. 4 [file 44319_2024_206_MOESM6_ESM.zip › Figure 4/4C/BAX/4C_BAX_EGFP.tif]

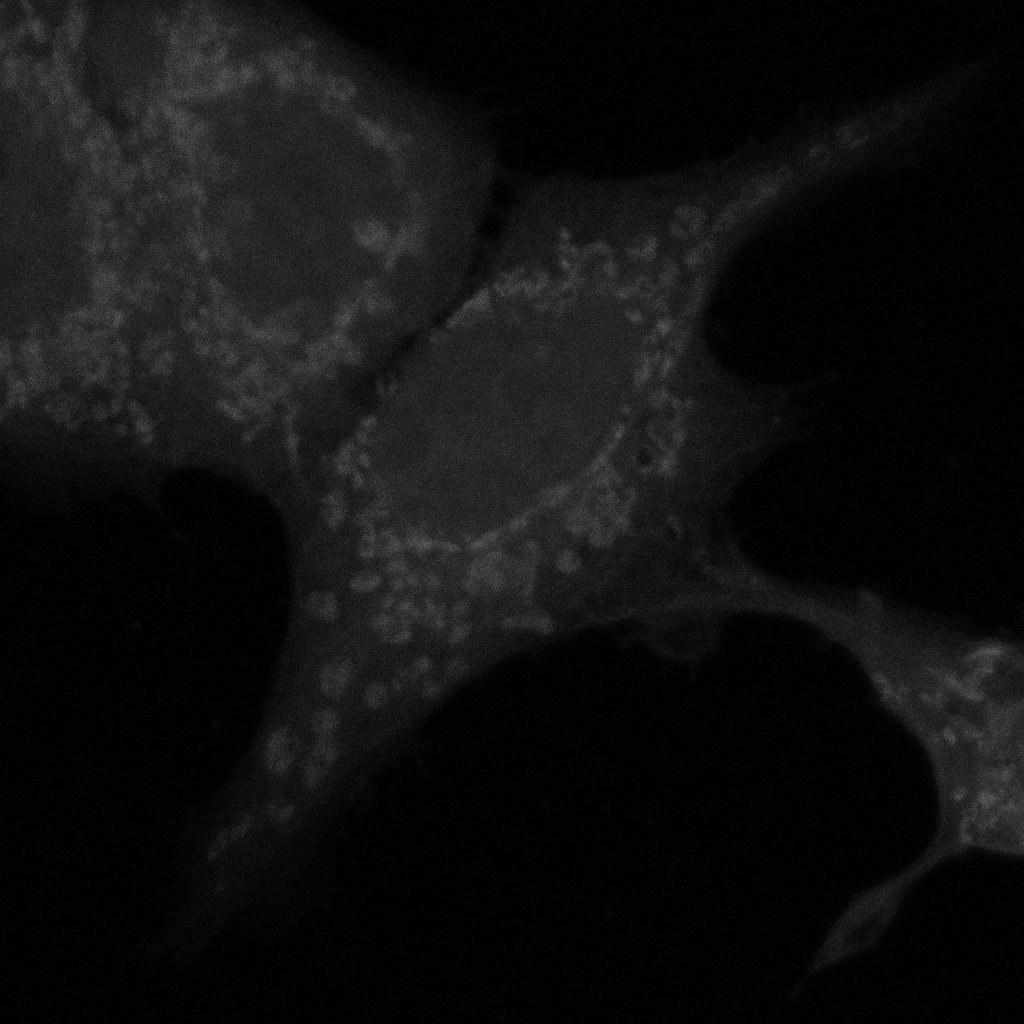

Supplement: Supplementary file 6 — Source data Fig. 4 [file 44319_2024_206_MOESM6_ESM.zip › Figure 4/4C/BAX/4C_BAX_Mito.tif]

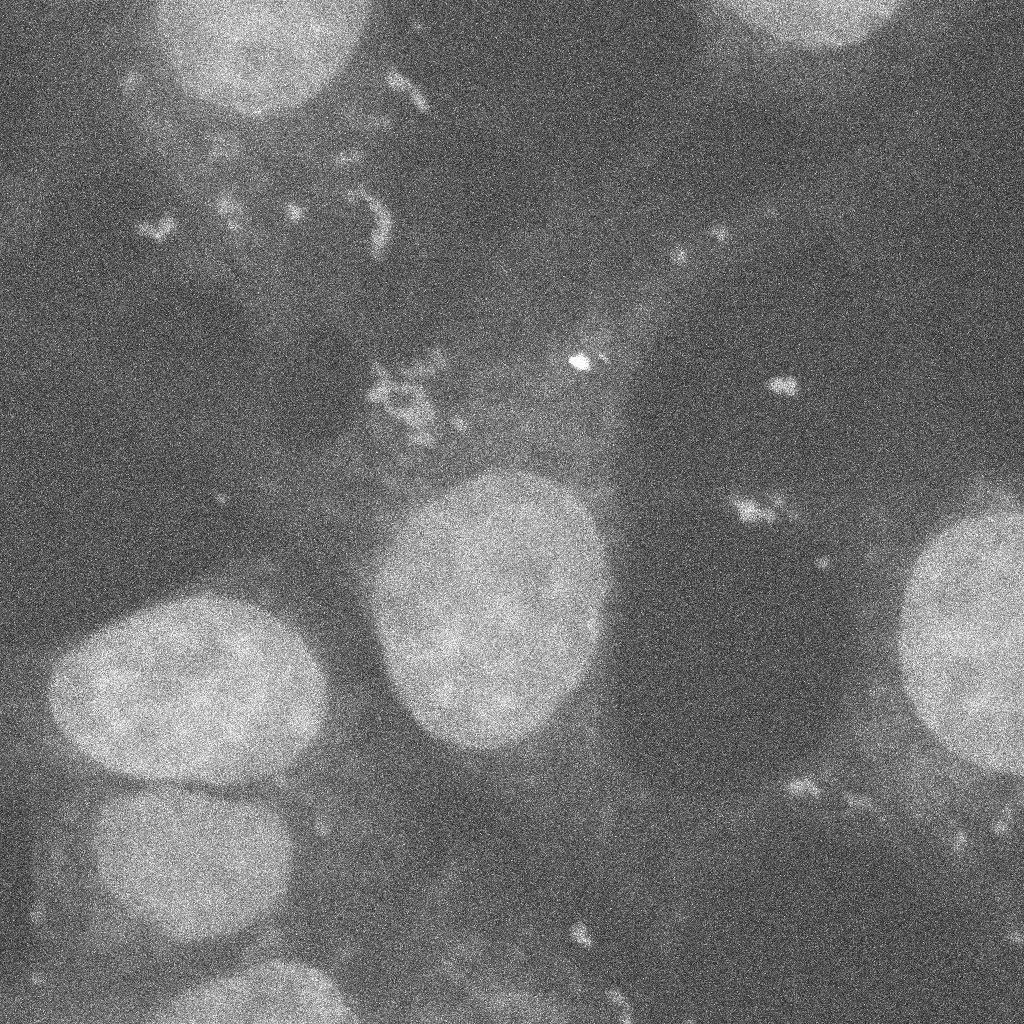

Supplement: Supplementary file 6 — Source data Fig. 4 [file 44319_2024_206_MOESM6_ESM.zip › Figure 4/4C/BOK/4C_BOK_DAPI.tif]

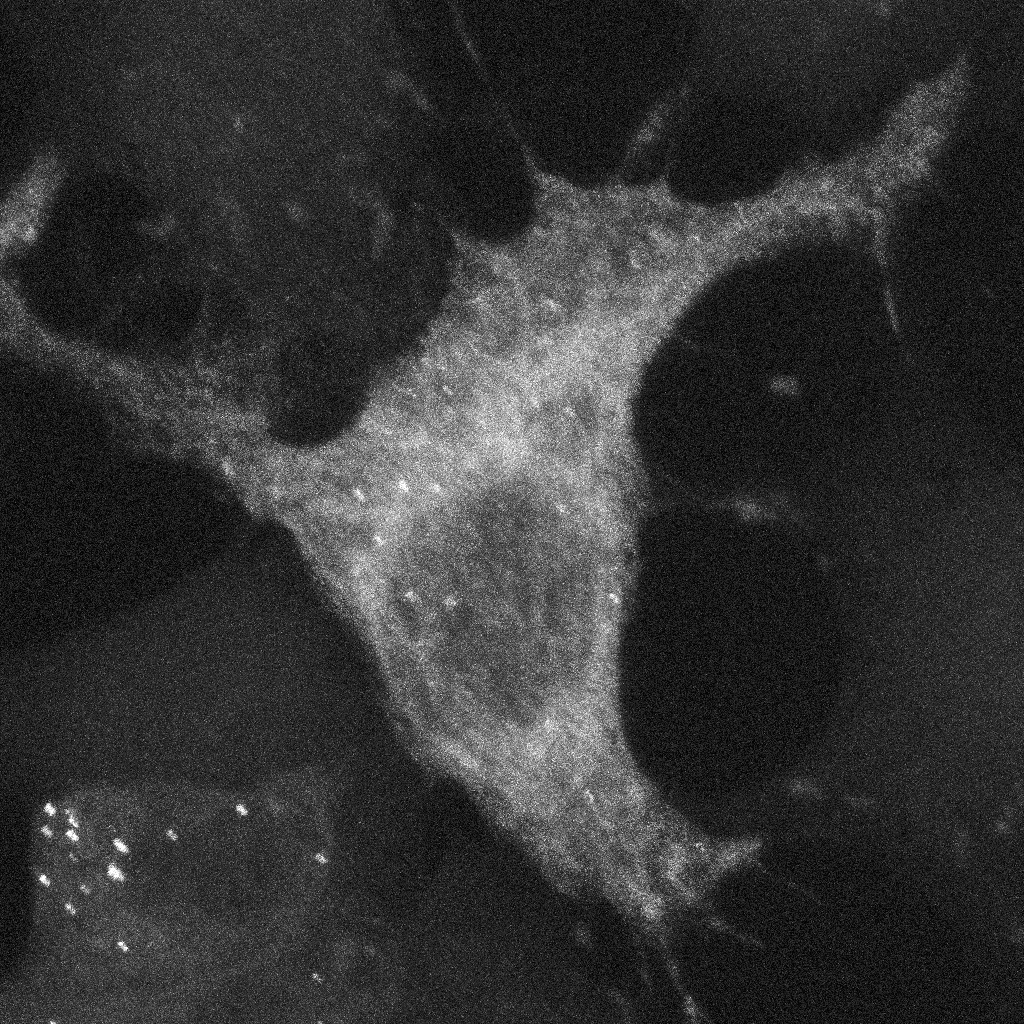

Supplement: Supplementary file 6 — Source data Fig. 4 [file 44319_2024_206_MOESM6_ESM.zip › Figure 4/4C/BOK/4C_BOK_EGFP.tif]

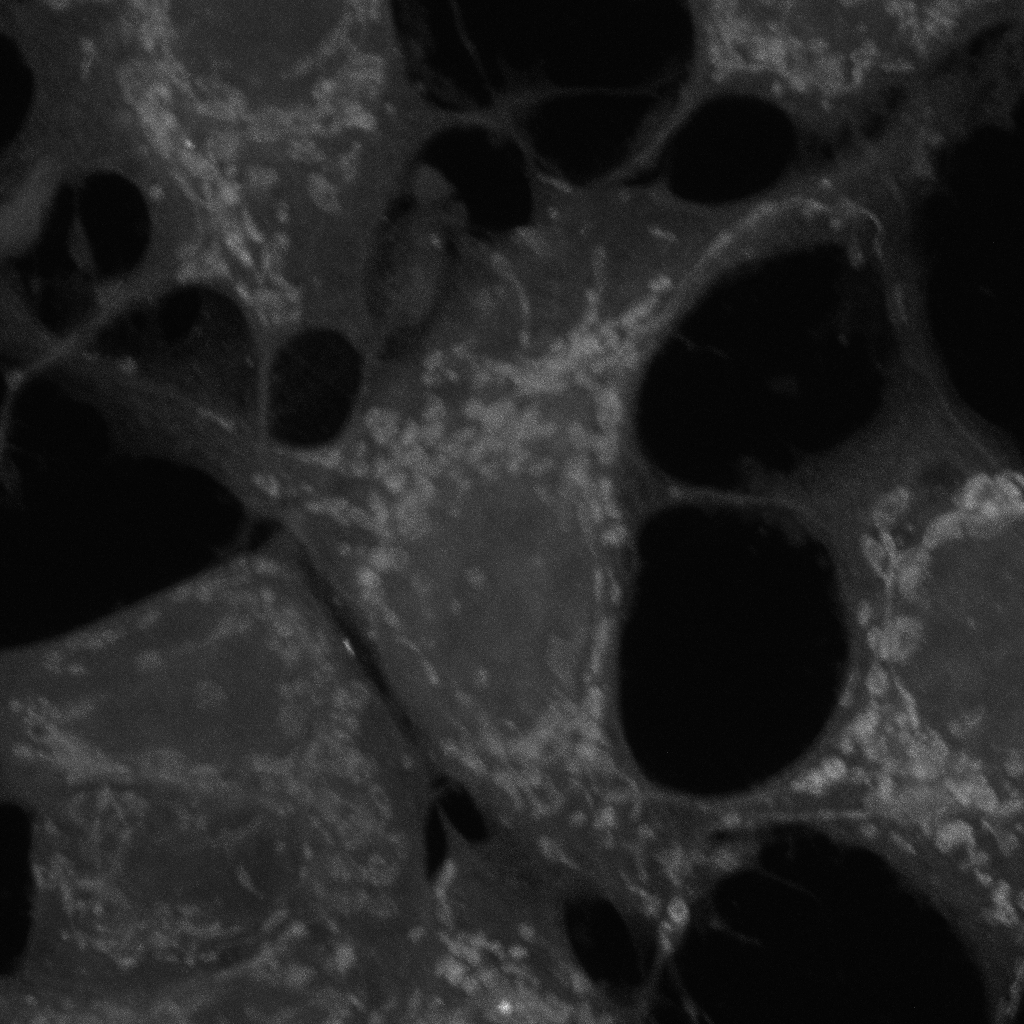

Supplement: Supplementary file 6 — Source data Fig. 4 [file 44319_2024_206_MOESM6_ESM.zip › Figure 4/4C/BOK/4C_BOK_Mito.tif]

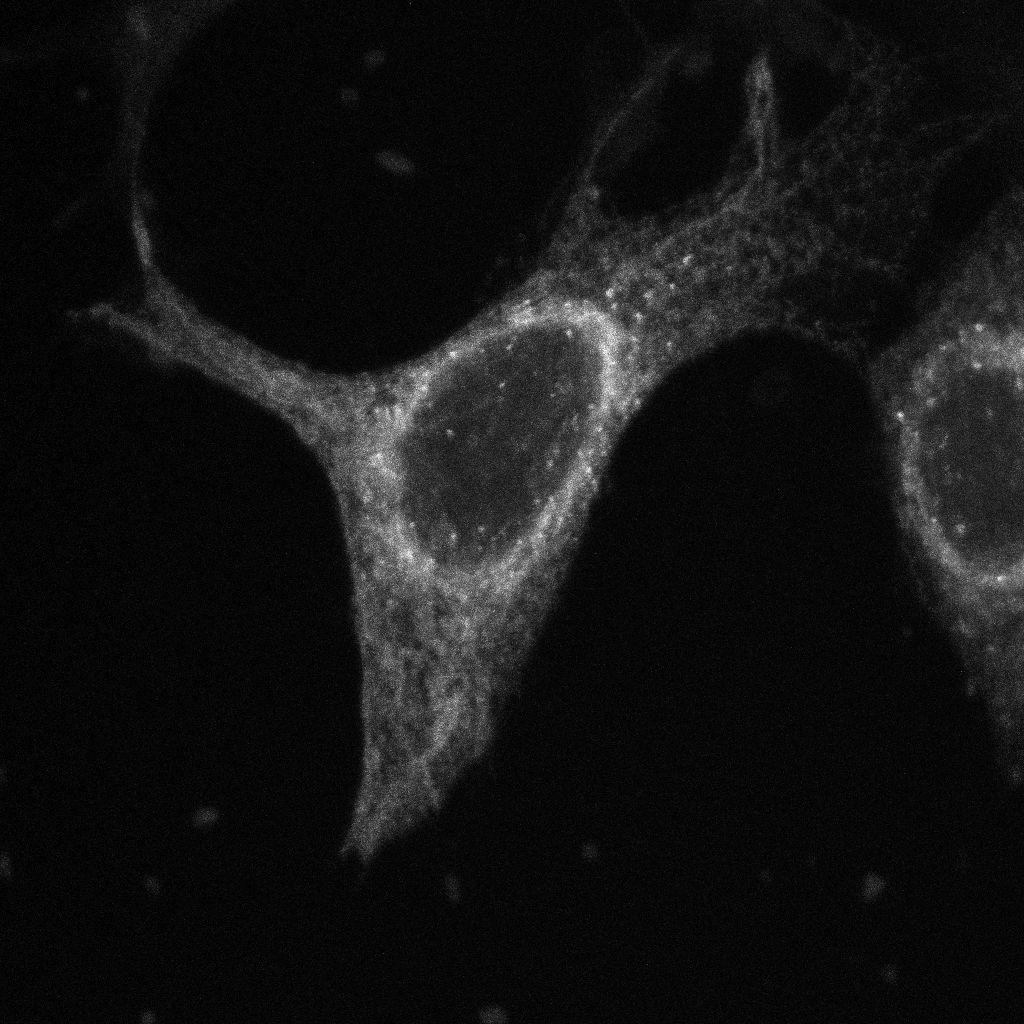

Supplement: Supplementary file 6 — Source data Fig. 4 [file 44319_2024_206_MOESM6_ESM.zip › Figure 4/4D/BAK/4D_BAK_BCL2.tif]

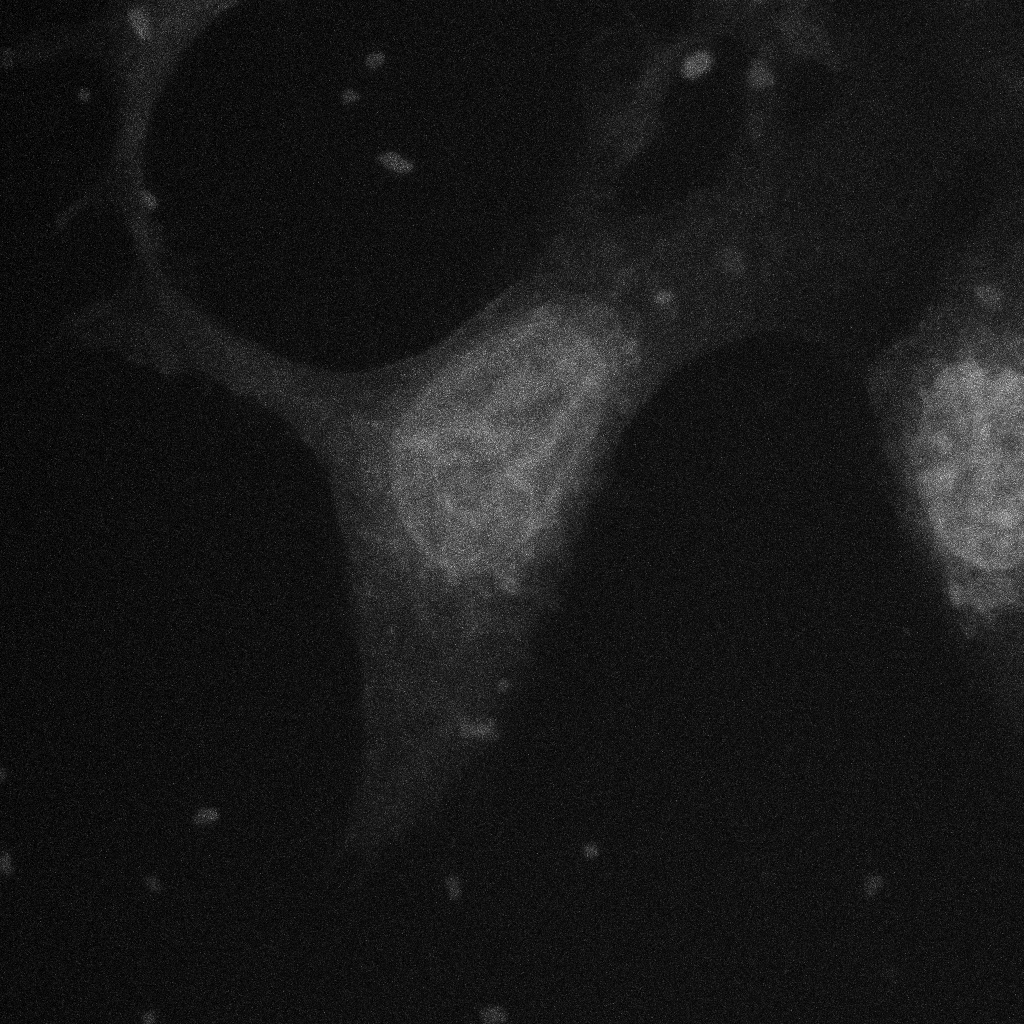

Supplement: Supplementary file 6 — Source data Fig. 4 [file 44319_2024_206_MOESM6_ESM.zip › Figure 4/4D/BAK/4D_BAK_DAPI.tif]

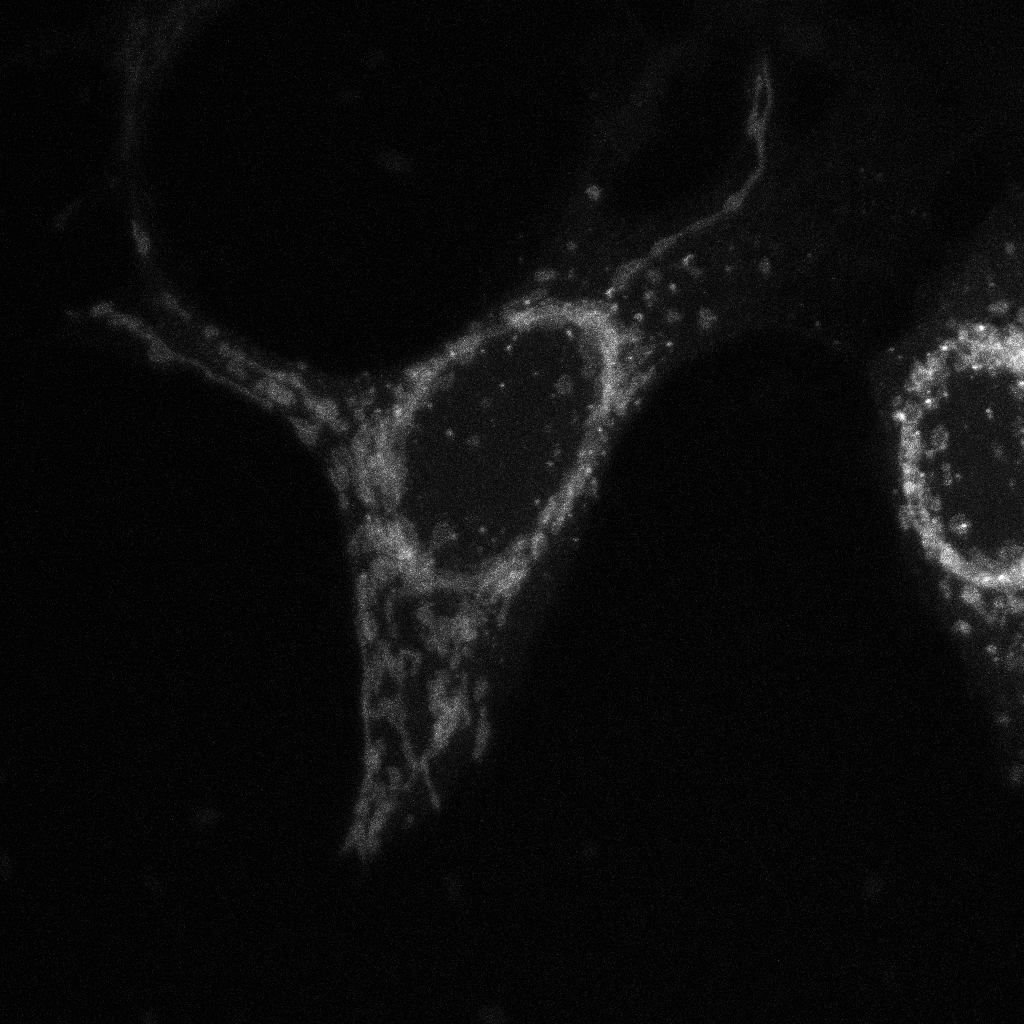

Supplement: Supplementary file 6 — Source data Fig. 4 [file 44319_2024_206_MOESM6_ESM.zip › Figure 4/4D/BAK/4D_BAK_EGFP.tif]

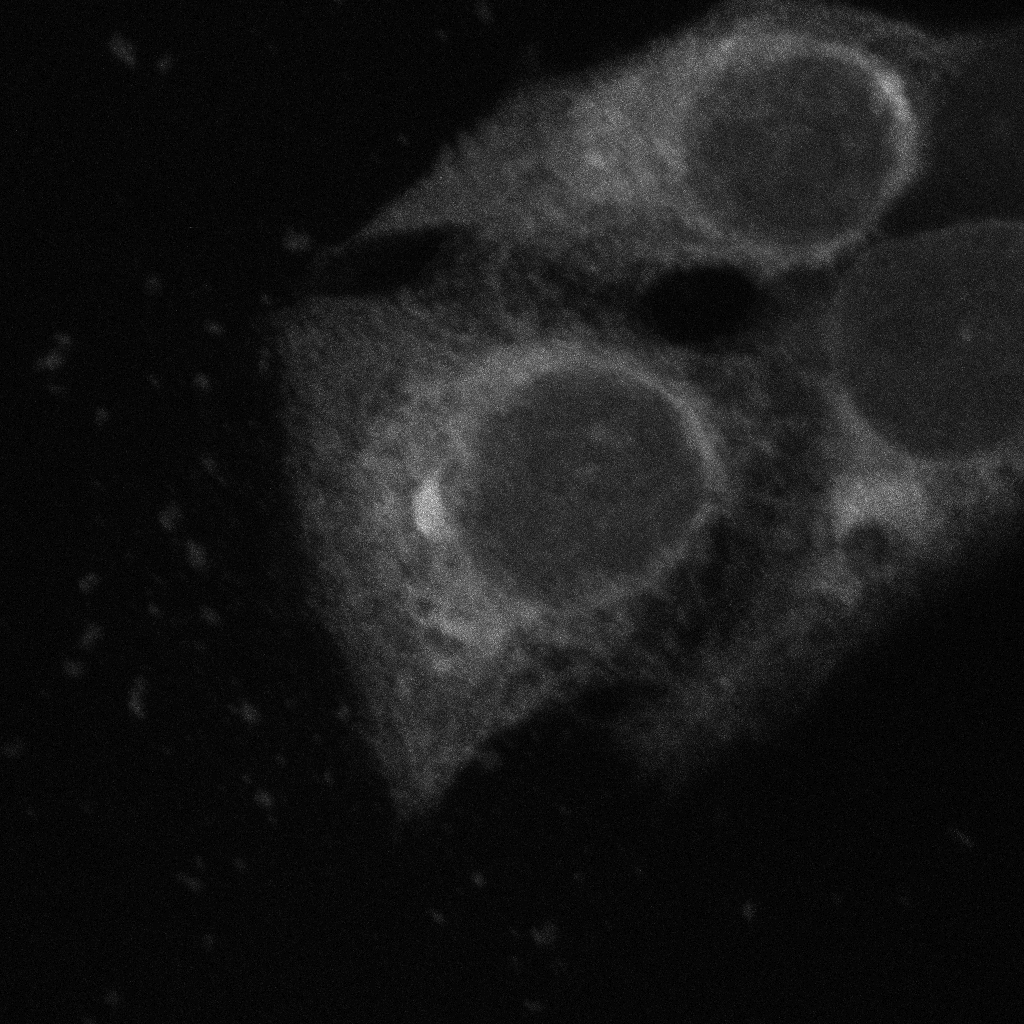

Supplement: Supplementary file 6 — Source data Fig. 4 [file 44319_2024_206_MOESM6_ESM.zip › Figure 4/4D/BAX/4D_BAX_BCL2.tif]

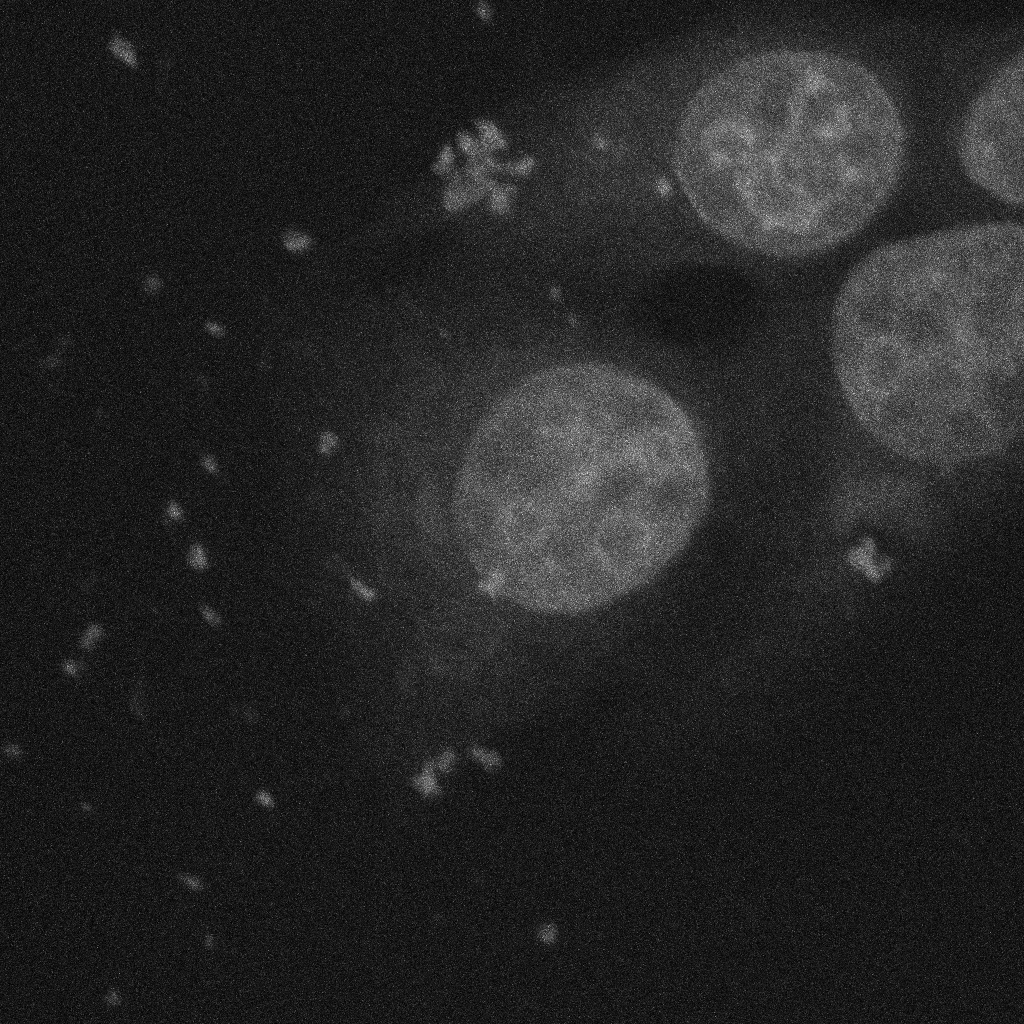

Supplement: Supplementary file 6 — Source data Fig. 4 [file 44319_2024_206_MOESM6_ESM.zip › Figure 4/4D/BAX/4D_BAX_DAPI.tif]

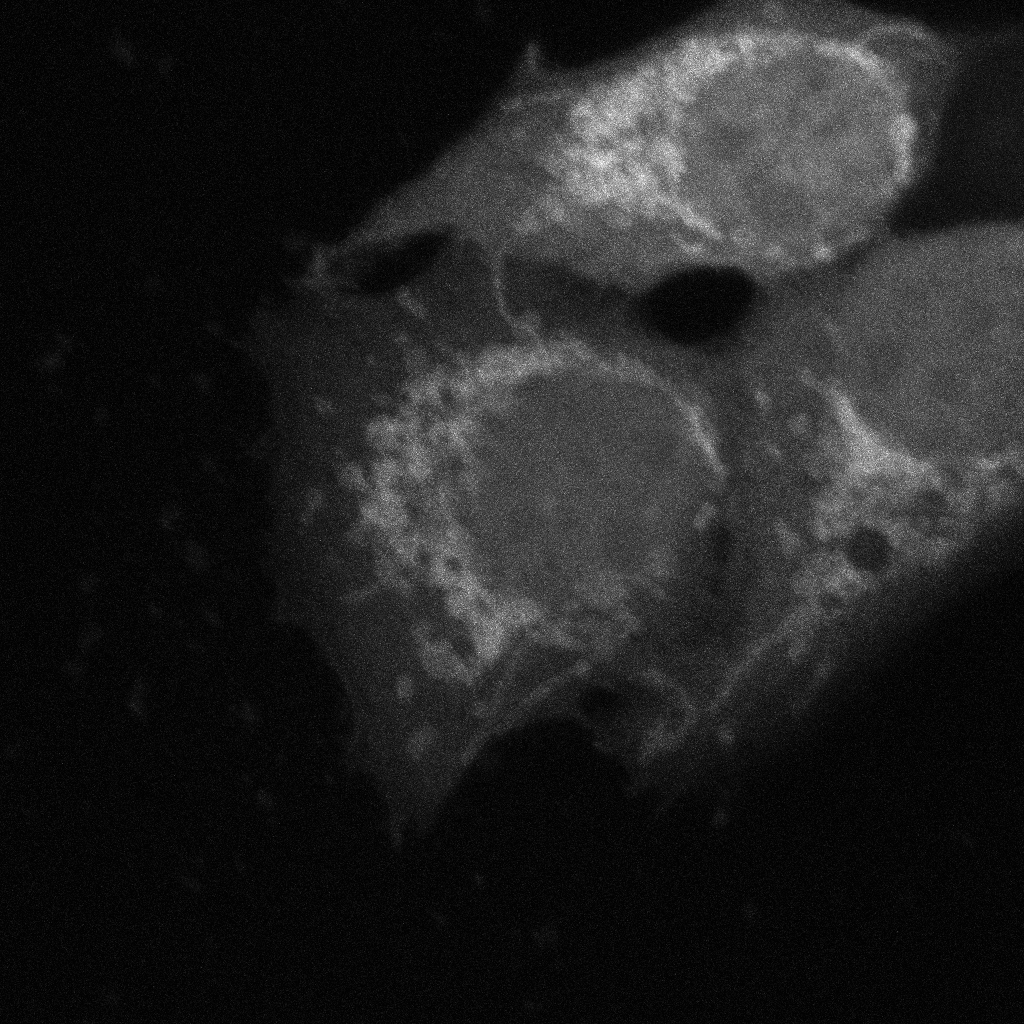

Supplement: Supplementary file 6 — Source data Fig. 4 [file 44319_2024_206_MOESM6_ESM.zip › Figure 4/4D/BAX/4D_BAX_EGFP.tif]

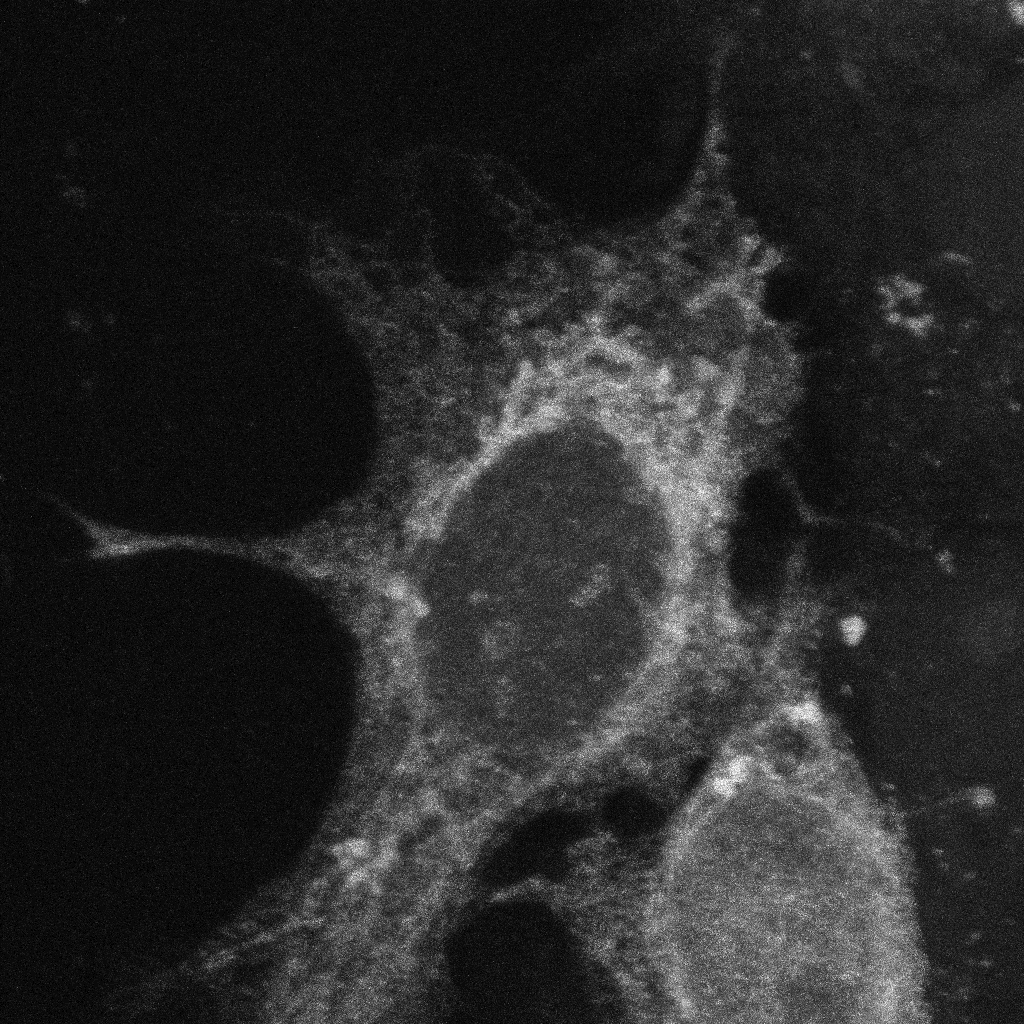

Supplement: Supplementary file 6 — Source data Fig. 4 [file 44319_2024_206_MOESM6_ESM.zip › Figure 4/4D/BOK/4D_BOK_BCL2.tif]

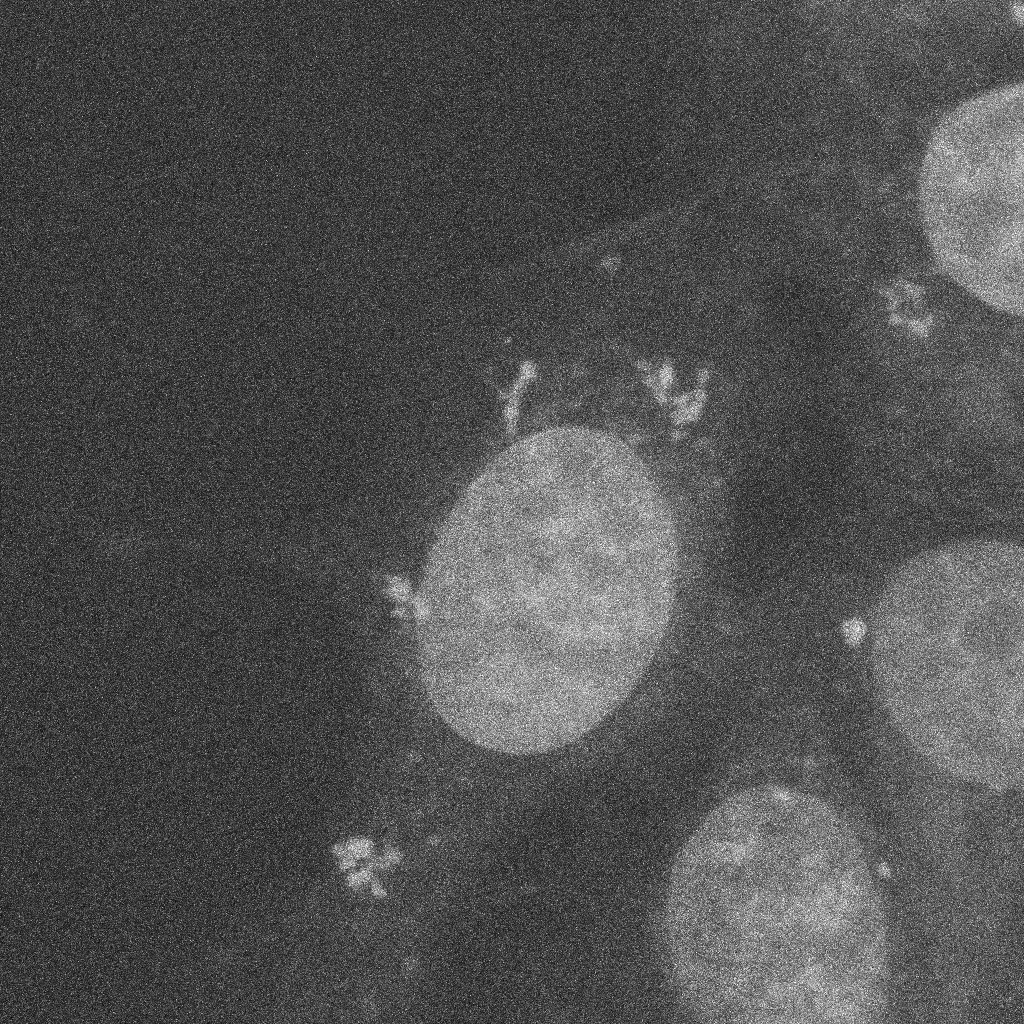

Supplement: Supplementary file 6 — Source data Fig. 4 [file 44319_2024_206_MOESM6_ESM.zip › Figure 4/4D/BOK/4D_BOK_DAPI.tif]

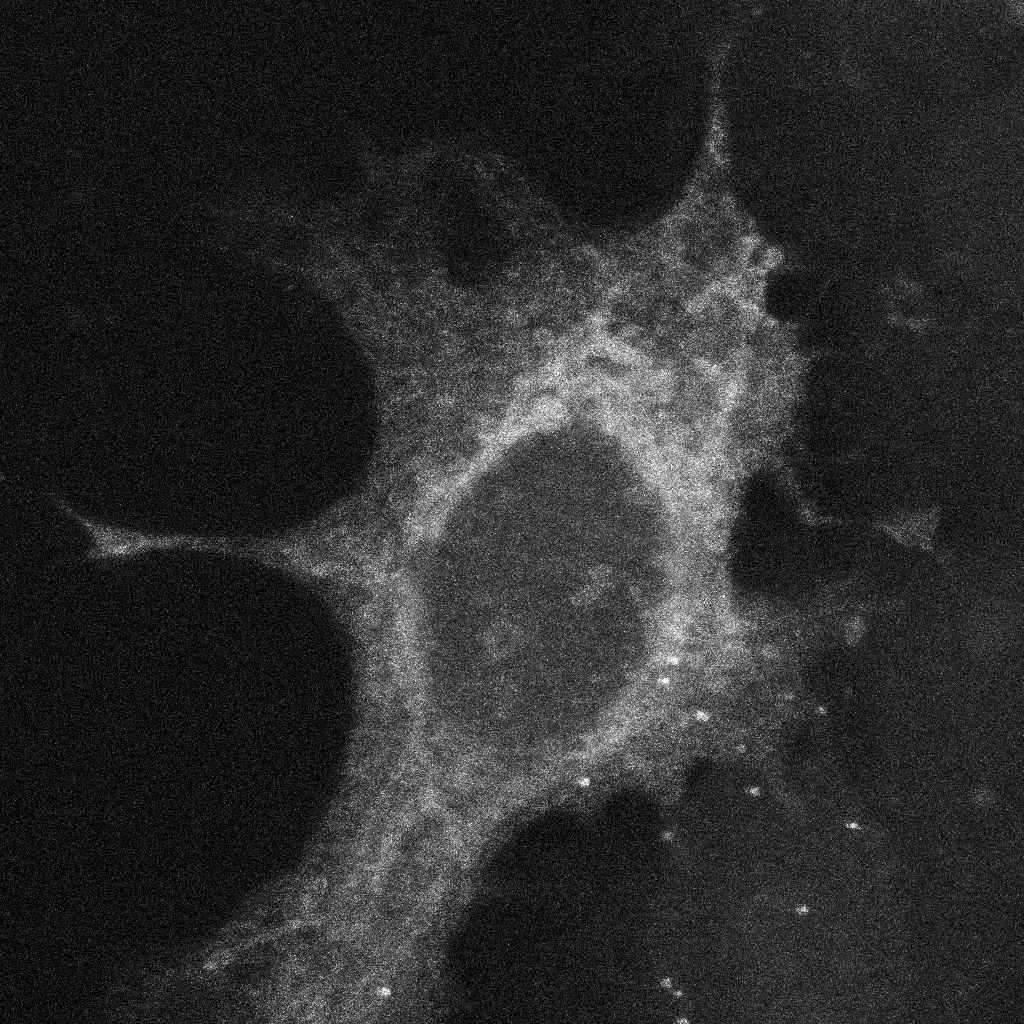

Supplement: Supplementary file 6 — Source data Fig. 4 [file 44319_2024_206_MOESM6_ESM.zip › Figure 4/4D/BOK/4D_BOK_EGFP.tif]

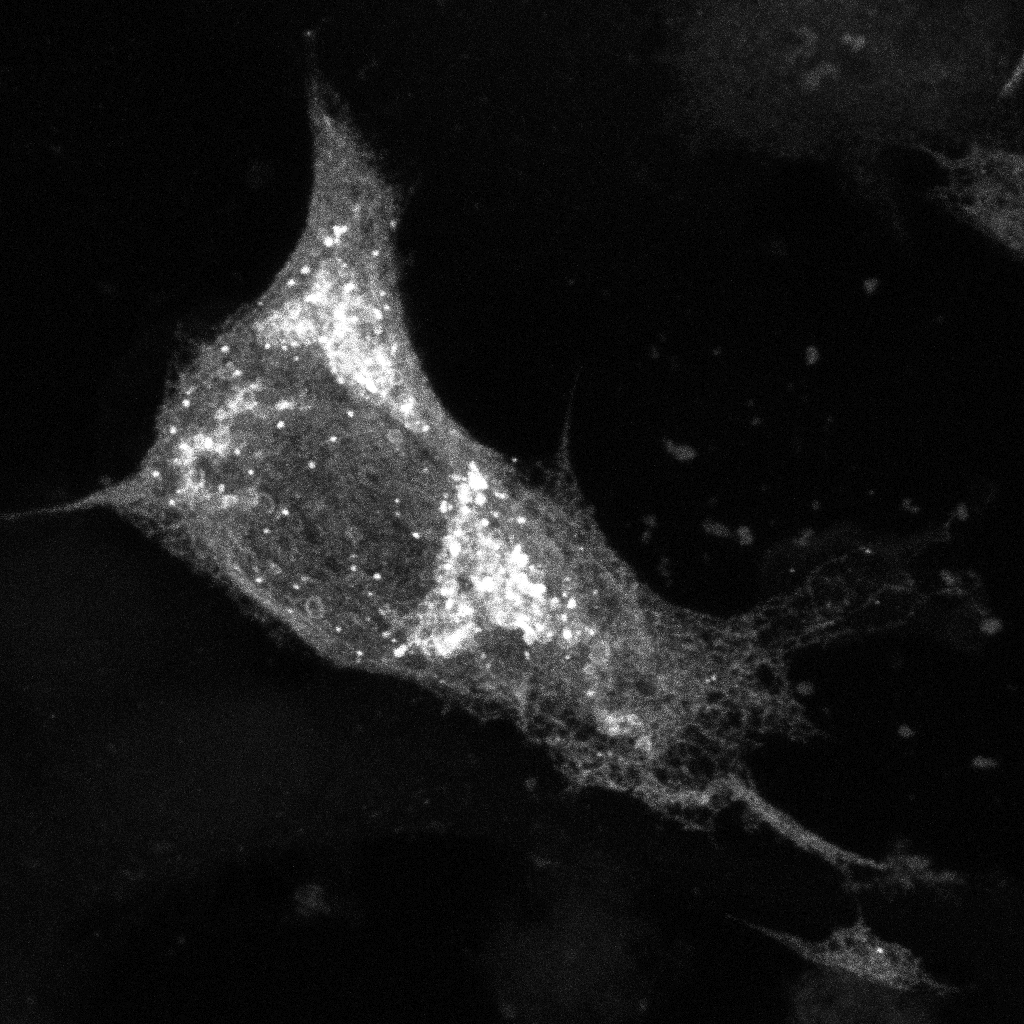

Supplement: Supplementary file 6 — Source data Fig. 4 [file 44319_2024_206_MOESM6_ESM.zip › Figure 4/4E/BAK/4E_BAK_BCL2.tif]

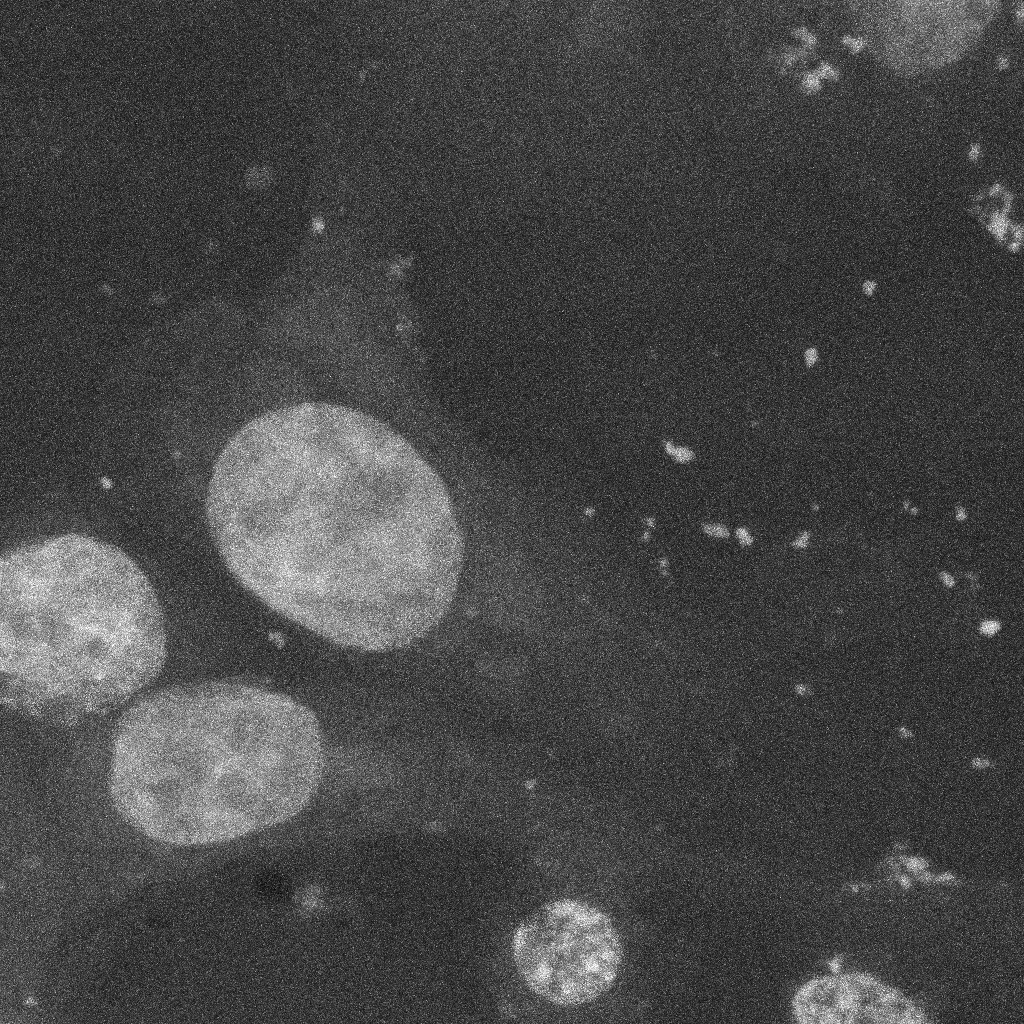

Supplement: Supplementary file 6 — Source data Fig. 4 [file 44319_2024_206_MOESM6_ESM.zip › Figure 4/4E/BAK/4E_BAK_DAPI.tif]

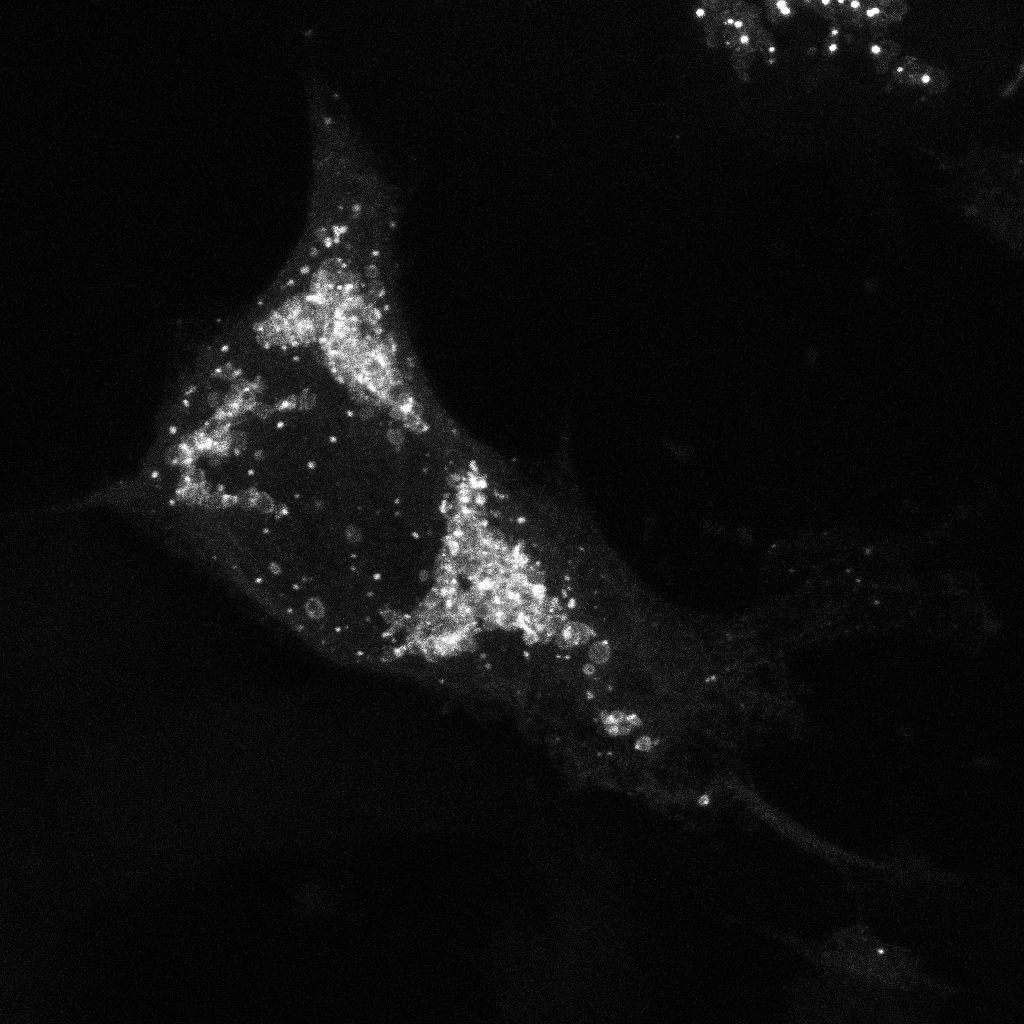

Supplement: Supplementary file 6 — Source data Fig. 4 [file 44319_2024_206_MOESM6_ESM.zip › Figure 4/4E/BAK/4E_BAK_EGFP.tif]

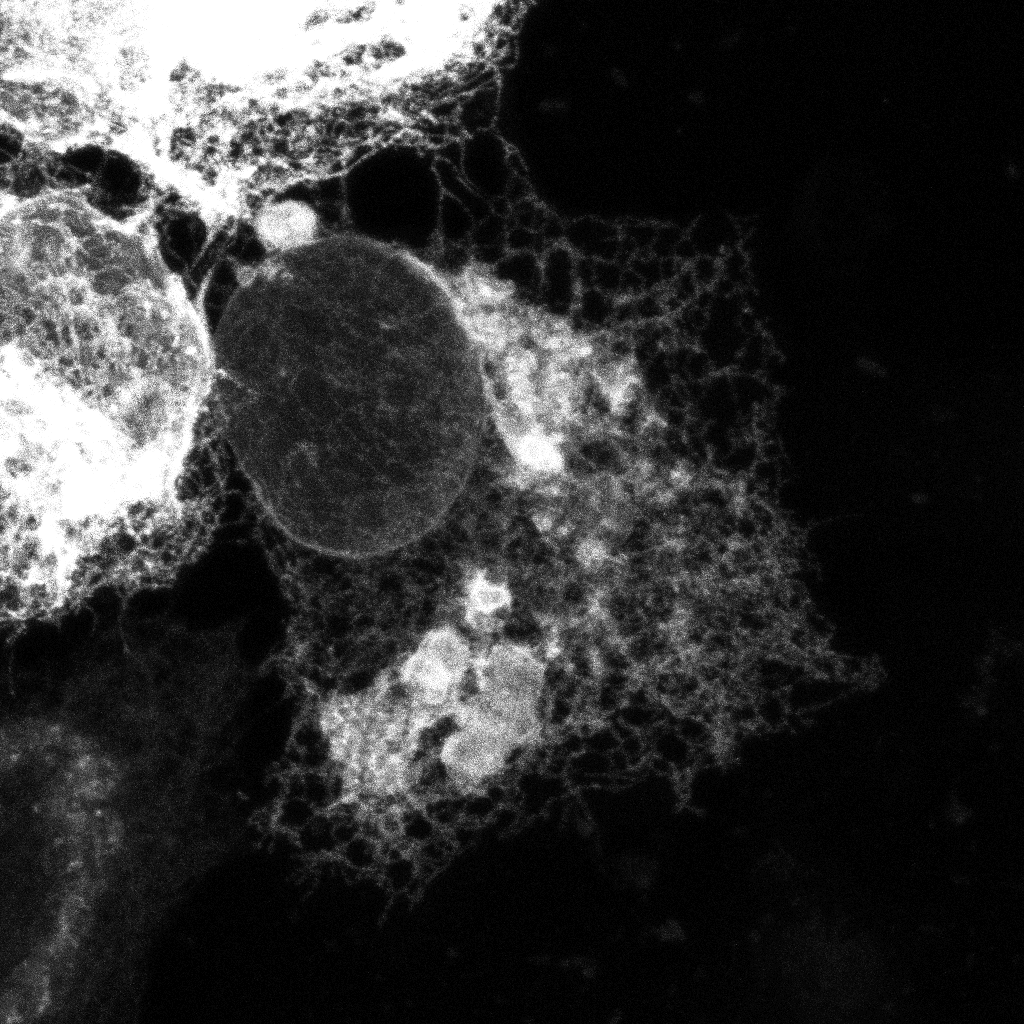

Supplement: Supplementary file 6 — Source data Fig. 4 [file 44319_2024_206_MOESM6_ESM.zip › Figure 4/4E/BAX/4E_BAX_BCL2.tif]

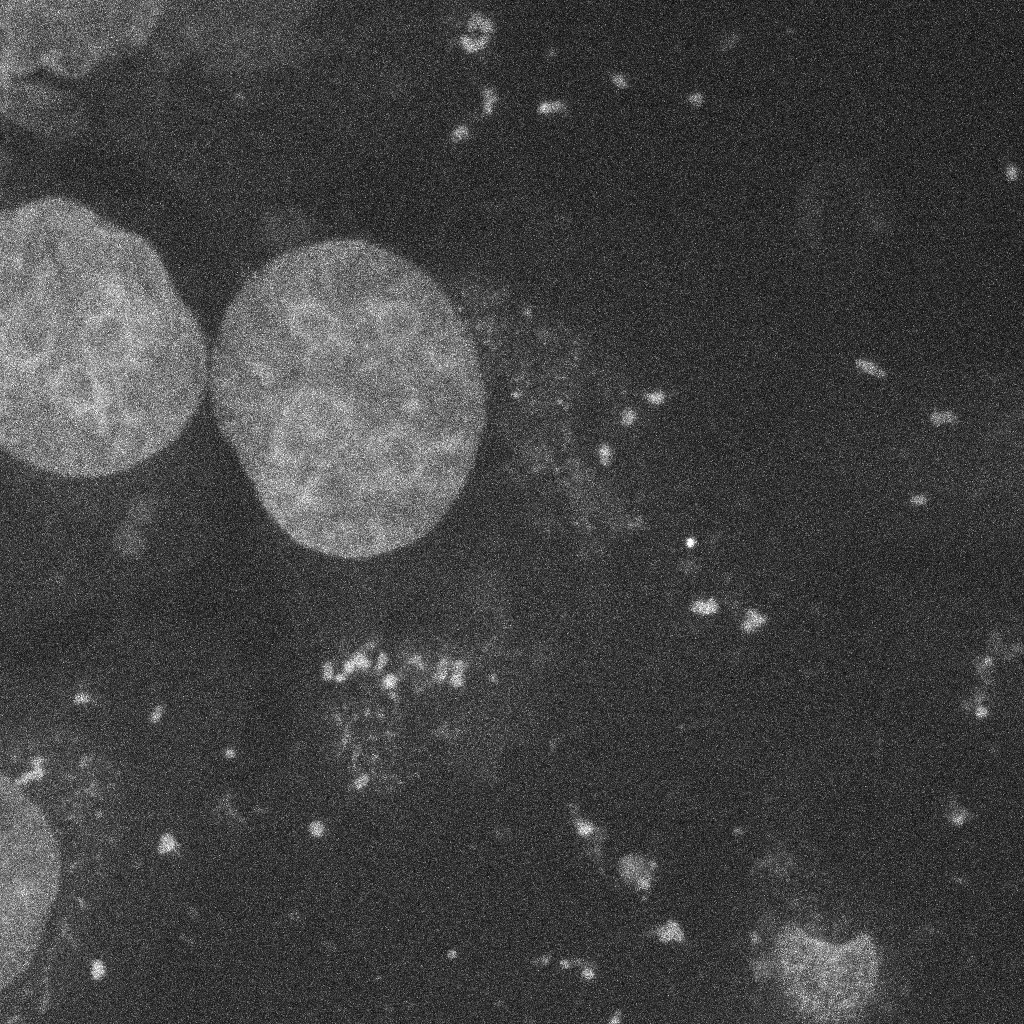

Supplement: Supplementary file 6 — Source data Fig. 4 [file 44319_2024_206_MOESM6_ESM.zip › Figure 4/4E/BAX/4E_BAX_DAPI.tif]

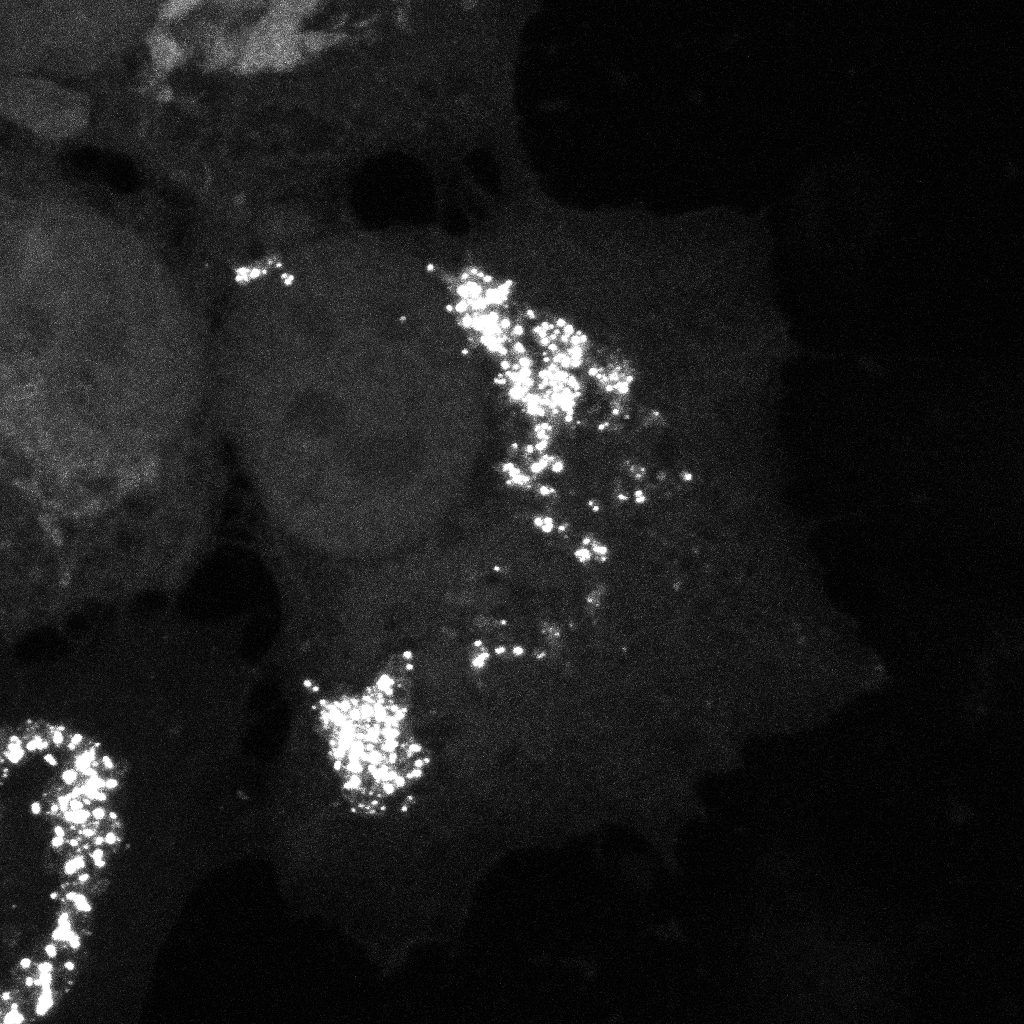

Supplement: Supplementary file 6 — Source data Fig. 4 [file 44319_2024_206_MOESM6_ESM.zip › Figure 4/4E/BAX/4E_BAX_EGFP.tif]

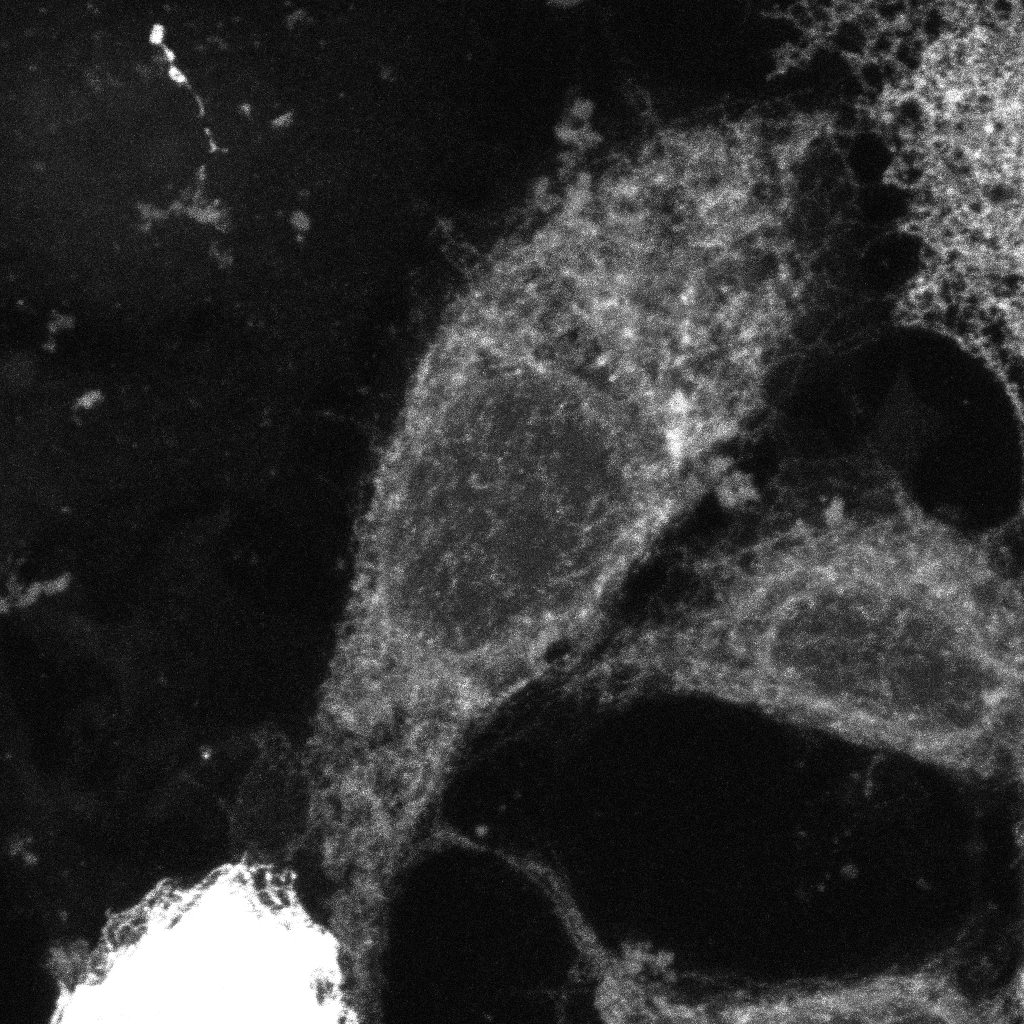

Supplement: Supplementary file 6 — Source data Fig. 4 [file 44319_2024_206_MOESM6_ESM.zip › Figure 4/4E/BOK/4E_BOK_BCL2.tif]

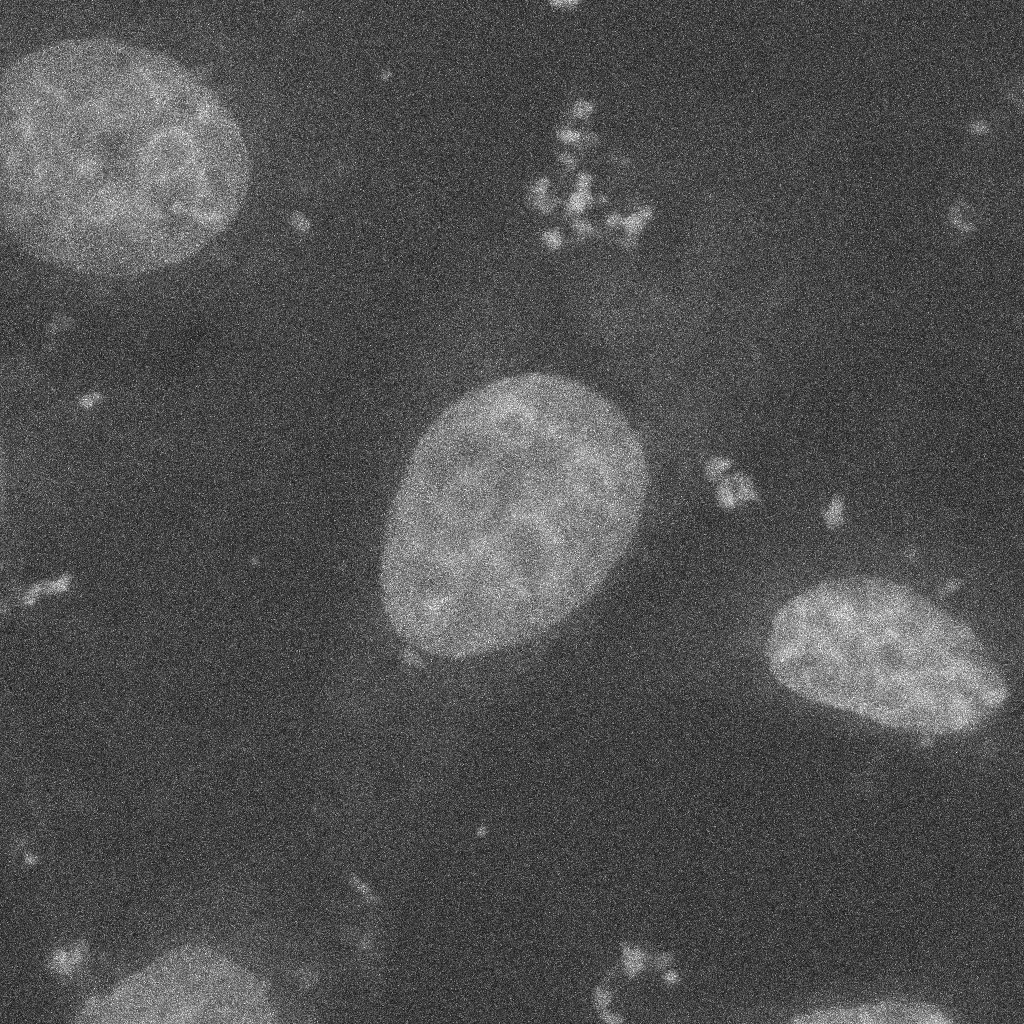

Supplement: Supplementary file 6 — Source data Fig. 4 [file 44319_2024_206_MOESM6_ESM.zip › Figure 4/4E/BOK/4E_BOK_DAPI.tif]

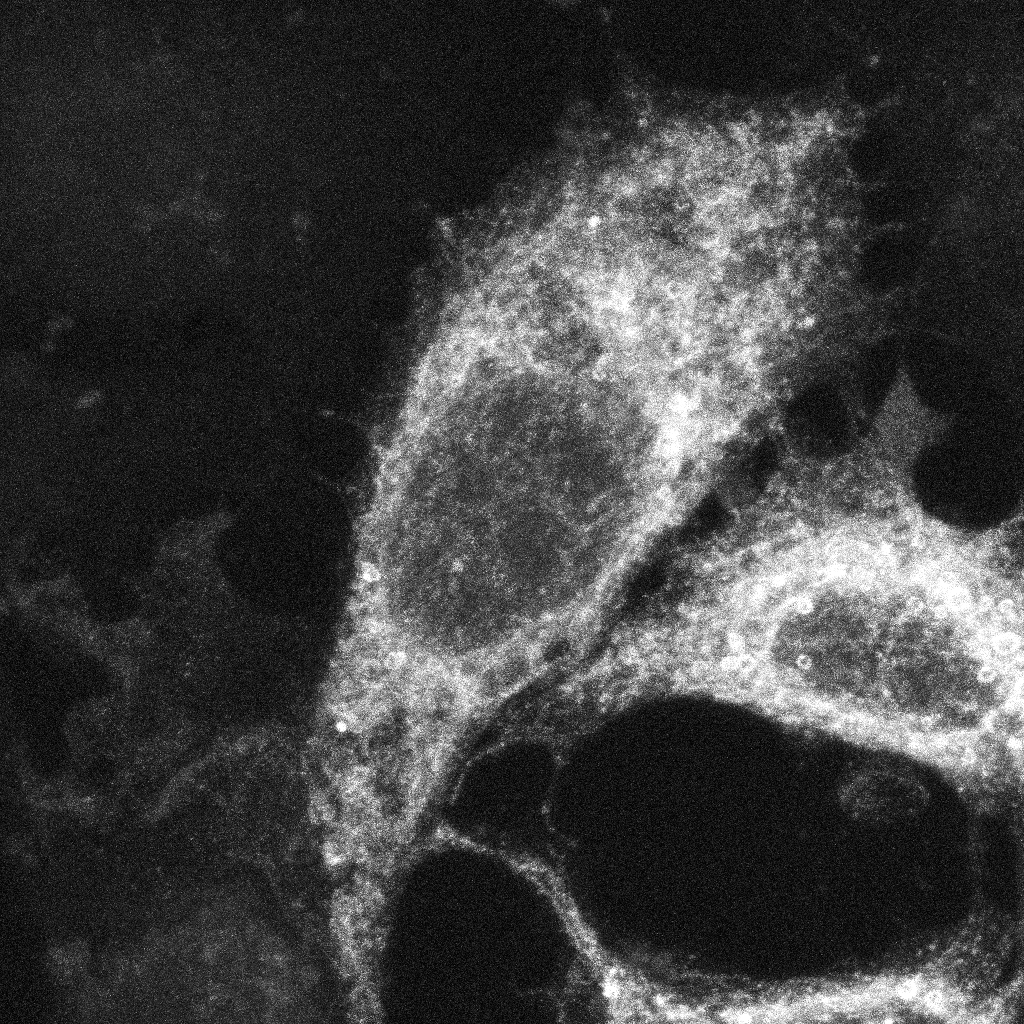

Supplement: Supplementary file 6 — Source data Fig. 4 [file 44319_2024_206_MOESM6_ESM.zip › Figure 4/4E/BOK/4E_BOK_EGFP.tif]

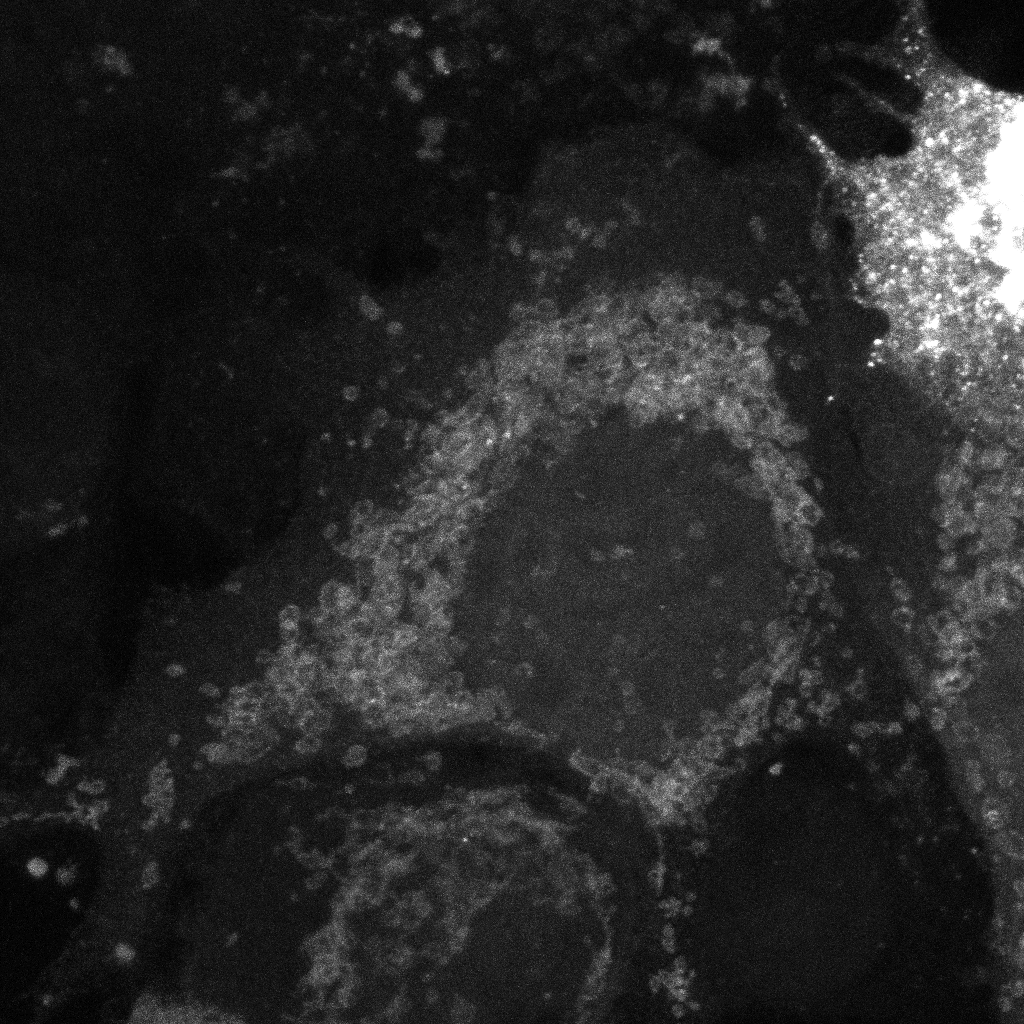

Supplement: Supplementary file 6 — Source data Fig. 4 [file 44319_2024_206_MOESM6_ESM.zip › Figure 4/4F/BAK/4F_BAK_BCL2.tif]

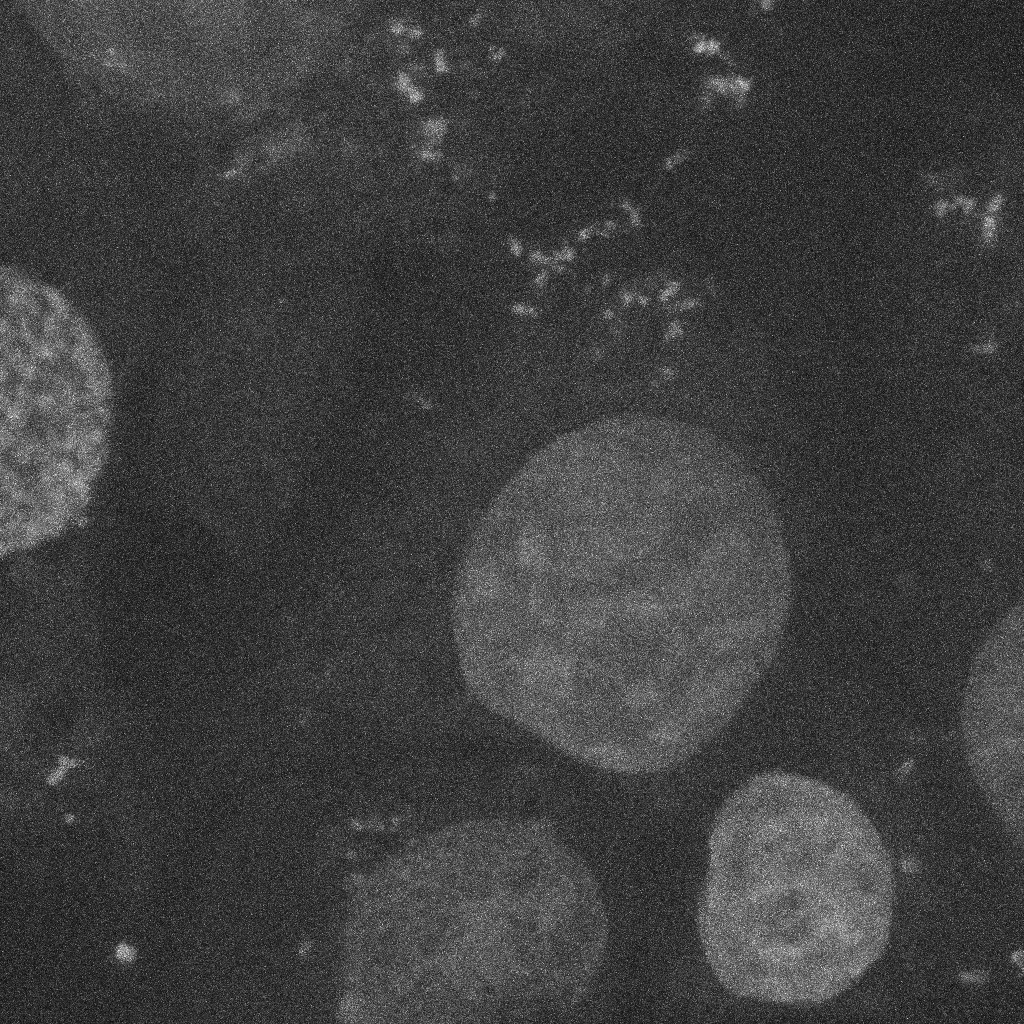

Supplement: Supplementary file 6 — Source data Fig. 4 [file 44319_2024_206_MOESM6_ESM.zip › Figure 4/4F/BAK/4F_BAK_DAPI.tif]

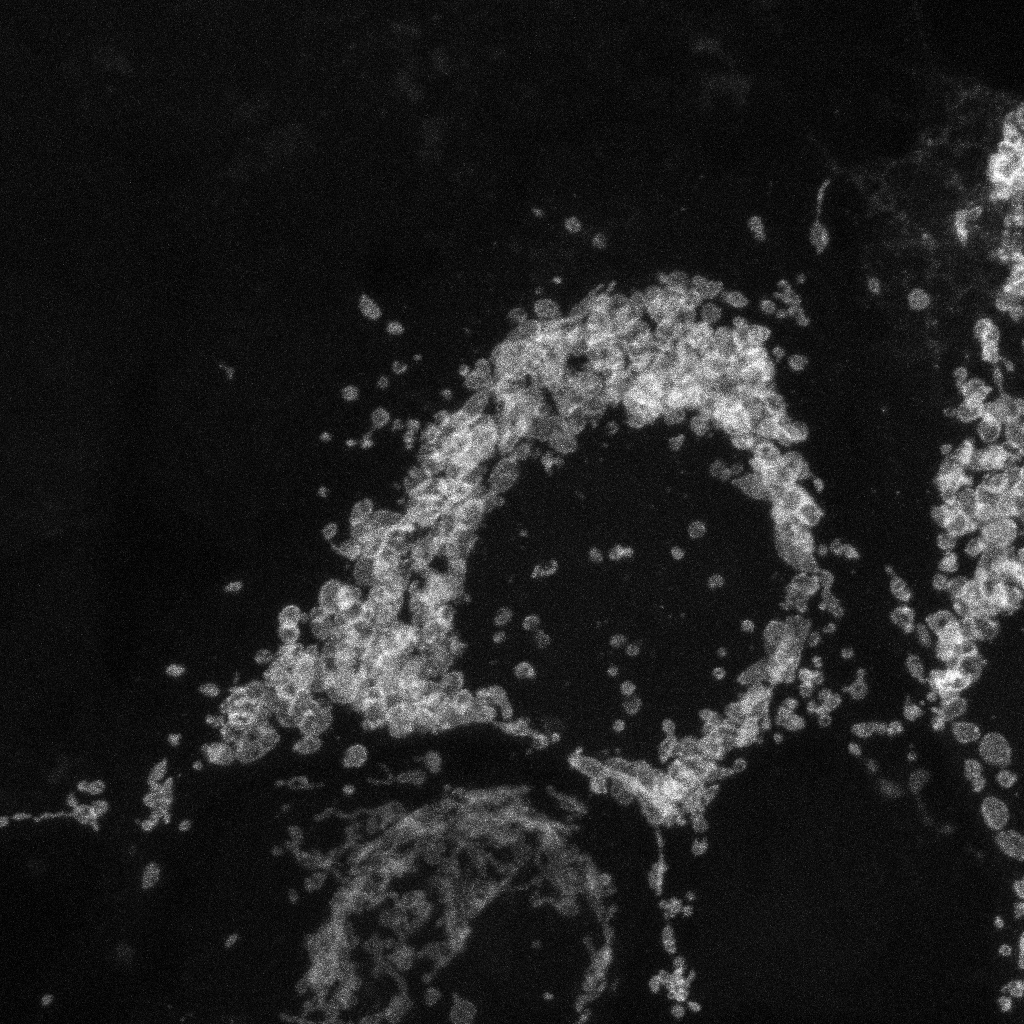

Supplement: Supplementary file 6 — Source data Fig. 4 [file 44319_2024_206_MOESM6_ESM.zip › Figure 4/4F/BAK/4F_BAK_EGFP.tif]

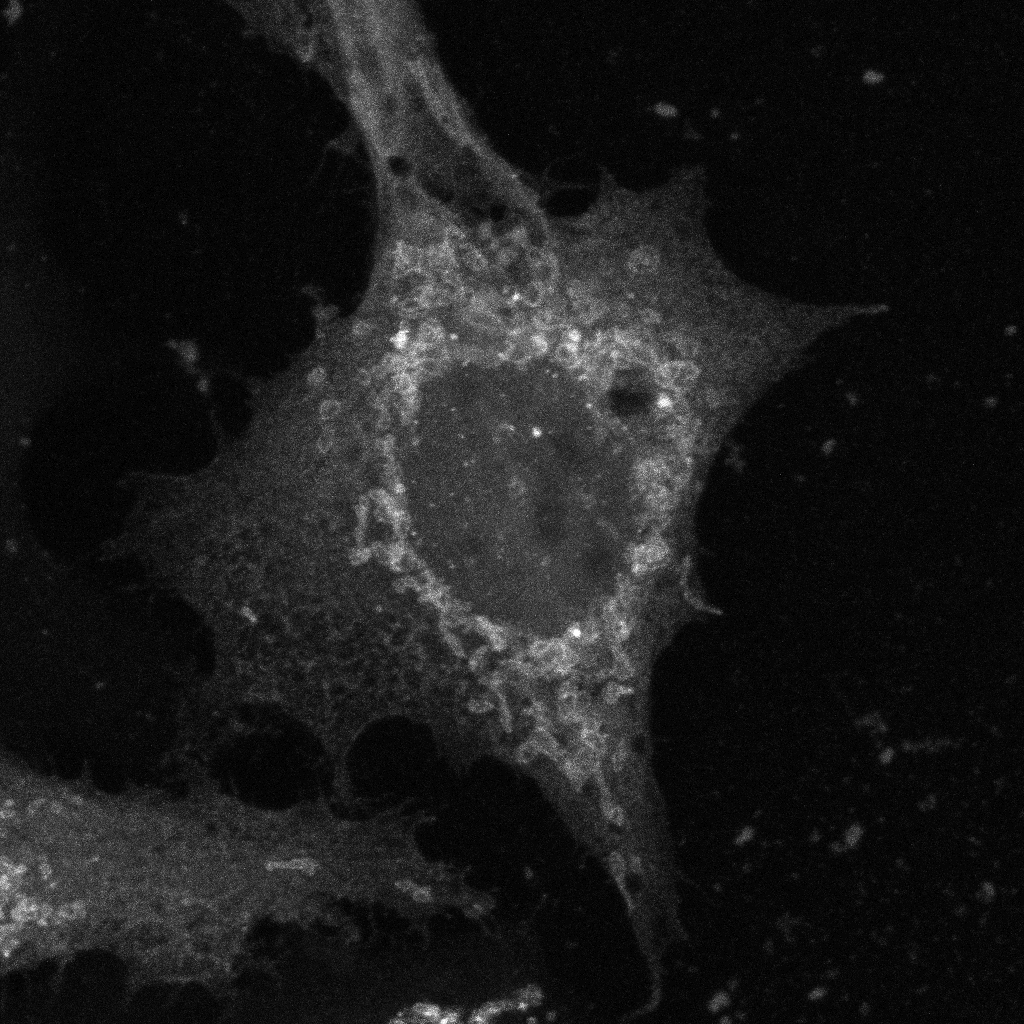

Supplement: Supplementary file 6 — Source data Fig. 4 [file 44319_2024_206_MOESM6_ESM.zip › Figure 4/4F/BAX/4F_BAX_BCL2.tif]

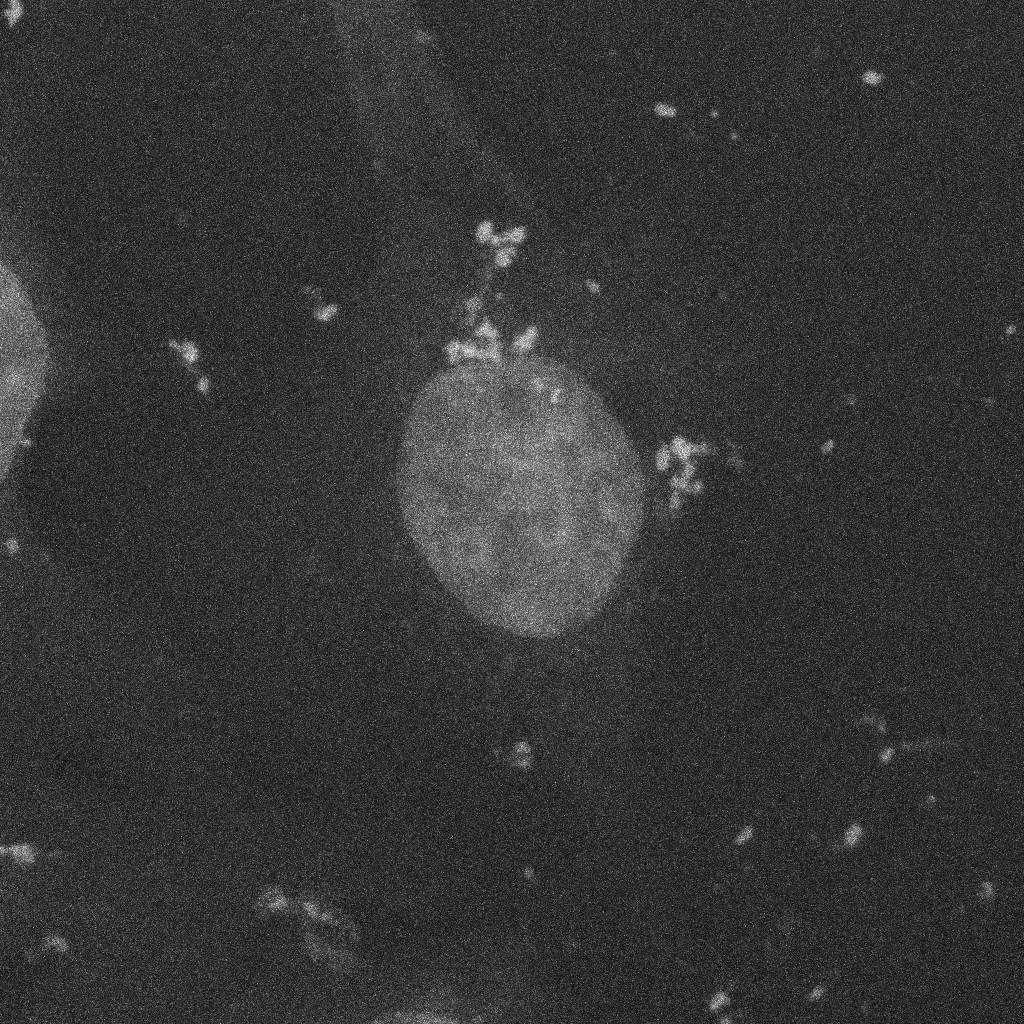

Supplement: Supplementary file 6 — Source data Fig. 4 [file 44319_2024_206_MOESM6_ESM.zip › Figure 4/4F/BAX/4F_BAX_DAPI.tif]

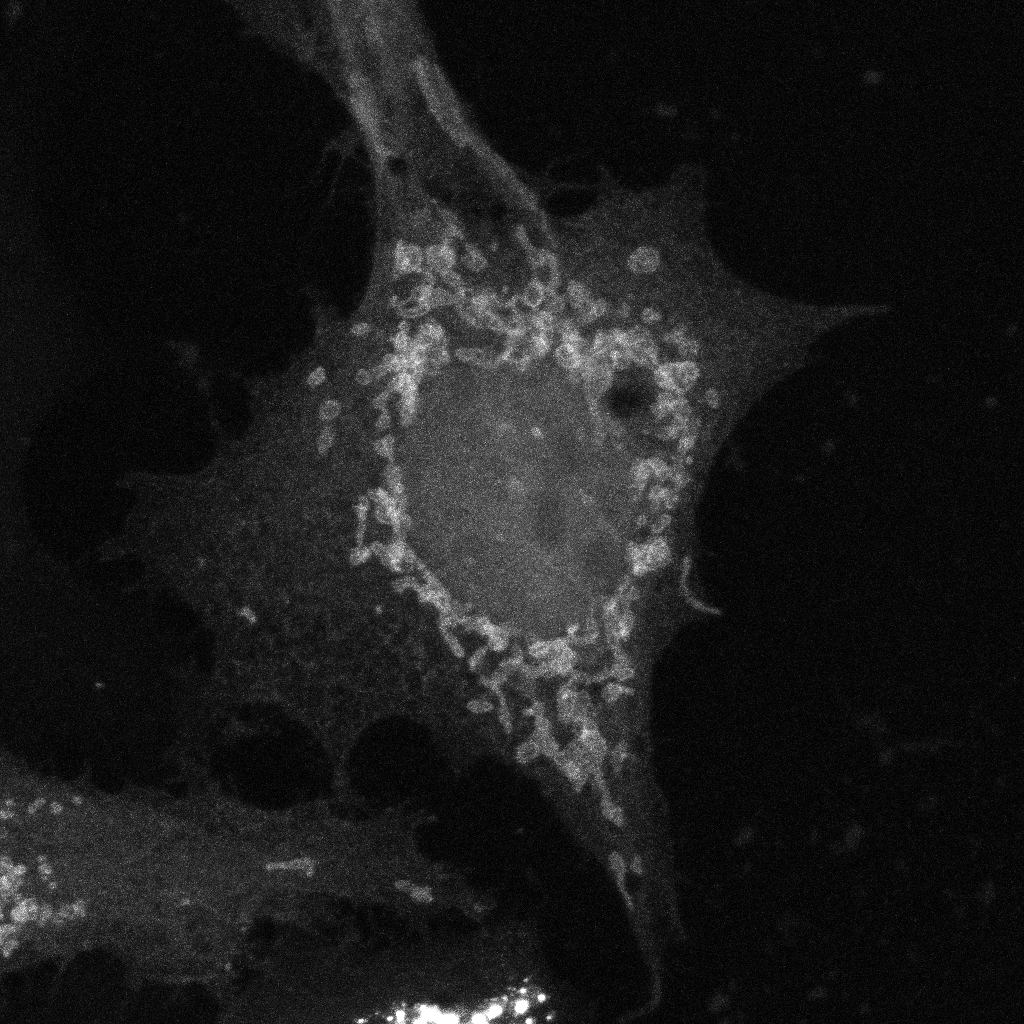

Supplement: Supplementary file 6 — Source data Fig. 4 [file 44319_2024_206_MOESM6_ESM.zip › Figure 4/4F/BAX/4F_BAX_EGFP.tif]

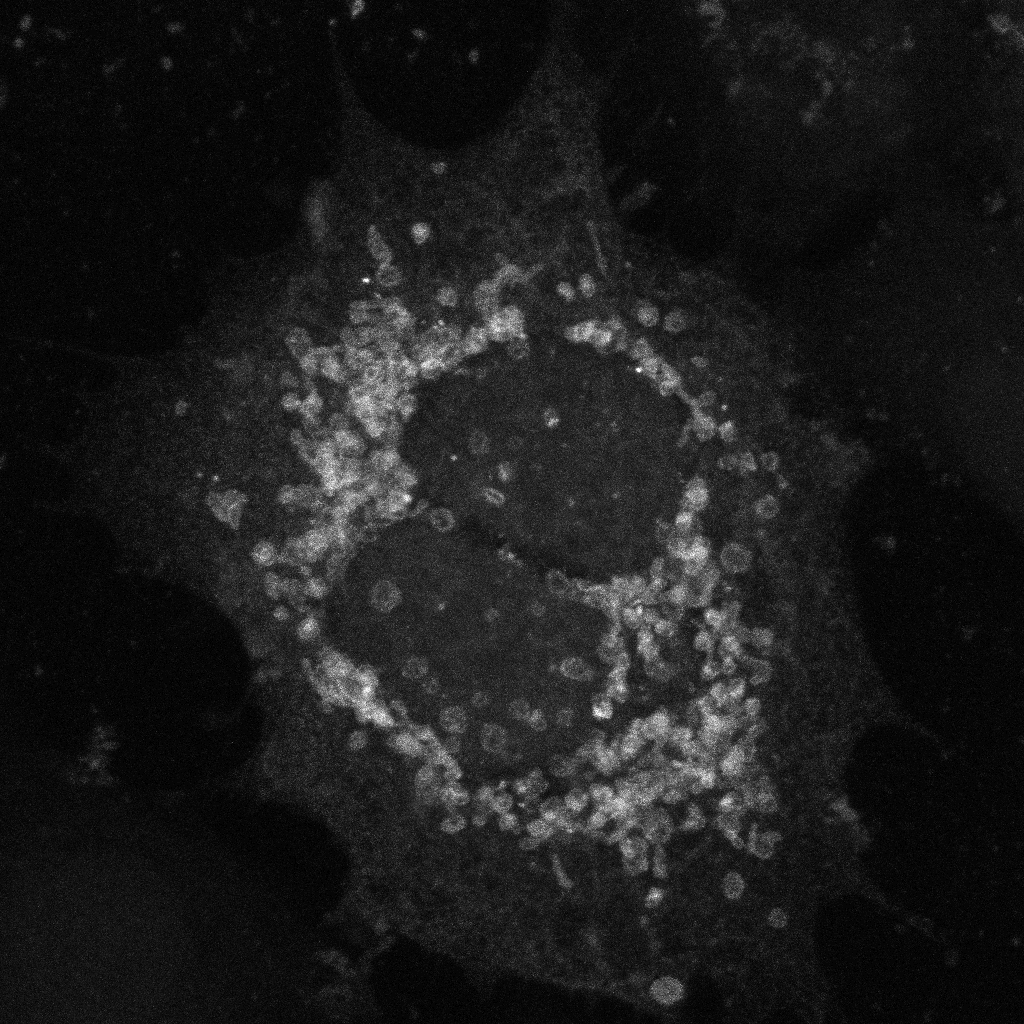

Supplement: Supplementary file 6 — Source data Fig. 4 [file 44319_2024_206_MOESM6_ESM.zip › Figure 4/4F/BOK/4F_BOK_BCL2.tif]

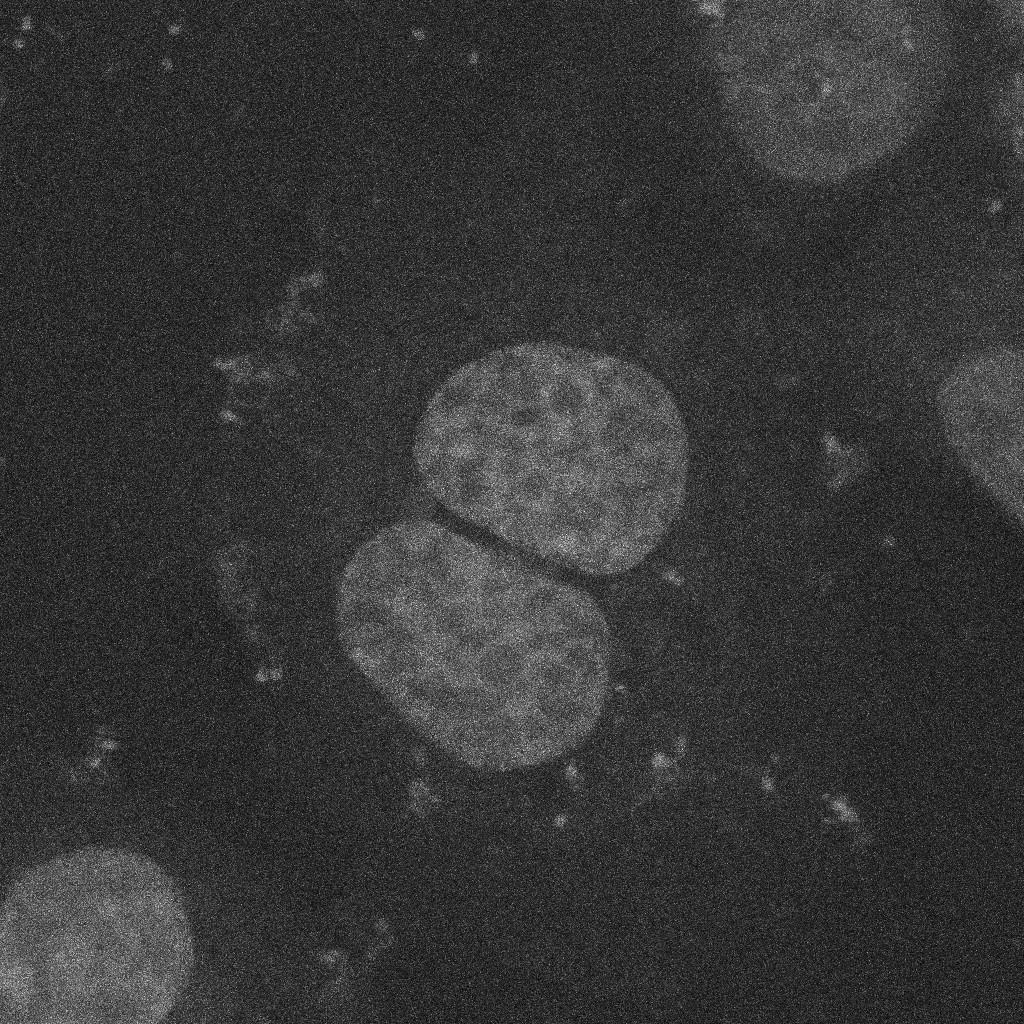

Supplement: Supplementary file 6 — Source data Fig. 4 [file 44319_2024_206_MOESM6_ESM.zip › Figure 4/4F/BOK/4F_BOK_DAPI.tif]

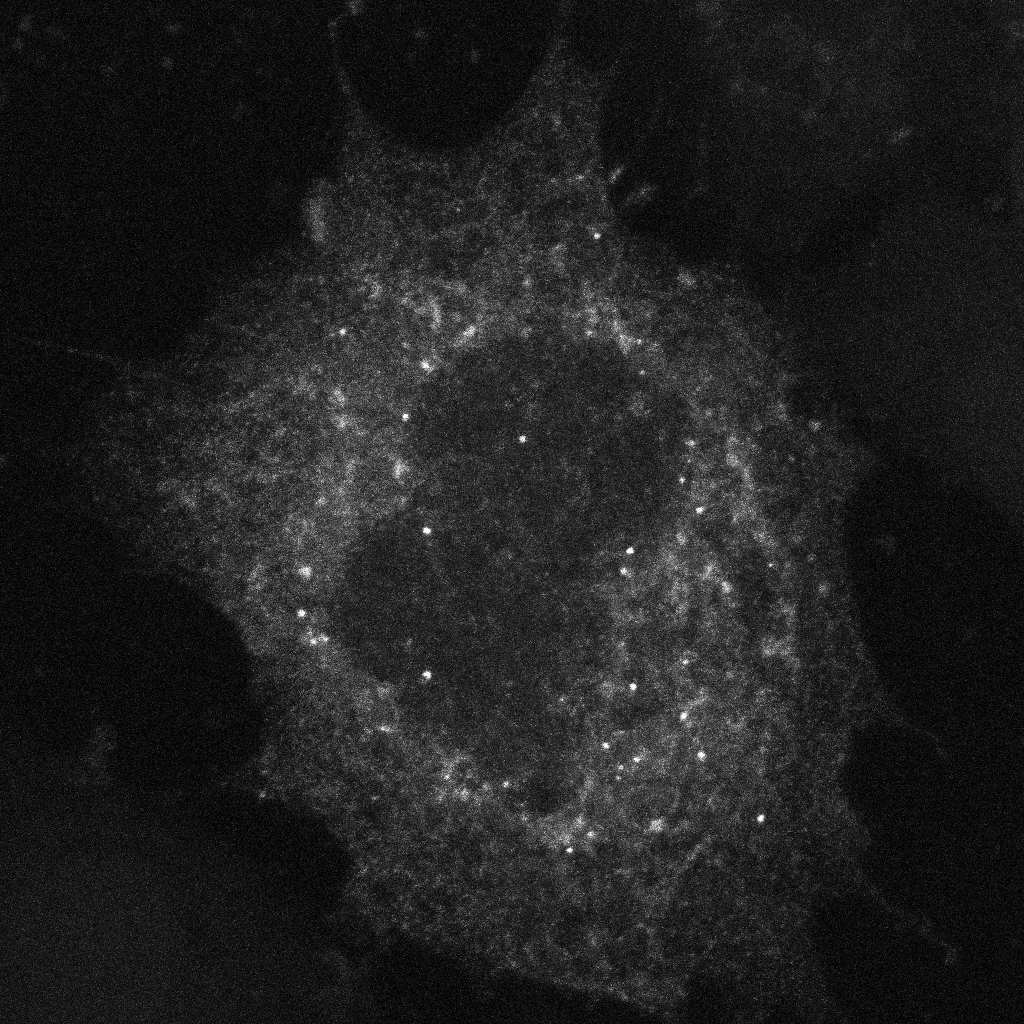

Supplement: Supplementary file 6 — Source data Fig. 4 [file 44319_2024_206_MOESM6_ESM.zip › Figure 4/4F/BOK/4F_BOK_EGFP.tif]

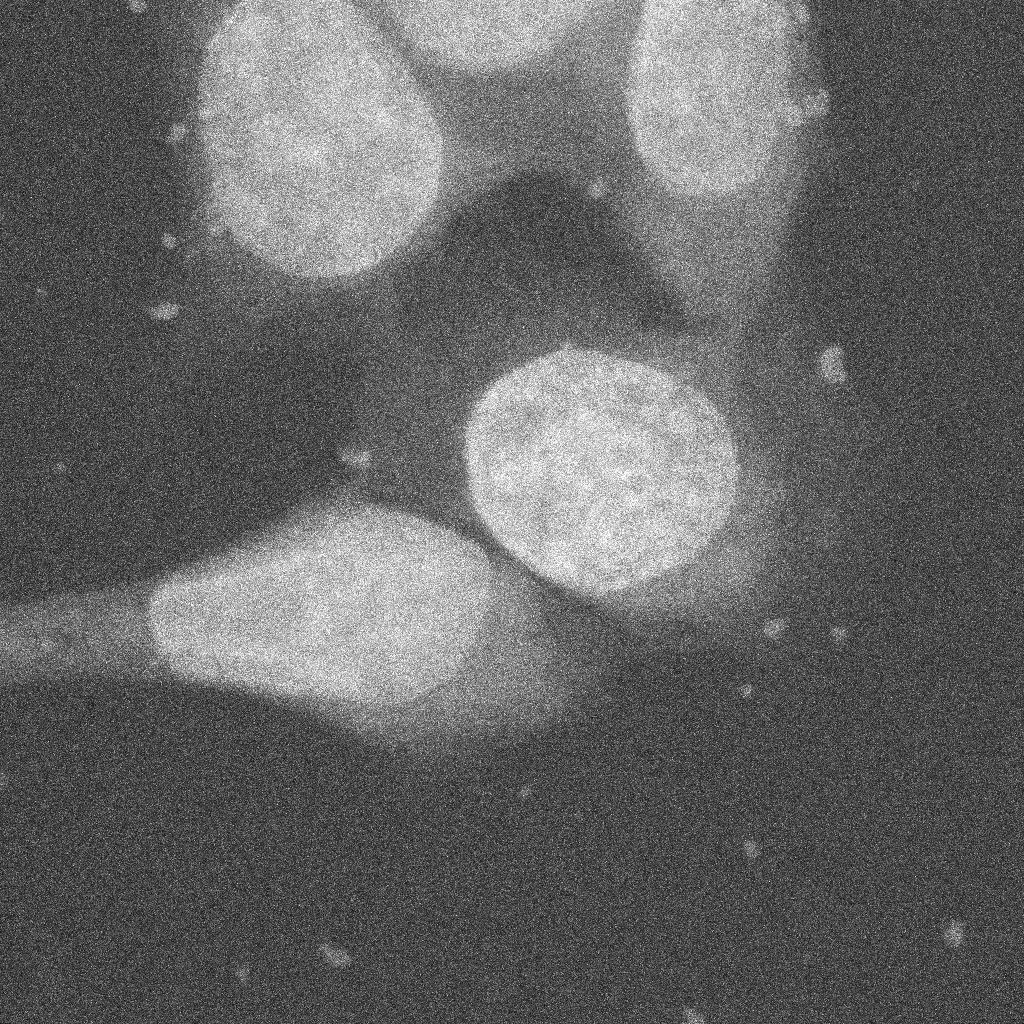

Supplement: Supplementary file 6 — Source data Fig. 4 [file 44319_2024_206_MOESM6_ESM.zip › Figure 4/4G/mCherry-BCL2/4G_BOKL70E_BCL2_DAPI.tif]

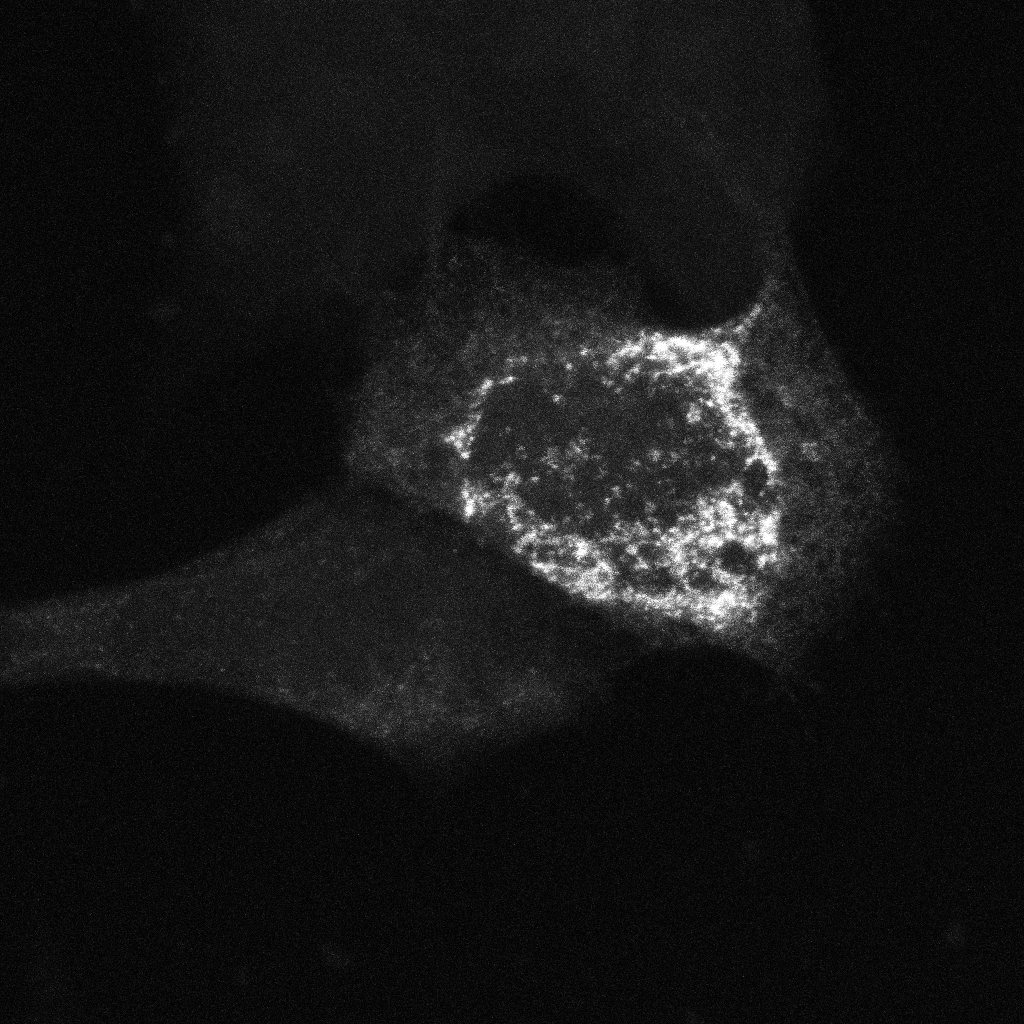

Supplement: Supplementary file 6 — Source data Fig. 4 [file 44319_2024_206_MOESM6_ESM.zip › Figure 4/4G/mCherry-BCL2/4G_BOKL70E_BCL2_EGFP.tif]

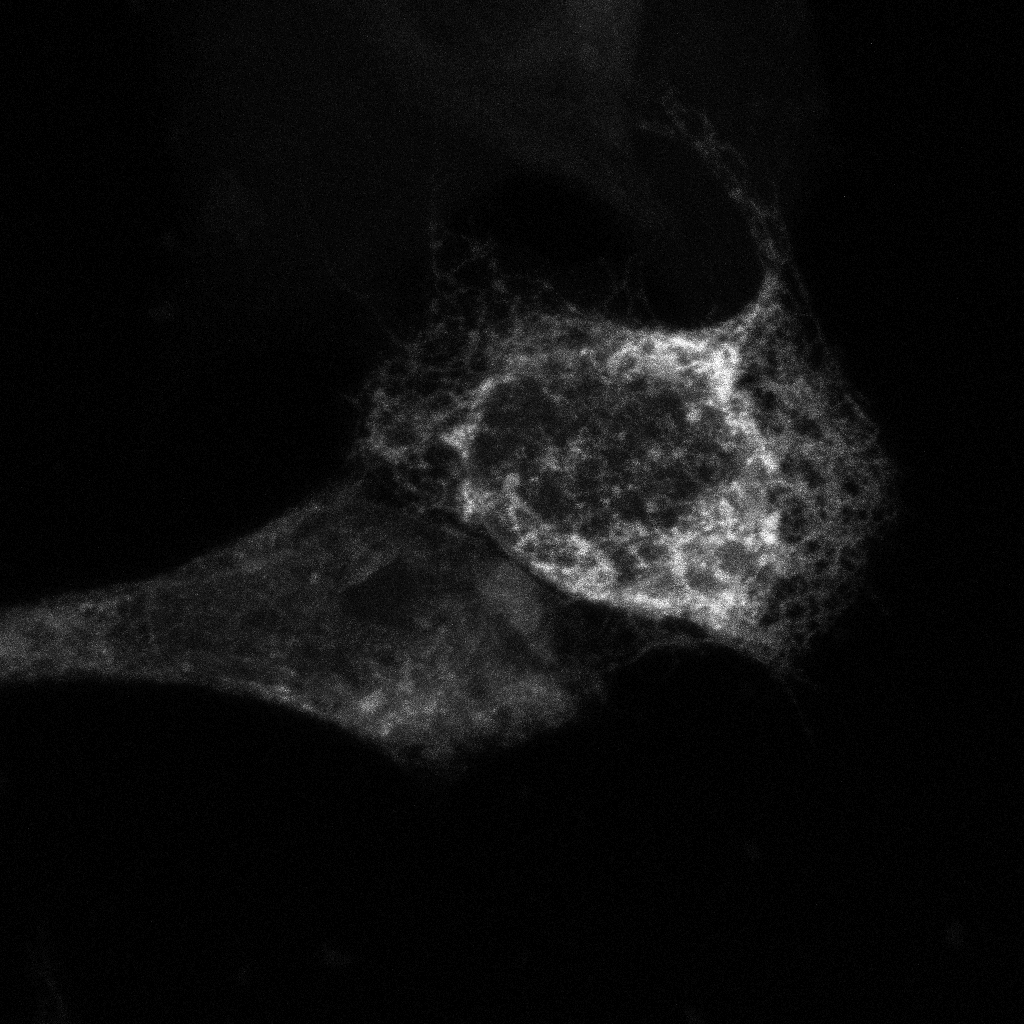

Supplement: Supplementary file 6 — Source data Fig. 4 [file 44319_2024_206_MOESM6_ESM.zip › Figure 4/4G/mCherry-BCL2/4G_BOKL70E_BCL2_mCherry.tif]

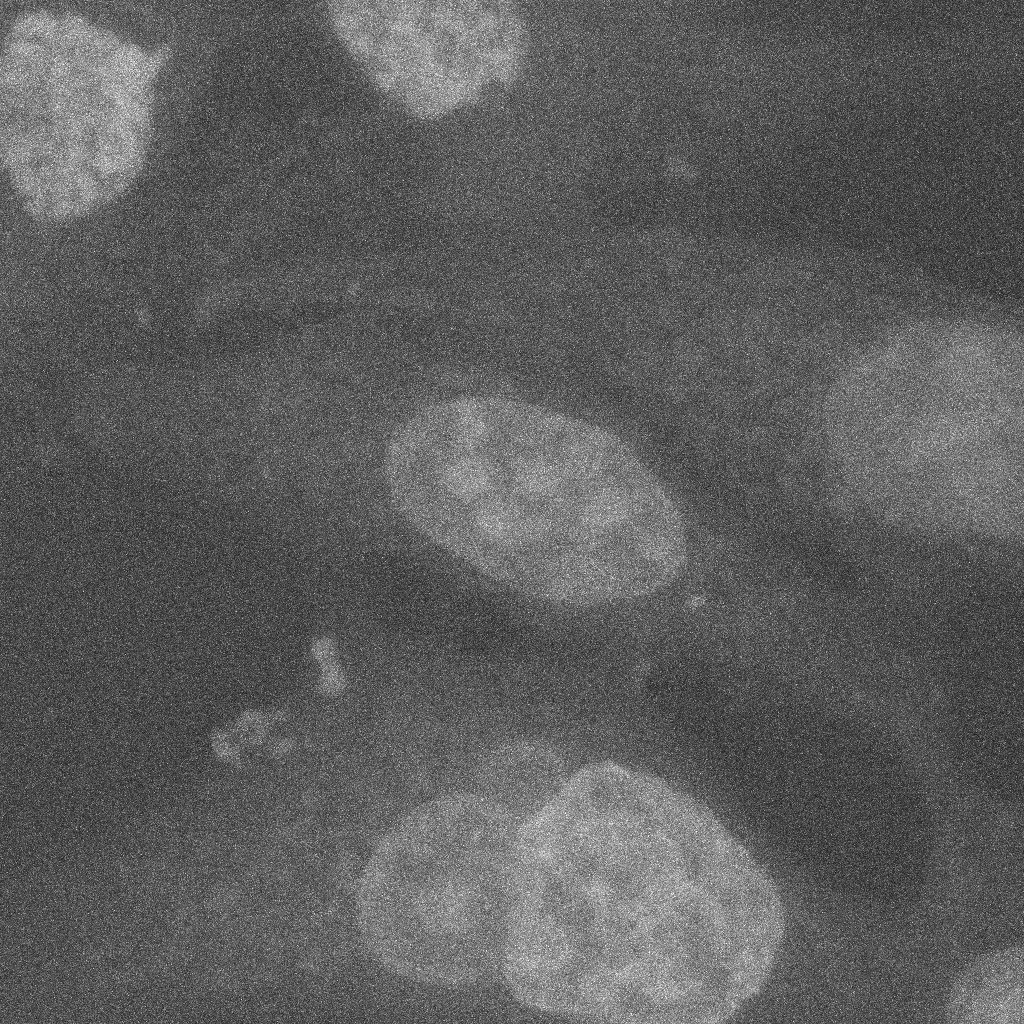

Supplement: Supplementary file 6 — Source data Fig. 4 [file 44319_2024_206_MOESM6_ESM.zip › Figure 4/4G/MitoRed/4G_BOKL70E_Mito_DAPI.tif]

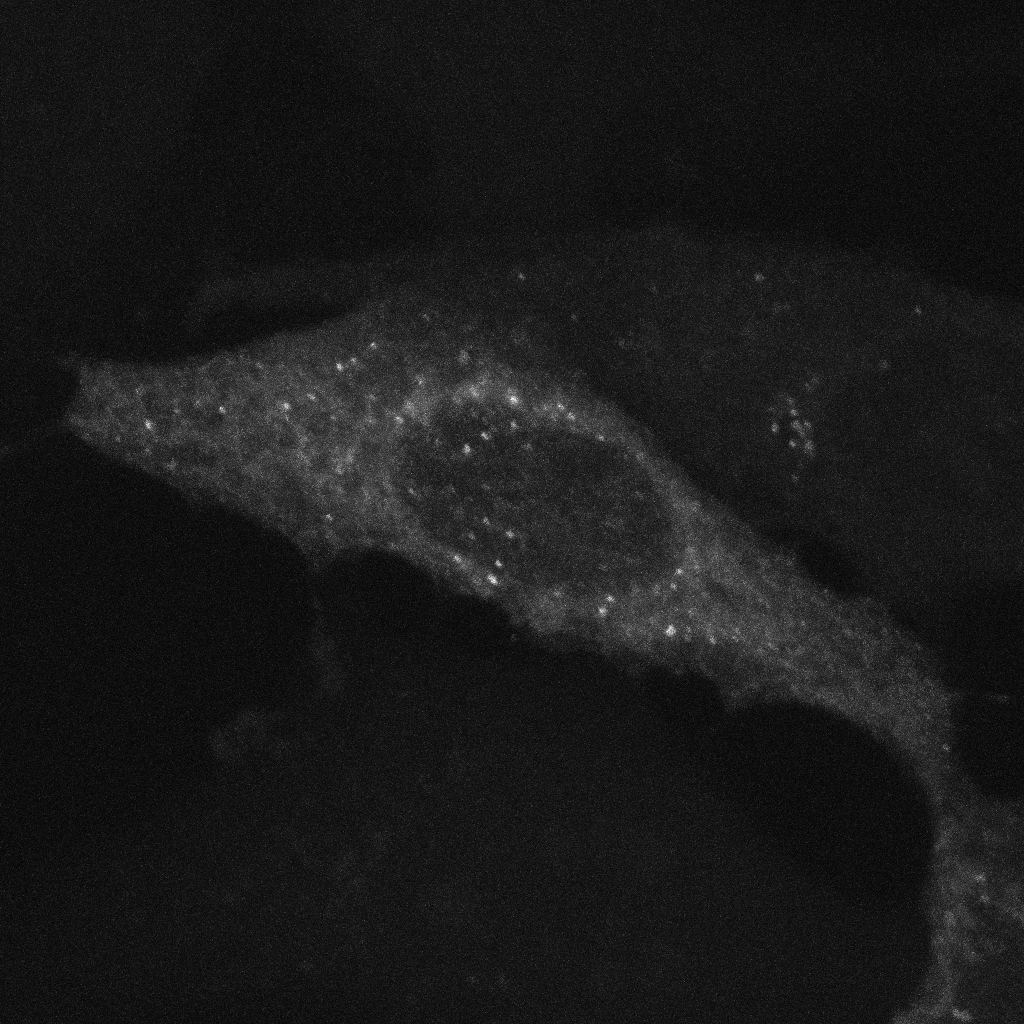

Supplement: Supplementary file 6 — Source data Fig. 4 [file 44319_2024_206_MOESM6_ESM.zip › Figure 4/4G/MitoRed/4G_BOKL70E_Mito_EGFP.tif]

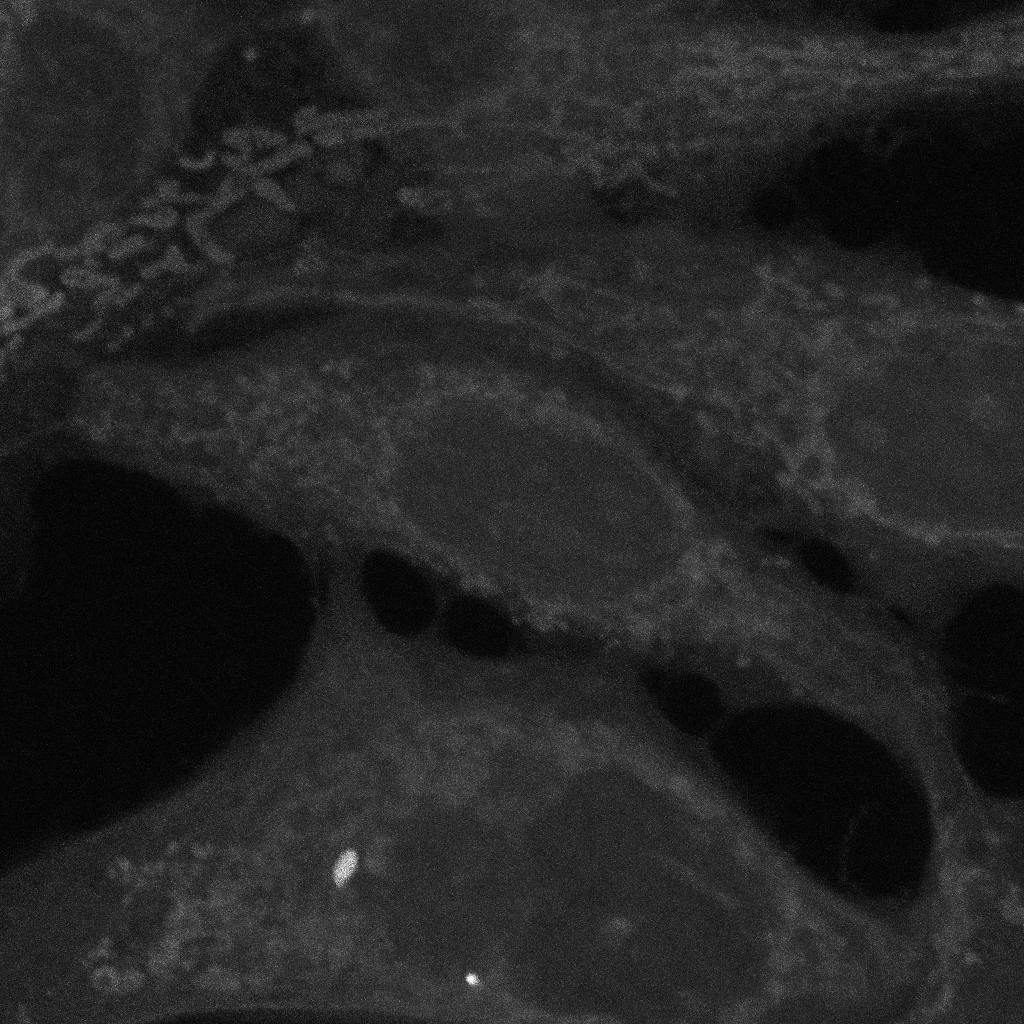

Supplement: Supplementary file 6 — Source data Fig. 4 [file 44319_2024_206_MOESM6_ESM.zip › Figure 4/4G/MitoRed/4G_BOKL70E_Mito_MitoRed.tif]

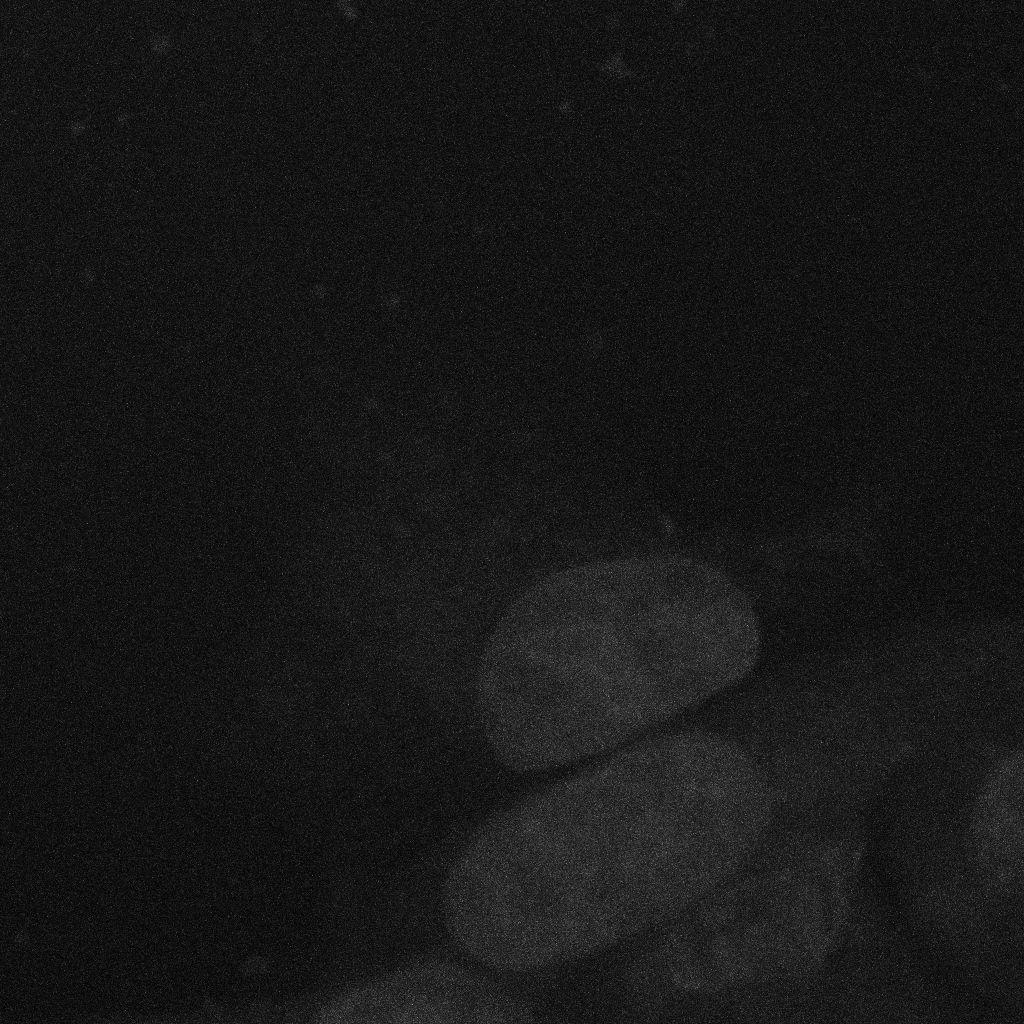

Supplement: Supplementary file 6 — Source data Fig. 4 [file 44319_2024_206_MOESM6_ESM.zip › Figure 4/4H/EGFP-BCL2/4H_BOKdeltaTMD_BCL2_DAPI.tif]

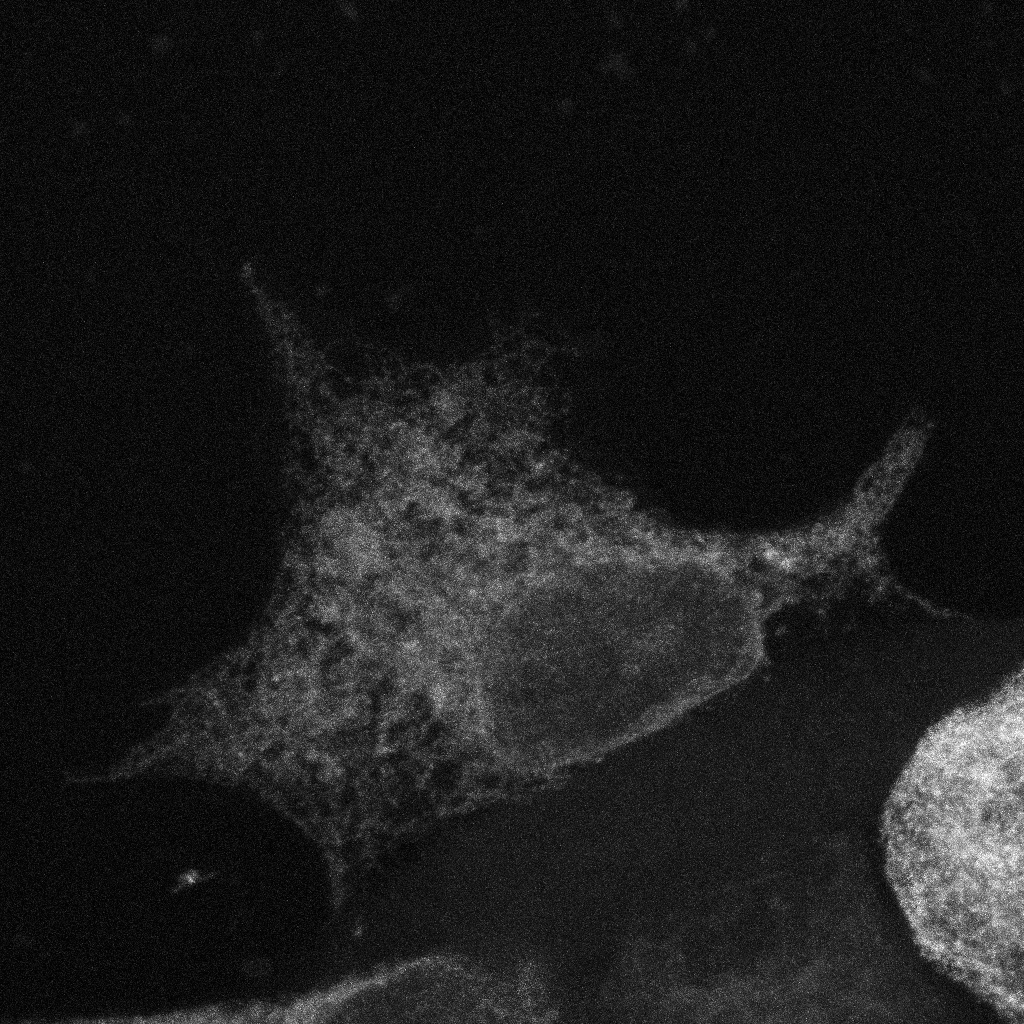

Supplement: Supplementary file 6 — Source data Fig. 4 [file 44319_2024_206_MOESM6_ESM.zip › Figure 4/4H/EGFP-BCL2/4H_BOKdeltaTMD_BCL2_EGFP.tif]

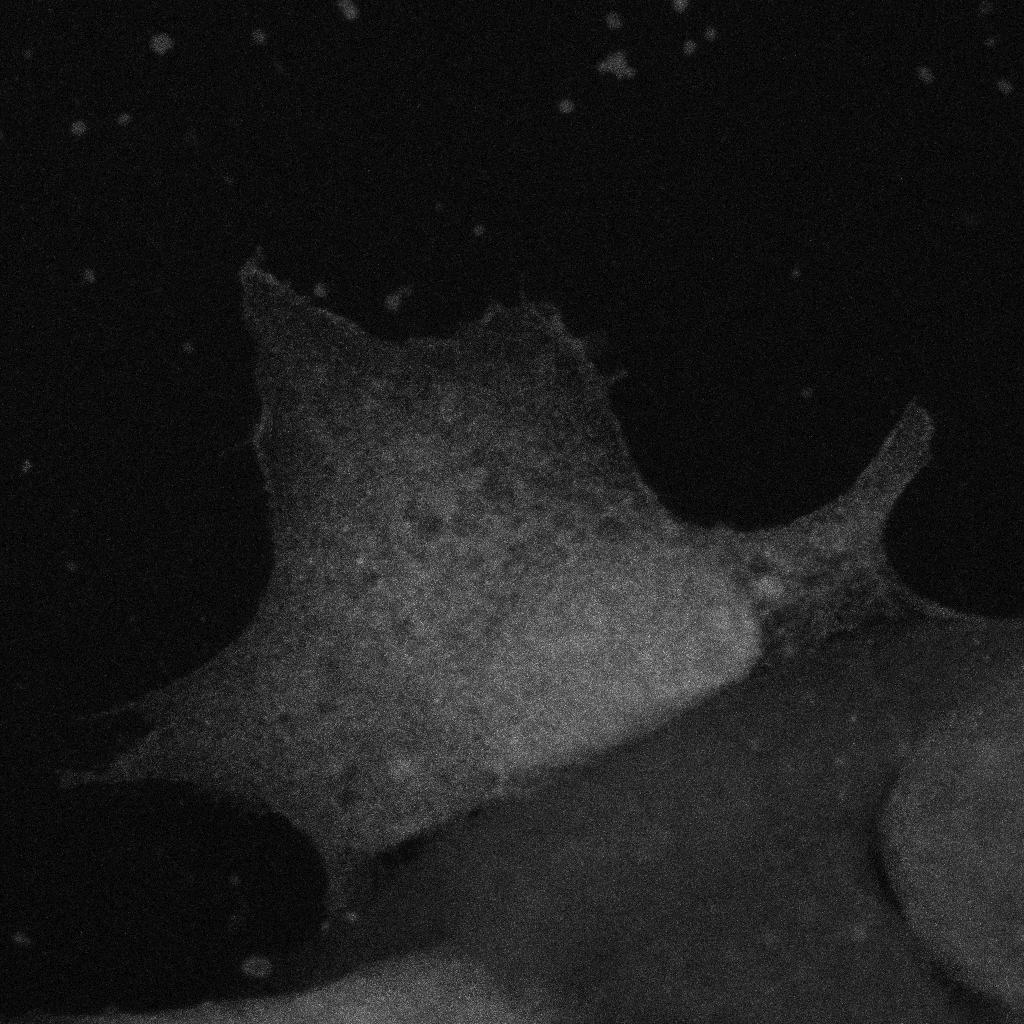

Supplement: Supplementary file 6 — Source data Fig. 4 [file 44319_2024_206_MOESM6_ESM.zip › Figure 4/4H/EGFP-BCL2/4H_BOKdeltaTMD_BCL2_mCherry.tif]

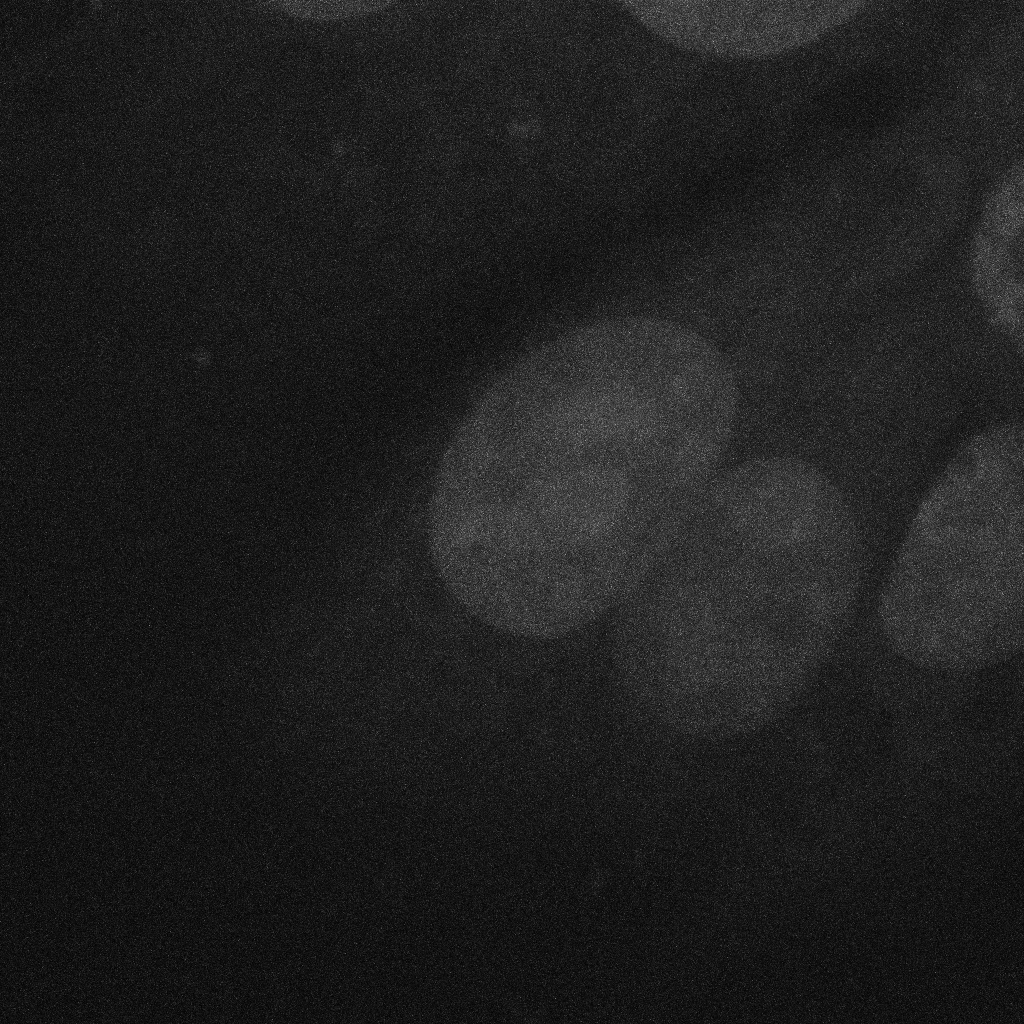

Supplement: Supplementary file 6 — Source data Fig. 4 [file 44319_2024_206_MOESM6_ESM.zip › Figure 4/4H/EYFP-Mito/4H_BOKdeltaTMD_Mito_DAPI.tif]

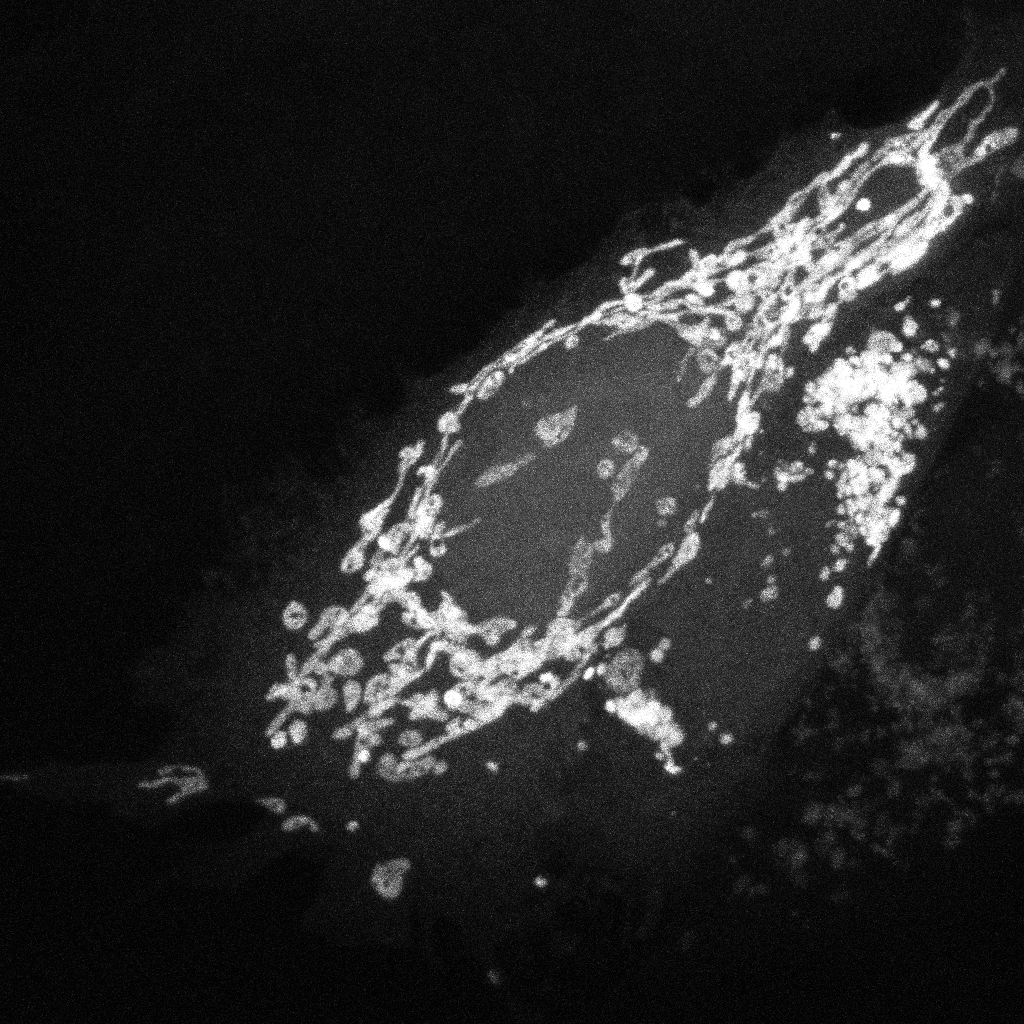

Supplement: Supplementary file 6 — Source data Fig. 4 [file 44319_2024_206_MOESM6_ESM.zip › Figure 4/4H/EYFP-Mito/4H_BOKdeltaTMD_Mito_EYFP.tif]

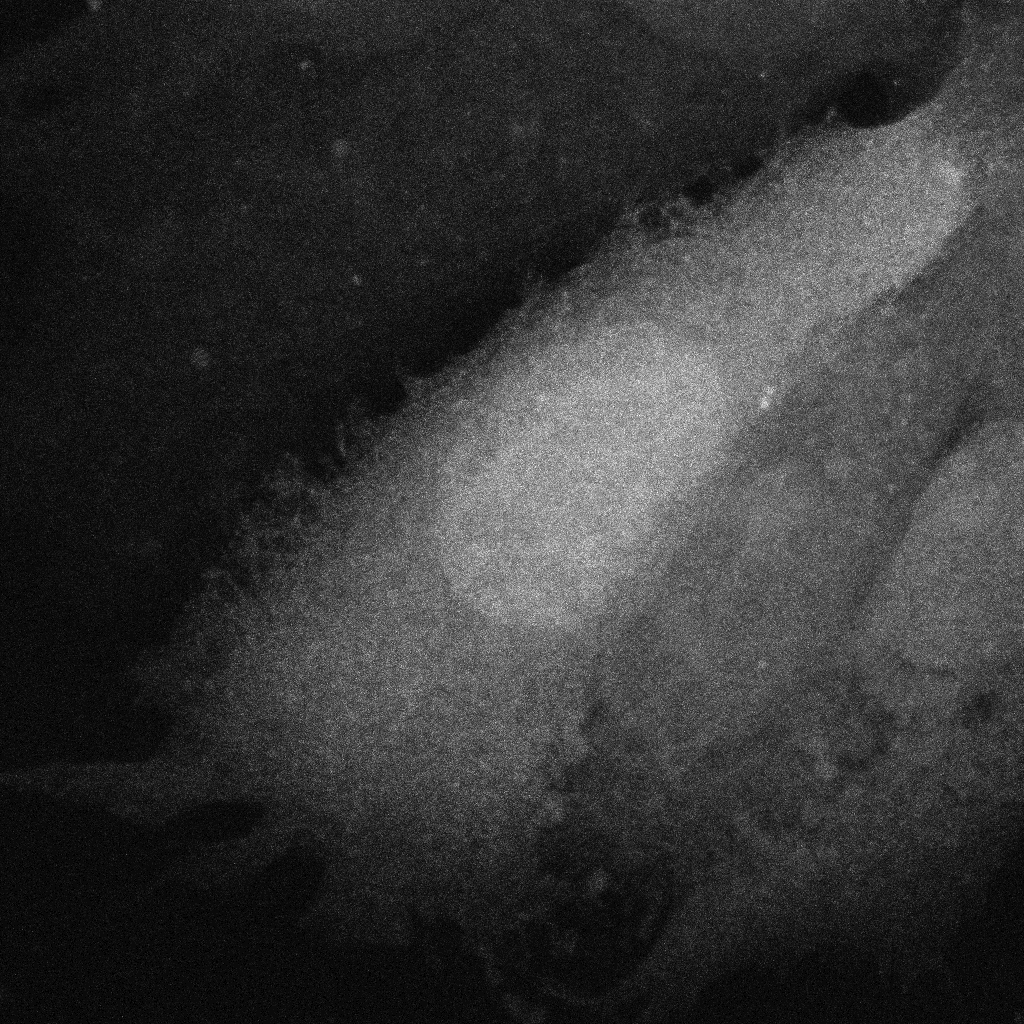

Supplement: Supplementary file 6 — Source data Fig. 4 [file 44319_2024_206_MOESM6_ESM.zip › Figure 4/4H/EYFP-Mito/4H_BOKdeltaTMD_Mito_mCherry.tif]

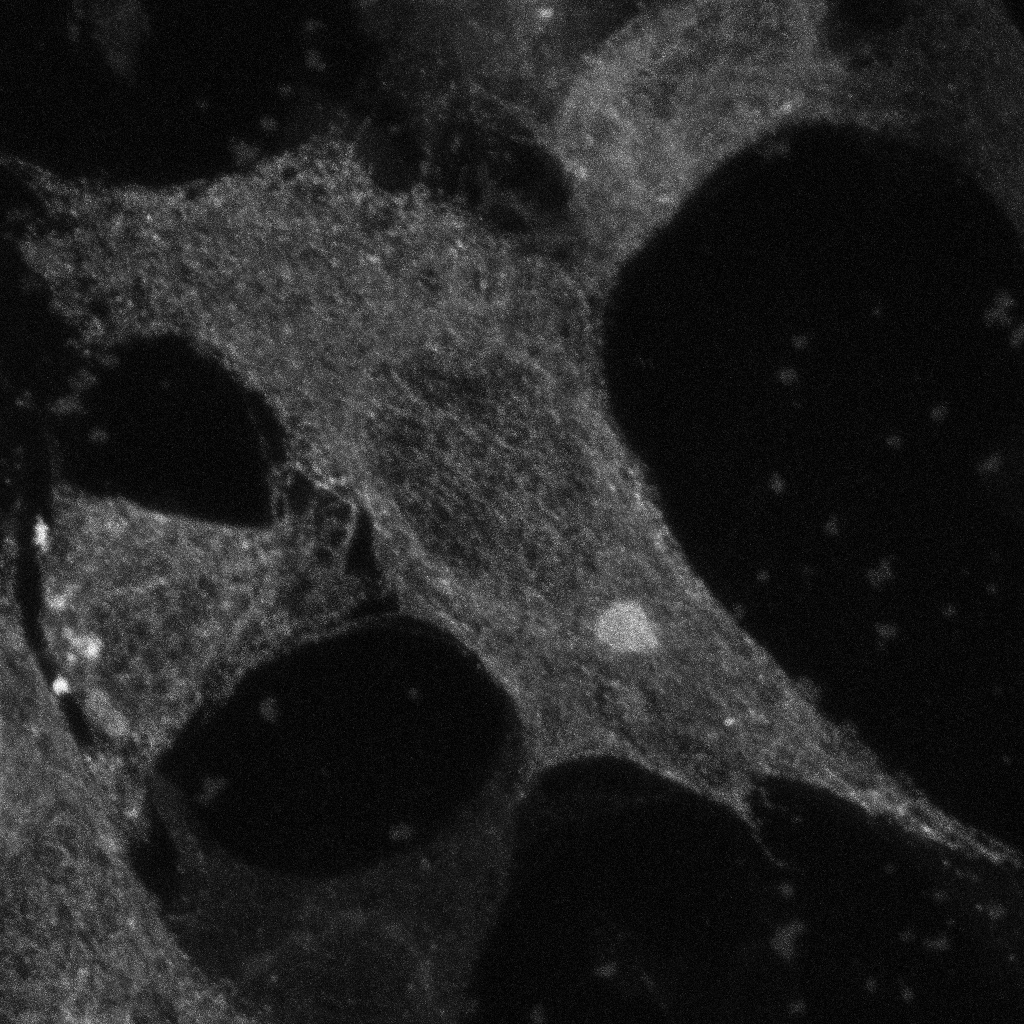

Supplement: Supplementary file 8 — Source data Fig. 6 [file 44319_2024_206_MOESM8_ESM.zip › Figure 6/6B/EYFP-ER/6B_BCL2_I_A_EYFP.tif]

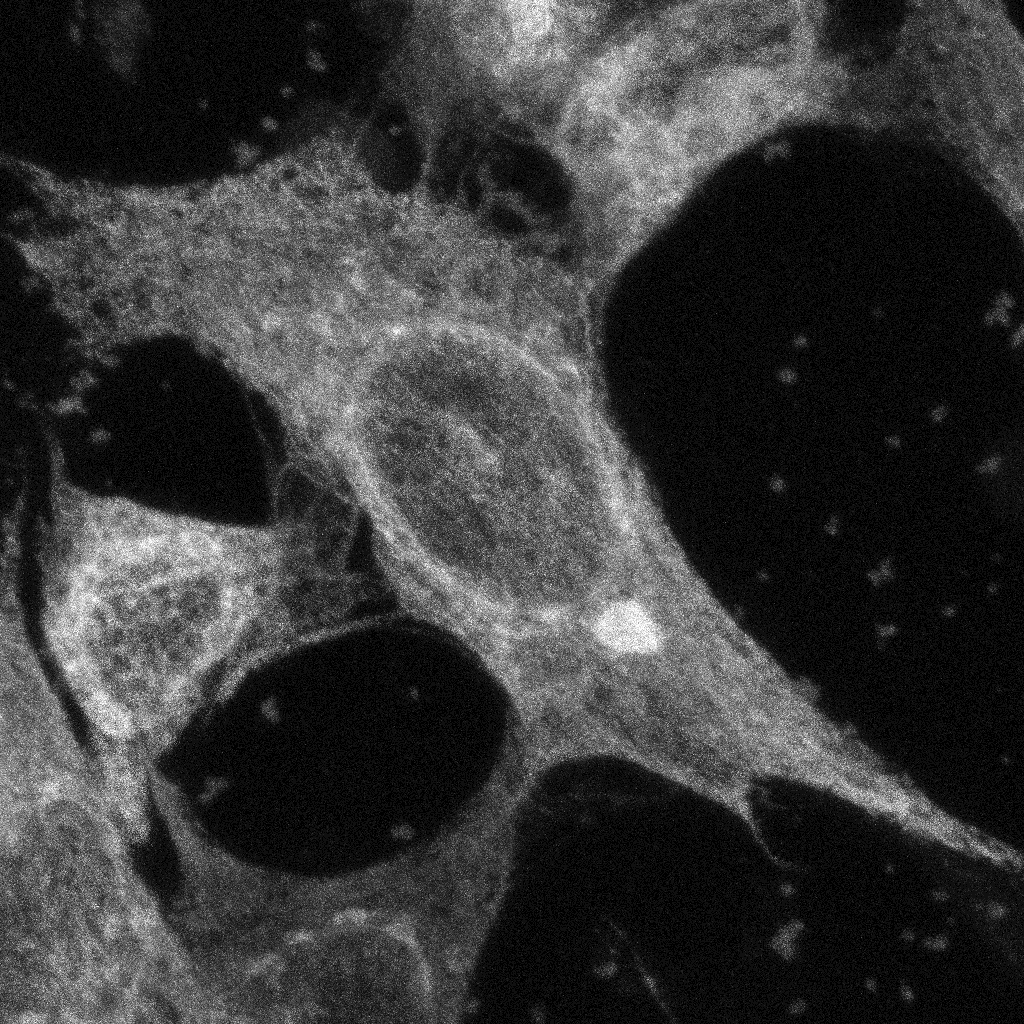

Supplement: Supplementary file 8 — Source data Fig. 6 [file 44319_2024_206_MOESM8_ESM.zip › Figure 6/6B/EYFP-ER/6B_BCL2_I_A_mTurq2.tif]

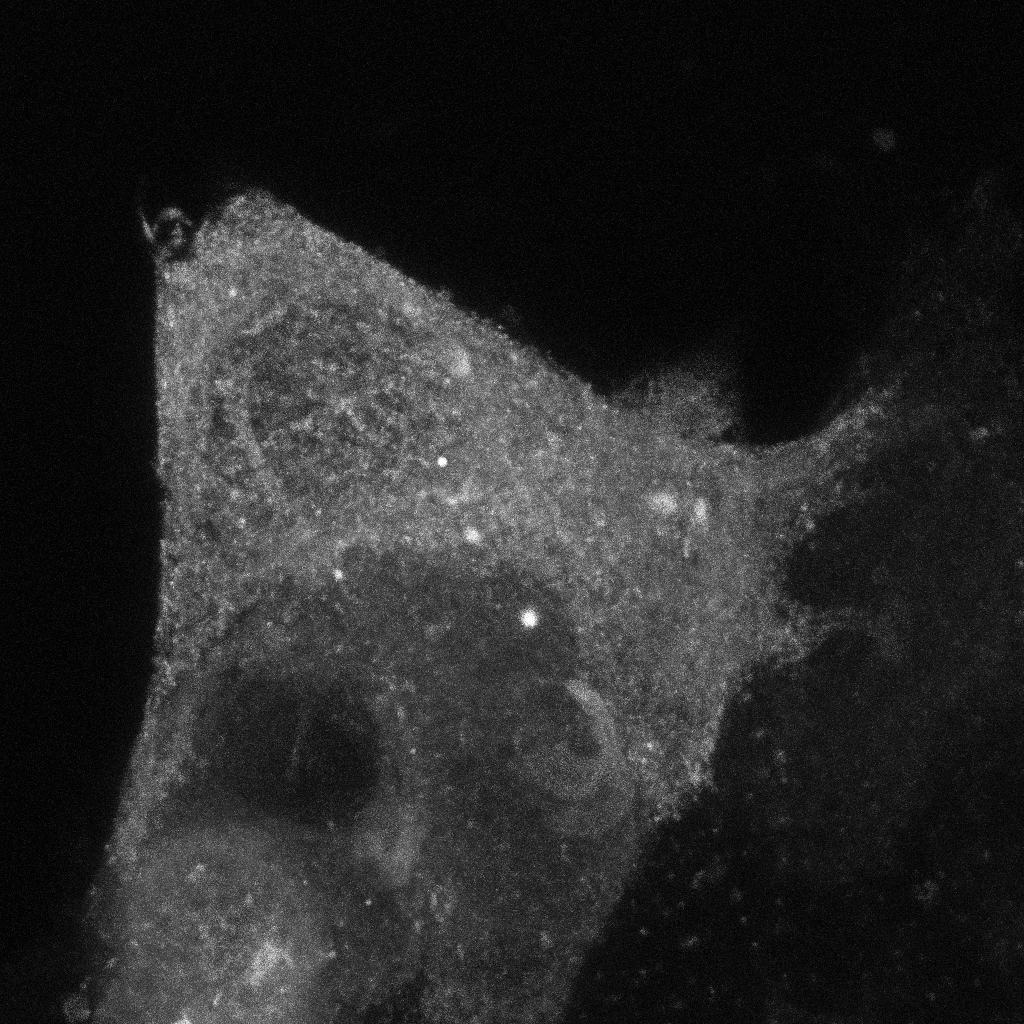

Supplement: Supplementary file 8 — Source data Fig. 6 [file 44319_2024_206_MOESM8_ESM.zip › Figure 6/6B/EYFP-ER/6B_BCL2_LVI_AAA_EYFP.tif]

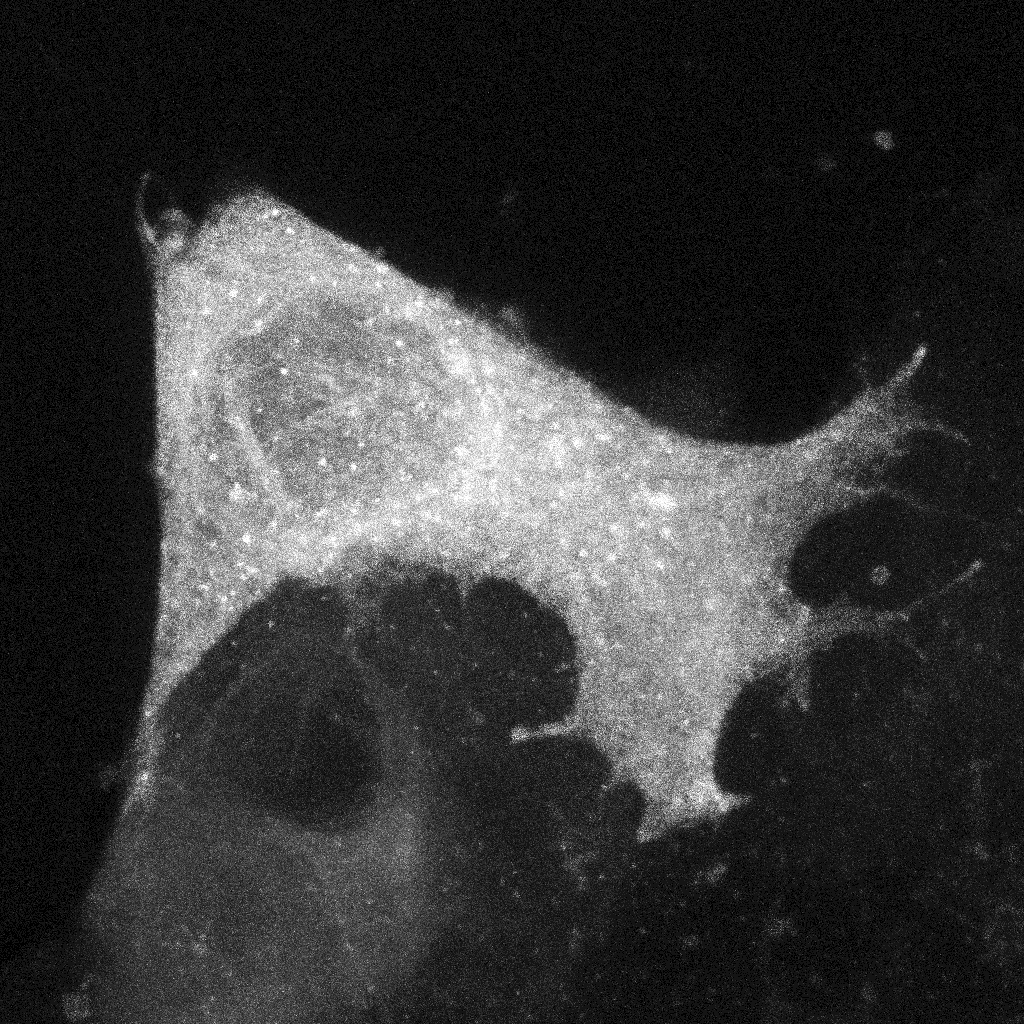

Supplement: Supplementary file 8 — Source data Fig. 6 [file 44319_2024_206_MOESM8_ESM.zip › Figure 6/6B/EYFP-ER/6B_BCL2_LVI_AAA_mTurq2.tif]
