# Supplementary material for: BCL-2 and BOK regulate apoptosis by interaction of their C-terminal transmembrane domains
Source: EMBO Rep. 2024 Jul 24;25(9):12. doi: 10.1038/s44319-024-00206-6 (PMC11387410; doi:10.1038/s44319-024-00206-6)
Supplement: Supplementary file 8 — Source data Fig. 6 [file 44319_2024_206_MOESM8_ESM.zip › Figure 6/6C/6C_western_annotated.pptx]

## Slide 1
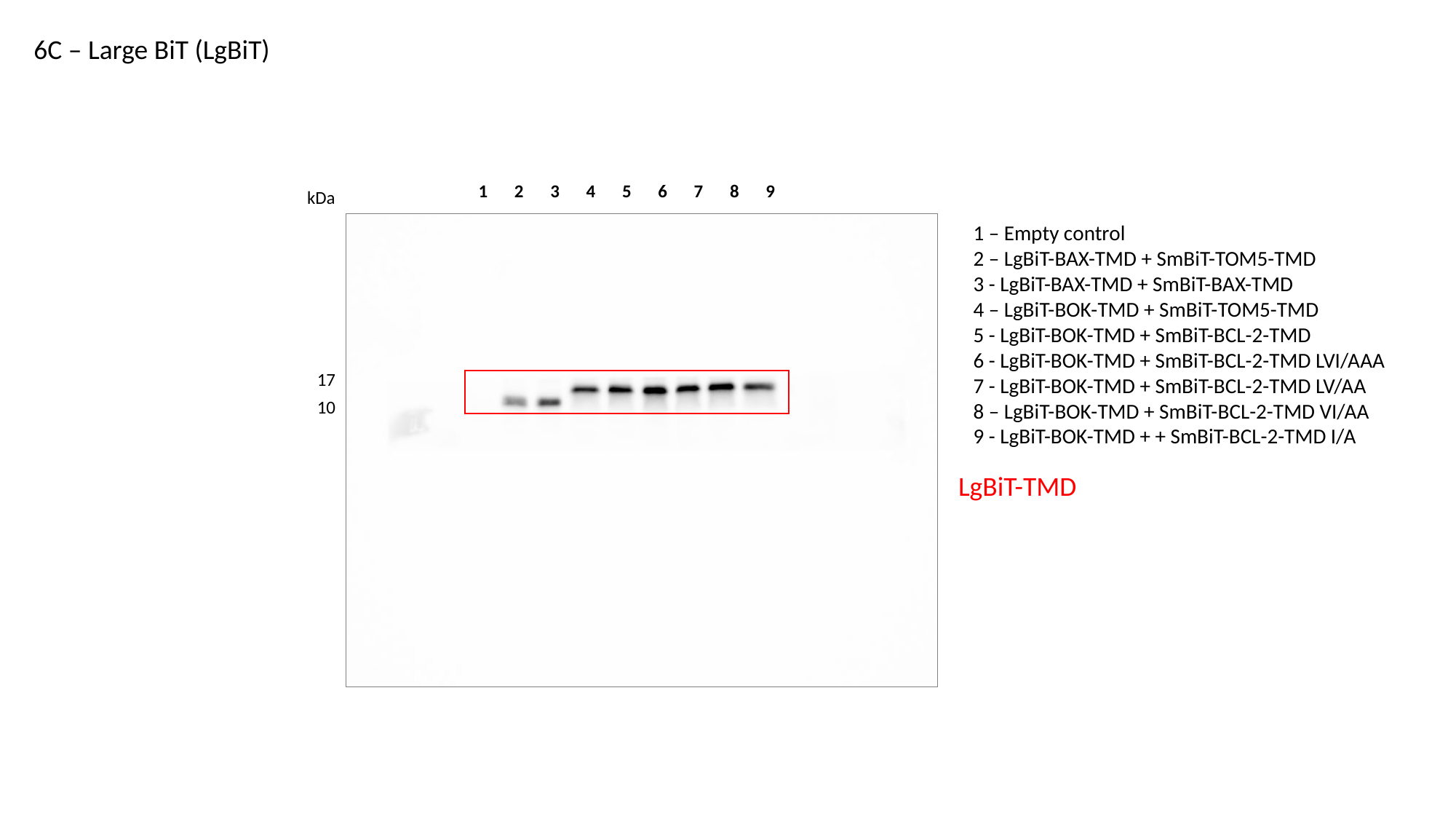

6C – Large BiT (LgBiT)
| 1 | 2 | 3 | 4 | 5 | 6 | 7 | 8 | 9 |
| --- | --- | --- | --- | --- | --- | --- | --- | --- |
kDa
1 – Empty control
2 – LgBiT-BAX-TMD + SmBiT-TOM5-TMD
3 - LgBiT-BAX-TMD + SmBiT-BAX-TMD
4 – LgBiT-BOK-TMD + SmBiT-TOM5-TMD
5 - LgBiT-BOK-TMD + SmBiT-BCL-2-TMD
6 - LgBiT-BOK-TMD + SmBiT-BCL-2-TMD LVI/AAA
7 - LgBiT-BOK-TMD + SmBiT-BCL-2-TMD LV/AA
8 – LgBiT-BOK-TMD + SmBiT-BCL-2-TMD VI/AA
9 - LgBiT-BOK-TMD + + SmBiT-BCL-2-TMD I/A
17
10
LgBiT-TMD

## Slide 2
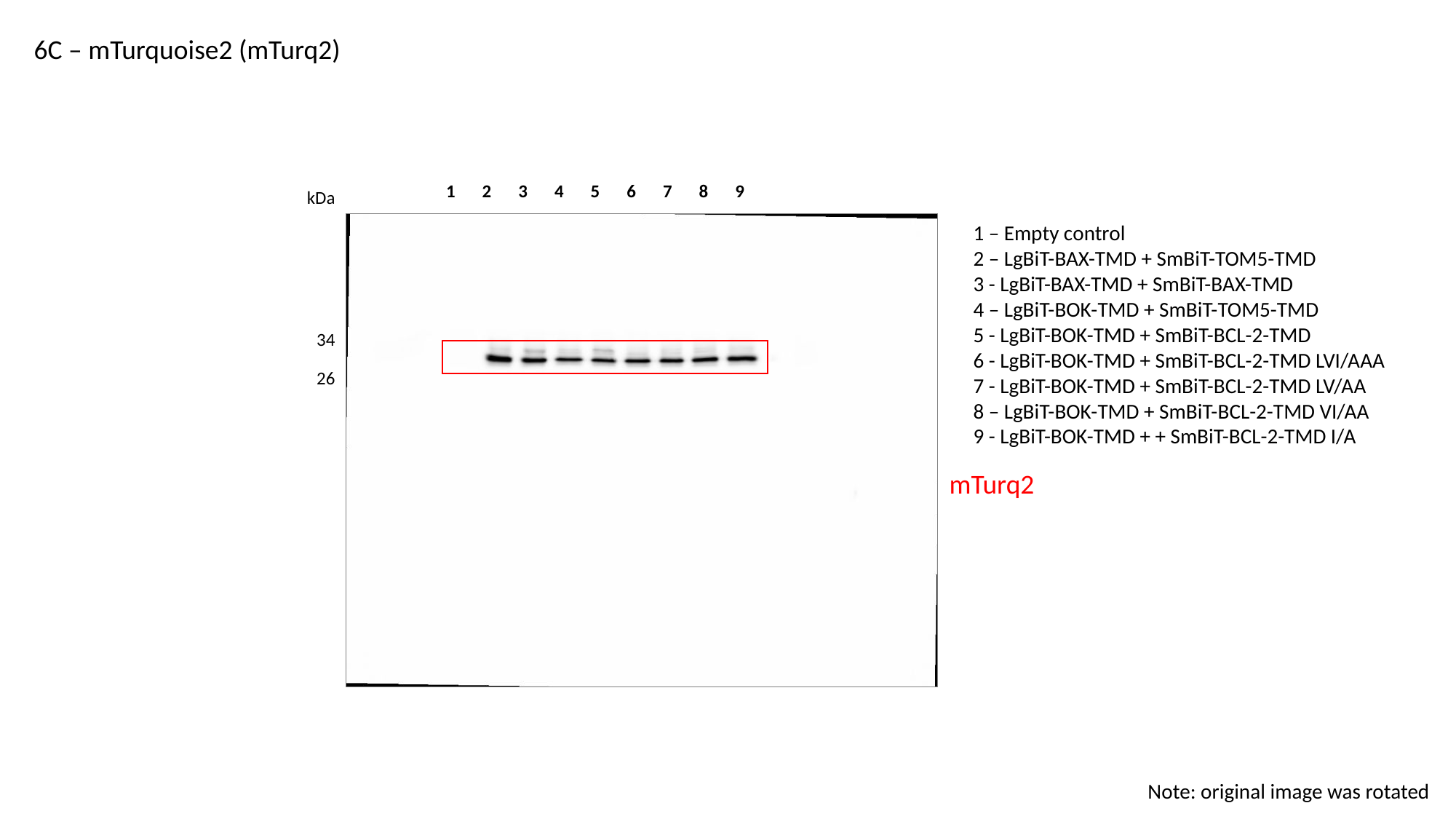

6C – mTurquoise2 (mTurq2)
| 1 | 2 | 3 | 4 | 5 | 6 | 7 | 8 | 9 |
| --- | --- | --- | --- | --- | --- | --- | --- | --- |
kDa
1 – Empty control
2 – LgBiT-BAX-TMD + SmBiT-TOM5-TMD
3 - LgBiT-BAX-TMD + SmBiT-BAX-TMD
4 – LgBiT-BOK-TMD + SmBiT-TOM5-TMD
5 - LgBiT-BOK-TMD + SmBiT-BCL-2-TMD
6 - LgBiT-BOK-TMD + SmBiT-BCL-2-TMD LVI/AAA
7 - LgBiT-BOK-TMD + SmBiT-BCL-2-TMD LV/AA
8 – LgBiT-BOK-TMD + SmBiT-BCL-2-TMD VI/AA
9 - LgBiT-BOK-TMD + + SmBiT-BCL-2-TMD I/A
34
26
mTurq2
Note: original image was rotated

## Slide 3
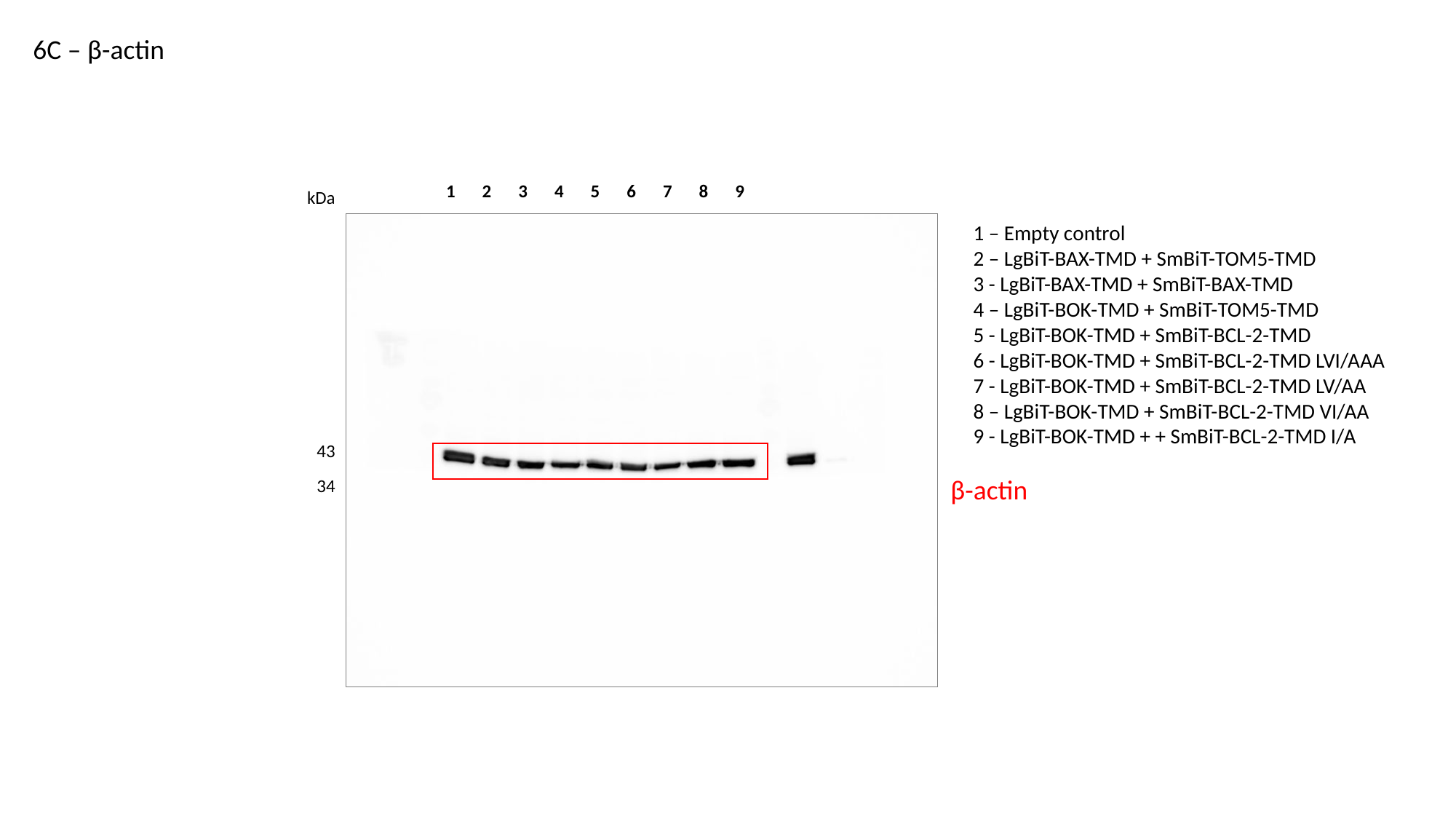

6C – β-actin
| 1 | 2 | 3 | 4 | 5 | 6 | 7 | 8 | 9 |
| --- | --- | --- | --- | --- | --- | --- | --- | --- |
kDa
1 – Empty control
2 – LgBiT-BAX-TMD + SmBiT-TOM5-TMD
3 - LgBiT-BAX-TMD + SmBiT-BAX-TMD
4 – LgBiT-BOK-TMD + SmBiT-TOM5-TMD
5 - LgBiT-BOK-TMD + SmBiT-BCL-2-TMD
6 - LgBiT-BOK-TMD + SmBiT-BCL-2-TMD LVI/AAA
7 - LgBiT-BOK-TMD + SmBiT-BCL-2-TMD LV/AA
8 – LgBiT-BOK-TMD + SmBiT-BCL-2-TMD VI/AA
9 - LgBiT-BOK-TMD + + SmBiT-BCL-2-TMD I/A
43
β-actin
34
