# Supplementary figures and images for: BCL-2 and BOK regulate apoptosis by interaction of their C-terminal transmembrane domains (part 2 of 2)
Source: EMBO Rep. 2024 Jul 24;25(9):12. doi: 10.1038/s44319-024-00206-6 (PMC11387410; doi:10.1038/s44319-024-00206-6)

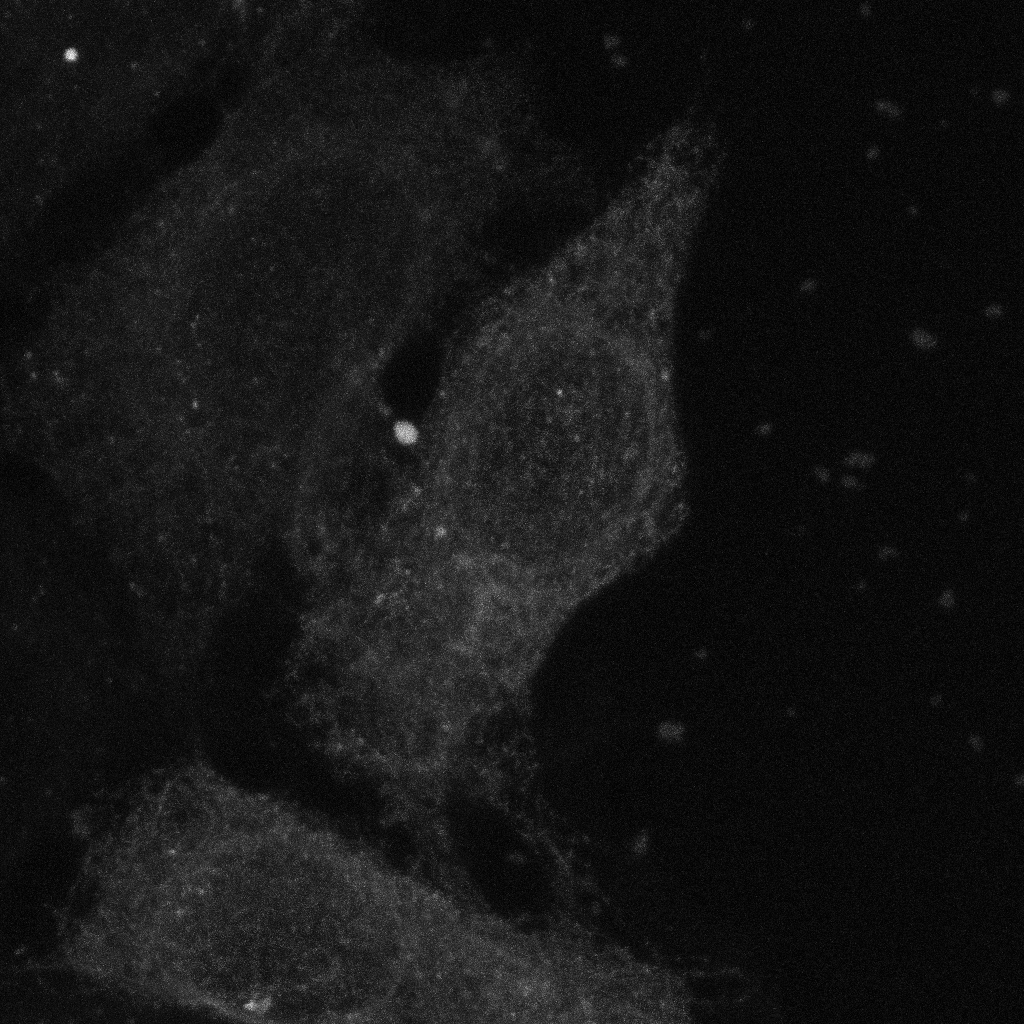

Supplement: Supplementary file 8 — Source data Fig. 6 [file 44319_2024_206_MOESM8_ESM.zip › Figure 6/6B/EYFP-ER/6B_BCL2_LV_AA_EYFP.tif]

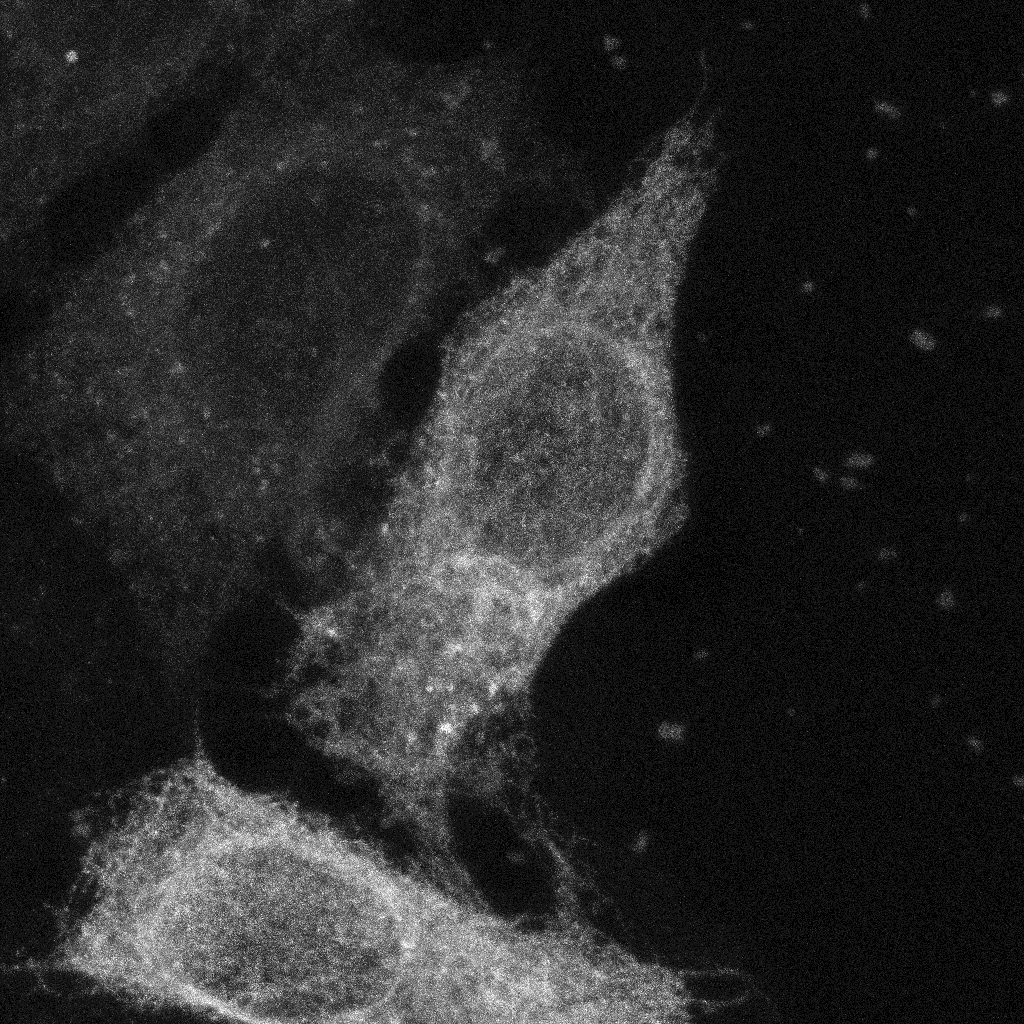

Supplement: Supplementary file 8 — Source data Fig. 6 [file 44319_2024_206_MOESM8_ESM.zip › Figure 6/6B/EYFP-ER/6B_BCL2_LV_AA_mTurq2.tif]

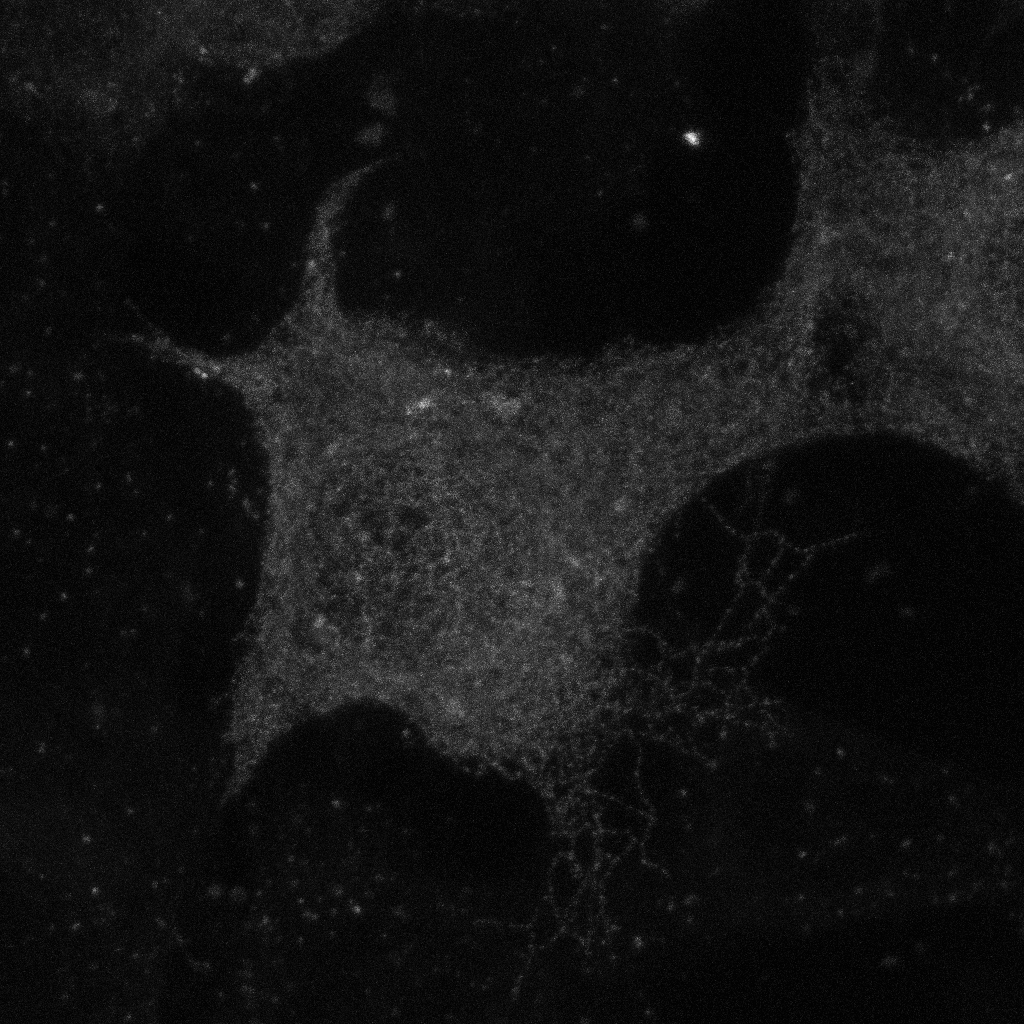

Supplement: Supplementary file 8 — Source data Fig. 6 [file 44319_2024_206_MOESM8_ESM.zip › Figure 6/6B/EYFP-ER/6B_BCL2_VI_AA_EYFP.tif]

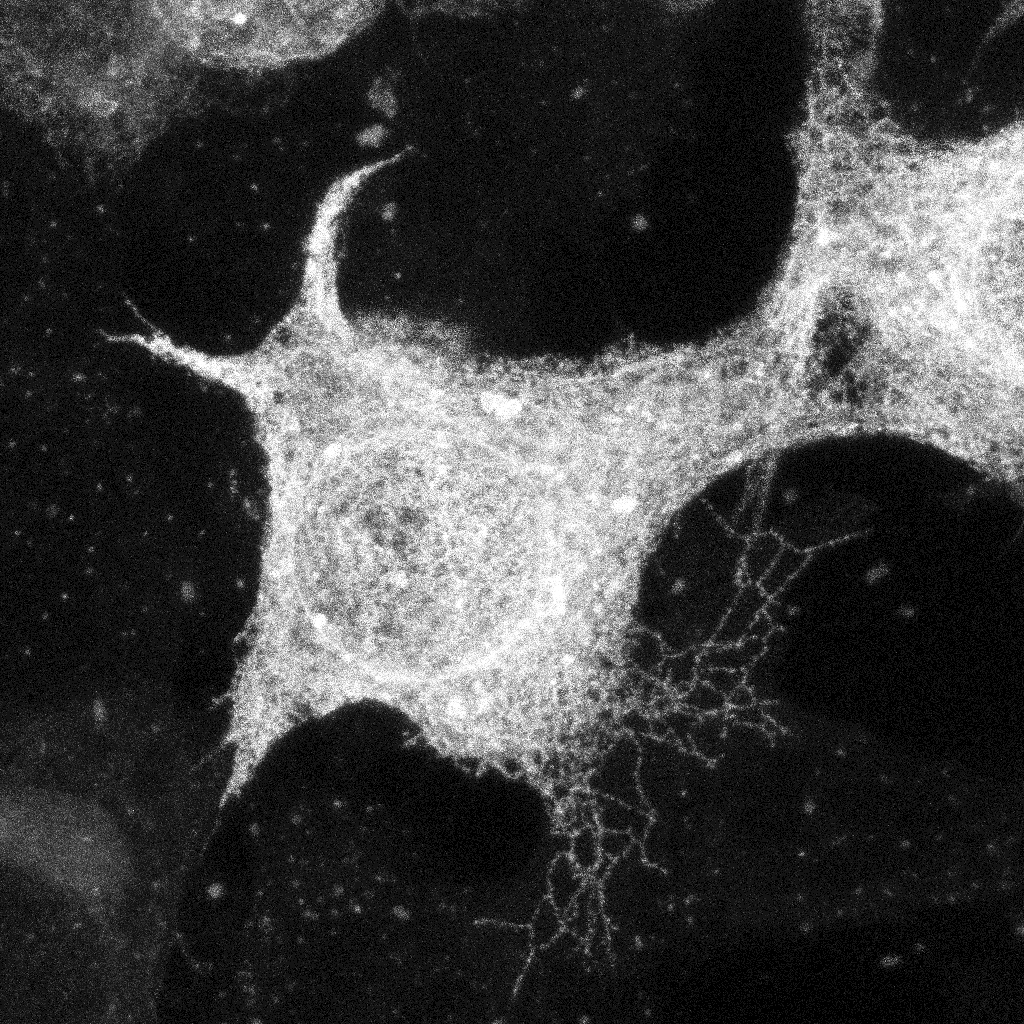

Supplement: Supplementary file 8 — Source data Fig. 6 [file 44319_2024_206_MOESM8_ESM.zip › Figure 6/6B/EYFP-ER/6B_BCL2_VI_AA_mTurq2.tif]

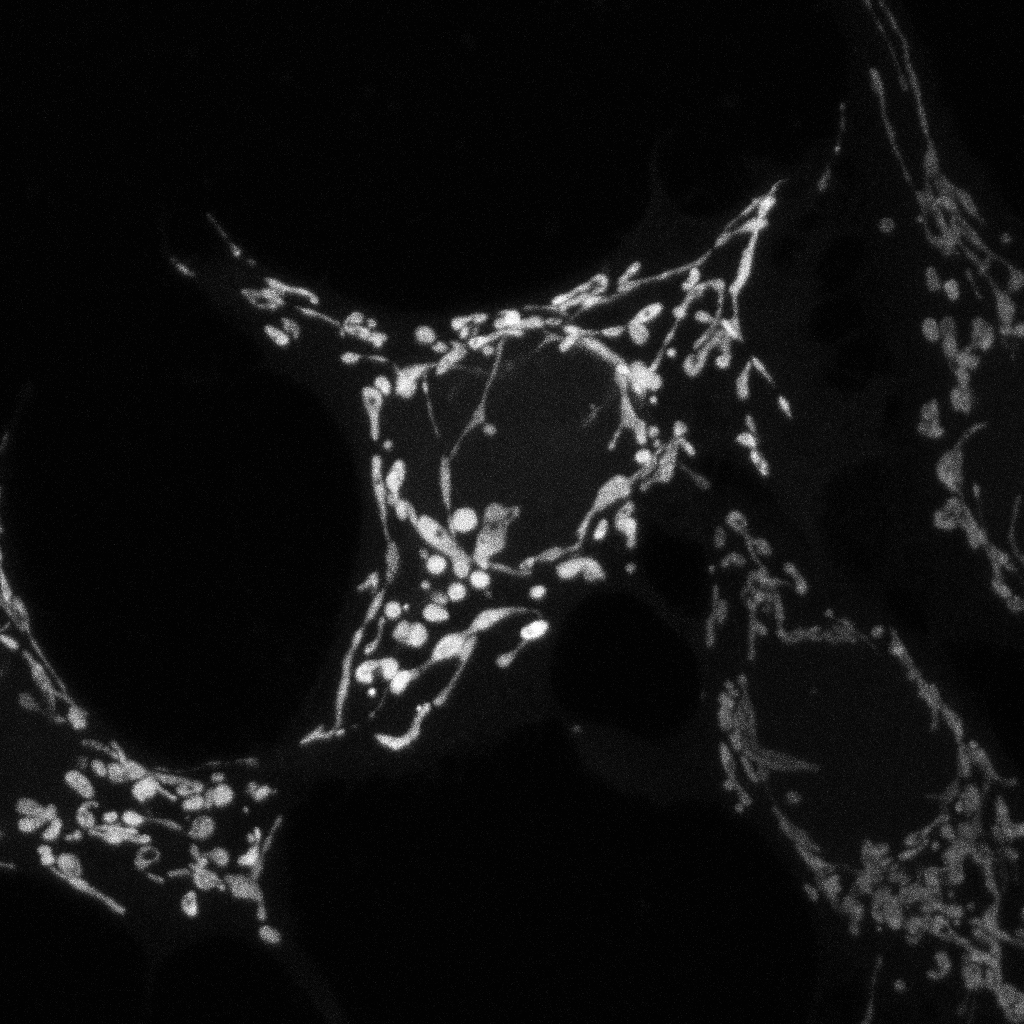

Supplement: Supplementary file 8 — Source data Fig. 6 [file 44319_2024_206_MOESM8_ESM.zip › Figure 6/6B/EYFP-Mito/6B_BCL2_I_A_EYFP.tif]

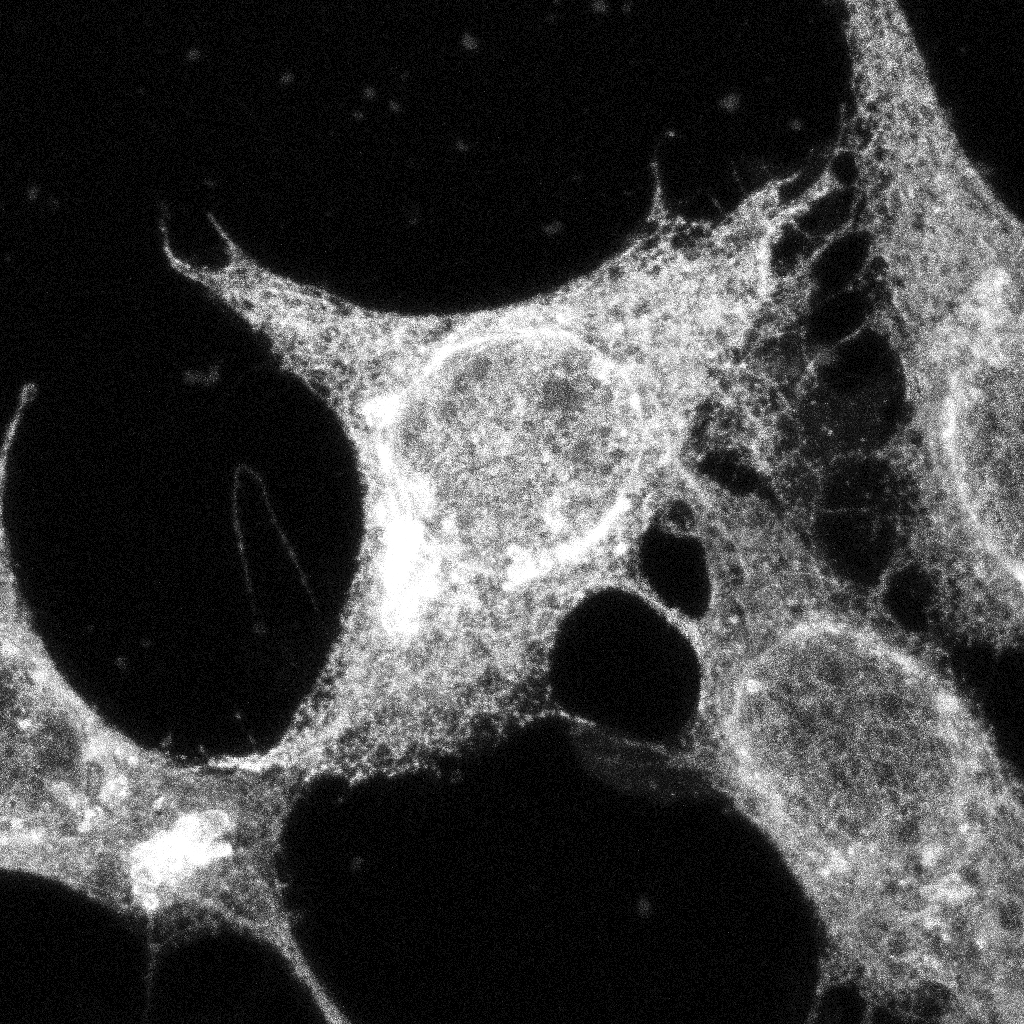

Supplement: Supplementary file 8 — Source data Fig. 6 [file 44319_2024_206_MOESM8_ESM.zip › Figure 6/6B/EYFP-Mito/6B_BCL2_I_A_mTurq2.tif]

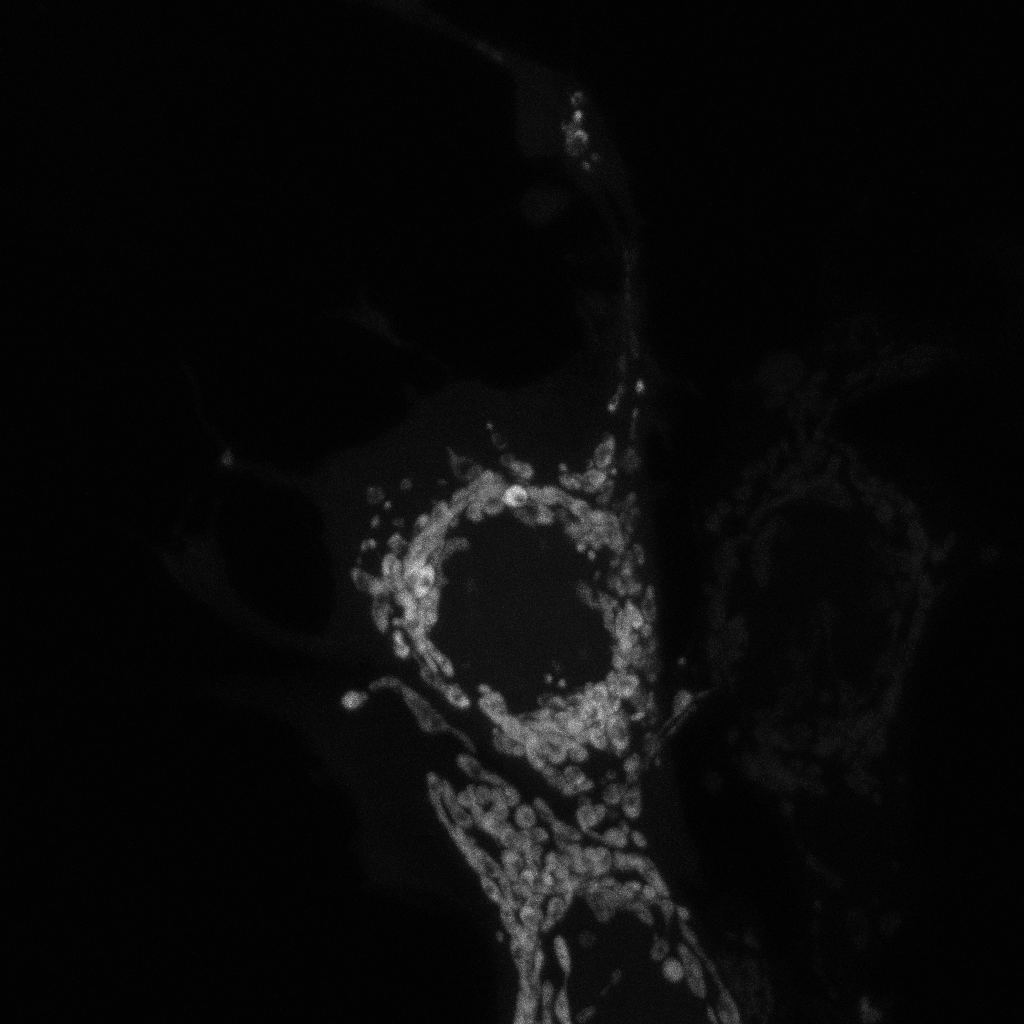

Supplement: Supplementary file 8 — Source data Fig. 6 [file 44319_2024_206_MOESM8_ESM.zip › Figure 6/6B/EYFP-Mito/6B_BCL2_LVI_AAA_EYFP.tif]

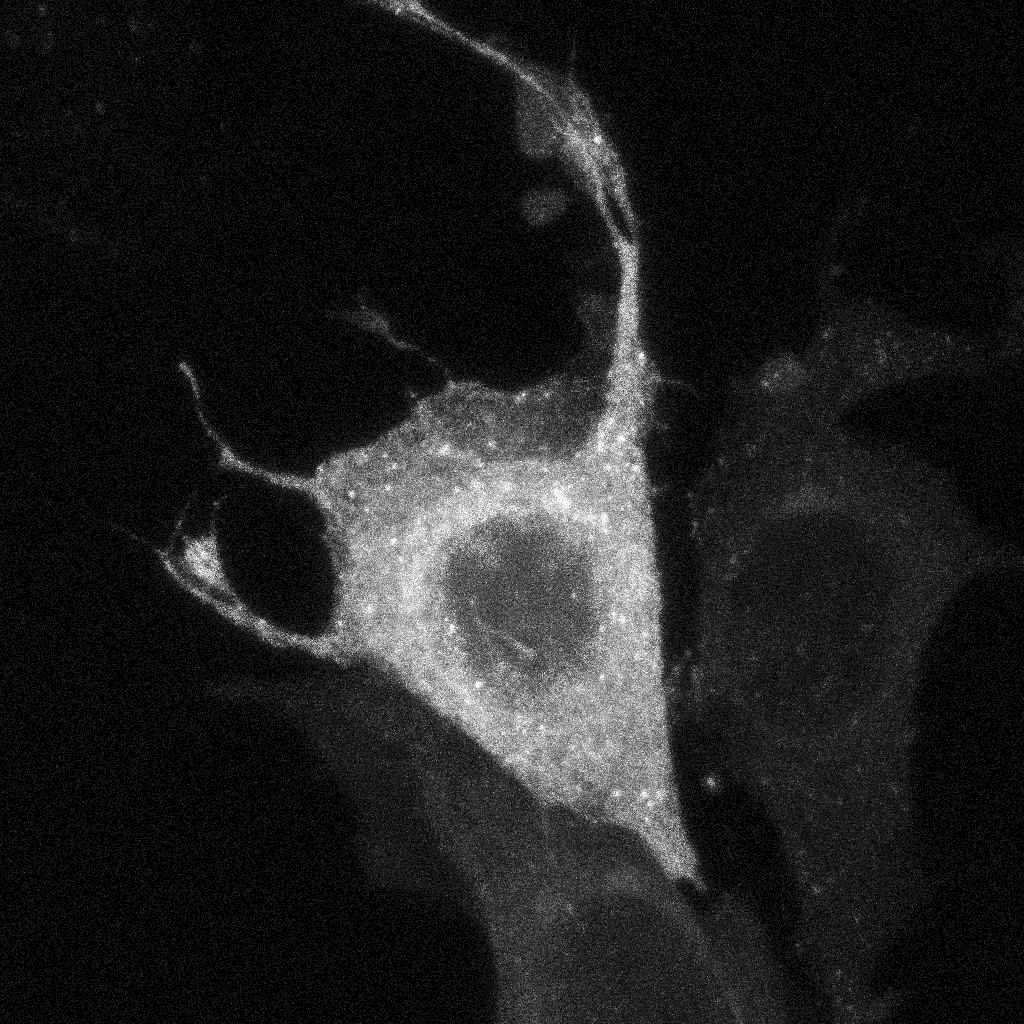

Supplement: Supplementary file 8 — Source data Fig. 6 [file 44319_2024_206_MOESM8_ESM.zip › Figure 6/6B/EYFP-Mito/6B_BCL2_LVI_AAA_mTurq2.tif]

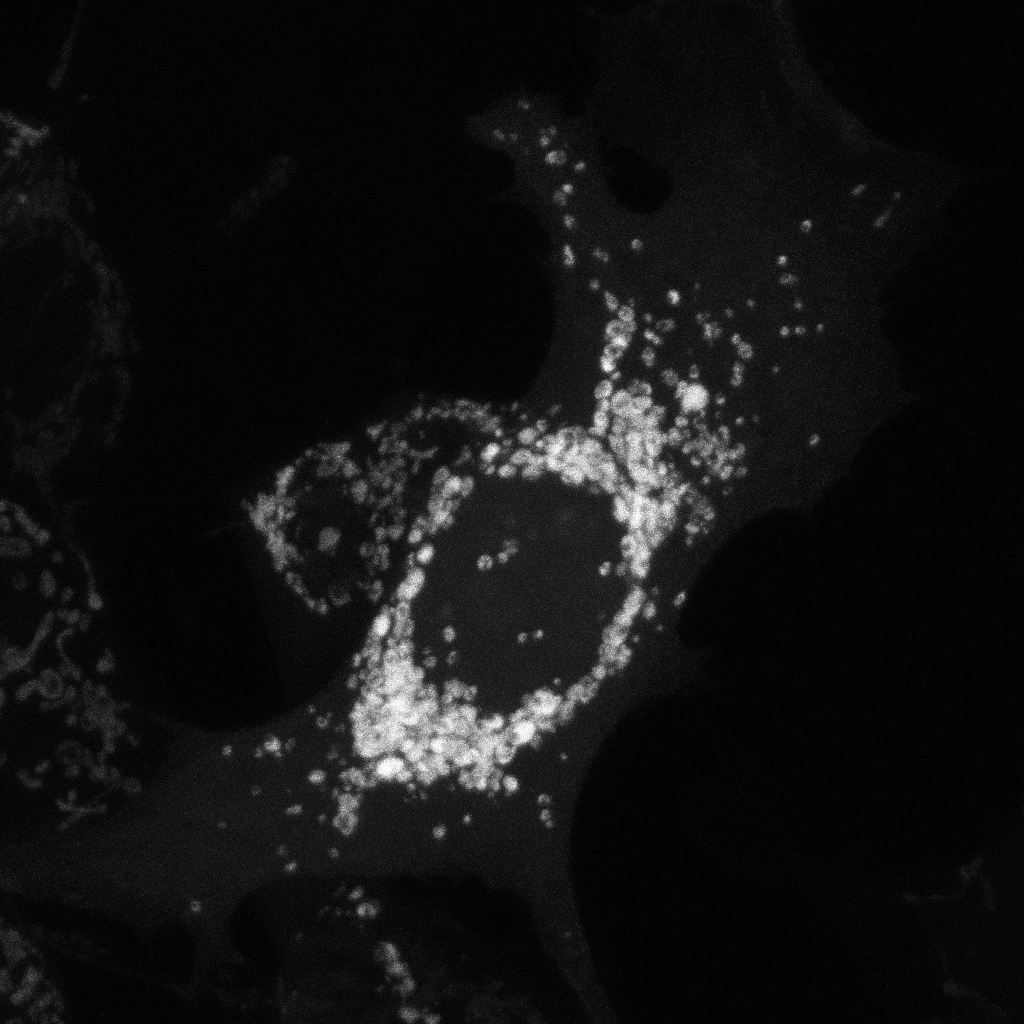

Supplement: Supplementary file 8 — Source data Fig. 6 [file 44319_2024_206_MOESM8_ESM.zip › Figure 6/6B/EYFP-Mito/6B_BCL2_LV_AA_EYFP.tif]

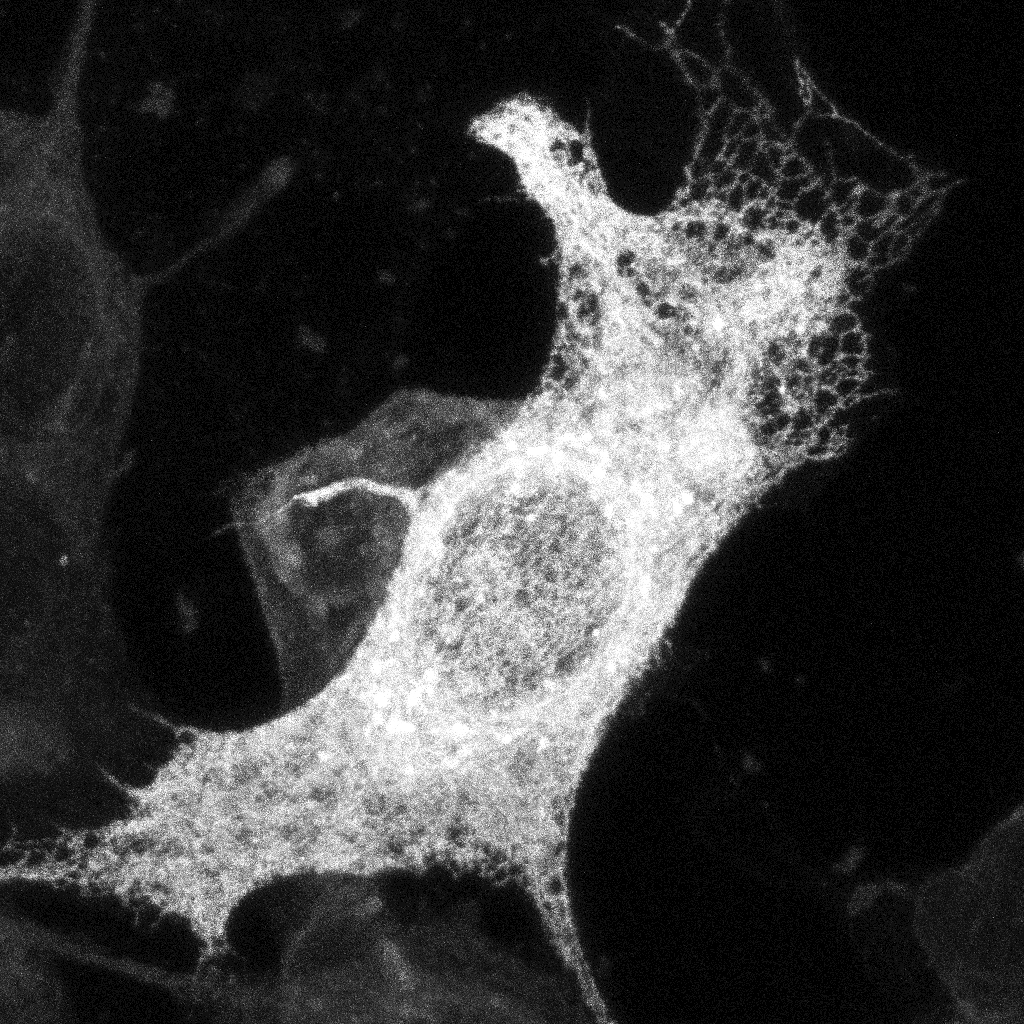

Supplement: Supplementary file 8 — Source data Fig. 6 [file 44319_2024_206_MOESM8_ESM.zip › Figure 6/6B/EYFP-Mito/6B_BCL2_LV_AA_mTurq2.tif]

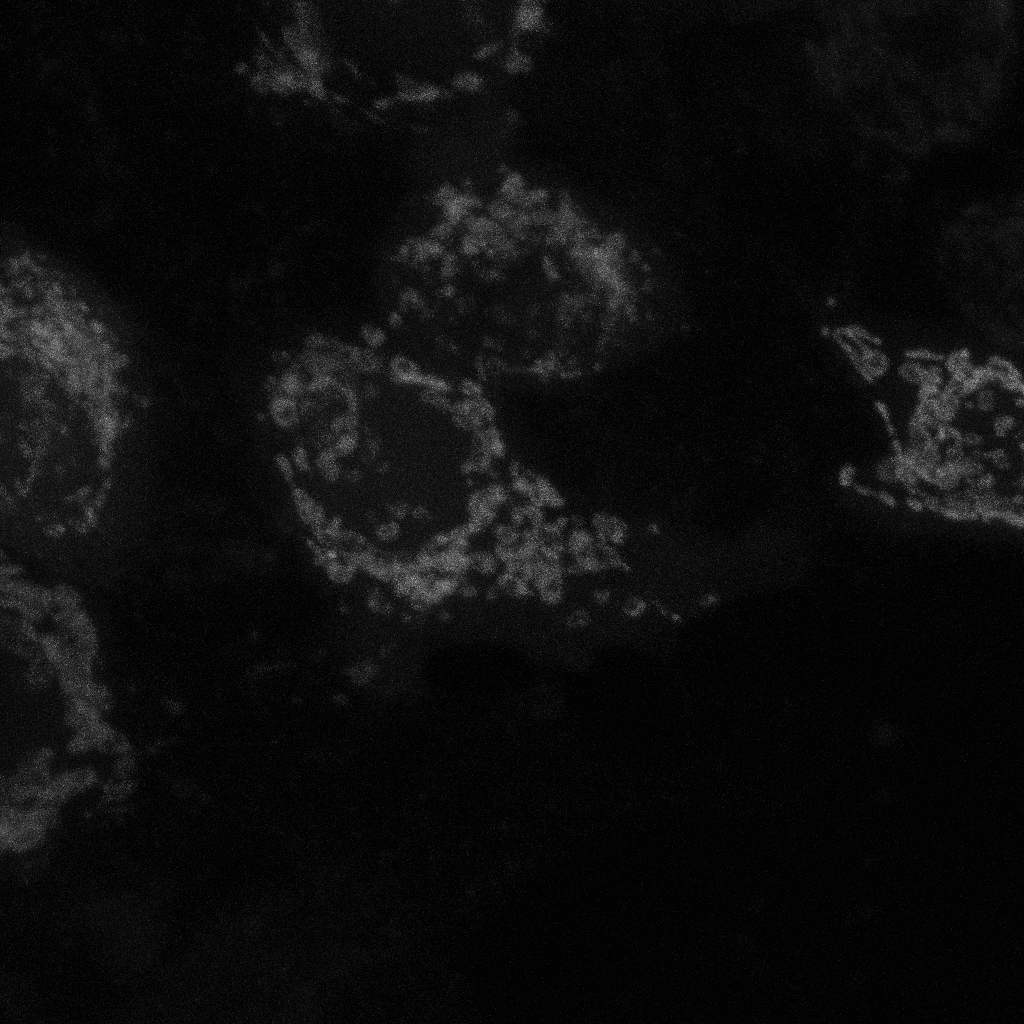

Supplement: Supplementary file 8 — Source data Fig. 6 [file 44319_2024_206_MOESM8_ESM.zip › Figure 6/6B/EYFP-Mito/6B_BCL2_VI_AA_EYFP.tif]

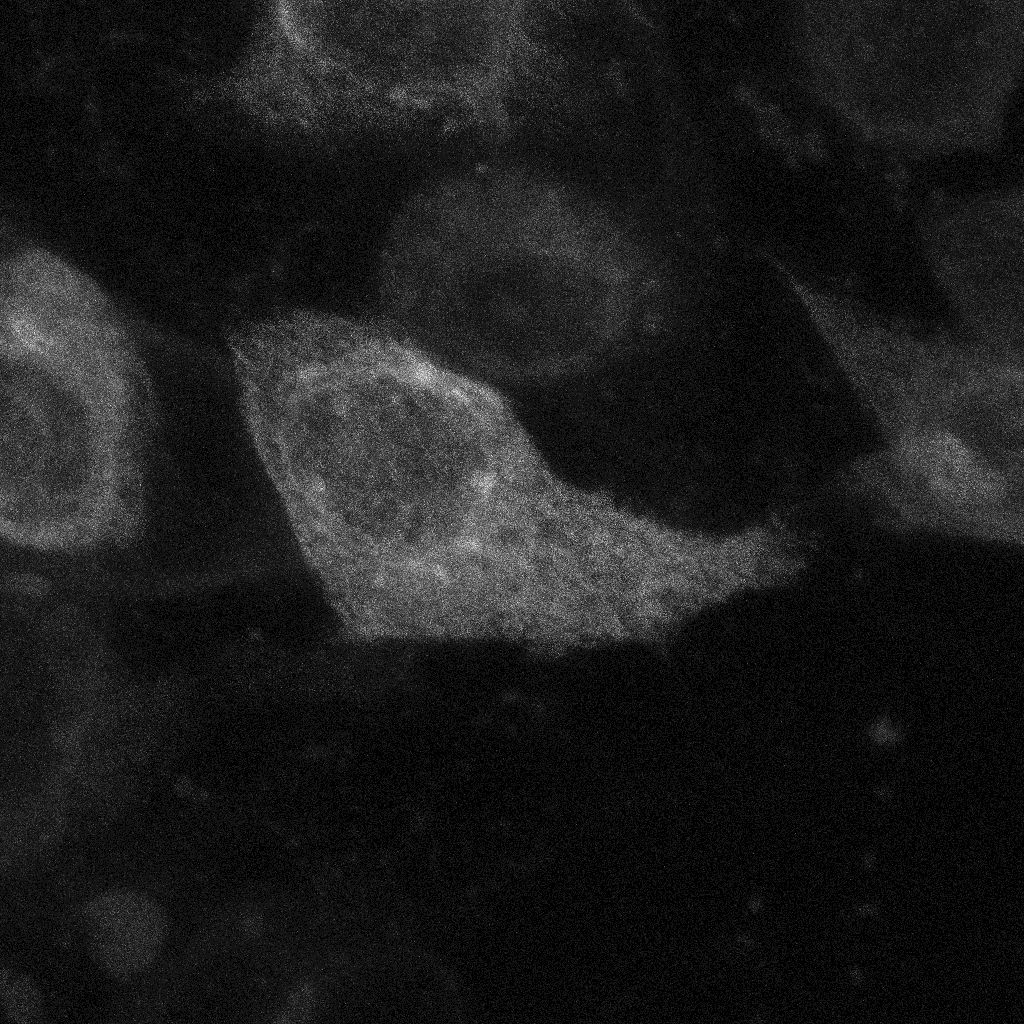

Supplement: Supplementary file 8 — Source data Fig. 6 [file 44319_2024_206_MOESM8_ESM.zip › Figure 6/6B/EYFP-Mito/6B_BCL2_VI_AA_mTurq2.tif]

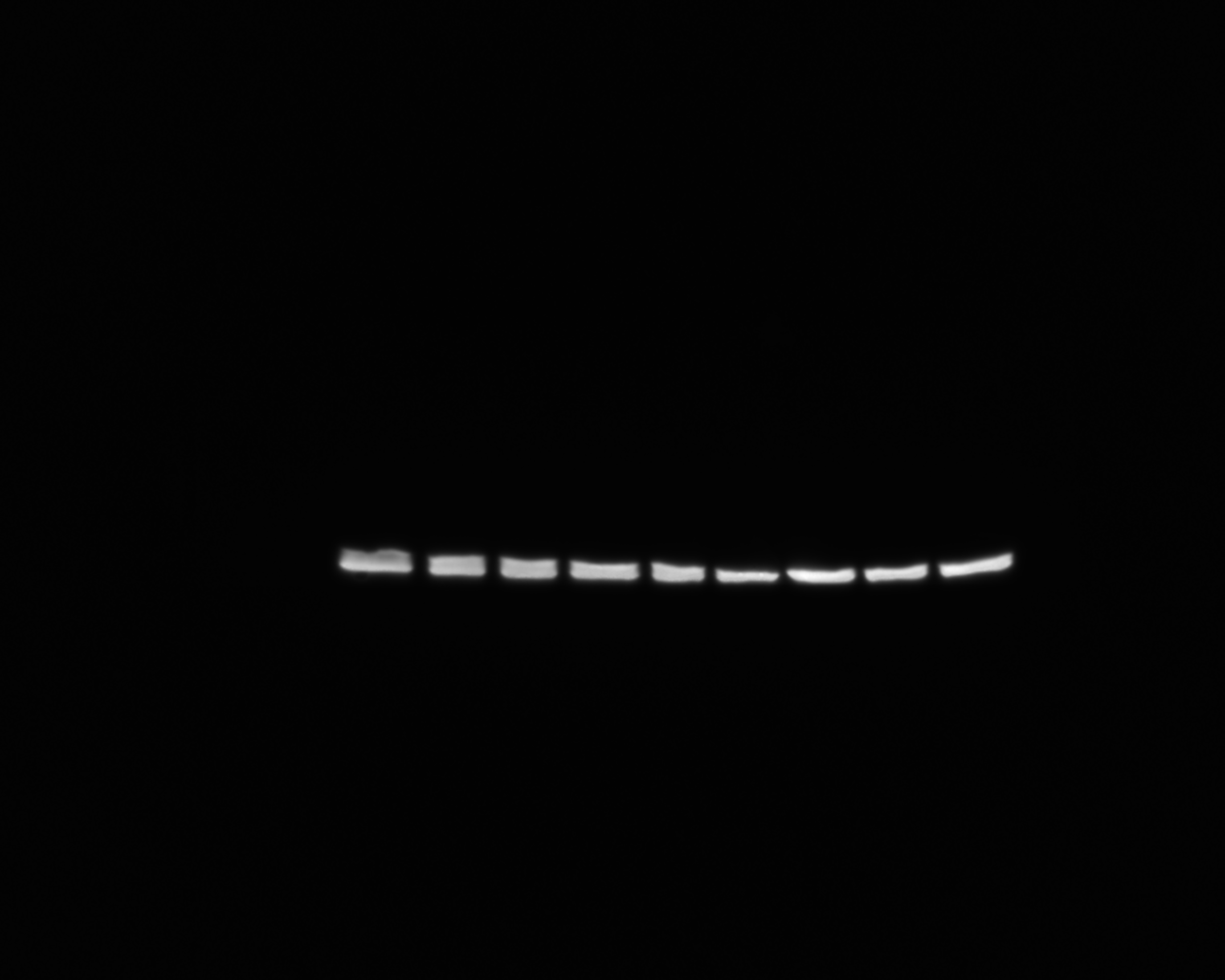

Supplement: Supplementary file 8 — Source data Fig. 6 [file 44319_2024_206_MOESM8_ESM.zip › Figure 6/6C/6C_western_betaActin.tif]

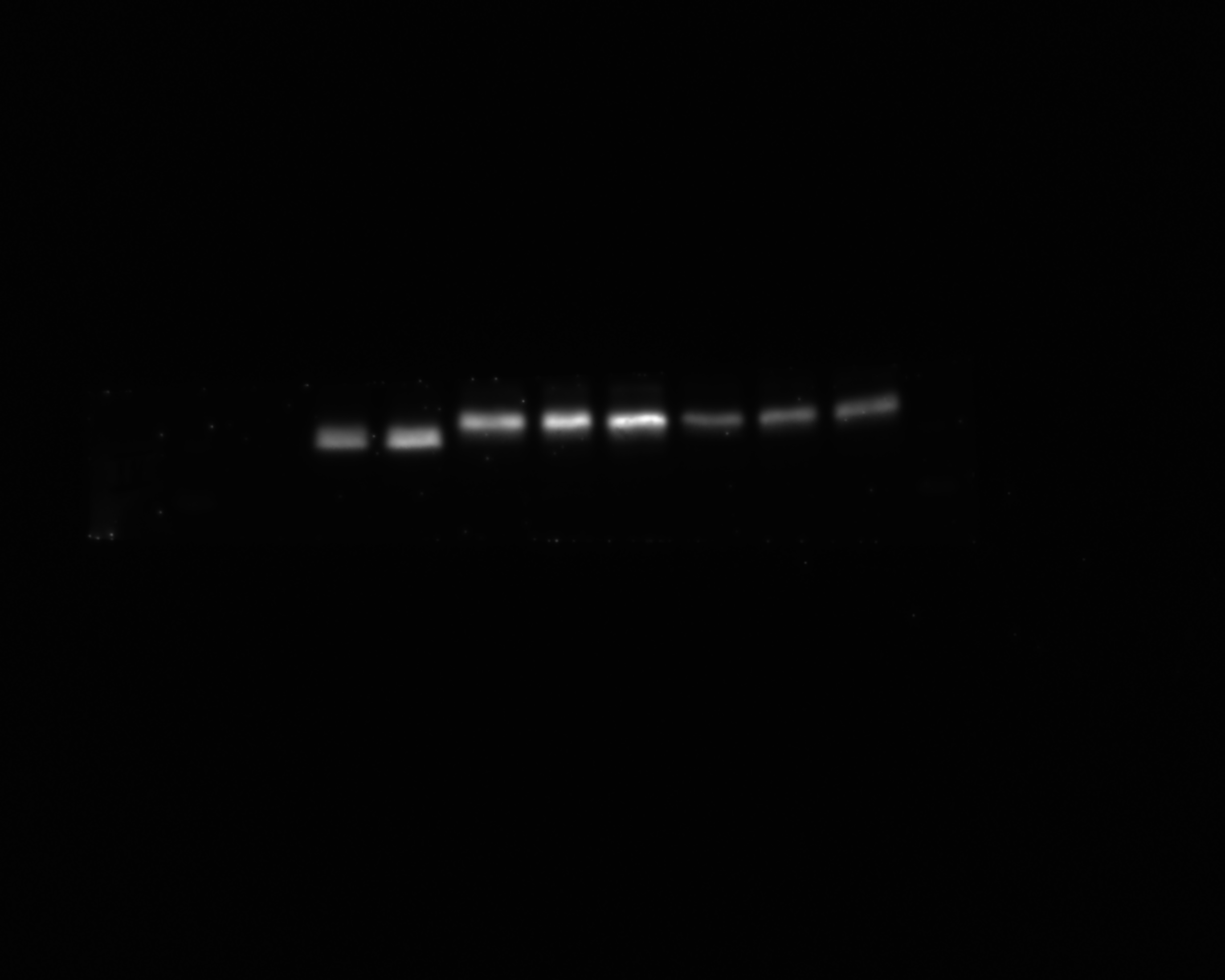

Supplement: Supplementary file 8 — Source data Fig. 6 [file 44319_2024_206_MOESM8_ESM.zip › Figure 6/6C/6C_western_LgBiT.tif]

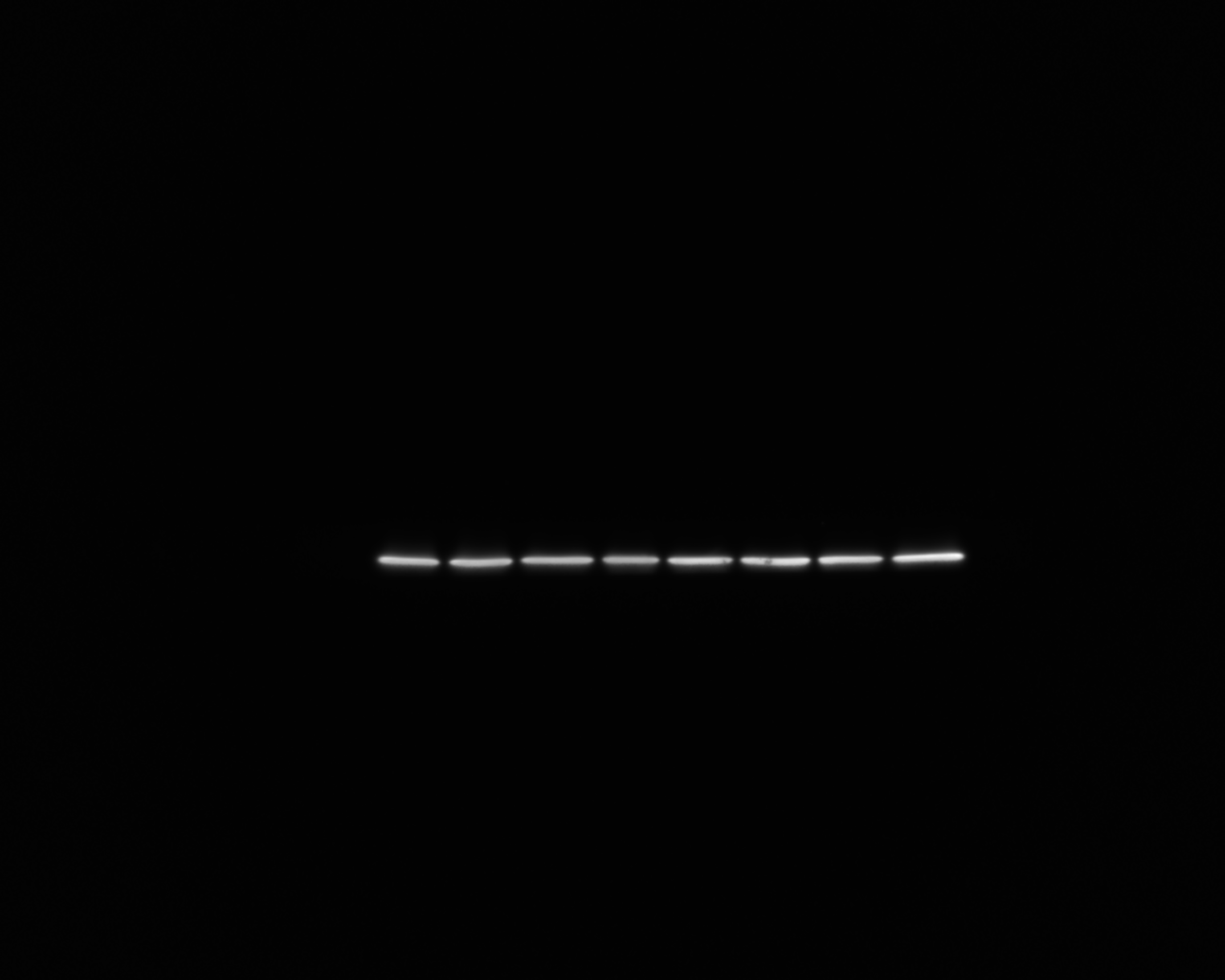

Supplement: Supplementary file 8 — Source data Fig. 6 [file 44319_2024_206_MOESM8_ESM.zip › Figure 6/6C/6C_western_mTurq2.tif]

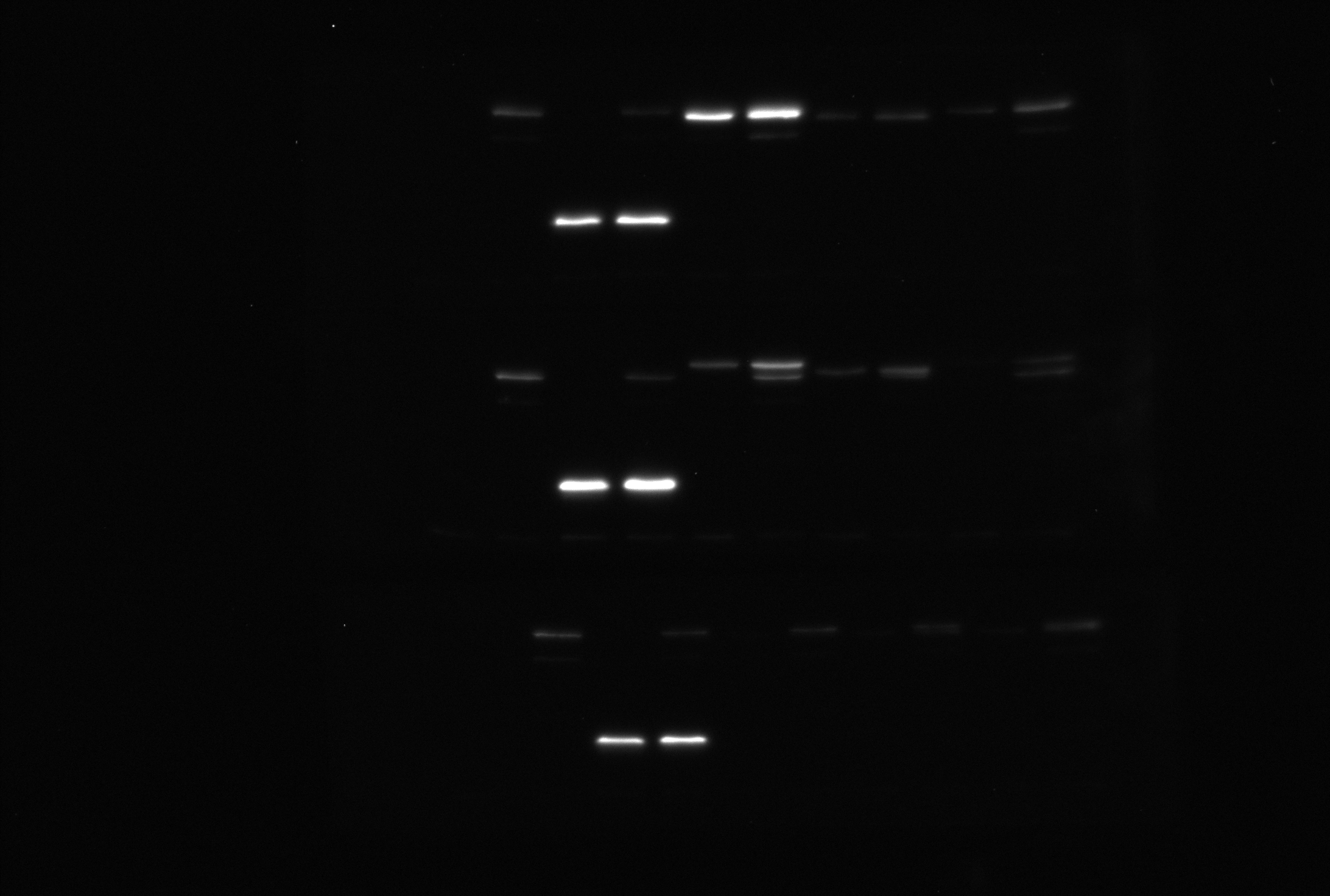

Supplement: Supplementary file 9 — Source data Fig. 7 [file 44319_2024_206_MOESM9_ESM.zip › Figure 7/7C/7C_BCL2.tif]

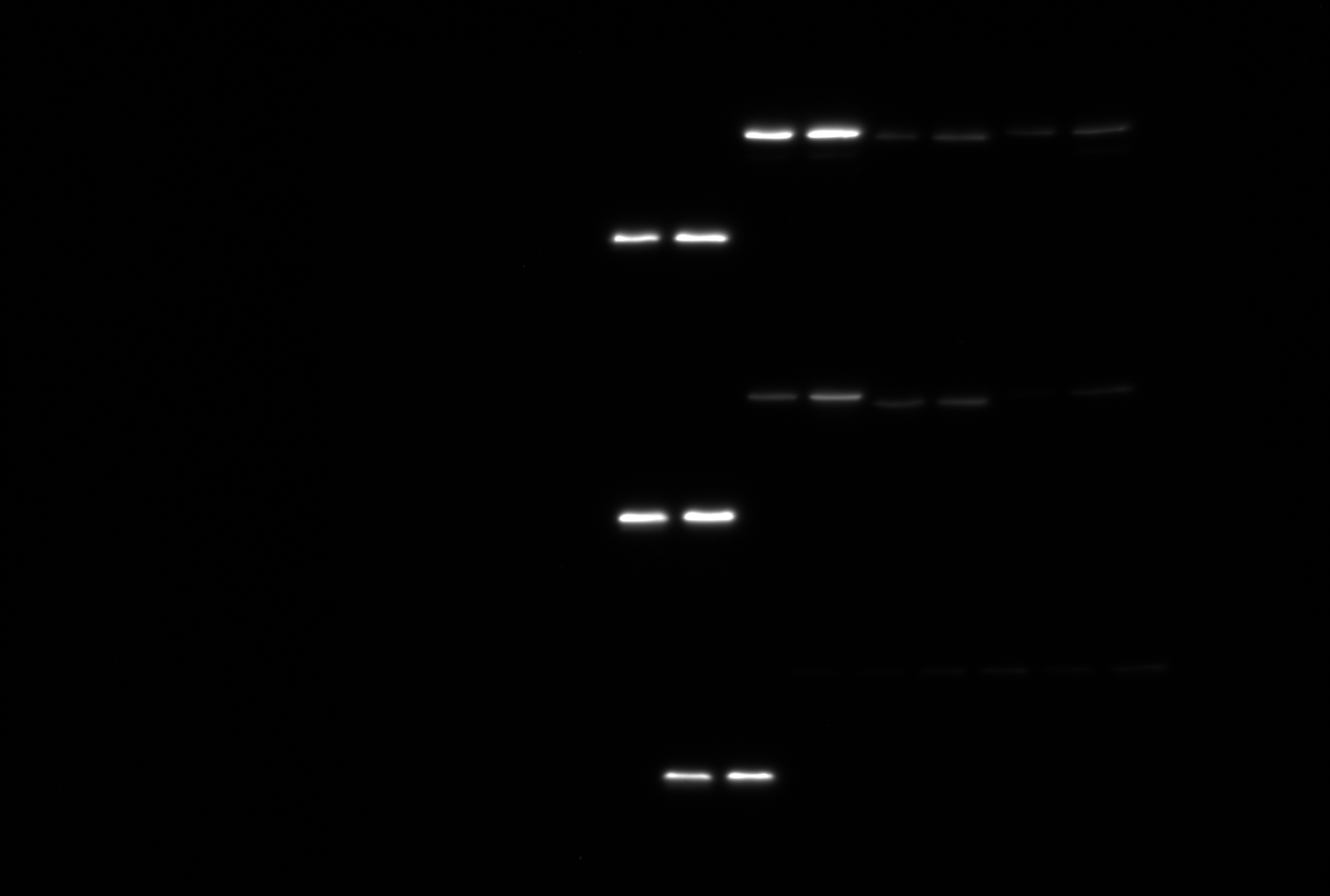

Supplement: Supplementary file 9 — Source data Fig. 7 [file 44319_2024_206_MOESM9_ESM.zip › Figure 7/7C/7C_EGFP.tif]

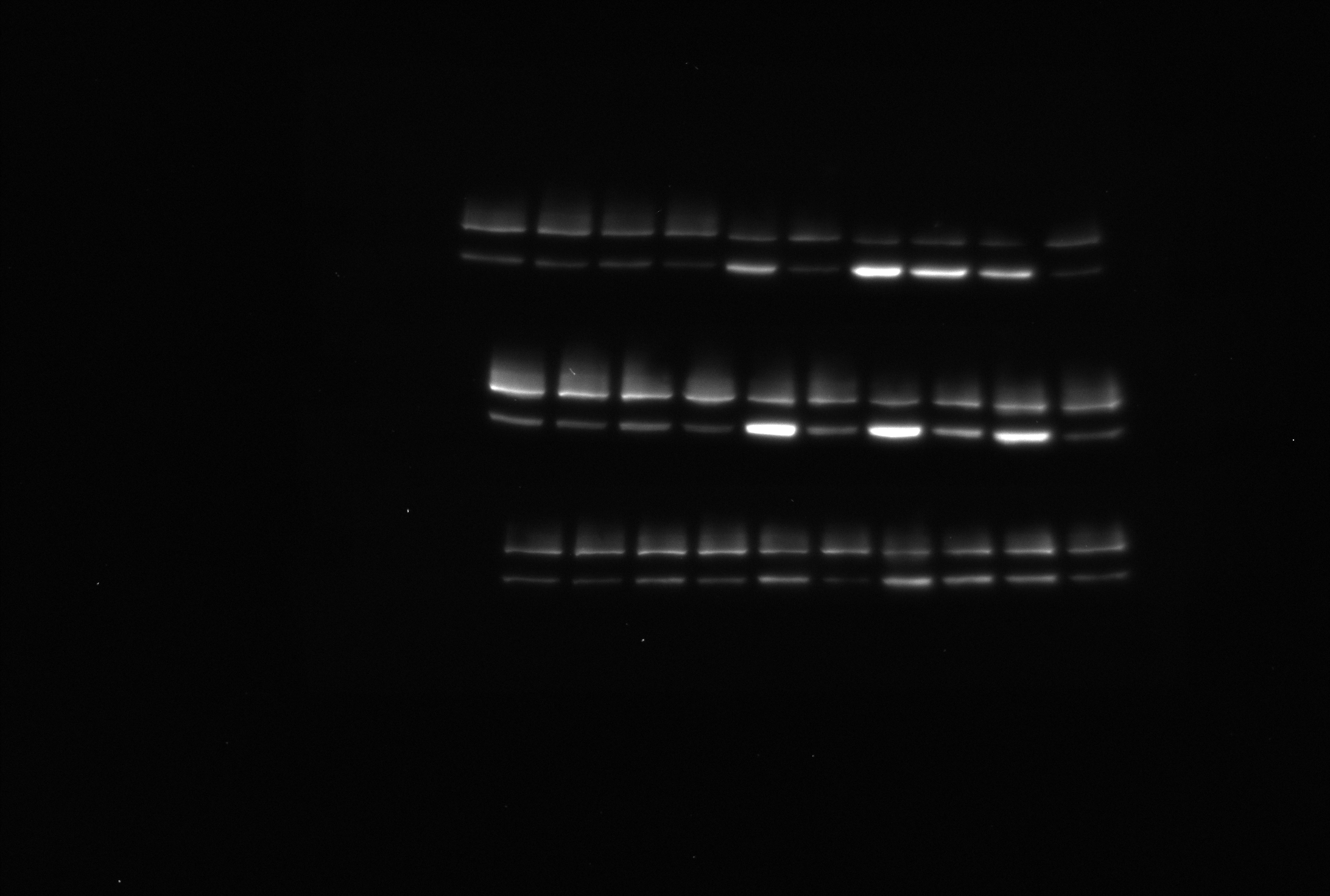

Supplement: Supplementary file 9 — Source data Fig. 7 [file 44319_2024_206_MOESM9_ESM.zip › Figure 7/7C/7C_PARP.tif]

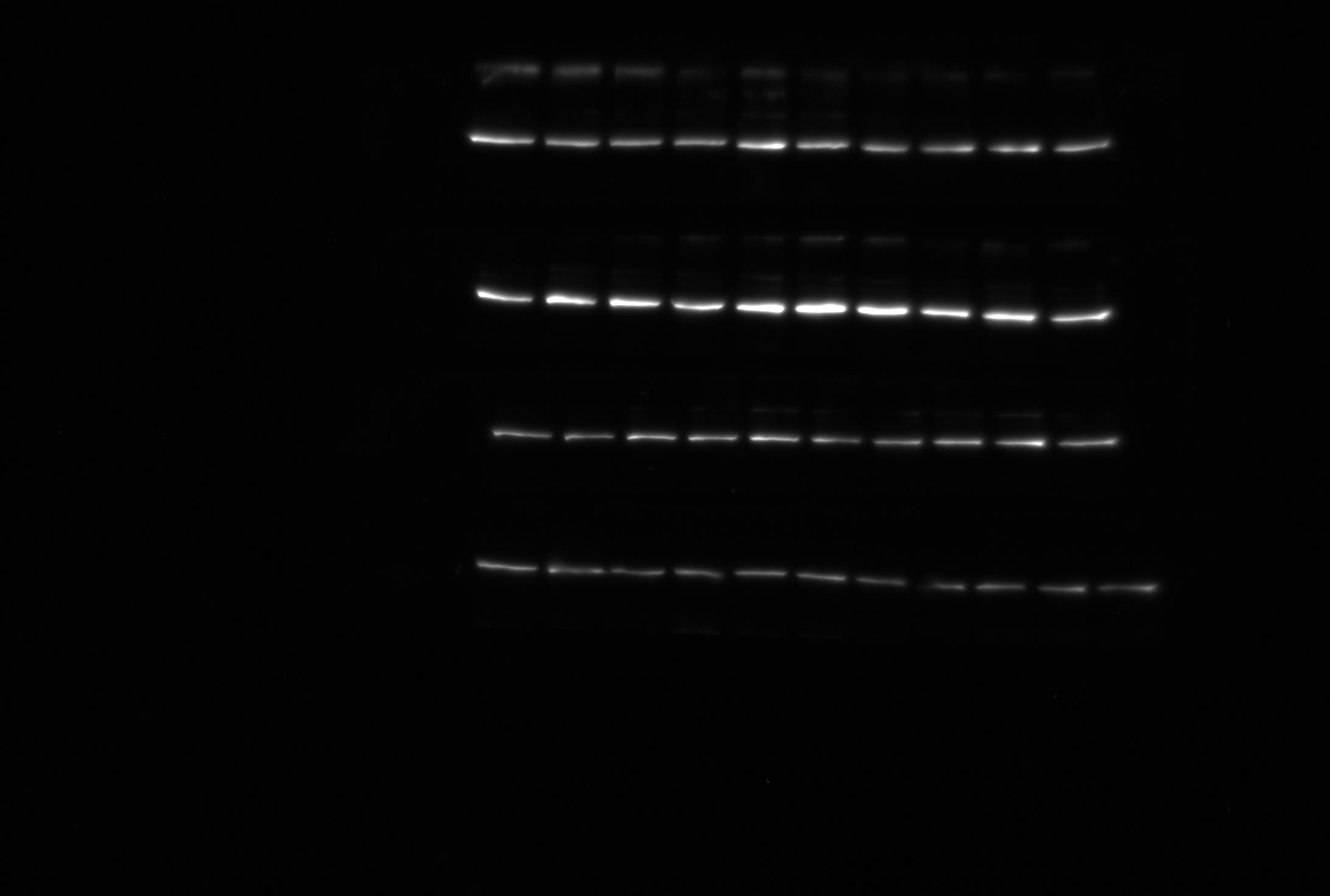

Supplement: Supplementary file 9 — Source data Fig. 7 [file 44319_2024_206_MOESM9_ESM.zip › Figure 7/7C/7C_Vinculin.tif]
