# Supplementary material for: BCL-2 and BOK regulate apoptosis by interaction of their C-terminal transmembrane domains
Source: EMBO Rep. 2024 Jul 24;25(9):12. doi: 10.1038/s44319-024-00206-6 (PMC11387410; doi:10.1038/s44319-024-00206-6)
Supplement: Supplementary file 9 — Source data Fig. 7 [file 44319_2024_206_MOESM9_ESM.zip › Figure 7/7C/7C_western_annotated.pptx]

## Slide 1
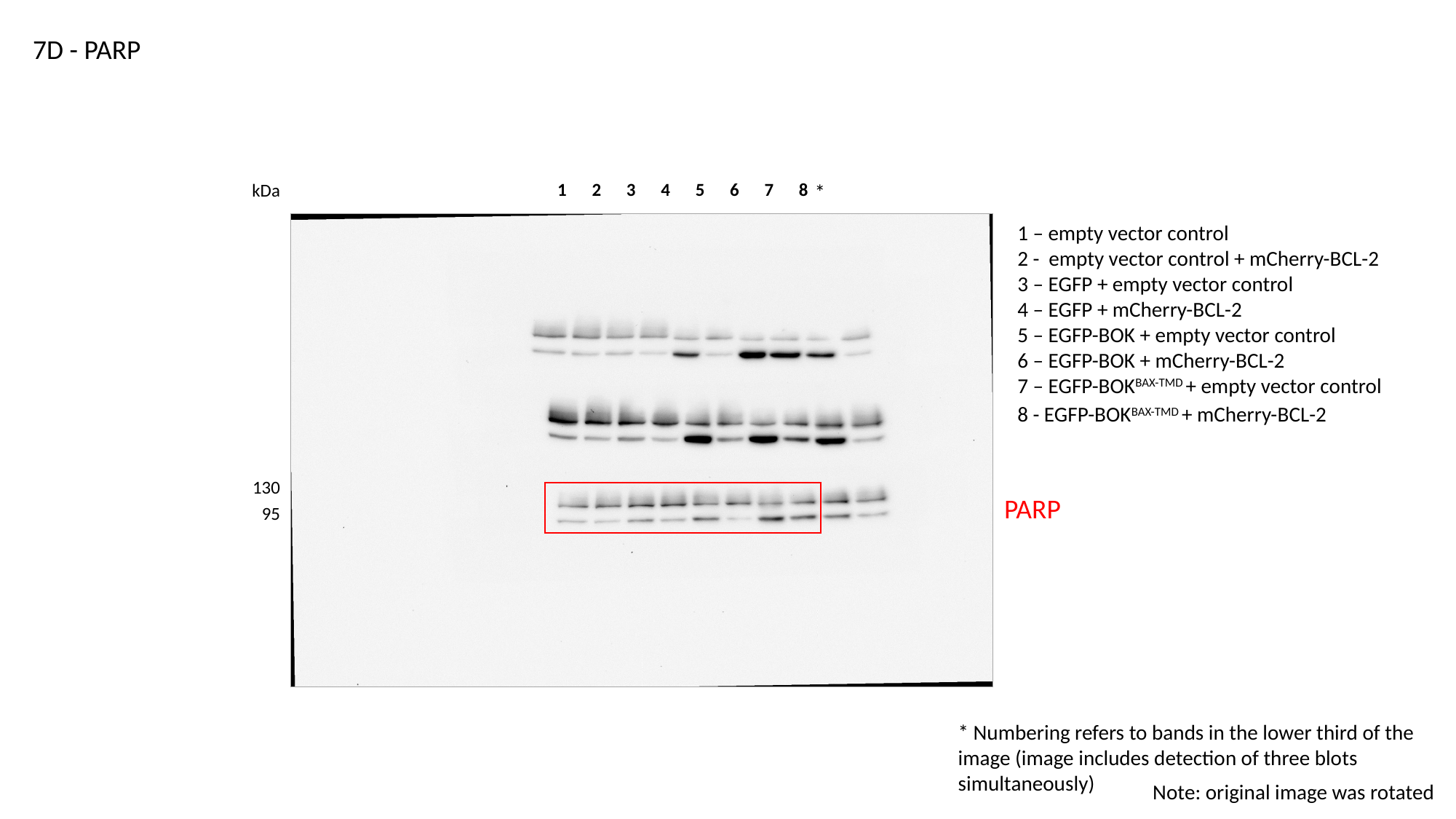

7D - PARP
kDa
*
| 1 | 2 | 3 | 4 | 5 | 6 | 7 | 8 |
| --- | --- | --- | --- | --- | --- | --- | --- |
1 – empty vector control
2 - empty vector control + mCherry-BCL-2
3 – EGFP + empty vector control
4 – EGFP + mCherry-BCL-2
5 – EGFP-BOK + empty vector control
6 – EGFP-BOK + mCherry-BCL-2
7 – EGFP-BOKBAX-TMD + empty vector control
8 - EGFP-BOKBAX-TMD + mCherry-BCL-2
130
PARP
95
* Numbering refers to bands in the lower third of the image (image includes detection of three blots simultaneously)
Note: original image was rotated

## Slide 2
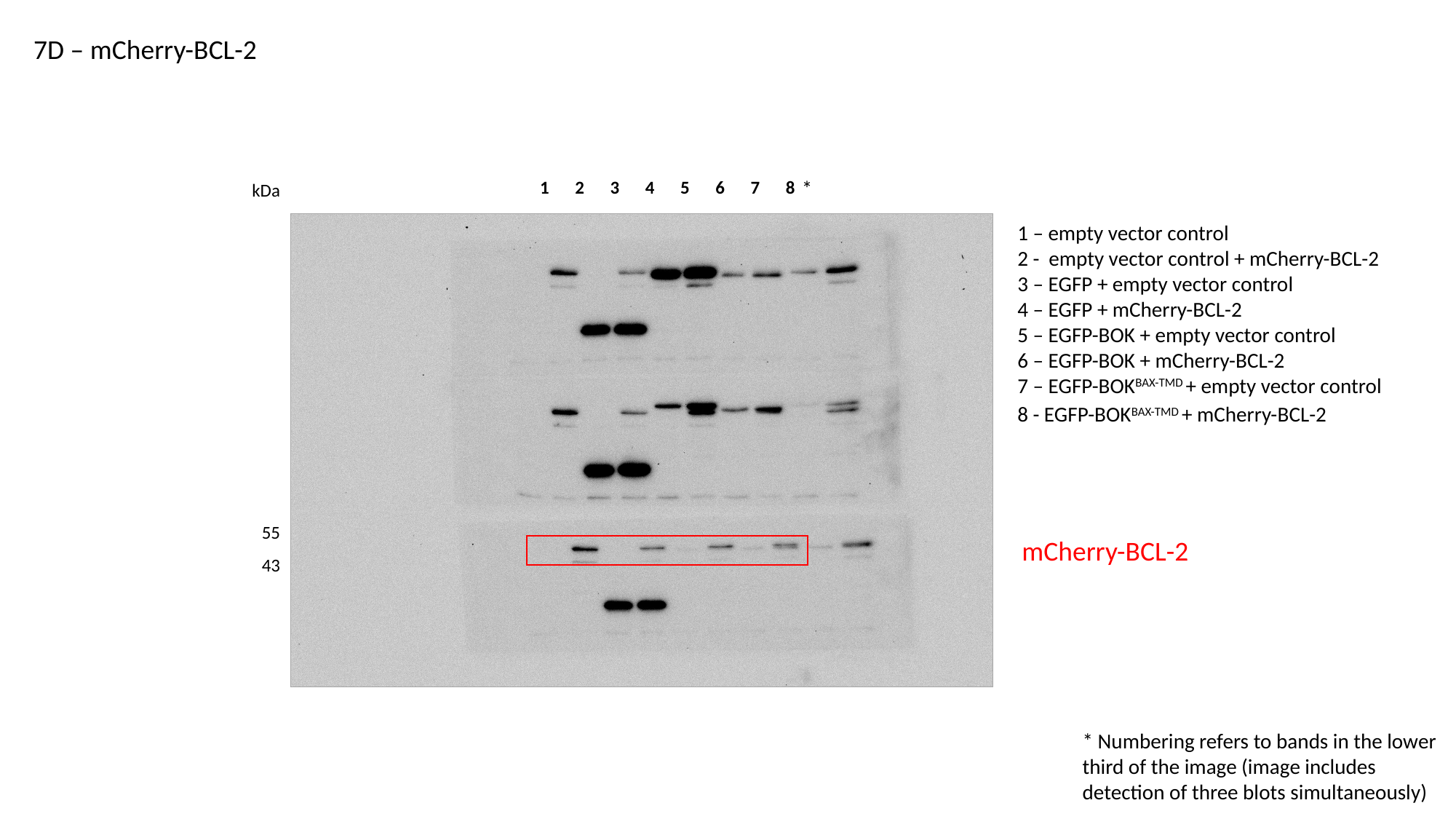

7D – mCherry-BCL-2
*
kDa
| 1 | 2 | 3 | 4 | 5 | 6 | 7 | 8 |
| --- | --- | --- | --- | --- | --- | --- | --- |
1 – empty vector control
2 - empty vector control + mCherry-BCL-2
3 – EGFP + empty vector control
4 – EGFP + mCherry-BCL-2
5 – EGFP-BOK + empty vector control
6 – EGFP-BOK + mCherry-BCL-2
7 – EGFP-BOKBAX-TMD + empty vector control
8 - EGFP-BOKBAX-TMD + mCherry-BCL-2
55
mCherry-BCL-2
43
* Numbering refers to bands in the lower third of the image (image includes detection of three blots simultaneously)

## Slide 3
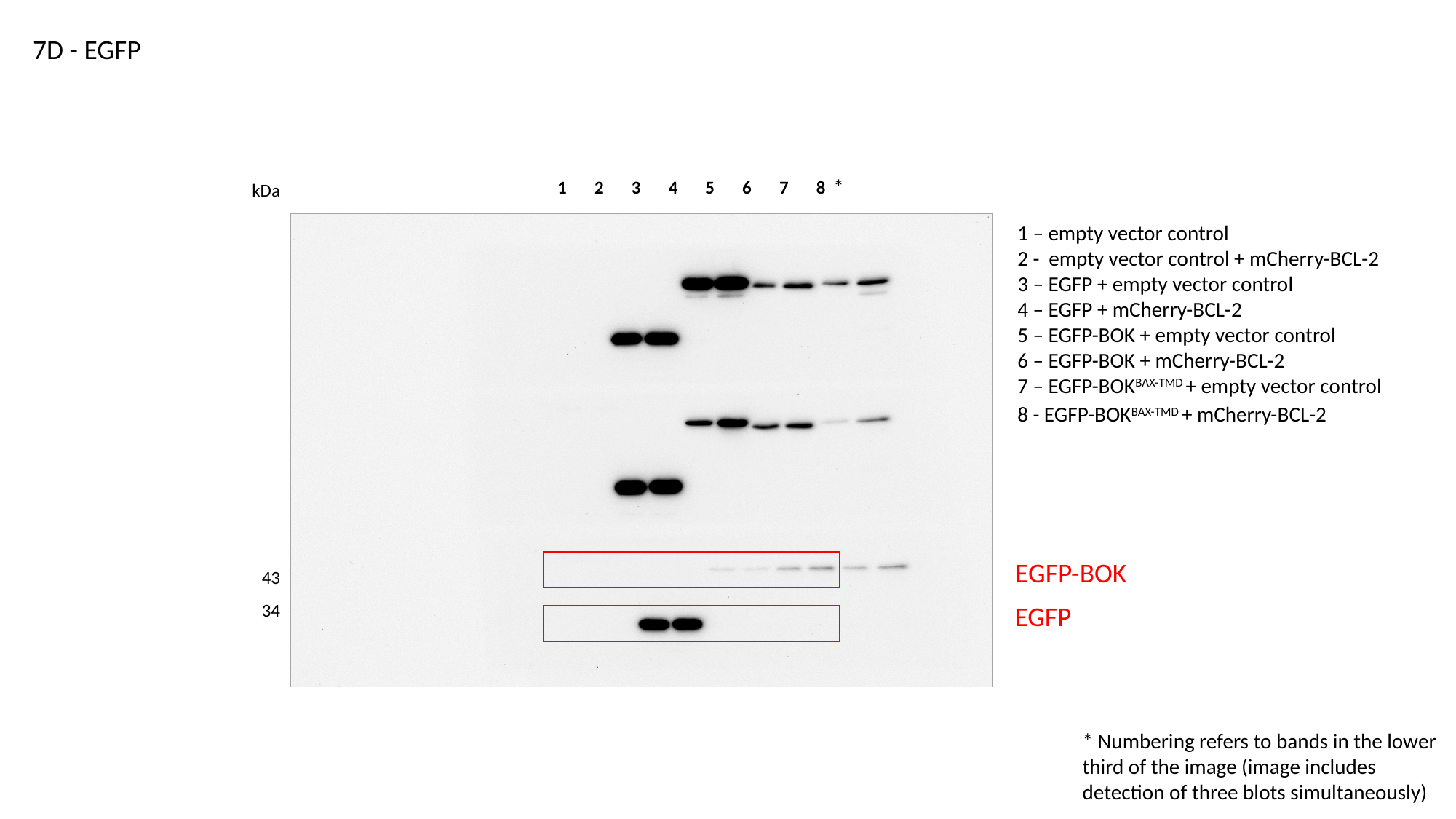

7D - EGFP
*
kDa
| 1 | 2 | 3 | 4 | 5 | 6 | 7 | 8 |
| --- | --- | --- | --- | --- | --- | --- | --- |
1 – empty vector control
2 - empty vector control + mCherry-BCL-2
3 – EGFP + empty vector control
4 – EGFP + mCherry-BCL-2
5 – EGFP-BOK + empty vector control
6 – EGFP-BOK + mCherry-BCL-2
7 – EGFP-BOKBAX-TMD + empty vector control
8 - EGFP-BOKBAX-TMD + mCherry-BCL-2
EGFP-BOK
43
34
EGFP
* Numbering refers to bands in the lower third of the image (image includes detection of three blots simultaneously)

## Slide 4
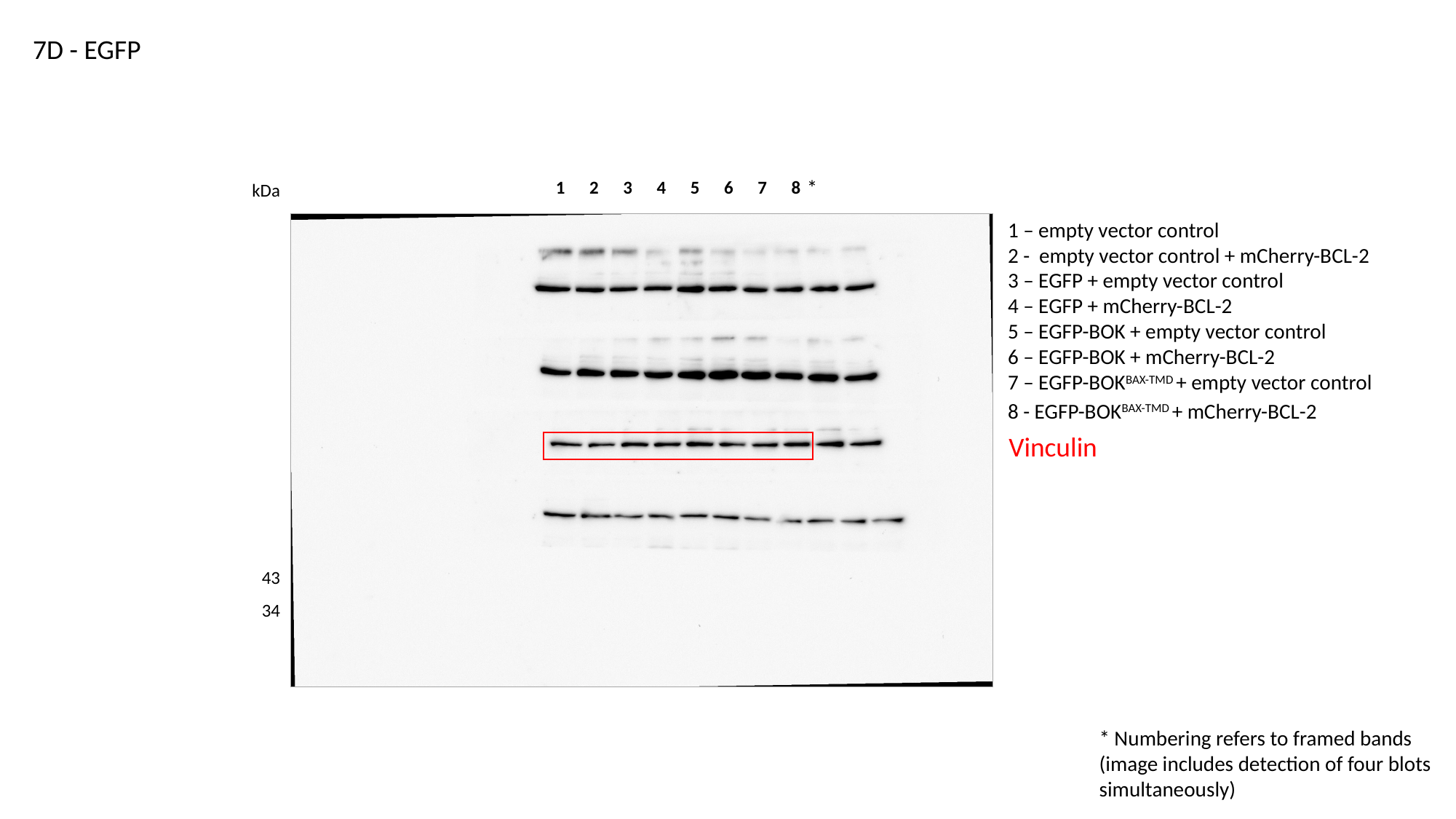

7D - EGFP
*
kDa
| 1 | 2 | 3 | 4 | 5 | 6 | 7 | 8 |
| --- | --- | --- | --- | --- | --- | --- | --- |
1 – empty vector control
2 - empty vector control + mCherry-BCL-2
3 – EGFP + empty vector control
4 – EGFP + mCherry-BCL-2
5 – EGFP-BOK + empty vector control
6 – EGFP-BOK + mCherry-BCL-2
7 – EGFP-BOKBAX-TMD + empty vector control
8 - EGFP-BOKBAX-TMD + mCherry-BCL-2
Vinculin
43
34
* Numbering refers to framed bands (image includes detection of four blots simultaneously)
