# Supplementary material for: BCL-2 and BOK regulate apoptosis by interaction of their C-terminal transmembrane domains
Source: EMBO Rep. 2024 Jul 24;25(9):12. doi: 10.1038/s44319-024-00206-6 (PMC11387410; doi:10.1038/s44319-024-00206-6)
Supplement: Supplementary file 10 — Expanded View Figures [file 44319_2024_206_MOESM10_ESM.pdf]

Expanded View Figures

Figure EV1. (belonging to Fig. 1): A bimolecular split luciferase assay reveals that TMDs of BCL-2 interact with TMDs of BOK but not with TMDs of BAX or BAK.

(**A**) Common structure of Bcl-2 family proteins. Shown are the BH (=Bcl-2 homology) motifs and TMD as boxes. Black dashed line indicates non-proportional representation. Position of alpha helices (α1-9) are indicated in dotted, light gray lines below. (**B**) Proportional co-expression of mCitrine and mTurquoise2. Scatter plots show mCitrine/mTurquoise2 fluorescence intensity detected in individual HEK293FT cells transfected with indicated LgBiT- and/or SmBiT-TMDs. Cells were analyzed 24 h after transfection using flow cytometry. Representative graphs out of three independent experiments. (**C**) Titration of HEK293FT cells transfected with increasing amounts of plasmid for the expression of SmBiT-BAX-TMD, while keeping co-transfected LgBiT-BAX-TMD-encoding plasmid constant. To achieve equal total amount of DNA per transfection, SmBiT-BAX-TMD was mixed with empty backbone vector. Cells were harvested 24 h post transfection and subjected to both split luciferase assay as well as Western blot. Luminescence intensities are shown as mean ± sd of technical triplicates from one representative experiment relative to transfection without SmBiT-BAX-TMD-encoding plasmid. Detection of LgBiT and mTurquoise2 (mTurq2) expression in same samples using Western blot is shown below. β-actin was used as loading control. (**D**) Luminescence of HEK293FT cells co-transfected with plasmids for the expression of LgBiT-BAX-TMD and SmBiT-TOM5-TMD or SmBiT-BAX-TMD, harvested after 24 h and subjected to the split luciferase assay. Detected luminescence was normalized to simultaneously acquired mTurquoise2 fluorescence (mTurq2) or mCitrine fluorescence (mCitr). Mean ± sd from three independent experiments shown in relation to simultaneously detected positive control (BAX-TMD/BAX-TMD, set to 1.00). (**E**) Split luciferase assay confirming BOK-TMD/BCL-2-TMD interaction in MCF-7 cells. MCF-7 cells were transfected with plasmids for the expression of indicated NanoBiT-TMD fusion proteins and harvested after 24 h to be subjected to NanoBiT assay. Luminescence intensities shown as mean ± sd from three independent experiment were set in relation to positive control (BAX-TMD/BAX-TMD). ****$p < 0.0001$, one-way ANOVA with Tukey's multiple comparison test.

© The Author(s)

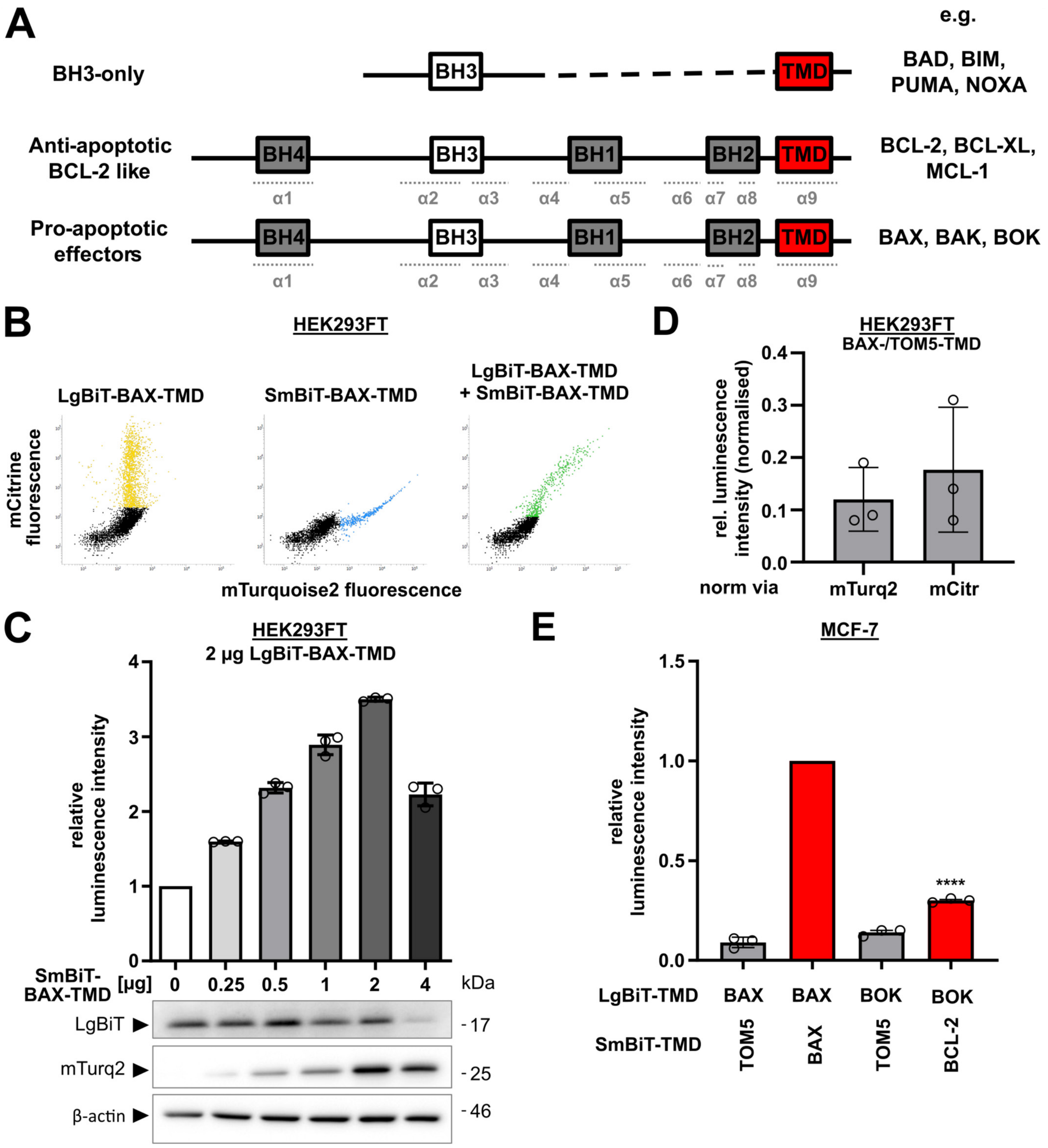
A
e.g.
BH3-only
BH3
TMD
BAD, BIM,
PUMA, NOXA
Anti-apoptotic
BCL-2 like
BH4
BH3
BH1
BH2
TMD
BCL-2, BCL-XL,
MCL-1
α1 α2 α3 α4 α5 α6 α7 α8 α9
Pro-apoptotic
effectors
BAX, BAK, BOK
B
HEK293FT
LgBiT-BAX-TMD
SmBiT-BAX-TMD
LgBiT-BAX-TMD
+ SmBiT-BAX-TMD
mCitrine
fluorescence
mTurquoise2 fluorescence
D
HEK293FT
BAX-/TOM5-TMD
rel. luminescence
intensity (normalised)
norm via
mTurq2
mCitr
C
HEK293FT
2 µg LgBiT-BAX-TMD
relative
luminescence intensity
SmBiT-
BAX-TMD
[µg]
0
0.25
0.5
1
2
4
kDa
LgBiT
mTurq2
β-actin
- 17
- 25
- 46
E
MCF-7
relative
luminescence intensity
****
LgBiT-TMD
BAX
BAX
BOK
BOK
SmBiT-TMD
TOM5
BAX
TOM5
BCL-2

 © The Author(s)

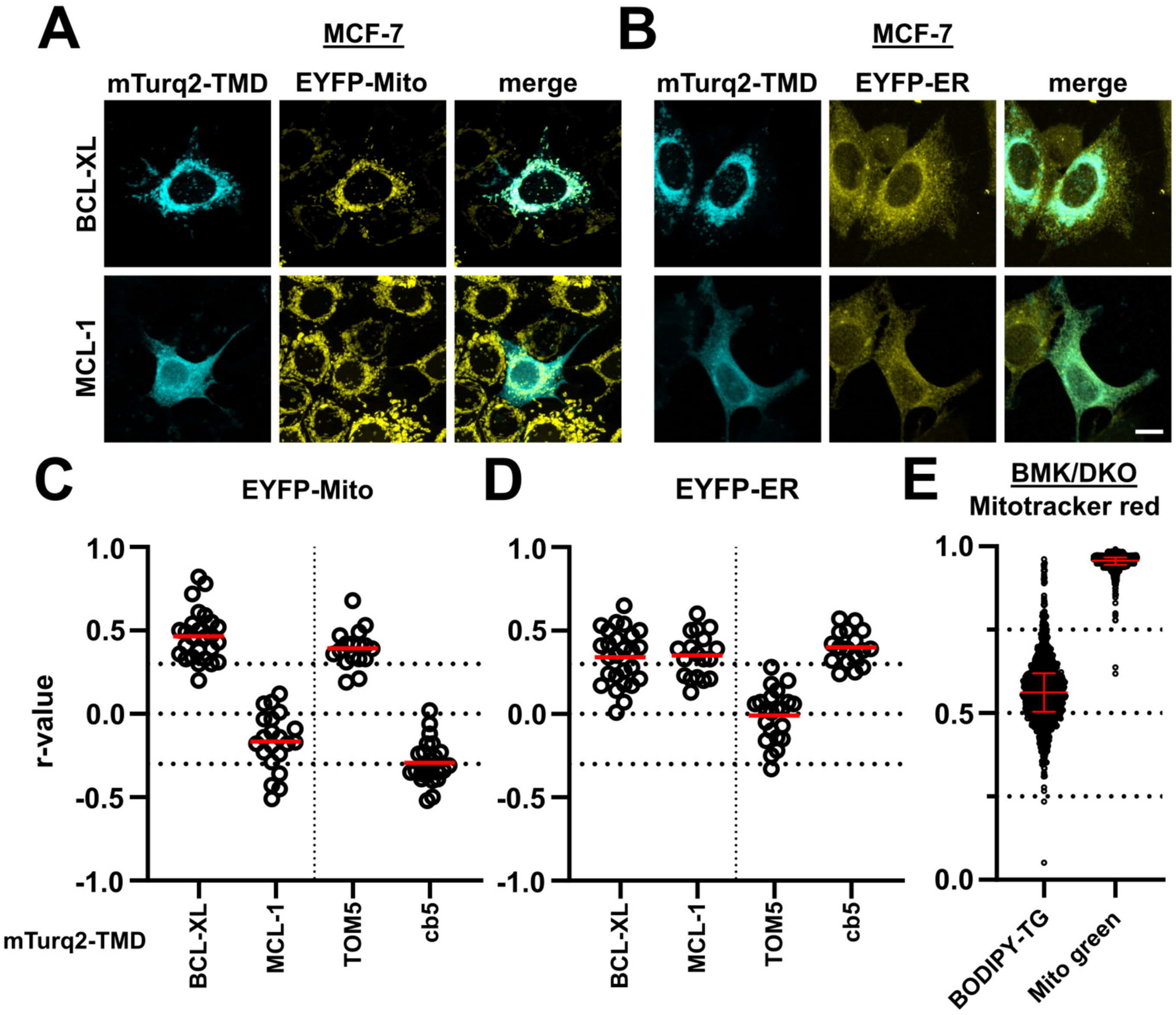

Figure EV2. (belonging to Fig. 2): BCL-2-TMD and BOK TMD are predominantly ER-localized.

(**A**, **B**) Subcellular localization of MCL-1-TMD and BCL-XL-TMD. MCF-7 cells expressing (**A**) EYFP-Mito or (**B**) EYFP-ER were transfected with plasmids for the expression of mTurquoise2 (mTurq2)-labeled BCL-XL-TMD or MCL-1-TMD and 24 h post transfection cells were imaged by cLSM. Images are maximum projections of representative z-stacks from three independent experiments. Scale bar = 10 µm. (**C**, **D**) Quantitative analysis of mTurquoise2-fused BCL-XL-TMD or MCL-1-TMD co-localization with (**C**) EYFP-labeled mitochondria and (**D**) EYFP-labeled ER from cLSM images (**A**, **B**). Graphs show Pearson's r correlation coefficient for a total of ≥15 cells combined from three independent experiments. The mean is marked as a red horizontal line. Data for mTurquoise-TOM5-TMD and mTurquoise2-cb5-TMD are shown for comparison. (**E**) Co-localization analysis of subcellular markers in BMK/DKO cells. BMK/DKO cells labeled with DRAQ5 (nuclei) and Mitotracker red were stained with BODIPY-Thapsigargin (BODIPY-TG, ER control) or Mitotracker green (Mito control). Images of $n \geq 1000$ cells were acquired using an Opera Phenix spinning disk system, images were analyzed and Pearson's r calculated. Shown in red are the median + IQR.

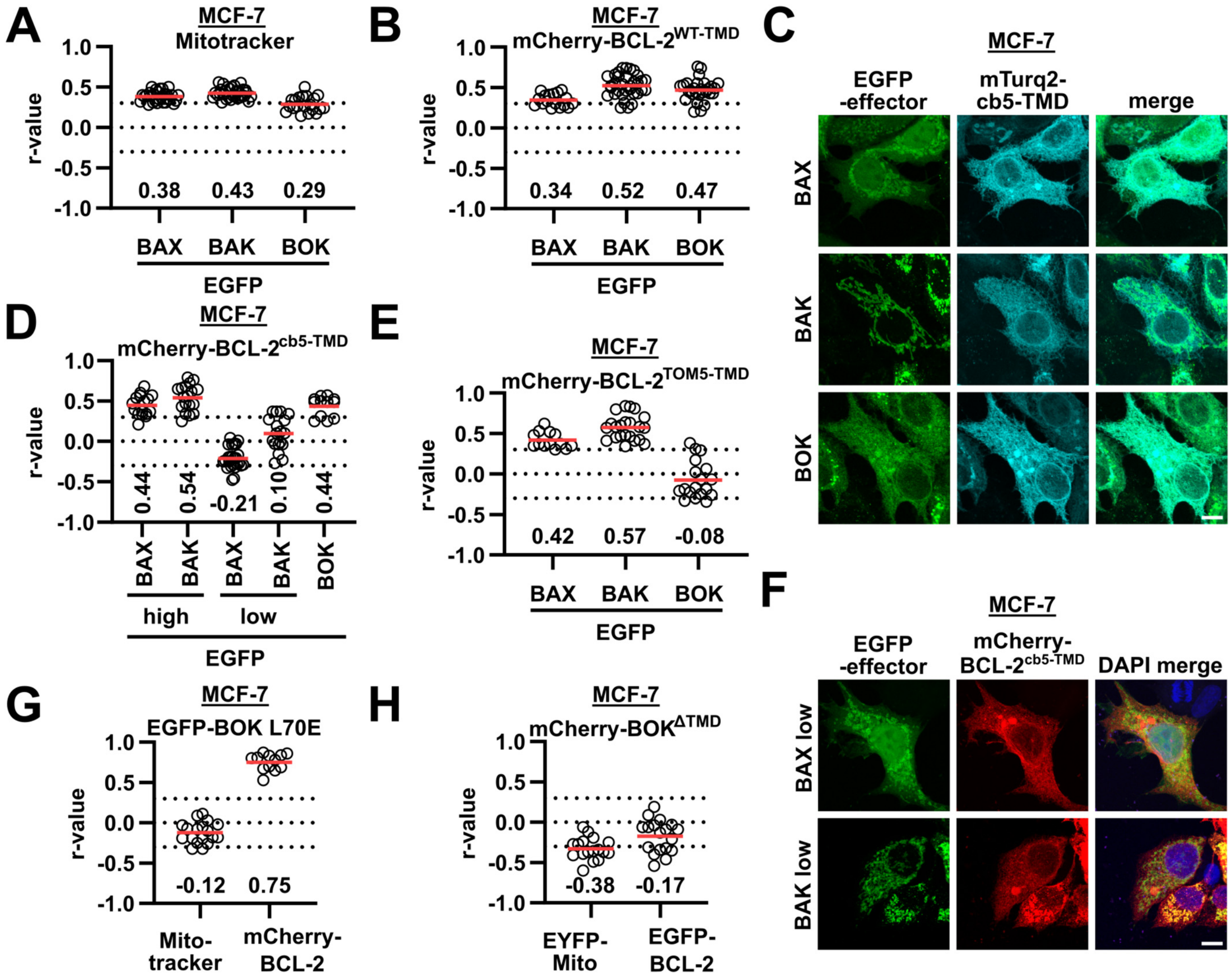

Figure EV3. (belonging to Fig. 4): BOK and BCL-2 co-localization at the ER is dictated by their TMDs.

(**A**, **B**) Quantitative analysis of co-localization from middle sections of cLSM images as shown in Fig. 4C,D. Scatter plots show Pearson's r correlation coefficients of in total ≥10 single cells from two independent experiments determined using Fiji software. Mean is marked as a red horizontal line. Co-localization between EGFP-effectors (BAX, BAK, and BOK) and Mitotracker (**A**) or mCherry-BCL-2 (**B**). (**C**) Co-localization of BAX, BAK, and BOK with ER (cb5-TMD). MCF-7 cells were co-transfected with plasmids for the expression of EGFP-BAX, EGFP-BAK or EGFP-BOK and mTurq2-cb5-TMD. cLSM images are maximum projections of z-stacks from two independent experiments. Scale bar = 10 µm. (**D**, **E**) Quantitative analysis of co-localization as in (**A**, **B**) from Fig. 4E,F and Fig. EV3F. Co-localization between EGFP-effectors (BAX, BAK, and BOK) and mCherry-BCL-$2^{cb5\text{-}TMD}$ (**D**) or mCherry-BCL-$2^{TOM5\text{-}TMD}$ (**E**). In (**D**), Co-localization in cells with high/low BAX/BAK expression was analyzed separately. (**F**) Co-localization of BAX and BAK (low expression) with BCL-$2^{cb5\text{-}TMD}$. MCF-7 cells were co-transfected with plasmids for the expression of EGFP-BAX or EGFP-BAK and mCherry-BCL-$2^{cb5\text{-}TMD}$. Shown images represent cells without cluster formation of BAX or BAK. cLSM images are maximum projections of z-stacks from two independent experiments. Scale bar = 10 µm. (**G**, **H**) Quantitative analysis of co-localization as in (**A**, **B**) from Fig. 4G,H. (**G**) Co-localization between EGFP-BOK L70E and Mitotracker red or mCherry-BCL-2. (**H**) Co-localization between mCherry-$BOK^{\Delta TMD}$ and EYFP-Mito or EGFP-BCL-2.

 © The Author(s)

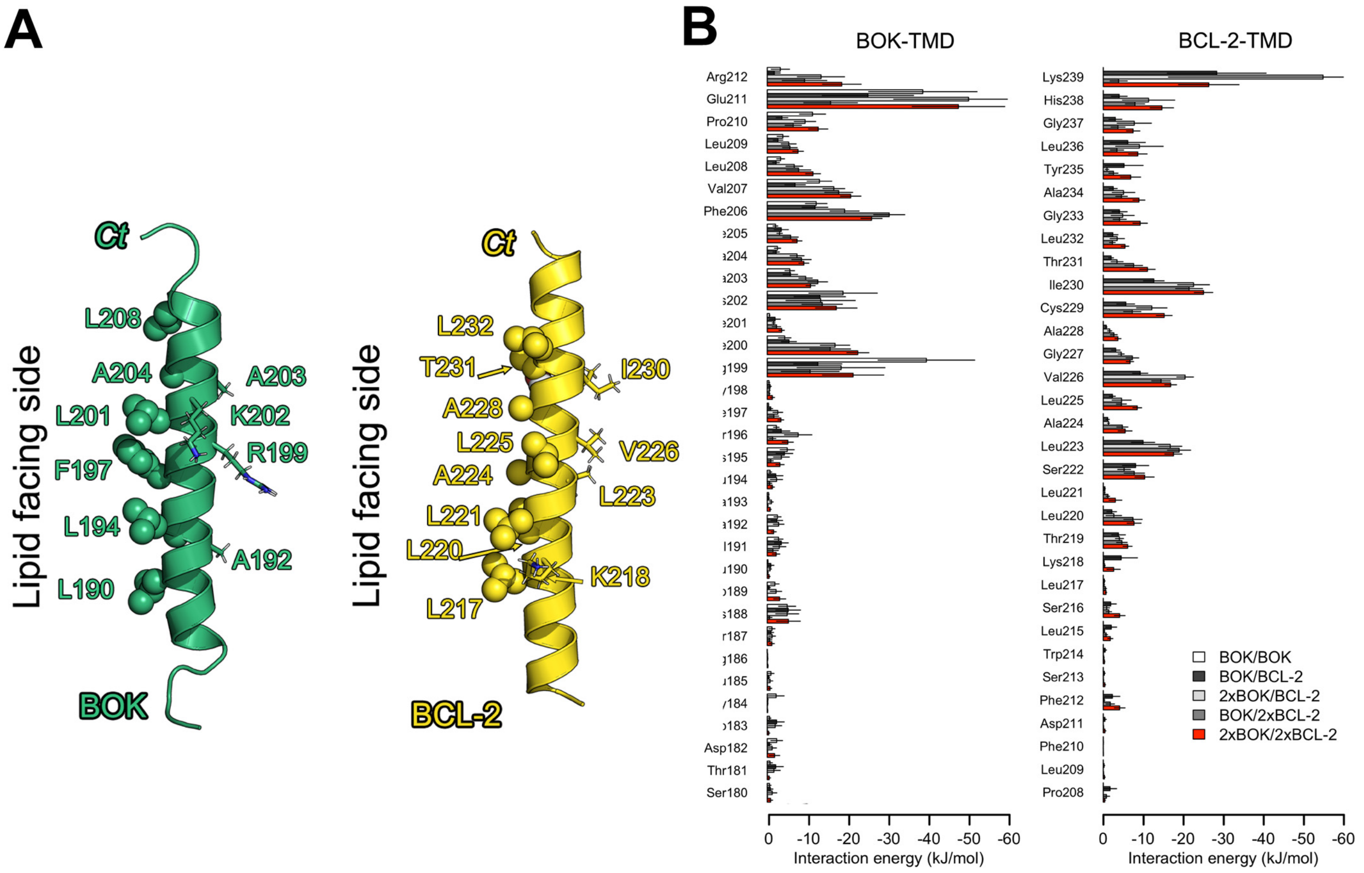

Figure EV4. (belonging to Fig. 6): Interaction of BOK-TMD and BCL-2-TMD depends on specific residues in the interaction interface.

(**A**) Visualization of BCL-2-TMD and BOK-TMD helices with membrane anchors ($K218^{BCL\text{-}2}$ and $R199^{BOK}$ and $K202^{BOK}$) and interaction interface residues ($I230^{BCL\text{-}2}$ $V226^{BCL\text{-}2}$ $L223^{BCL\text{-}2}$, $A203^{BOK}$ $A192^{BOK}$). The lipid facing residues are highlighted as spheres. (**B**) Average interaction energies of each residue of BOK-TMD (left) or BCL-2-TMD (right) with all other peptides over the last 100 ns of AA simulations of dimers, trimer, and tetramers. The error bars denote SEM over individual simulations. The outer error bars of $E211^{BOK}$ in 2xBOK/BCL-2 trimer and $K239^{BCL\text{-}2}$ 2xBOK/BCL-2 trimer simulations are cut at −60 kJ/mol due to visualization purposes.

© The Author(s)

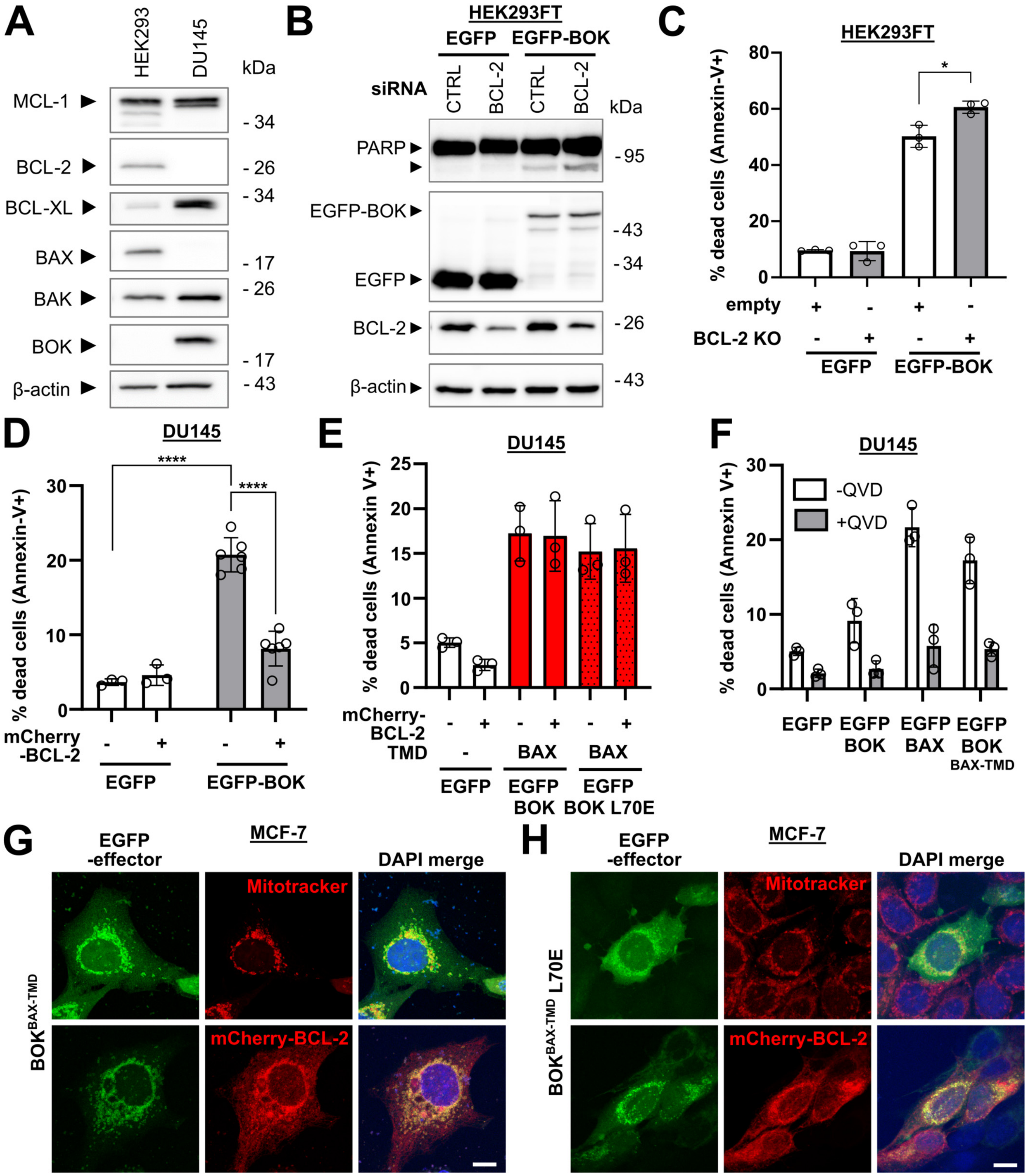
A
HEK293
DU145
kDa
MCL-1
BCL-2
BCL-XL
BAX
BAK
BOK
β-actin
- 34
- 26
- 34
- 17
- 26
- 17
- 43
B
HEK293FT
EGFP
EGFP-BOK
siRNA
CTRL
BCL-2
CTRL
BCL-2
kDa
PARP
EGFP-BOK
EGFP
BCL-2
β-actin
- 95
- 43
- 34
- 26
- 43
C
HEK293FT
% dead cells (Annexin-V+)
empty
BCL-2 KO
EGFP
EGFP-BOK
D
DU145
% dead cells (Annexin-V+)
mCherry -BCL-2
EGFP
EGFP-BOK
E
DU145
% dead cells (Annexin V+)
mCherry-BCL-2
TMD
BAX
BAX
EGFP
EGFP BOK
EGFP BOK L70E
F
DU145
% dead cells (Annexin V+)
-QVD
+QVD
EGFP
EGFP BOK
EGFP BAX
EGFP BOK BAX-TMD
G
EGFP -effector
MCF-7
DAPI merge
Mitotracker
mCherry-BCL-2
BOK BAX-TMD
H
EGFP -effector
MCF-7
DAPI merge
Mitotracker
mCherry-BCL-2
BOK BAX-TMD L70E

Figure EV5. (belonging to Fig. 7): Inhibition of BOK-mediated apoptosis by BCL-2 depends on BOK-TMD.

(**A**) Detection of various Bcl-2 family proteins in whole cell lysates of HEK293FT and DU145 cells using Western blot. β-actin was used as loading control. One independent experiment. (**B**) HEK293FT cells were transfected with siRNA for BCL-2 (siBCL-2) or control siRNA (siCTRL) for 24 h and were subsequently transfected with plasmids for the expression of EGFP-BOK or EGFP. After 18 h, cells were harvested and analyzed by Western blot. PARP, EGFP(-BOK) and BCL-2 expression were detected. β-actin was used as loading control. Representative blot from two independent experiments. (**C**) HEK293FT cells were co-transfected with plasmids for the expression of a CRISPR/Cas9 vector targeting *BCL2* and EGFP or EGFP-BOK (empty = empty vector backbone control). After 18 h, cell death was assessed using Annexin-V-APC staining and flow cytometry. EGFP+/Annexin-V+ cells were detected by flow cytometry. Mean ± sd from three independent experiments. $^{*}p < 0.05$, one-way ANOVA with Tukey's multiple comparison test. (**D**) DU145 cells were transfected with plasmids for the expression of EGFP-BOK or EGFP in combination with mCherry-BCL-2 or an empty vector as a control. After 42 h, cells were stained with Annexin-V-APC and cell death (EGFP+/Annexin-V+ cells) was assessed using flow cytometry. Mean ± sd from three (EGFP) or six (EGFP-BOK) independent experiments. $^{****}p < 0.0001$, one-way ANOVA with Tukey's multiple comparison test. (**E**) DU145 cells were transfected with plasmids for the expression of EGFP, EGFP-BOK$^{BAX\text{-}TMD}$, or EGFP-BOK$^{BAX\text{-}TMD}$ L70E in combination with mCherry-BCL-2 or an empty vector as a control. After 18 h, cells were stained with Annexin-V-APC and cell death (EGFP+/Annexin-V+ cells) was assessed using flow cytometry. Mean ± sd from three independent experiments. (**F**) DU145 cells were transfected with plasmids for the expression of EGFP, EGFP-BOK, EGFP-BAX and EGFP-BOK$^{BAX\text{-}TMD}$ and incubated with 10 μM pan-caspase inhibitor QVD-OPh (+QVD) or DMSO as control (-QVD). After 18 h, cells were stained with Annexin-V-APC and cell death (EGFP+/Annexin-V+ cells) was assessed using flow cytometry. Mean ± sd from three independent experiments. (**G**, **H**) MCF-7 cells were transfected with plasmids for the expression of either EGFP-BOK$^{BAX\text{-}TMD}$ (**G**) or EGFP-BOK$^{BAX\text{-}TMD}$ L70E (**H**). Cells were either stained with Mitotracker red after 24 h or co-transfected to express mCherry-BCL-2. Twenty-four hours post transfection cells were fixed and analyzed by cLSM. Images are maximum projection of z-stacks representative of two independent experiments. Scale bar = 10 μm.

© The Author(s)
